# The Critical Role Of VP1 In Forming The Necessary Cavities For Receptor-mediated Entry Of FMDV To The Host Cell

**DOI:** 10.1038/srep27140

**Published:** 2016-06-02

**Authors:** Jahanshah Ashkani, D. J. G. Rees

**Affiliations:** 1 Agricultural Research Council, Biotechnology Platform, Private Bag X5, Onderstepoort, 0110, South Africa

## Abstract

The antigenic inconsistency of the foot-and-mouth disease virus (FMDV) is very broad, such that a vaccine made from one isolate will not offer protection against infection with other isolates from the same serotype. Viral particles (VPs) or surface exposed capsid proteins, VP1–VP3, of FMDV determine both the antigenicity of the virus and its receptor-mediated entry into the host cell. Therefore, modifications of these structural proteins may alter the properties of the virus. Here we show putative cavities on the FMDV-SAT1 (FMDV Southern African Territories1) capsid as possible binding sites for the receptor-mediated viral entry into the host cell. We identified three possible cavities on the FMDV capsid surface, from which the largest one (C2) is shaped in the contact regions of VP1–VP3. Our results demonstrate the significance of VP1, in the formation of FMDV-SAT1 surface cavities, which is the main component in all the identified cavities. Our findings can have profound implications in the protein engineering of FMDV in the contact region of VP1–VP3 found to be embedded in several cavities. Such information is of great significance in the context of vaccine design, as it provides the ground for future improvement of synthetic vaccines to control FMD caused by FMDV-SAT1 serotypes.

FMDV with a single-stranded (ss) positive-sense RNA genome of about 8.4 kb, belongs to the genus *Aphthovirus* in the family *Picornaviridae*^1^,^2^,^3^. FMD is an extremely infectious disease of livestock^4^,^5^,^6^, while hand-foot-and-mouth disease (HFMD) virus, another subfamily in the family *Picornaviridae,* has shown several outbreaks of the disease in humans^7^,^8^,^9^,^10^,^11^. FMD is mostly spread by direct and indirect animal contacts in specific areas while the movement of infected animals across international borders might be a major reason for the spatial epidemiology of FMD^12^.

Among FMDVs (*i.e.* O, A, C, SAT1, SAT2, SAT3 and Asia-1), SAT types exhibit large inter- and intra-serotype genetic variability^13^,^14^. SAT1, SAT2, and SAT3 are most widely represented in the southern Africa. The RNA genome of FMDV is enclosed within a protein shell or capsid, consisting of 60 copies each of four proteins (VP1–VP4)^15^. The three surface-exposed proteins, VP1–VP3, assemble into a protein trimeric complex, with the smaller VP4 located internally^2^. Capsids are broadly classified according to their structures, mainly as helical or icosahedral^16^,^17^.

Capsids should be stable to protect the virus from stresses in the extracellular space during transmission from a host cell to another host cell. However, upon entry into a new host cell, particles fall apart to deliver their genetic materials to the site of replication with minimal energies^18^,^19^. Viruses have evolved such that they use a broad range of cell-surface molecules as their receptors, which include proteins, carbohydrates, and glycolipids^20^. Icosahedral viruses have a cavity/cleft on the surface of their capsids that can recognize the receptor. It has further been suggested that having the receptor-binding site covered by a cavity would permit the virus to escape host immune surveillance because only long and narrow molecules could bind to conserved amino acids within the cavities^21^,^22^ and the residues that bind to the receptor are protected from immune attack^20^. As a result, antiviral strategies aim to target molecules in these structures and the processes they facilitate, in order to block viral infection, hence prevent or treat viral diseases^23^.

## Results and Discussion

**Figure 1:**
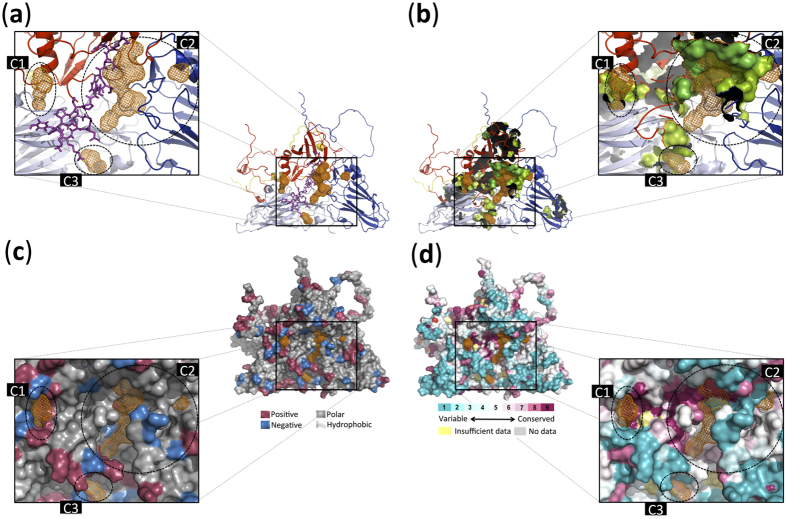
Illustration of the identified protein cavities in FMDV-SAT1 capsid using molecular dynamic simulation and structure-based analysis tools. (**a**) Cartoon representation of VP1–VP4 subunits of FMDV-SAT1 capsid (coloured in red, light blue, blue and yellow, respectively) and the location of identified cavities using molecular dynamic simulation technique (shown in orange mesh). Sticks representation of the predicted VP1 G-H loop and beyond (residues 132–174) containing the major antigenic site for FMDV-SAT1 as well as residues in the H-I loop by Reeve and colleagues^24^, coloured in purple. (**b**) Cartoon representation of VP1–VP4 subunits of the FMDV-SAT1 capsid (coloured in red, light blue, blue and yellow, respectively), the location of identified cavities using molecular dynamic simulation method and the surface presentation of identified residues involved in cavities using structure-based analysis tools (residues identified by 7, 8, 9 and more tools are coloured in lemon, light green and green). (**c**) Surface representation of the positively and negatively charged (coloured in red and blue respectively), polar and hydrophobic (coloured in grey and light grey respectively) residues. (**d**) Surface representation of evolutionarily conserved residues identified using ConSurf-DB^42^. C1–C3 refer to cavity 1-cavity 3 and Pymol package^40^ was used to represent the molecules.

Three major cavities (C1–C3) are proposed as consensus sites identified by seven or more prediction tools (Fig. 1). Additionally, to complement and confirm the identified cavities we performed a Molecular Dynamic (MD) simulation analysis using GROMACS and the resulting 1000 snapshots taken from a 50 ns MD trajectory were further assessed using MDpocket.

The combination of the consensus result of ten different structural-based cavity prediction tools with the results of the molecular dynamic simulation shows that the predicted cavities are placed in the contact region of VP1, VP2, and VP3. Furthermore, two small cavities, 1 and 3 (C1 and C3) are shaped in the contact regions of VP1 and VP2, and VP1 and VP3, respectively. Additionally, a large cavity (C2) is shaped in the contact regions of all three surface-exposed proteins (VP1–VP3).

**Figure 2:**
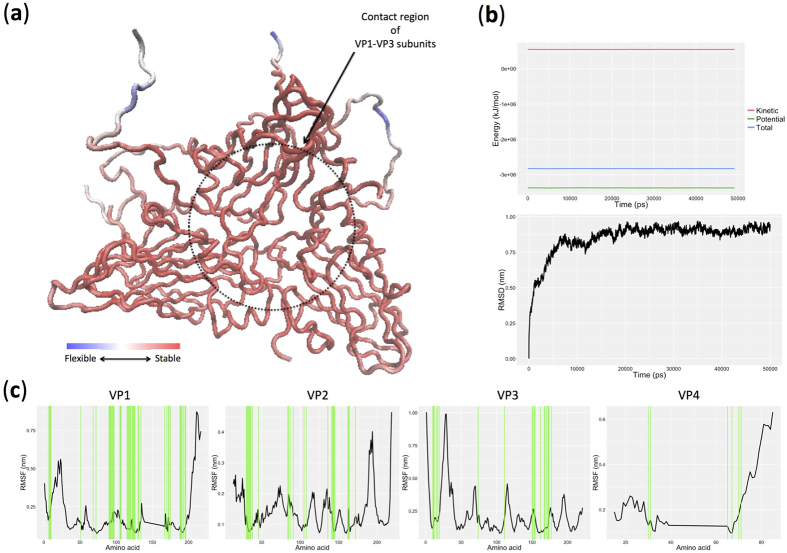
System stability and root mean square fluctuation (RMSF) analysis of FMDV-SAT1 capsid subunits (VP1–VP4). (**a**) Root mean square division per residue (RMSDres) in FMDV-SAT1 capsid calculated from 50 ns simulations. Residue stability increases from blue to red. (**b**) Energy stability (top plot) and Root mean square division, RMSD, (bottom plot) of the system throughout the 50 ns simulation time. (**c**) Root mean square fluctuation of residues (RMSF) of FMDV-SAT1 capsid viral particles (VP1–VP4) throughout the 50 ns simulation time. Identified residues that are involved in cavities in FMDV-SAT1 capsid viral particles (VP1–VP4) using ten structure-based analysis tools were illustrated using green vertical lines. GROMACS package was used for simulations and trajectory analysis while VMD^44^ and R^45^ package were used for the representation of the molecule and drawing the plots respectively.

Mapping the results of MDpocket (presented as orange mesh) on the FMDV-SAT1 capsid structure indicates that the identified cavities are placed in the contacting loop regions of VP1–VP3 surrounding major antigenic sites identified by Reeve and colleagues (2010) for FMDV-SAT1^24^ (Fig. 1a), which is ideally situated to form receptor-binding sites. Furthermore, there is a clear overlap between the cavities identified by MDpocket and the three identified cavities using ten different structure-based cavity prediction tools (Fig. 1b). In addition, the interior of the identified cavities, especially C1 and C2 are extremely charged (Fig. 1c) and conserved (Fig. 1d), accentuating their possible implications for receptor binding. It can, therefore, be speculated that VP1 is indispensable for the formation of these cavities due to it being the main component in all the identified pockets (Fig. 2) while contributing the maximum number of residues in the identified cavities compared to VP2, VP3, and VP4 (Table 1S).

Considering that cavity with putative roles in the receptor-mediated entry of FMDV to the host cell should maintain a stable structural conformation in the extracellular environment, we further assessed the root mean square fluctuations (RMSF) of the amino acids at the identified cavities. Accordingly, after confirming the system stability through the simulation, the RMSDs per residue (RMSDres) were calculated along the 50 ns trajectory (Fig. 2). Our results indicate that the contact region of VP1–VP3 subunits is the most stable part of the FMDV-SAT1 capsid (Fig. 1a), which could act as a probable receptor-binding site whose modifications may alter viral entry into the host cell^25^. However, considering the identified cavities are in the loop region, a greater amino acid fluctuation would have been expected, but this can be explained by the possible accumulation of smaller cavities to give rise to the larger cavity identified here. This is consistent with the dynamic nature of cavity formation^26^.

## Conclusion

FMD is a highly contagious disease, affecting mammals. In the context of viral structural features, it is known that the VP4 is internally located within the capsid, whereas VP1, VP2 and VP3 are surface exposed, contributing towards viral antigenicity^27^,^28^. There are at least two immunogenic sites in VP1, the G-H loop and the C-terminus^29^,^30^. However, it has been suggested that the virus can infect cells through alternative approaches as well. Furthermore, viral mechanisms of receptor recognition can evolve over the course of infection leading to the emergence of viruses with different receptor binding specificities^31^.

While considerable knowledge exists about the FMDV, the disease remains a major threat worldwide. As the virus evolves, novel and resistant strains are produced resulting in disease epidemic. It is therefore of great importance to further understand viral mechanisms of infection in search of alternative preventive methods and targets. To this end, we present novel findings as putative targets for future research. Here we have identified novel cavities in the capsid of FMDV located in the loop regions of viral particles, VP1–VP3, consisting of highly charged and conserved amino acids. The structural features identified here and the stability of their structures allows for speculating a role for the identified cavities in receptor-mediated virulence, the exact system of which is in need of further research. We propose the identified cavities hold the binding sites required for receptor-mediated viral entry into the host cell while protecting them from immune system recognition. Our results further suggest that VP1 contributes the most to the interaction with the host cells through the identified cavities on its surface. This information can be widely used in protein engineering of FMDV-SAT1 with emphasize on the possible improvement of synthetic vaccines formulation, hence controlling FMD caused by FMDV-SAT1 serotypes.

## Methods

The protein structure of the FMDV-SAT1 capsid was retrieved from the Protein Data Bank (PDB) with the identification code of 2WZR. The 2WZR PDB file, containing VP1–VP4 chains, was used as input for structure-based prediction tools and a template for using the construction of the initial structure for the simulation studies. As the identification of cavities is the starting point for structure-based vaccine design, the consensus result of 10 different structure-based pocket/cavity prediction tools was used to improve the prediction success rates (Table 1).

GROMACS package^32^ implementing SPC/E water model^33^ and the GROMOS96 43A1 force field^34^ was used for molecular dynamic (MD) simulation on a neutralized system containing water molecules and sodium ions (Na+). The LINCS and the SETTLE algorithms were used to constrain protein covalent bonds involving hydrogen atoms and to maintain the rigid structure of the water molecules, respectively^35^,^36^. The temperature and pressure, regulated by the Berendsen’s algorithms^37^, was at 300 K and 1.0 Atm, respectively. To control the temperature, a minimal invasive thermostat was applied. A 1.0 nm cutoff in the interactions was utilized, and the particle mesh Ewald summation method^38^,^39^ was applied to estimate the long-range electrostatic interactions. An energy minimization phase, using a steepest descent algorithm, was applied to initiate the simulation while removing bad contacts and unfavorable forces. Six simulations of 10 ps were conducted, by increasing the temperature from 50–300 K for the equilibration of each system. Finally, the simulation of the system was performed at 50 ns using GROMACS. For the purpose of protein structure visualization, the PyMol package^40^ was used. The protein pockets were detected using the MDpocket package^41^, which is based on the geometric α-sphere theory, with specific parameters to detect small molecule binding sites.

Table 1 shows the list of the ten most popular structure-based pocket/cavity prediction tools that were used for the prediction of cavities in FMDV-SAT1 capsid surface. The results of these analyses are shown in Fig. 1 while cavity contributor amino acids that were predicted by 7, 8, 9 and more tools (listed in Table S1) are colored by lemon, light green and green, respectively. ConSurf-DB^42^ was used to map evolutionary conservation scores calculated for each residue from multiple sequence alignment of similar proteins using an empirical Bayesian inference in Rate4Site^43^ (Figs S1–S3).

## Additional Information

**How to cite this article**: Ashkani, J. and Rees, D. J. G. The Critical Role Of VP1 In Forming The Necessary Cavities For Receptor-mediated Entry Of FMDV To The Host Cell. *Sci. Rep.* **6**, 27140; doi: 10.1038/srep27140 (2016).

## Supplementary Material

Supplementary Information

## Figures and Tables

**Table 1:** List of the protein pocket/cavity prediction tools were used in this study.

Tool	Description	Link
CASTp^46^	Computed Atlas of Surface Topography of proteins (CASTp) provides pockets located on protein surfaces and voids buried in the interior of proteins.	http://sts.bioengr.uic.edu/castp/
DEPTH^47^	Depth measures the closest distance of a residue/atom to bulk solvent.	http://mspc.bii.astar.edu.sg/tankp/run_depth.html
DogSiteScorer^48^	An automated pocket detection and analysis tool based on support vector machine, which uses calculated size, shape and chemical features of automatically predicted.	http://dogsite.zbh.uni-hamburg.de/
fpocket^49^	A high performance protein pocket (cavity) detection algorithm based on Voronoi tessellation.	http://bioserv.rpbs.univ-paris-diderot.fr/fpocket/
GHECOM^50^,^51^	A program for finding multi-scale pockets on protein surfaces using mathematical morphology.	http://strcomp.protein.osaka-u.ac.jp/ghecom/
LIGSITEcsc^52^	An automatic detection of pockets on protein surface using the Connolly surface and the degree of conservation.	http://projects.biotec.tu-dresden.de/pocket/
PDBinder^53^	A bioinformatic tool for the prediction of small ligand binding sites, which compares a query protein against a library of binding and non-binding protein surface regions derivative from the PDB.	http://pdbinder.bio.uniroma2.it/PDBinder/
Pocket-finder	Pocket-Finder is based on the Ligsite algorithm written by Hendlich *et al*.^54^.	http://www.modelling.leeds.ac.uk/pocketfinder/
Q-SiteFinder^55^	An energy-based method for the prediction of protein-ligand binding sites.	http://www.modelling.leeds.ac.uk/qsitefinder/
SiteHound^56^	SiteHound finds protein regions that are probable to interact with ligands by computing interactions between a chemical probe and a protein structure.	http://scbx.mssm.edu/sitehound/sitehound-download/download.html

## References

[b1] Lohse L., Jackson T., Bøtner A. & Belsham G. J. Capsid coding sequences of foot-and-mouth disease viruses are determinants of pathogenicity in pigs. Vet Res 43, 46–46 (2012). 22624592 10.1186/1297-9716-43-46 PMC3431240

[b2] Sobrino F. . Foot-and-mouth disease virus: a long known virus, but a current threat. Vet Res 32, 1–30 (2001). 11254174 10.1051/vetres:2001106

[b3] Domingo E., Baranowski E., Escarmís C. & Sobrino F. Foot-and-mouth disease virus. Comp Immunol Microb 25, 297–308 (2002). 10.1016/s0147-9571(02)00027-9 12365806

[b4] Kim H. B., Kim S. C., Lee S. I. & Kim I. H. Attenuation of the adverse effects caused by the foot-and-mouth disease vaccination in pigs. Vet Rec 177, 494 (2015). 26553554 10.1136/vr.103231

[b5] Bachrach H. L. Foot-And-Mouth Disease. Annu Rev Microbiol 22, 201–244 (1968). 4301615 10.1146/annurev.mi.22.100168.001221

[b6] Paton D., Valarcher J.-F., Bergmann I., Matlho O. & Zakharov V. Selection of foot and mouth disease vaccine strains–a review. Rev Sci Tech Off Int Epiz 24, 981–993 (2014). 16642769

[b7] Mirand A. . Outbreak of hand, foot and mouth disease/herpangina associated with coxsackievirus A6 and A10 infections in 2010, France: a large citywide, prospective observational study. Clin Microbiol Infect 18, E110–E118 (2012). 22404077 10.1111/j.1469-0691.2012.03789.x

[b8] Lu Q.-B. . Circulation of Coxsackievirus A10 and A6 in hand-foot-mouth disease in China, 2009–2011. PLoS One 7, e52073 (2012). 23272213 10.1371/journal.pone.0052073 PMC3525556

[b9] Hu M. . Determinants of the incidence of hand, foot and mouth disease in China using geographically weighted regression models. PLoS One 7, e38978 (2012). 22723913 10.1371/journal.pone.0038978 PMC3377651

[b10] Puenpa J. . Hand, foot, and mouth disease caused by coxsackievirus A6, Thailand, 2012. Emerg Infect Diseases 19, 641 (2013). 23631943 10.3201/eid1904.121666 PMC3647428

[b11] Sankar A., Samatha Y., Ravi Kiran A. & Poornachandra N. Hand Foot and Mouth Disease. J Bioeng Biomed Sci 5, 2 (2015).

[b12] Richards K. . Using exceedance probabilities to detect anomalies in routinely recorded animal health data, with particular reference to foot-and-mouth disease in Viet Nam. Spat Spatiotemporal Epidemiol 11, 125–133 (2014). 25457601 10.1016/j.sste.2014.08.002

[b13] Bastos A. S. . Molecular Epidemiology of SAT3-Type Foot-and-Mouth Disease. Virus Genes 27, 283–290 (2003). 14618089 10.1023/a:1026352000959

[b14] Bastos A. . The implications of virus diversity within the SAT 2 serotype for control of foot-and-mouth disease in sub-Saharan Africa. J Gen Virol 84, 1595–1606 (2003). 12771430 10.1099/vir.0.18859-0

[b15] Belsham G. Translation and replication of FMDV RNA. Curr Top Microbiol Immunol 288, 43–70 (2005). 15648174 10.1007/3-540-27109-0_3

[b16] Lidmar J., Mirny L. & Nelson D. R. Virus shapes and buckling transitions in spherical shells. Phys Rev E 68, 051910 (2003). 10.1103/PhysRevE.68.051910 14682823

[b17] Vernizzi G. & Olvera de la Cruz M. Faceting ionic shells into icosahedra via electrostatics. Proc Natl Acad Sci 104, 18382–18386 (2007). 18003933 10.1073/pnas.0703431104 PMC2141786

[b18] Marsh M. & Helenius A. Virus Entry: Open Sesame. Cell 124, 729–740 (2006). 16497584 10.1016/j.cell.2006.02.007 PMC7112260

[b19] Marjomäki V., Turkki P. & Huttunen M. Infectious Entry Pathway of Enterovirus B Species. Viruses 7, 6387–6399 (2015). 26690201 10.3390/v7122945 PMC4690868

[b20] Rossmann M. G. . Cell Recognition and Entry by Rhino- and Enteroviruses. Virology 269, 239–247 (2000). 10753702 10.1006/viro.2000.0258

[b21] Rossmann M. G. . Structure of a human common cold virus and functional relationship to other picornaviruses. Nature 317, 145–153 (1984). 2993920 10.1038/317145a0

[b22] Rossmann M. G. . Crystallographic and cryo EM analysis of virion-receptor interactions. Arch Virol Suppl 9, 531–541 (1994). 7913361 10.1007/978-3-7091-9326-6_51 PMC4140090

[b23] Lucas W. & Knipe D. M. Viral Capsids and Envelopes: Structure and Function. eLS, e1–7 (2001).

[b24] Reeve R. . Sequence-based prediction for vaccine strain selection and identification of antigenic variability in foot-and-mouth disease virus. PLoS Comput Biol 6, e1001027 (2010). 21151576 10.1371/journal.pcbi.1001027 PMC3000348

[b25] Fuzo C. A. & Degrève L. New pockets in dengue virus 2 surface identified by molecular dynamics simulation. J Mol Model 19, 1369–1377 (2013). 23197323 10.1007/s00894-012-1687-6 PMC3578724

[b26] Kocher J.-P., Prévost M., Wodak S. J. & Lee B. Properties of the protein matrix revealed by the free energy of cavity formation. Structure 4, 1517–1529 (1996). 8994976 10.1016/s0969-2126(96)00157-8

[b27] Lea S. . The structure and antigenicity of a type C foot-and-mouth disease virus. Structure 2, 123–139 (1994). 8081743 10.1016/s0969-2126(00)00014-9

[b28] Thomas A. A., Woortmeijer R. J., Puijk W. & Barteling S. J. Antigenic sites on foot-and-mouth disease virus type A10. J Virol 62, 2782–2789 (1988). 2455819 10.1128/jvi.62.8.2782-2789.1988 PMC253712

[b29] Fox G. . The Cell Attachment Site on Foot-and-Mouth Disease Virus Includes the Amino Acid Sequence RGD (Arginine-Glycine-Aspartic Acid). J Gen Virol 70, 625–637 (1989). 2543752 10.1099/0022-1317-70-3-625

[b30] Jackson T. . Arginine-glycine-aspartic acid-specific binding by foot-and-mouth disease viruses to the purified integrin alpha(v)beta3 *in vitro*. J Virol 71, 8357–8361 (1997). 9343190 10.1128/jvi.71.11.8357-8361.1997 PMC192296

[b31] Mason P. W., Rieder E. & Baxt B. RGD sequence of foot-and-mouth disease virus is essential for infecting cells via the natural receptor but can be bypassed by an antibody-dependent enhancement pathway. Proc Natl Acad Sci 91, 1932–1936 (1994). 8127909 10.1073/pnas.91.5.1932 PMC43278

[b32] Lindahl E., Hess B. & Van Der Spoel D. GROMACS 3.0: a package for molecular simulation and trajectory analysis. J Mol Model 7, 306–317 (2001).

[b33] Lee S. H. & Rasaiah J. C. Molecular dynamics simulation of ion mobility. 2. Alkali metal and halide ions using the SPC/E model for water at 25 C. J Phys Chem 100, 1420–1425 (1996).

[b34] Daura X., Mark A. E. & Van Gunsteren W. F. Parametrization of aliphatic CHn united atoms of GROMOS96 force field. J Comput Chem 19, 535–547 (1998).

[b35] Hess B., Bekker H., Berendsen H. J. C. & Fraaije J. G. E. M. LINCS: A linear constraint solver for molecular simulations. J Comput Chem 18, 1463–1472 (1997).

[b36] Miyamoto S. & Kollman P. A. SETTLE: an analytical version of the SHAKE and RATTLE algorithm for rigid water models. J Comput Chem 13, 952–962 (1992).

[b37] Berendsen H. J. C., Postma J. P. M., van Gunsteren W. F., DiNola A. & Haak J. R. Molecular dynamics with coupling to an external bath. J Chem Phys 81, 3684–3690 (1984).

[b38] Essmann U. . A smooth particle mesh Ewald method. J Chem Phys 103, 8577–8593 (1995).

[b39] Darden T., York D. & Pedersen L. Particle mesh Ewald: An N⋅ log (N) method for Ewald sums in large systems. J Chem Phys 98, 10089–10092 (1993).

[b40] Schrodinger L. L. C. *The PyMOL Molecular Graphics System, Version~1.3r1* (2010).

[b41] Schmidtke P., Bidon-Chanal A., Luque F. J. & Barril X. MDpocket: open-source cavity detection and characterization on molecular dynamics trajectories. Bioinformatics 27, 3276–3285 (2011). 21967761 10.1093/bioinformatics/btr550

[b42] Goldenberg O., Erez E., Nimrod G. & Ben-Tal N. The ConSurf-DB: pre-calculated evolutionary conservation profiles of protein structures. Nucleic Acids Res 37, D323–D327 (2009). 18971256 10.1093/nar/gkn822 PMC2686473

[b43] Pupko T., Bell R. E., Mayrose I., Glaser F. & Ben-Tal N. Rate4Site: an algorithmic tool for the identification of functional regions in proteins by surface mapping of evolutionary determinants within their homologues. Bioinformatics 18, S71–S77 (2002). 12169533 10.1093/bioinformatics/18.suppl_1.s71

[b44] Humphrey W., Dalke A. & Schulten K. VMD: Visual Molecular dynamics. J Mol Graph 14, 33–38 (1996). 8744570 10.1016/0263-7855(96)00018-5

[b45] R-Core-Team. R: A language and environment for statistical computing. R Foundation for Statistical Computing. R Foundation for Statistical Computing, Vienna, Austria. URL http://www.R-project.org/ (2013).

[b46] Dundas J. . CASTp: computed atlas of surface topography of proteins with structural and topographical mapping of functionally annotated residues. Nucleic Acids Res 34, W116–W118 (2006). 16844972 10.1093/nar/gkl282 PMC1538779

[b47] Tan K. P., Nguyen T. B., Patel S., Varadarajan R. & Madhusudhan M. S. Depth: a web server to compute depth, cavity sizes, detect potential small-molecule ligand-binding cavities and predict the pKa of ionizable residues in proteins. Nucleic Acids Res 41, W314–W321 (2013). 23766289 10.1093/nar/gkt503 PMC3692129

[b48] Volkamer A., Kuhn D., Rippmann F. & Rarey M. DoGSiteScorer: a web server for automatic binding site prediction, analysis and druggability assessment. Bioinformatics 28, 2074–2075 (2012). 22628523 10.1093/bioinformatics/bts310

[b49] Le Guilloux V., Schmidtke P. & Tuffery P. Fpocket: An open source platform for ligand pocket detection. BMC Bioinformatics 10, 168 (2009). 19486540 10.1186/1471-2105-10-168 PMC2700099

[b50] Kawabata T. Detection of multiscale pockets on protein surfaces using mathematical morphology. Proteins Struct Funct Bioinf 78, 1195–1211 (2010). 10.1002/prot.22639 19938154

[b51] Kawabata T. & Go N. Detection of pockets on protein surfaces using small and large probe spheres to find putative ligand binding sites. Proteins Struct Funct Bioinf 68, 516–529 (2007). 10.1002/prot.21283 17444522

[b52] Huang B. & Schroeder M. LIGSITEcsc: predicting ligand binding sites using the Connolly surface and degree of conservation. BMC Struct Biol 6, 19 (2006). 16995956 10.1186/1472-6807-6-19 PMC1601958

[b53] Bianchi V., Mangone I., Ferrè F., Helmer-Citterich M. & Ausiello G. webPDBinder: a server for the identification of ligand binding sites on protein structures. Nucleic Acids Res 41, W308–W313 (2013). 23737450 10.1093/nar/gkt457 PMC3692056

[b54] Hendlich M., Rippmann F. & Barnickel G. LIGSITE: automatic and efficient detection of potential small molecule-binding sites in proteins. J Mol Graph Model 15, 359–363 (1997). 9704298 10.1016/s1093-3263(98)00002-3

[b55] Laurie A. T. R. & Jackson R. M. Q-SiteFinder: an energy-based method for the prediction of protein–ligand binding sites. Bioinformatics 21, 1908–1916 (2005). 15701681 10.1093/bioinformatics/bti315

[b56] Hernandez M., Ghersi D. & Sanchez R. SITEHOUND-web: a server for ligand binding site identification in protein structures. Nucleic Acids Res 37, W413–W416 (2009). 19398430 10.1093/nar/gkp281 PMC2703923

