# Supplementary material for: The Critical Role Of VP1 In Forming The Necessary Cavities For Receptor-mediated Entry Of FMDV To The Host Cell
Source: Sci Rep. 2016 Jun 2;6:27140. doi: 10.1038/srep27140 (PMC4890027; doi:10.1038/srep27140)
Supplement: Supplementary Information [file srep27140-s1.pdf]

The Critical Role Of VP1 In Forming The Necessary Cavities For Receptor-mediated Entry Of FMDV To The Host Cell

Jahanshah Ashkani*, D.J.G Rees

Agricultural Research Council, Biotechnology Platform, Private Bag X5, Onderstepoort, 0110, South Africa

*Corresponding author

Email: jahanshah.a@gmail.com

Tel: +27(0)216503982

Supplementary Figures
Figures 1S – 3S
Supplementary Tables
Table 1S

Supplementary Figures

Figure 1S: Multiple sequence alignment (MSA) illustrations of the evolutionary conservation profile of VP1 calculated by ConSurf-DB[1]. Amino acid sequences similar to VP1 sequence in the 2WZR PDB (2WZR1) are retrieved and aligned using CSI-BLAST[2,3] and MAFFT[4], respectively. The MSA is coloured by evolutionarily conservation scores calculated for each residue using an empirical Bayesian inference in Rate4Site[5].

Figure 2S: Multiple sequence alignment (MSA) illustrations of the evolutionary conservation profile of VP2 calculated by ConSurf-DB[1]. Amino acid sequences similar to VP2 sequence in the 2WZR PDB (2WZR2) are retrieved and aligned using CSI-BLAST[2,3] and MAFFT[4], respectively. The MSA is coloured by evolutionarily conservation scores calculated for each residue using an empirical Bayesian inference in Rate4Site[5].

Figure 3S: Multiple sequence alignment (MSA) illustrations of the evolutionary conservation profile of VP3 calculated by ConSurf-DB[1]. Amino acid sequences similar to VP3 sequence in the 2WZR PDB (2WZR3) are retrieved and aligned using CSI-BLAST[2,3] and MAFFT[4], respectively. The MSA is coloured by evolutionarily conservation scores calculated for each residue using an empirical Bayesian inference in Rate4Site[5].

Figure 1S

```
2WZR1                        TTSAGEGA--DPV-TTDASAHGGDT-RTTRRAHTDVTFLLDRFTLVGKTN
UniRef90_Q6PMU0_725_943      TTSAGEGA--EPV-TTDASQHGGDR-RTARRYHTDVSFLLDRFTLVGRTQ
UniRef90_F1AW55_725_943      TTSAGEGA--EPV-TTDATQHGGHS-RRTRRHHTDVSFLLDRFTLIGKTT
UniRef90_A0PG93_1_219        TTSAGEGA--EPV-TTDASQHGGTS-RRVRRQHTDVSFLLDRFTLIGKTH
UniRef90_A0PG86_1_219        TTSAGEGA--DVV-TVDAAAHGGNQ-RRTRRVHTDVAFLLDRFTLVGKTQ
UniRef90_A0PG77_1_219        TTSAGEGA--DPV-TTDATQHGGGR-RTARRHHTDVSFLLDRFTLVGKTQ
UniRef90_Q6PMU2_725_943      TTSAGEGA--DVV-TVDATVHGGNQ-RRTRRVHTDVAFLLDRFTLVGKTQ
UniRef90_Q8V0G8_526_744      TTSAGEGA--EPV-TTDASQHGGNA-RPTRRYHTNVEFLLDRFTLIGKTH
UniRef90_A0PG80_1_219        TTSAGEGA--EPV-TTDASQHGGGR-RAARRQHTDVSFLLDRFTLVGKTV
UniRef90_Q8BDT2_1_218        TTSAGEGA--DVV-TXDASAHGGNT-RPTRRVHTDVAFLLDRFTLVGKTV
UniRef90_A0PG94_1_219        TTSAGEGA--DVV-TVDATAHGGTQ-RATRRVHTDVAFLLDRFTLVGKTV
UniRef90_C8YS70_1_219        TTSAGEGA--EVV-STDATAHGGNR-RRARRVHTDVAFLLDRFTLIGKTV
UniRef90_Q6PMT8_726_934      TTSAGEGA--EVV-TTDPSTHGGQV-KEKRRMHTDVAFVLDRFTHV-HTN
UniRef90_Q6PMT3_724_939      TTSAGEGA--DVV-TTDVTTHGGEV-SVPRRQHTNVEFLLDRFTHVGKVN
UniRef90_B8Y0J7_726_934      TTSAGEGA--DVV-TTDPSTHGGAV-REGSRKHTEVAFLLDRSTHV-HTN
UniRef90_E2IU95_1_209        TTSAGEGA--DVV-TTDPSTHGGSV-VEKRRMHTDVAFVLDRFTHV-HTN
UniRef90_Q80JY0_1_209        TTSSGEGA--DVI-TTDPSTHGGSV-VEKRRMHTDVAFVLDRFTHV-HTN
UniRef90_A0PFY2_1_209        TTSAGEGA--DVV-TTDPSTHGGNV-QEGRRKHTEVAFLLDRSTHV-HTN
UniRef90_Q80K03_1_209        TTTAGEGA--DVV-TSDPSTHGGQV-SEKRRMHTDVTXVLDRFTHV-HTN
UniRef90_Q1L757_525_734      TTSAGEGA--DVV-TTDPSTHGGVVDTKKRRWHTDVSFVMDRATHV-HTN
UniRef90_Q80K16_1_209        TTSAGEGA--DVV-TTDPTTHGGSV-QTPRRVHTDVAFLLDRSTHV-FTD
UniRef90_Q1L752_527_735      TTSAGESA--DVV-TTDPTTHGGAV-TNPRRKHTDVAFLLDRSTHV-HTG
UniRef90_A0PG17_1_209        TTSAGEGA--DVV-TTDPTTHGGSV-RNPRRIHTDVTFLLDRSTHV-HTN
UniRef90_Q80JY2_1_209        TTSAGEGA--DVV-TTDPTTHGGHP-NAARRKHTDIAFLLDRSTHV-HTN
UniRef90_Q80JY8_1_209        TTSSGEGA--DVV-TTDPSTHGGAV-TEKRRMHTDVAFVMDRFTHV-LTD
UniRef90_Q8JVD0_525_739      TTSAGEGG--DVV-TTDVTTHGGAV-DTPRRQHTNVEFLLDRFTHIGTIT
UniRef90_Q80JU2_1_209        TTSAGEGA--DVV-TTDPTTHGGAV-ANPRRKHTDVSFLLDRSTHV-HTG
UniRef90_A0PFY7_1_209        TTSAGEGA--DVV-TIDPTTHGGSV-TPARRIHTDVAFLLDRSTHV-HTN
UniRef90_A0PFY4_1_209        TTSAGEGA--DVV-TTDPSTHGGNV-LEKRRMHTDVAFVLDRFTHV-HTS
UniRef90_Q80JZ5_1_205        TTSAGEGA--DVV-TTVPSTHGGRV-VEKRRMHTDVAFVLDRFTHV-HTS
UniRef90_A0PFZ0_1_209        TTSAGEGA--DVV-TTDPTTHGGTV-TAARRVHTDVAFLLDRSTHV-HTN
UniRef90_Q1L748_525_740      TTSSGEGA--DVV-STTVETHGGTE-QVARRQHTDVAFLLDRFTHIGSMA
UniRef90_Q80JY7_1_209        TTSAGEGA--DVV-TTDPSTHGGVV-VEKRRMHTDVAFVMDRFTHV-LTD
UniRef90_A0PG12_1_209        TTSAGEGA--EVV-TTDPTTHGGSV-GAPRRVHTDVAFLLDRSTHV-HTQ
UniRef90_A0PFY8_1_209        TTSASEGA--DVI-TTGPATHGGTE-GTARRIHTDVAFLLDRSTHV-HTN
UniRef90_Q80JZ1_1_209        TTSAGEGA--DVV-TTDPSTHGGSV-IEKRXMHTDVALXLDRFTHV-HTN
UniRef90_Q80JU1_1_209        TTSAGEGA--DVV-TTDPSTHGGAV-TSVKRKHTEVAFVMDRFTHV-HTN
UniRef90_B2MZR9_1_211        TTSTGESA--DPV-TTAVEYYGGET-QIQRRQHTDVSFIMDRFVNIDSL-
UniRef90_P49303_726_936      TTSTGESA--DPV-TTTVENYGGET-QVQRRQHTDVTFIMDRFVKIQNL-
UniRef90_Q2I5Z0_71_281       TTSTGESA--DPV-TATVENYGGET-QVQRRQHTDVSFILDRFAKVTP-V
UniRef90_Q9DJ49_1_212        TTSAGESA--DPV-TATVENYGGET-QVQRRQHTDVSFILDRFVKVTPTN
UniRef90_A1EBZ1_1_211        TTTAGESA--DPV-STTVENYGGET-QAQRRQHTDVSFIMDRFVRINAV-
UniRef90_A1EBZ4_1_211        TTSAGESA--DPV-TTTVENYGGET-QVQRRHHTDVGFIMDRFVKIGNT-
UniRef90_I0AZ32_1_211        TTATGESA--DPV-TTTVENYGGET-QVQRRHHTDIGFIMDRFVKITRT-
UniRef90_A8D5K4_1_211        TTSTGESA--DPV-TATVENYGGET-QVQRRQHTDVSFILDRFVKVTP-K
UniRef90_Q2I5X3_80_290       TTSAGESA--DPV-TTTVENYGGET-QVQRRHHTDVGFIMDRFVKINNT-
UniRef90_I0AYZ5_1_211        TTATGESA--DPV-TTTVENYGGET-QTQRRQHTDVGFIMDRFVKIDQQ-
UniRef90_I0AZ39_1_210        TTATGESA--DPV-TTTVENYGGET-QVQRRHHTDVSFILDRFVKISTQ-
UniRef90_H9T7U2_127_337      TTSVGESA--DPV-TATVENYGGET-QVQRRQHTDVAFILDRFVKVTP-K
UniRef90_Q9DJF9_1_211        TTSAGESA--DPV-TTTVENYGGET-QVQRRQHTDVXIILDRFVKVTP-K
UniRef90_I0AYY0_1_211        TTATGESA--DPV-TTTVENYGGET-QVQRRQHTDIGFIMDRFVKIKDP-
UniRef90_Q2I5X1_23_232       TTTAGESA--DPV-TTTVENYGGET-QVQRRHHTDVHFIMDRFVKISPT-
UniRef90_B2ZHQ6_1_211        TTSTGESA--DPV-TATVENYGGET-QVQRRHHTDVSFILDRFVKVTP-Q
UniRef90_A4ZPM0_1_211        TTATGESA--DPV-TTTVENYGGET-QVRRRQHTDVSFIMDRFVKINSP-
UniRef90_E7D638_1_210        TTTTGESA--DPV-TTTVENYGGET-QVQRRRHTDVSFIMDRFVQIKPA-
UniRef90_Q8JUM5_1_211        TTTAGESA--DPV-TTTVENYGGET-QDQRRQHTDVSFIMDRFVKVNTV-
UniRef90_Q8BED5_1_209        TTTTGESA--DPV-TTTVENYGGET-QTARRLHTDVAFVLDRFVKLTAP-
UniRef90_A0ZXF2_1_211        TTSTGESA--DPV-TSTVENYGGET-QVQRRHHTDVAFILDRFVKVTP-K
UniRef90_F1BVI2_1_211        TTTVGESA--DPV-TTTVENYGGET-QIQRRHHTDVGLIMDRFVKIGTT-
UniRef90_I0AYX9_1_210        TTAVGESA--DPV-TTTVENYGGET-QTQRRQHTDTGFIMDRFVKISPQ-
UniRef90_I0AZ13_1_211        TTATGESA--DPV-TTTVENYGGET-QVQRRHHTDIGFIMDRFVKIKDL-
UniRef90_A4ZPL8_1_211        TTATGESA--DPV-TTTVENYGGET-QVQRRHHTDVGFIMDRFVKVNSS-
UniRef90_Q6PMY9_724_932      TTTAGESA--DPV-TTTVENYGGET-QAARRLHTDVAFVLDRFVKLTQP-
UniRef90_Q8JUP6_1_211        TTTTGESA--DPV-TTTVENYGGET-QVQRRKHTDVGFVLDRFVKINSI-
UniRef90_B1A0W6_1_210        TTATRESA--DPV-TTTVENYGGET-QTRRRQHTDVDFIMDRFVKISPV-
UniRef90_C0LRW1_1_211        TTTTGESA--DPV-TTTVENYGGET-QAQRRHHTDISFIMDRFVKVNAV-
UniRef90_Q8JUN6_1_211        TTTAGESA--DPV-TTTVENYGGET-QVQRRHHTDVSFIMDRFVKISTV-
UniRef90_A1EC01_1_211        TTLTGESA--DPV-TTTVENYGGET-QVQRRHHTDVSFILDRFVKINNP-
UniRef90_A2I8Z5_725_935      TTSAGESA--DPV-TATVENYGSET-QVQRRQHTDVTFILDRFVKVKP-K
```

```
UniRef90_B1A0W7_1_211      TTTAGESA--DPV-TTTVENYGGET-QAQRRQHTDVGFIMDRFVKINPV-
UniRef90_Q9DJ38_1_212      TTSAGESA--DPV-TATVENYGGET-QVRRRQHTDVSFILDRFVKVTPKA
UniRef90_A1EC10_1_211      TTSAGESA--EPV-TTTVEDYGGET-QVQRRHHTDVSFIMDRFVKIGTT-
UniRef90_E2FZV2_1_211      TTSPGESA--DPV-TATVENYGGET-QIQRRQHTDVSFILDRFVKVTP-Q
UniRef90_I0AYY6_1_211      TTATGESA--DPV-TTTVENYGGET-QIQRRHHTDIGFIMDRFVKIAPL-
UniRef90_A7XPU3_1_209      TTTTGESA--DPV-TTTVENYGGET-QAARRLHTDVAFVLDRFVKLTQP-
UniRef90_Q999Y2_1_211      TSASGESV--DPV-TTTVENYGGET-QVQRRHHTDVGFIMDRFVKINSP-
UniRef90_B2MZR8_1_211      TTDTGESA--DPV-TTTVENYGGET-QVQRRHHTDIGFIMDRFVKIKXV-
UniRef90_A1EC03_1_211      TTSAGESA--DPV-TTTVENYGGET-QAQRRHHTDVSFIMDRFVKIDAA-
UniRef90_Q8BEA7_1_208      TTTAGESA--DPV-TTTVENYGGET-QSARRIHTDVAFVLDRFVKLT-P-
UniRef90_C0LYR0_1_209      TTTTGESA--DPV-TTTVEDYGGET-QTARRLHTDVSFVLDRFVKLTRP-
UniRef90_C0LS35_1_211      TTTTGGSA--DPV-TTTVGNYGGET-QVQRRHHTDVGFIMDRFVKIYYT-
UniRef90_I0AZ16_1_210      TTATGESA--DPV-TTAVENYGGET-QTQRRHHTDVSFIMDRFVKIQNP-
UniRef90_A4ZPP1_1_210      TTATGESA--DPV-TTTVENYGGET-QAQRRHNTDVSFIMDRFVRIKPV-
UniRef90_A0ZXC4_1_211      TTATGXXX--DPV-TTTVENYGGET-QVQRRHHTDVGFIMDRFVKINSP-
UniRef90_Q8JUS6_1_211      TTTAGESA--DPV-TTTVENYGGET-QAQRRHHTDVSFIMDRFVKVNTV-
UniRef90_A1EC12_1_211      TTSAGESA--EPV-TTTVEDYGGET-QVQRRHHTDVSFIMDRFVKNWTT-
UniRef90_F6KYM9_1_211      TTSAGESA--DPV-TSTVENYGGET-QVQRRHHTDVSFIMDRFVKVTP-T
UniRef90_B1A0X1_1_211      TTATGESA--DPV-TTTVENFGGET-QARRRQHTDVGFIMDRFVKINPV-
UniRef90_I0AZ19_1_211      TTATGESA--DPV-TTTVENYGGET-QVQKRHHTDIGFIMDRFVKVSAL-
UniRef90_Q912K6_1_211      TTATGESA--DPV-TTTVENYGGET-QVQRRHHTXVGFIMDRFVKINXV-
UniRef90_A4ZPL9_1_211      TTAAGESA--DPV-TTTVENYGGET-QVQRRHHTSVGFILDRFVKVNNP-
UniRef90_Q9YPW9_1_211      TTSAGESA--DPV-TATVENYGGET-QVQRRQHTDISFILDRFVKVTP-K
UniRef90_A1EC13_1_211      TTSGGESP--DPV-TTTVENYGGET-QAQRRHHTDVSFIMDRFVKIGTI-
UniRef90_B2MZQ3_1_211      TTSAGESA--YPV-TTTVENYGGET-QIQRRQHTDVSFIMDRFVKVTP-Q
UniRef90_E2J011_1_211      TTSPGESA--DPV-TATVENYGGET-QVQRRQHTDVSFILDRFVKVTP-Q
UniRef90_I0AZ31_1_211      TTATGESA--DPV-TTTVENYGGET-QVQRRHHTDVGFIMDRFVKIKDT-
UniRef90_D7RFC4_1_211      TTSRSSQA--DPV-SATVENYGGET-QVQRRHHTDVSFILDRFVKVTP-Q
UniRef90_E2IZY3_1_211      TTCPGESA--GPV-NATVEKTGGAT-QVQRRQHTDVSFILDRFVKVTP-Q
UniRef90_Q9DJ33_1_211      TTSPGESA--DPV-TATVENYGGET-QXXRRQHTDXXXXLDRFVKVTP-X
UniRef90_Q9DJ21_1_192      TTSAGESA--DPV-TTTVENYGAET-QVQRRQHTDVXFILDRFVKVTPXE
UniRef90_Q0PV54_36_239     TTATGESA--DPI-TTTVENYGGET-QPRRRQHTDVGFIMDRFVKINPV-
UniRef90_C8YS67_1_209      TTTTGESA--DPV-TTTVENYGGET-QVQRRHHTDVAFILDRFVRAQVA-
UniRef90_F6KYY3_1_211      TTSAGESA--DPV-TATVENYGGET-QIQRRQHTDVSFIMDRFVKVTP-K
UniRef90_B1A0W4_1_211      TTATGESA--DPV-TTTVENYGGET-QAQGGQHTDVGFIMDRFVKISPK-
UniRef90_B3FHW7_1_211      TTTAGESA--DPV-TTTVENYGGET-QVQRRHHTDVGFIMDRFVKIGNT-
UniRef90_A0ZXB5_1_211      TTATGXSA--DPV-TTTVENYGGET-QVQRRHHTDVGFIMDRFVKVTTT-
UniRef90_A1EBZ3_1_211      TTSAGEIL--DHR-VPTVENYGGET-QAQRRQHTDVSFIMDRFVRINAV-
UniRef90_Q9DJA4_1_210      TTSLGESA--XPV-TATVENYGGAT-XVQRRQHTDVSFILDRFVKITP-Q
UniRef90_Q0QED4_407_613    TTTTGESA--DPV-ITTVENYGGET-QVQRRHHTDVAFVLDRFVEVTVS-
UniRef90_Q8JUU0_1_211      TTATGESA--DPV-TTTVENYGGET-QVQRRQHTDVGFIMDRFVKINSL-
UniRef90_Q9DJF0_19_211     ---------------------GGXT-QGXRRQHTDVXFILDRFVKVTP-K
UniRef90_I0AZ37_1_211      TTATGESA--DPV-TTTVGNYGGET-QTQRRHHTDIGFIMDRFVKINSL-
UniRef90_Q67462_1_211      TTSMGESA--DPV-TATVENYGGDT-QVQRRQHTDVSFIMDRFVKVTP-Q
UniRef90_I0AZ36_1_211      TTATGESA--DPV-TTTVENYGGET-QTQRRHHTDIGFIMDRFVKITSL-
UniRef90_Q91EM4_1_211      TPXTGESA--DPV-TATVENYGGXT-QVXRRQHTDVAXIXDRFVXVTP-X
UniRef90_Q0PV56_33_229     TTATGESA--DPV-TTTVENYGGET-QVRRRQHTDVSFIMDRFVKIKPV-
UniRef90_C0LYW2_1_209      TTTAGESA--DPV-TTTVENYGGET-QTARRLHTDVAFVLDRFVKLTQP-
UniRef90_B2MZX2_1_207      TTTAGESA--DPG-TTTVENYGGET-QVQRRHHTDVAFVLDRFVKVQVS-
UniRef90_Q01124_1_209      TTTTXXSA--DPV-TTTVENYGGET-QVQRRHHTDVAFVLDRFVKVPVSG
UniRef90_E2J004_1_192      TTSPGQSV--DPV-TATVENYGGAT-QVQRAQPTDVSFILDTFVKVTP-Q
UniRef90_Q8JUL9_1_209      TTATGESA--DPV-TTTVENYGGET-QVQRRHHTDVGFIMDRFVKINSL-
UniRef90_Q6X5M0_1_179      TTATGESA--DPV-TTTVENYGGET-QVQRRHHTDVSFIMDRFVKINSP-
UniRef90_Q2I5Y3_113_321    TTSAGESA--DPV-TTTVENYGGET-QVQRRHHTDVGFIMDRFVKINNT-
UniRef90_Q01121_1_189      TTATGESA--DPV-TTTVENYGGET-QVQRRHHTDVAXVLDRFVKVKAS-
UniRef90_Q6X5M1_1_179      TTATGESA--DPV-TTTVENYGGET-QVQRRHHTDIAFIMDRFVKIESL-
UniRef90_A3RGT7_2_181      ----------------------------------DVSFIMDRFVKINPV-
UniRef90_P03310_11_218     TTTTGESA--DPV-TTTVENYGGET-QIQRRHHTDVAFVLDRFVKVHVS-
UniRef90_B3FHW2_1_211      TTSTFESA--DPL-TTTVDNYGGKT-QTRRRQHTDVSFMLDRFVKLTP-N
UniRef90_Q8JUM1_1_211      TTTAGESA--DPV-TTTVENYGGET-QAQRRHHTDVSFIMDRFVKVNTV-
UniRef90_Q65051_1_200      ------------V-TTTXENYGGET-QTQRRHHTDVGFTMDRFVKINSL-
UniRef90_Q8JUL2_2_187      -----------------------------RRQHTDVTFIMDRFVKVNHV-
UniRef90_B2BF63_11_200     ----RRSR--DAS-TRMSRSY------------------WDRFVKVTP-K
UniRef90_Q0PV58_32_197     TTATGESA--DPV-TTTVENYGGET-QVRRRQHTDVSFIMDRFVKINPV-
UniRef90_Q65102_9_211      ----------DPV-TATVENYGGDT-QVQRRQHTDVSFIMDRFVKVTP-Q
UniRef90_Q8BED3_1_210      TTTTGESA--DPV-TTTVENYGGET-QTARRLHTDVAFVLDRFVKLTAP-
UniRef90_A0PG53_1_160      --------------------------------------------------
UniRef90_B3FHW0_1_211      TTSTGESA--DPV-ATTVKNYGGKT-QVHRRQHVYVPFILDRFVKVTP-N
UniRef90_B3FHV9_1_211      TTSTGESA--DPV-ATTVKNYGGKT-QIRRGQHAYASFIRDRFVKLTP-R
UniRef90_B3FHV8_1_211      TTSTGESA--EPA-ATTVENYGGKT-NIRRRQHTYAFFIMDRLVKLTP-H
UniRef90_A0PG60_5_160      --------------------------------------------------
UniRef90_Q91NQ7_2_163      --------------------------------------------------
UniRef90_B3FHV6_1_211      TTSTGESA--DPL-ATTVDNYGGKT-QNRRRQHTYASFIMDRFVTLTP-N
```

```
UniRef90_B3FHV7_1_211     TTSTGESA--DPV-ATTVENYGGKA-HIRRGQHAYAFFIRDRFVKLTP-R
UniRef90_A0PG54_1_160     --------------------------------------------------
UniRef90_B3FHW1_1_211     TTSTGESA--DPV-ATTVKNYGGKT-QIRRRQHAYASFMRDRFVKLTP-N
UniRef90_Q65068_1_206     TTATGXSA--DPV-XTTVENYGXET-QVQRRHHTDVAXVLDRFVKVTVS-
UniRef90_Q9YPF0_1_208     TTTTGXSA--DPX-XTTVENYGGET-QVQRRHXXDVAFVLDRFVKVHVSD
UniRef90_Q8BEF3_1_209     TTTTGESA--DPVTTTTVENYGGET-QXXXXXXXXVAFVLDRFVKLTTP-
UniRef90_B3FHV4_1_211     TTSTGESA--EPA-ATTVENYGGKA-HIGMGQHADVSFIMDRFVKLTP-R
UniRef90_Q8JUN9_1_211     TTTAGESA--DPV-TTTVENYGGET-QAQRRHHTDVSFIMDRFVKVNTV-
UniRef90_Q9YPG1_1_165     -TAXGESA--DPV-XTTVENYGGDT-QTQRRHHTDVXFIMDRFVKXNSL-
UniRef90_A0PG23_5_162     --------------------------------------------------
UniRef90_Q9DJ61_1_156     --------------------------------------------------
UniRef90_Q65097_1_206     TTTTGXSA--XPV-TTTVENYGGET-QVQRRHHTDVAXVLDRFVKVTVS-
UniRef90_Q01126_1_208     TTTTGESA--DPX-TTTVEXYGGET-QVQXRYHTXXXXXXXXXXKIPVSD
UniRef90_Q67461_13_173    --------------------------------------------------
UniRef90_Q00280_54_209    --------------------------------------------------
UniRef90_H9AY35_719_926   TTDVGESGVGH---DATPEQLGGTT-GPAFRSHTEVSWVLDRYALAAKVN


2WZR1                     D--------------NKLVLDLLSTKEKSLVGALLRAA-TYYFSDLEVAC
UniRef90_Q6PMU0_725_943   N--------------CRLTLDLLQTKEKALVGAILRAA-TYYFSDLEVAC
UniRef90_F1AW55_725_943   D--------------NKIVLDLLQTREKALVGAILRSA-TYYFSDLEVAC
UniRef90_A0PG93_1_219     D--------------NKLVLDLLQTKEKALVGAILRSA-TYYFSDLEVAC
UniRef90_A0PG86_1_219     D--------------NKMVLDMLKTKEKALVGAILRAA-TYYFSDLEVAC
UniRef90_A0PG77_1_219     N--------------NRLTLDLLQTKEKALVGAILRAA-TYYFSDLEVAC
UniRef90_Q6PMU2_725_943   D--------------NRMVLDMLKTKEKALVGAILRSA-TYYFADLEVAC
UniRef90_Q8V0G8_526_744   N--------------NKMVLDMLRTEKKALVGAILRSA-TYYFSDLEVAC
UniRef90_A0PG80_1_219     N--------------NKLTLDLLKTKEKALVGAILRSA-TYYFADLEVAC
UniRef90_Q8BDT2_1_218     D--------------NKMVLDLLKTKEKALVGAVLRSA-TYYFSDLEVAC
UniRef90_A0PG94_1_219     D--------------NKMVLDMLQTKKKALVGAILRSA-TYYFADLEVAC
UniRef90_C8YS70_1_219     N--------------NKMVLDLLKTKEKALVGAILRSA-TYYFSDLEVAC
UniRef90_Q6PMT8_726_934   K--------------TTFNVDLMDTKEKTLVGALLRAS-TYYFCDLEIAC
UniRef90_Q6PMT3_724_939   E--------------SR-TISLMDTKEHTLVGAILRSA-TYYFCDLEVAI
UniRef90_B8Y0J7_726_934   K--------------TSFAVDLMDTKEGALVGAILRAS-TYYFCDLEIAC
UniRef90_E2IU95_1_209     K--------------TTFNVDLMNTKQQTLVGALLRAS-TYYFCDLEIAC
UniRef90_Q80JY0_1_209     K--------------TSFVVDLMDTNERTLVGALLRAS-TYYFCDLEIAC
UniRef90_A0PFY2_1_209     K--------------TSFVVDLMDTKKKALVGAILRAS-TYYFCDLEIAC
UniRef90_Q80K03_1_209     K--------------TSFNVDLMDTKDKALVGALLRAS-TYYFCDLEIAC
UniRef90_Q1L757_525_734   K--------------TTFAVDLLDTKDKTLVGALLRAT-TYYFADLEIAC
UniRef90_Q80K16_1_209     K--------------TSFAVDLMDTKEKALVGAILRSA-TYYFCDLEIAC
UniRef90_Q1L752_527_735   K--------------TTFEVNLMDTKEKALVGAVLRAA-TYYFCDLEIAC
UniRef90_A0PG17_1_209     K--------------TSFAVDLMDTKEKALVGAILRSA-THYFCDLEIAC
UniRef90_Q80JY2_1_209     K--------------TSFAVDLMDTKEKALVGAVLRSA-TYYFCDLEVAC
UniRef90_Q80JY8_1_209     K--------------TAFAVDLMDTNEKTLIGALLRAA-TYYFCDLEIAC
UniRef90_Q8JVD0_525_739   G--------------SK-TIDLMDTKEHTLVGAILRSV-TYYFCDLEVAV
UniRef90_Q80JU2_1_209     K--------------TTFEVNLMDTKEKALVGAVLRAA-TYYFCDLEITC
UniRef90_A0PFY7_1_209     K--------------TTFNIDLMDTKEKALVGAILRSA-TYYFCDLEIAC
UniRef90_A0PFY4_1_209     K--------------TTFNVDLMDTKQHALVGALLRAS-TYYFCDLEITC
UniRef90_Q80JZ5_1_205     K--------------TTFNVDLMDTKEKTLVGALLRAS-TYYFCDLEIAC
UniRef90_A0PFZ0_1_209     K--------------TTFAVDLMDTKEKALVGAILRSA-TYYFCDLEIAC
UniRef90_Q1L748_525_740   G--------------TK-TLDLLTTKEHTLVGAILRSA-TYYFCDLEVAV
UniRef90_Q80JY7_1_209     K--------------SAFAVDLMDTNEKTLVGALLRAS-TYYFCDLEIAC
UniRef90_A0PG12_1_209     K--------------TSFAVDLLDTKEKALVGAILRSA-TYYFCDMDITC
UniRef90_A0PFY8_1_209     K--------------TAFAVDLMDTKRKALVGAILRSA-TYYFCDLEVAC
UniRef90_Q80JZ1_1_209     K--------------TTFNVDLMDTKDKTLVGALLRAS-TYYFCDLEIAC
UniRef90_Q80JU1_1_209     K--------------TTFEINLMDTNDKTLIGALLRSA-TYYFCDLEIAC
UniRef90_B2MZR9_1_211     S--------------PTHVIDLMQTHQHGLVGALLRAA-TYYFSDLEIVV
UniRef90_P49303_726_936   N--------------PIHVIDLMQTHQHGLVGALLRAA-TYYFSDLEIVV
UniRef90_Q2I5Z0_71_281    D--------------QINVLDLMQTPAHTLVGALLRAA-TYYFADLEVAV
UniRef90_Q9DJ49_1_212     N--------------QINVLDLMQIPAHTLVGALLRAA-TYYFSDLELAV
UniRef90_A1EBZ1_1_211     S--------------PTHVIDLMQTHQRGLVGALLRAA-TYYFSDLEIVA
UniRef90_A1EBZ4_1_211     S--------------PTHVIDLMQTHQHGLVGALLRAA-TYYFSDLEIVV
UniRef90_I0AZ32_1_211     S--------------PTHVIDLMQTHQHGLVGALLRAA-TYYFSDLEIVV
UniRef90_A8D5K4_1_211     D--------------QINVLDLMQTPAHTLVGALLRTA-TYYFADLEVAV
UniRef90_Q2I5X3_80_290    S--------------PTHVIDLMQTHQHGLVGALLRAA-TYYFSDLEIVV
UniRef90_I0AYZ5_1_211     S--------------PTHVIDLMQTHQNGLVGAMLRAA-TYYFSDLEIVV
UniRef90_I0AZ39_1_210     T--------------PTHVIDLMQTHQHSLVGALLRAA-TYYFSDLEIVV
UniRef90_H9T7U2_127_337   D--------------QINVLDLMQTPAHTLVGALLRTA-TYYFADIEVAV
UniRef90_Q9DJF9_1_211     D--------------QINVLDLMQTPSYTLVGALLRTA-TYYFADLEVAV
UniRef90_I0AYY0_1_211     T--------------PTHVIDLMQTHQHGLVGAMLRAA-TYYFSDLEIVV
UniRef90_Q2I5X1_23_232    T--------------PTHVIDLMQTHQHSLVGALLRAA-TYYFSDLEVVV
UniRef90_B2ZHQ6_1_211     P--------------QINVLDLMQTPSHTLVGALLRTA-TYYFADLEVAV
```

```
UniRef90_A4ZPM0_1_211      G--------------PTHVIDLMQTHQHGLVGALLRAA-TYYFSDLEIVV
UniRef90_E7D638_1_210      S--------------PIHVIDLMQTHQHGLVGAMLRAA-TYYFSDLEIVV
UniRef90_Q8JUM5_1_211      S--------------PTHVIDLMQTHQHALVGALLRAA-TYYFSDLEIVV
UniRef90_Q8BED5_1_209      K--------------NTQTLDLMQIPSHTLVGALLRSA-TYYFSDLEVAL
UniRef90_A0ZXF2_1_211      D--------------QINILDLMQIPSHTLVGALLRAS-TYYFSDMEIVV
UniRef90_F1BVI2_1_211      N--------------PTHVIDLMQTHQHGLVGALLRAA-TYYFSDLEIMV
UniRef90_I0AYX9_1_210      T--------------PTHVIDLMQTHQHGLVGALLRAA-TYYFSDLEIVV
UniRef90_I0AZ13_1_211      S--------------PTHVIDLMQTHQHGLVGALLRAA-TYYFSDLEIVV
UniRef90_A4ZPL8_1_211      T--------------PTHVIDLMQTHQHGLVGALLRSA-TYYFSDLEVVV
UniRef90_Q6PMY9_724_932    K--------------NTQTLDLMQIPSHTLVGALLRSA-TYYFSDLEVAL
UniRef90_Q8JUP6_1_211      S--------------PTHVIDLMQTHQHGLVGALFRAA-TYYFSDLEIVV
UniRef90_B1A0W6_1_210      S--------------PTHVIDLMQTHQHALVGALLRAA-TYYFSDLEVVV
UniRef90_C0LRW1_1_211      S--------------PIHVIDLMQTPQNTLVGALLRAA-TYYFSDLEIVV
UniRef90_Q8JUN6_1_211      S--------------PTHVIDLMQTHQHGLVGALLRAA-TYYFSDLEIVV
UniRef90_A1EC01_1_211      S--------------PTHVIDLMQTHQHGLVGAMLRAA-TYYFSDLEIVV
UniRef90_A2I8Z5_725_935    E--------------QVNVLDLMQIPAHTLVGALLRTA-TYYFSDLELAV
UniRef90_B1A0W7_1_211      S--------------PTHVIDLMQTHPHALVGALLRAA-TYYFSDLEIVV
UniRef90_Q9DJ38_1_212      D--------------QINVLDLMQIPAHTLVGGLVRAA-TYYFSDLELAV
UniRef90_A1EC10_1_211      N--------------PTHVIDLMQTHHHGLVGALLRAA-TYYFSDLEIVV
UniRef90_E2FZV2_1_211      E--------------QINVLDLMQTPAHTLVGALLRTA-TYYFADLEVAV
UniRef90_I0AYY6_1_211      T--------------PTHVIDLMQTHQHGLVGALLRAA-TYYFSDLEIVV
UniRef90_A7XPU3_1_209      K--------------STQVLDLMQIPSHTLVGALLRSA-TYYFSDLEIAL
UniRef90_Q999Y2_1_211      S--------------PTHVIDLMQTHQHGLVGGLLRAA-TYYFSDLEIVV
UniRef90_B2MZR8_1_211      Q--------------PTHVIDLMQTHQHGLVGAMLRAA-TYYFSDLEIVV
UniRef90_A1EC03_1_211      S--------------PIHVIDLMQTPHNTLVGALFRAA-TYYFSDLEIVV
UniRef90_Q8BEA7_1_208      K--------------NTQILDLMQIPSHTLVGALLRSA-TYYFSDLEVAL
UniRef90_C0LYR0_1_209      K--------------DTQTLDLMQIPSHTLVGALLRSA-TYYFSDLEVAL
UniRef90_C0LS35_1_211      R--------------PTHVIDLMQTHQHGLVGALLRAA-TYYFSDLEIVV
UniRef90_I0AZ16_1_210      S--------------PIHAIDLMQTHQHGLVGALLRAA-TYYFSDLEIVV
UniRef90_A4ZPP1_1_210      S--------------PTHVIDLMQTHQHGLVGALLRAA-TYYFSDLEIVV
UniRef90_A0ZXC4_1_211      K--------------STHVIDLMQTHQHGLVGALLRAA-TYYFSDLEIVA
UniRef90_Q8JUS6_1_211      S--------------STHVIDLTQTPQHTLVGALLRAA-TYYFSDLEIVV
UniRef90_A1EC12_1_211      T--------------HAHFMDLMQTHQHGLVGALLRAA-TYYFSDLEIVV
UniRef90_F6KYM9_1_211      S--------------GINVLDLMQIXAHTLVGALLXAX-TYYFSDMEIAV
UniRef90_B1A0X1_1_211      T--------------PTHVIDLMQTHQHALVGALLRAA-TYYFSDLEIVV
UniRef90_I0AZ19_1_211      S--------------PIHVIDLMQTHQHGLVGALLRAA-TYYFSDLEIVV
UniRef90_Q912K6_1_211      K--------------PTHVIDLMQTHQHGLVGALLRAA-TYYFSDLEIVV
UniRef90_A4ZPL9_1_211      N--------------PTHVIDLMQTHQHGLVGAMLRAA-TYYFSDLEIVV
UniRef90_Q9YPW9_1_211      D--------------QINVLDLMQIPAHTLVGALLRTA-AYYFSDSELAV
UniRef90_A1EC13_1_211      T--------------PTHVIDLMQTHQHGLVGALLRAA-TYYFSDLEIVV
UniRef90_B2MZQ3_1_211      N--------------QINTLDLMQIPSHTLVGALXRAS-TYYFSDLEIAV
UniRef90_E2J011_1_211      D--------------QINVLDLMQTPAHTLVGALLRTA-TYYFADIEVAV
UniRef90_I0AZ31_1_211      D--------------STHVIDLMQTHQHGLVGALLRAA-TYYFSDLEIVV
UniRef90_D7RFC4_1_211      D--------------QINVLNQMQAAPNTLVGSLLRTA-TYYFADLEVAV
UniRef90_E2IZY3_1_211      D--------------QVNVLDLMQIPAHTLMGALLHAS-TYYFGDLEVAV
UniRef90_Q9DJ33_1_211      D--------------QINVLDLMQTPAHTLVGALLRTA-TYYFADIXVAV
UniRef90_Q9DJ21_1_192      N--------------QINVLDLMQTPAHTLVGALXRVA-TYYFADLEVAV
UniRef90_Q0PV54_36_239     T--------------PTHVIDLMQTHQHALVGALLRAA-TYYFSDLEVVV
UniRef90_C8YS67_1_209      G--------------NQHTLDMMQTHKDGIVGALLRAA-TYYFSDLEIAV
UniRef90_F6KYY3_1_211      N--------------QVNVLDLMQIXSHTLVGALLRAF-TYYFSDLXIXX
UniRef90_B1A0W4_1_211      T--------------PTHVIDLMQTHQYALVGALLRAA-TYYFSDLEIVV
UniRef90_B3FHW7_1_211      S--------------PTHVIDLMQTHQHGLVGALLRAA-TYYFSDLEIVV
UniRef90_A0ZXB5_1_211      A--------------PTHVIDLMQTHQHGLVGALLRAA-TYYFSDLEIVV
UniRef90_A1EBZ3_1_211      S--------------PTHVIDLMQTHQRGLVGALLRAA-TYYFSDLEIVA
UniRef90_Q9DJA4_1_210      D--------------QINVXDLMQIPAHTLVGALLRAS-TYYFADLEVAV
UniRef90_Q0QED4_407_613    G--------------NQHTLDVMQAHKDNIVGALLRAA-TYYFSDLEIAV
UniRef90_Q8JUU0_1_211      S--------------PTHVIDLMHTHKHGIVGALPRAA-TYYFSDLEIVV
UniRef90_Q9DJF0_19_211     D--------------QINVLDLMQTPAHTLVGALLRTA-TYYFADLEVAV
UniRef90_I0AZ37_1_211      S--------------PTHVIDLMQTHQNGLVGALLRAA-TYYFSDLEIVV
UniRef90_Q67462_1_211      S--------------SINTLDLMQIPSHTLVGALLRAS-TYYFSDLEIVV
UniRef90_I0AZ36_1_211      S--------------PIHVIDLMQTHQNGLVGALLRAA-TYYFSDLEIVV
UniRef90_Q91EM4_1_211      A--------------QINVXDLMXTPXHTLVGALLRTA-TYYFADLEVAV
UniRef90_Q0PV56_33_229     T--------------PTHVIDLMQTHQHALVGALLRAA-TYYFSDLEIVV
UniRef90_C0LYW2_1_209      K--------------NTQTLDLMQIPSHTLVGALLRSA-TYYFSDLEVAL
UniRef90_B2MZX2_1_207      D--------------NQHTLDMMQVHKDSIVGALLRAA-TYYFSDLEIAV
UniRef90_Q01124_1_209      G--------------QQHTLDVMQVHKDSIVGALLRAA-TYXFSXLEIAV
UniRef90_E2J004_1_192      D--------------QINVLDLMQIPAHTLVGALLRAS-TYNFADLEVAV
UniRef90_Q8JUL9_1_209      S--------------PTHVIDLMHTQKHGTVGAXXXAA-TYYFSDLEIVV
UniRef90_Q6X5M0_1_179      S--------------STHVIDLMQTHKHGLVGALLRAA-TYYFSDLEIVV
UniRef90_Q2I5Y3_113_321    S--------------PTHVIDLMQTHQHGLVGAL-------------CVL
UniRef90_Q01121_1_189      G--------------NQHTLDVMQVHKDSIXXALLRAA-TYYFSDLEIAV
UniRef90_Q6X5M1_1_179      S--------------PTHVIDLMQTHQHGLVGALLRAA-TYYFSDLEIVV
UniRef90_A3RGT7_2_181      T--------------PTHVIDLMQTHQHALVGALLRAA-TYYFSDLEIVV
```

```
UniRef90_P03310_11_218   G--------------NQHTLDVMQVHKDSIVGALLRAA-TYYFSDLEIAV
UniRef90_B3FHW2_1_211    Y--------------LIPVLNLMQTPPHTLVGTLVRSA-PYYFADLYSTV
UniRef90_Q8JUM1_1_211    G--------------STHVIDLTXXXXXXXXXXXXRAA-TYYFSDLEIVV
UniRef90_Q65051_1_200    S--------------PTHVIDLMQXHQHGLXXALLRAA-TYYFSDSEXVV
UniRef90_Q8JUL2_2_187    S--------------PTHVIDLMQTHQHALVGALLRAA-TYYFSDLEIVV
UniRef90_B2BF63_11_200   D--------------QINVLDLMQTPAHTLVGALLRTA-TYYFADLEVAV
UniRef90_Q0PV58_32_197   A--------------PTHVIDLMQTHQHALVGALLRAA-TYYFSDLEIVV
UniRef90_Q65102_9_211    S--------------SINTLDLMQIQSHTLVXXXXXAS-TYYFSDLEIVV
UniRef90_Q8BED3_1_210    K--------------NTQTLDLMQIPSHTLVGALFRSAXXXXXXXXXXXX
UniRef90_A0PG53_1_160    -----------------YVLDLMQDPAHTLVGALLRTA-TYYFADLEVAV
UniRef90_B3FHW0_1_211    Y--------------LITVLNLMRTPSHTLGGTLLRSA-IYYFSDWKGTG
UniRef90_B3FHV9_1_211    L--------------QITILNLVRIPSHTLGGTLVRTA-TYYFSDLYITG
UniRef90_B3FHV8_1_211    L--------------HIPVLNLVGSPSDTLGGTLFRTA-TYYFSDMESKA
UniRef90_A0PG60_5_160    ---------------------LMQTPAHTLVGALLRTA-TYYFADLEVAV
UniRef90_Q91NQ7_2_163    ------------------VLDLMQAPAHTLVGALLRTA-TYYFADLEVAV
UniRef90_B3FHV6_1_211    Y--------------LIPILNQMRAPPHTLVGTLVRTA-PYYFSDLYSTV
UniRef90_B3FHV7_1_211    L--------------QIPFLNLVRIPPHTLVGTLVRTS-TYYFSDLYRTC
UniRef90_A0PG54_1_160    -----------------LCLGLDATPAHTLVGALLRTA-TYYFADLEVAV
UniRef90_B3FHW1_1_211    Y--------------LINVLNLVRTPSHTLGGTLFRTA-TYYFADLDMTG
UniRef90_Q65068_1_206    D--------------NQHXLDVMQAXRDXXVXALLRAA-TYYFSDLEIAV
UniRef90_Q9YPF0_1_208    X--------------XXXXLDVMQVHKDSIVGALLRAA-TYYFSDLEXAV
UniRef90_Q8BEF3_1_209    K--------------NTQTLDLMQIPSHTLVGALLRSA-TYYFSDL-XXX
UniRef90_B3FHV4_1_211    L--------------QIPILNLVRVPSHTLGGTLVRTS-TYYFSDLYRAV
UniRef90_Q8JUN9_1_211    G--------------STHVIDLTQTPQHTLVGALLRAA-TYYFSDLEIVV
UniRef90_Q9YPG1_1_165    S--------------PTHVIDLMXTXQHGLVXALLRAA-TYYFSDLEIVV
UniRef90_A0PG23_5_162    ---------------------LFQTPAHSLVVRLLRTA-TYYFADLEVAV
UniRef90_Q9DJ61_1_156    ------------------------TPAHTLVGALLRTA-TYYFADLEVAV
UniRef90_Q65097_1_206    D--------------NQHTLDVMQAHKDNIVGALLRAA-TYXXSXLEIAV
UniRef90_Q01126_1_208    G--------------QQHTLDLMQVHKDSIVGALLRAA-TYYFSXLEIAV
UniRef90_Q67461_13_173   -------------------LDLMQIPSHTLVGALLRAS-TYYFSDLEIVV
UniRef90_Q00280_54_209   ---------------------VMQVHKDSIVGALLRAA-TYYFSALEIAV
UniRef90_H9AY35_719_926  G--------YKEALGAGLVLDPVALPETSMMGQLLRAA-TYYFADMELAV
```

```
2WZR1                    ----------VGTNA-----WVGW--------TPNGSPVLT----EVGD-
UniRef90_Q6PMU0_725_943  ----------VGTNE-----WVGW--------TPNGAPELS----EVGD-
UniRef90_F1AW55_725_943  ----------VGNNK-----WVGW--------TPNGAPLLH----EVGD-
UniRef90_A0PG93_1_219    ----------VGTNK-----WVGW--------TPNGAPQLT----EVGD-
UniRef90_A0PG86_1_219    ----------VGTNK-----WVGW--------TPNGAPELN----EVGD-
UniRef90_A0PG77_1_219    ----------VGENK-----WVGW--------TPNGAPELS----EVGD-
UniRef90_Q6PMU2_725_943  ----------VGTNK-----WVGW--------LPNGAPVPK----EVGD-
UniRef90_Q8V0G8_526_744  ----------VGTNE-----WVGW--------VPNGTPDVS----EVGD-
UniRef90_A0PG80_1_219    ----------VGTNR-----WVGW--------LPNGAPVPQ----EVGD-
UniRef90_Q8BDT2_1_218    ----------VGTNK-----WVGW--------VPNGAPVPK----EVGD-
UniRef90_A0PG94_1_219    ----------VGTNK-----WVGW--------LPNGAPVPS----EVGD-
UniRef90_C8YS70_1_219    ----------LGTNK-----WVGW--------LPNGAPMPT----EVGD-
UniRef90_Q6PMT8_726_934  ----------VGTHK-----RVYW--------QPNGAPRTT----QLGD-
UniRef90_Q6PMT3_724_939  ----------LGTAP-----WAAW--------VPNGCPHTG----RVED-
UniRef90_B8Y0J7_726_934  ----------VGDHK-----RVFW--------QPNGAPRTT----QLGD-
UniRef90_E2IU95_1_209    ----------VGTHQ-----RVYW--------QPNGAPRTT----KLGD-
UniRef90_Q80JY0_1_209    ----------LGKHA-----RVWW--------QPNGAPRTT----QLRD-
UniRef90_A0PFY2_1_209    ----------VGDHT-----RVFW--------QPNGAPRTT----QLGD-
UniRef90_Q80K03_1_209    ----------VGKHT-----RVFW--------QPNGAPRTS----ELGD-
UniRef90_Q1L757_525_734  ----------VGDHK-----RVYW--------QPNGAPRTT----QLGD-
UniRef90_Q80K16_1_209    ----------VGDHT-----RVFW--------QPNGAPRTT----KLGD-
UniRef90_Q1L752_527_735  ----------VGDHK-----RVFW--------QPNGAPRAT----QLGD-
UniRef90_A0PG17_1_209    ----------VGDHK-----RVFW--------QPNGAPRTV----QLGD-
UniRef90_Q80JY2_1_209    ----------VGDHA-----RVFW--------QPNGAPRTT----QLGD-
UniRef90_Q80JY8_1_209    ----------LGEHT-----RVWW--------QPNGSPRTT----XLRD-
UniRef90_Q8JVD0_525_739  ----------LGTG------WVGW--------VPNGCPHTT----RVED-
UniRef90_Q80JU2_1_209    ----------VGEHA-----RVFW--------QPNGAPRTT----QLGD-
UniRef90_A0PFY7_1_209    ----------VGEHK-----RVFW--------QPNGAPRTT----TLGD-
UniRef90_A0PFY4_1_209    ----------VGEHS-----RVYW--------QPNGAPRTT----QLGD-
UniRef90_Q80JZ5_1_205    ----------VGEHA-----RVYW--------QPNGAPRTT----QLGD-
UniRef90_A0PFZ0_1_209    ----------VGEHK-----RVFW--------QPNGAPRTT----QLGD-
UniRef90_Q1L748_525_740  ----------KGDSP-----YAAW--------VPNGCPEVS----RVED-
UniRef90_Q80JY7_1_209    ----------IGEHA-----RVWW--------QPNGAPRTT----QLRD-
UniRef90_A0PG12_1_209    ----------VGEHA-----RVFW--------QPNGAPRTN----ELGE-
UniRef90_A0PFY8_1_209    ----------VGEHK-----RVFW--------QPNGAPRTT----VLGD-
UniRef90_Q80JZ1_1_209    ----------VGDHT-----RVFW--------QPNGAPRTT----VLGD-
UniRef90_Q80JU1_1_209    ----------VGDHQ-----RVFW--------QPNGAPRTT----QLRD-
```

```
UniRef90_B2MZR9_1_211      ----------RHDGN------LTW--------VPNGTPEAA---LSNAS-
UniRef90_P49303_726_936    ----------RHDGN------LTW--------VPNGAPEAA---LSNMG-
UniRef90_Q2I5Z0_71_281     ----------KHEGN------LTW--------VPNGAPETA---LDNTT-
UniRef90_Q9DJ49_1_212      ----------VHEGN------LTW--------VPNGAPEAA---LNNST-
UniRef90_A1EBZ1_1_211      ----------RHEGN------LTW--------VPNGAPEAA---LSNAS-
UniRef90_A1EBZ4_1_211      ----------RHDGN------LTW--------VPNGAPEAA---LSNTG-
UniRef90_I0AZ32_1_211      ----------RHDGS------LTW--------VPNGAPETA---LSNAS-
UniRef90_A8D5K4_1_211      ----------KHEGN------LTW--------VPNGAPEAA---LDNTT-
UniRef90_Q2I5X3_80_290     ----------RHEGN------LTW--------VPNGAPEAA---LSNAG-
UniRef90_I0AYZ5_1_211      ----------RHDGN------LTW--------VPNGAPESA---LSNAS-
UniRef90_I0AZ39_1_210      ----------RHDGN------LTW--------VPNGAPEAA---LLNTS-
UniRef90_H9T7U2_127_337    ----------KHEGN------LTW--------VPNGAPETA---LDNAT-
UniRef90_Q9DJF9_1_211      ----------KHEGN------LTW--------XPNGAPETA---LDNTT-
UniRef90_I0AYY0_1_211      ----------RHDGN------LTW--------VPNGAPETA---LDNTS-
UniRef90_Q2I5X1_23_232     ----------RHDGN------LTW--------VPNGAPEAA---LSNTS-
UniRef90_B2ZHQ6_1_211      ----------KHEGD------LTW--------VPNGAPEAA---LENTT-
UniRef90_A4ZPM0_1_211      ----------KHEGN------LTW--------VPNGTPESA---LQNAS-
UniRef90_E7D638_1_210      ----------NHTGN------LTW--------VPNGAPEAA---LNNTS-
UniRef90_Q8JUM5_1_211      ----------RHDGN------LTW--------VPNGAPEAA---LSNTS-
UniRef90_Q8BED5_1_209      ----------VHTGP------ATW--------VPNGSPKHA---LDNQT-
UniRef90_A0ZXF2_1_211      ----------KHEGN------LTW--------XPNGAPEVA---LDNTT-
UniRef90_F1BVI2_1_211      ----------RHSGN------LTW--------VPNGAPEAA---LSNTG-
UniRef90_I0AYX9_1_210      ----------RHEGN------LTW--------VPNGAPEGA---LANTS-
UniRef90_I0AZ13_1_211      ----------RHDGN------LTW--------VPNGAPVSA---LLNTS-
UniRef90_A4ZPL8_1_211      ----------KHQGN------LTW--------VPNGAPEAA---LANTS-
UniRef90_Q6PMY9_724_932    ----------VHTGS------VTW--------VPNGAPKDA---LDNHT-
UniRef90_Q8JUP6_1_211      ----------RHDGN------LTW--------VPNGAPEAA---LRNTG-
UniRef90_B1A0W6_1_210      ----------RHEGN------LTW--------VPNGAPEAA---LSNTS-
UniRef90_C0LRW1_1_211      ----------RHTDN------LTW--------VPNGAPEAA---LSNTS-
UniRef90_Q8JUN6_1_211      ----------HHEGN------LTW--------VPNGAPEAA---LGNVS-
UniRef90_A1EC01_1_211      ----------RHDDN------LTW--------VPNGAPVDA---LSNTS-
UniRef90_A2I8Z5_725_935    ----------KHEGD------LTW--------VPNGAPETA---LDNTT-
UniRef90_B1A0W7_1_211      ----------RHDGN------LTW--------VPNGAPVEA---LANTG-
UniRef90_Q9DJ38_1_212      ----------KHEGN------LTW--------VPNGAPEAA---LDNTT-
UniRef90_A1EC10_1_211      ----------RHAGN------LTW--------APNGAPEAA---LSNTG-
UniRef90_E2FZV2_1_211      ----------KHEGD------LTW--------VPNGAPETA---LDNTT-
UniRef90_I0AYY6_1_211      ----------RNEGN------LTW--------VPNGPPNRA---LLKAS-
UniRef90_A7XPU3_1_209      ----------VHTGP------VTW--------VPNGAPKVA---LDNQT-
UniRef90_Q999Y2_1_211      ----------QHDGN------LTW--------VPNGAPESA---LSYSS-
UniRef90_B2MZR8_1_211      ----------RHDGN------LTW--------VPNGAPESA---LDNTG-
UniRef90_A1EC03_1_211      ----------RHTDN------LTW--------VPNGAPEAA---LSNTG-
UniRef90_Q8BEA7_1_208      ----------IHTGS------VTW--------VPNGAPRTR---LDNYT-
UniRef90_C0LYR0_1_209      ----------VHTGP------VTW--------VPNGAPKTA---LDCQT-
UniRef90_C0LS35_1_211      ----------RHEGN------LTW--------VPNGAPEVA---LNNAG-
UniRef90_I0AZ16_1_210      ----------RHDGN------LTW--------VPNGAPVAA---LSNTS-
UniRef90_A4ZPP1_1_210      ----------HHTGN------LTW--------VPNGAPEAA---LDNTS-
UniRef90_A0ZXC4_1_211      ----------RHDGN------LTW--------VPNGAPVSA---LSNTS-
UniRef90_Q8JUS6_1_211      ----------RHTGN------LTW--------VPNGAPEAA---LSNTS-
UniRef90_A1EC12_1_211      ----------RHDGN------LTW--------VPNGAPEAA---LSNTG-
UniRef90_F6KYM9_1_211      ----------RHEGN------LTW--------VPNGAPEQA---LDNTT-
UniRef90_B1A0X1_1_211      ----------RHEGN------LTW--------VPNGTPEVA---LSNMS-
UniRef90_I0AZ19_1_211      ----------RHDGN------LTW--------VPNGAPESD---LSNTS-
UniRef90_Q912K6_1_211      ----------HHDGN------LTW--------VPNGAPESA---LSNIS-
UniRef90_A4ZPL9_1_211      ----------RHEGN------LTW--------VPNGAPESA---LLNTS-
UniRef90_Q9YPW9_1_211      ----------KHKGG------LTW--------VPNGAPETA---LDNTT-
UniRef90_A1EC13_1_211      ----------RHDGT------LTW--------VPNGAPEAA---LSNTG-
UniRef90_B2MZQ3_1_211      ----------KHEGD------LTW--------VPNGAPEKA---LDNTT-
UniRef90_E2J011_1_211      ----------KHERN------LTW--------VPNGAPEST---LDNTT-
UniRef90_I0AZ31_1_211      ----------RHDGN------LTW--------VLNGALESA---LLNTS-
UniRef90_D7RFC4_1_211      ----------KHEGD------LTW--------VPNGAPEAA---LGNTT-
UniRef90_E2IZY3_1_211      ----------KHDGN------LTW--------VPNGAPESA---LDNTT-
UniRef90_Q9DJ33_1_211      ----------KHEGN------LXG--------APNGAPESA---XDNTT-
UniRef90_Q9DJ21_1_192      ----------KHEGN------LTW--------VPNGAPETA---LENTT-
UniRef90_Q0PV54_36_239     ----------RHEGN------LTW--------VPNGTPEAA---LSNMS-
UniRef90_C8YS67_1_209      ----------THTGK------LTW--------VPNGAPVSA---LDNTS-
UniRef90_F6KYY3_1_211      ----------EXEXD------LTW--------VPTEAPEKA---LDNTT-
UniRef90_B1A0W4_1_211      ----------RHEGN------LTW--------VPNGAPEAA---LSNTS-
UniRef90_B3FHW7_1_211      ----------RHDGN------LTW--------VPNGAPEAA---LSNTG-
UniRef90_A0ZXB5_1_211      ----------RHEGN------LTW--------VPNGAPESA---LSNTS-
UniRef90_A1EBZ3_1_211      ----------RHEGN------LTW--------VPNGAPEAA---LSNAS-
UniRef90_Q9DJA4_1_210      ----------KHEGN------LTW--------VPNGAPETA---XDNTT-
UniRef90_Q0QED4_407_613    ----------THTGK------LTW--------VPNGAPVSA---LNNTT-
UniRef90_Q8JUU0_1_211      ----------RHDGN------LTW--------VPNGAPEAA---LSNTS-
```

```
UniRef90_Q9DJF0_19_211     ----------KHEGD------LTW--------VPNGAPETA---LDNTT-
UniRef90_I0AZ37_1_211      ----------RHDGN------LTW--------VPNGPPKSA---LQKTS-
UniRef90_Q67462_1_211      ----------KHEGD------LTW--------VPNGAPVKA---LGNTT-
UniRef90_I0AZ36_1_211      ----------RHDGN------LTW--------VPNGAPKSA---LSNTS-
UniRef90_Q91EM4_1_211      ----------KHXGN------LTX--------XPNGAPETA---LDNTT-
UniRef90_Q0PV56_33_229     ----------RHEGN------LTW--------VPNGAPEAA---LSNTS-
UniRef90_C0LYW2_1_209      ----------VHTGP------VTW--------VPNGPPKDA---LNNHT-
UniRef90_B2MZX2_1_207      ----------THTGK------LTW--------VPNGAPVSA---LENTS-
UniRef90_Q01124_1_209      ----------THTGK------LXW--------VPNGAPVSA---LDNTT-
UniRef90_E2J004_1_192      ----------KHEGN------LTW--------VSNGAPESA---LDNTT-
UniRef90_Q8JUL9_1_209      ----------RHDGN------LTW--------VPNGAPEAA---LSXXX-
UniRef90_Q6X5M0_1_179      ----------KHDGN------LTW--------VPNGAPEAA---LQNMS-
UniRef90_Q2I5Y3_113_321    ----------PRTTS------QTWRLWCTMKATDVGAQRGTRGGLSNAG-
UniRef90_Q01121_1_189      ----------THTGK------LTW--------VPNGAPVSA---LDNTS-
UniRef90_Q6X5M1_1_179      ----------RHEGN------LTW--------VPNGAPEAA---LQNTS-
UniRef90_A3RGT7_2_181      ----------RHEGN------LTW--------VPNGAPEAA---LSNTS-
UniRef90_P03310_11_218     ----------THTGK------LTW--------VPNGAPVSA---LDNTA-
UniRef90_B3FHW2_1_211      ----------KLPVY------LTW--------IPNGAPDTA---LDNTS-
UniRef90_Q8JUM1_1_211      ----------RXXXX------XXX--------XXXXXXXXX---XXXXX-
UniRef90_Q65051_1_200      ----------RHDGN------XTR--------VPNGAPEAA---LSNTS-
UniRef90_Q8JUL2_2_187      ----------RHDGN------LTW--------VPNGAPEAA---LSNTG-
UniRef90_B2BF63_11_200     ----------KHEGN------LTW--------VPNGAPETA---LDNTT-
UniRef90_Q0PV58_32_197     ----------RHEGN------LTW--------VPNGAPEAA---LSNTS-
UniRef90_Q65102_9_211      ----------KHEGD------LTW--------VRNGAXXQA---LDNTT-
UniRef90_Q8BED3_1_210      ----------XXXXX------XXX--------XXXXXPKIA---LDNQT-
UniRef90_A0PG53_1_160      ----------KHEGN------LTW--------VPNGAPESA---LDNTT-
UniRef90_B3FHW0_1_211      ----------KHEGN------LTW--------IPNGAPDKA---LDNTT-
UniRef90_B3FHV9_1_211      ----------KHQGN------LTW--------IPNGAPEKA---LDNTT-
UniRef90_B3FHV8_1_211      ----------KHEGN------LTW--------VPNGAPDKA---MHNTT-
UniRef90_A0PG60_5_160      ----------KHEGN------LTW--------XPNGAPESA---LDNTT-
UniRef90_Q91NQ7_2_163      ----------KHEGD------LTW--------VPNGAPESA---LDNTT-
UniRef90_B3FHV6_1_211      ----------KLQGN------LTW--------IPNVAPDKA---LDNTT-
UniRef90_B3FHV7_1_211      ----------KHQGN------LTW--------IPNGAPGKA---LDNTT-
UniRef90_A0PG54_1_160      ----------KHEGN------LTW--------VPNGAPESA---LDNTT-
UniRef90_B3FHW1_1_211      ----------KHEGN------LTW--------IPNGAPDKA---LDNTT-
UniRef90_Q65068_1_206      ----------THXGK------LTW--------VPXGAPVSA---LNNXT-
UniRef90_Q9YPF0_1_208      ----------THTGK------LTW--------VPXGAPVSA---LDXXT-
UniRef90_Q8BEF3_1_209      ----------XXXXX------XXX--------XXXXXXXXX---XXXXT-
UniRef90_B3FHV4_1_211      ----------KHQGN------LTW--------VPNGAPEKA---LDNTT-
UniRef90_Q8JUN9_1_211      ----------RHTGN------LTW--------VPNGAPXXX---XXXXX-
UniRef90_Q9YPG1_1_165      ----------RHXGN------LTW--------VPNGAPEAA---LSNTS-
UniRef90_A0PG23_5_162      ----------KHEGN------FTW--------VPNGAPESV---WDNTT-
UniRef90_Q9DJ61_1_156      ----------KHEGN------LTW--------VPNGAPETA---LDNTT-
UniRef90_Q65097_1_206      ----------THTGK------LTW--------VPXGAPVSX---LNNTT-
UniRef90_Q01126_1_208      ----------AHXXX------XXX--------XXXXXXXXX---XNNTT-
UniRef90_Q67461_13_173     ----------KHEGD------LTW--------VPNGASEKA---LDNTT-
UniRef90_Q00280_54_209     ----------THTGK------LTW--------VPXGAPVSA---LDNTT-
UniRef90_H9AY35_719_926    ----------VPRGQPGEYAMVKW--------LPVGTPFDL-RDSSL---
```

```
2WZR1                      --------------NPVVF---------------SRRGTTRFALPYTAPH
UniRef90_Q6PMU0_725_943    --------------NPVVF---------------STNNTTRFALPYTAPH
UniRef90_F1AW55_725_943    --------------NPVVF---------------SHNGTTRFALPYTAPH
UniRef90_A0PG93_1_219      --------------NPSHF---------------SHNGTTRFALPYTAPH
UniRef90_A0PG86_1_219      --------------NPVVF---------------SHNETTRFALPYTAPH
UniRef90_A0PG77_1_219      --------------NPVVF---------------SHNGTTRFALPYTAPH
UniRef90_Q6PMU2_725_943    --------------NPVVF---------------SHNGTTRFALPFTAPH
UniRef90_Q8V0G8_526_744    --------------NPVVF---------------SRDGATRFALPFTAPH
UniRef90_A0PG80_1_219      --------------NPVVF---------------SHNGTTRFALPFTAPH
UniRef90_Q8BDT2_1_218      --------------NPVIF---------------SHNGTTRFALPYTAPH
UniRef90_A0PG94_1_219      --------------NPVVF---------------SHNGVTRFALPYTAPH
UniRef90_C8YS70_1_219      --------------NPVVF---------------STNGATRFALPFTAPH
UniRef90_Q6PMT8_726_934    --------------NPMVF---------------SNNGVTRFAVPYTAPH
UniRef90_Q6PMT3_724_939    --------------NPVVH---------------SKGSVVRFALPYTAPH
UniRef90_B8Y0J7_726_934    --------------NPMVF---------------ATNGVTRFAVPFTAPH
UniRef90_E2IU95_1_209      --------------NPMVF---------------SHNGVTRFAIPYTAPH
UniRef90_Q80JY0_1_209      --------------NPMVF---------------SHNNVTRFALPYTAPH
UniRef90_A0PFY2_1_209      --------------NPMVY---------------AKGGVTRFAIPFTAPH
UniRef90_Q80K03_1_209      --------------NPMVF---------------SHNAVTRFAVPYTAPH
UniRef90_Q1L757_525_734    --------------NPMVF---------------SHNRVTRFAIPYTAPH
UniRef90_Q80K16_1_209      --------------NPMVY---------------AHNHVTRFAIPFTAPH
```

```
UniRef90_Q1L752_527_735    --------------NPMVF---------------SHNKVARFAIPFTAPH
UniRef90_A0PG17_1_209      --------------NPMVF---------------SHNGVPRFAIPFTAPH
UniRef90_Q80JY2_1_209      --------------NPMVF---------------AHNKVTRFAIPYTAPH
UniRef90_Q80JY8_1_209      --------------NPMVF---------------SHNKVTRFAVPYTAPH
UniRef90_Q8JVD0_525_739    --------------NPVVH---------------AKGGVTRFALPYTAPH
UniRef90_Q80JU2_1_209      --------------NPMVF---------------SHNNVTRFAIPYTAPH
UniRef90_A0PFY7_1_209      --------------NPMVY---------------SHNGVTRFAIPYTAPH
UniRef90_A0PFY4_1_209      --------------NPMVF---------------AHNGVTRFAVPYTAPH
UniRef90_Q80JZ5_1_205      --------------NPMVF---------------SHNKVTRFAIPYTAPH
UniRef90_A0PFZ0_1_209      --------------NPMVF---------------SHKKVTRFAIPFTAPH
UniRef90_Q1L748_525_740    --------------NPVVF---------------AKGGLARFALPYTAPH
UniRef90_Q80JY7_1_209      --------------NPMVF---------------SHNNVTRFAVPYTAPH
UniRef90_A0PG12_1_209      --------------NPMVF---------------SHNKVTRFAIPYTAPH
UniRef90_A0PFY8_1_209      --------------NPMVF---------------AHNGVTRFAIPYTAPH
UniRef90_Q80JZ1_1_209      --------------NPMVF---------------PHNKVTRFAVPYTAPH
UniRef90_Q80JU1_1_209      --------------NPMVF---------------AHNNVTRFALPYTRPH
UniRef90_B2MZR9_1_211      --------------NPTAY---------------NKAPFTRLALPYTAPH
UniRef90_P49303_726_936    --------------NPTAY---------------PKAPFTRLALPYTAPH
UniRef90_Q2I5Z0_71_281     --------------NPTAY---------------HKAPLTRLALPYTAPH
UniRef90_Q9DJ49_1_212      --------------NPTAY---------------HKAPLTRLALPYTAPH
UniRef90_A1EBZ1_1_211      --------------NPTAY---------------HKEPFTRLALPYTAPH
UniRef90_A1EBZ4_1_211      --------------NPTAY---------------NKAPFTRLALPYTAPH
UniRef90_I0AZ32_1_211      --------------NPTVY---------------KKEPFTRLALPYTAPH
UniRef90_A8D5K4_1_211      --------------NPTAY---------------HKAPLTRLALPYTAPH
UniRef90_Q2I5X3_80_290     --------------NPTAY---------------NKAPFTRLALPYTAPH
UniRef90_I0AYZ5_1_211      --------------NPTAY---------------NKAPFTRLALPYTAPH
UniRef90_I0AZ39_1_210      --------------NPTAY---------------NKAPFTRLALPYTAPH
UniRef90_H9T7U2_127_337    --------------NPTAY---------------HKAPLTRLALPYTAPH
UniRef90_Q9DJF9_1_211      --------------NPTAY---------------HKAXLTRLALPYTAPH
UniRef90_I0AYY0_1_211      --------------NPTAY---------------VKSPFTRLALPYTAPH
UniRef90_Q2I5X1_23_232     --------------NPTAY---------------RKEPFTRLALPYTAPH
UniRef90_B2ZHQ6_1_211      --------------NPTAY---------------HKAPLTRLALPYTAPH
UniRef90_A4ZPM0_1_211      --------------NPTAY---------------HKAPFTRLALPYTAPH
UniRef90_E7D638_1_210      --------------NPTAY---------------HKAPFTRLALPYTAPH
UniRef90_Q8JUM5_1_211      --------------NPTAY---------------HRAPFTRLALPYTAPH
UniRef90_Q8BED5_1_209      --------------NPTAY---------------QKQPITRLALPYTAPH
UniRef90_A0ZXF2_1_211      --------------NPTAY---------------YKAPITRLALPYTAPH
UniRef90_F1BVI2_1_211      --------------NPTAY---------------NKEPFTRLALPYTAPH
UniRef90_I0AYX9_1_210      --------------NPTAY---------------AKAPFTRLALPYTAPH
UniRef90_I0AZ13_1_211      --------------NPTAY---------------NKKPFTRLALPYTAPH
UniRef90_A4ZPL8_1_211      --------------NPTAY---------------HKAPFTRLALPYTAPH
UniRef90_Q6PMY9_724_932    --------------NPTAY---------------QKQPITRLALPYTAPH
UniRef90_Q8JUP6_1_211      --------------NPTAY---------------HKAPFTRLALPYTAPH
UniRef90_B1A0W6_1_210      --------------NPTVY---------------PKQPFTRLALPYTAPH
UniRef90_C0LRW1_1_211      --------------NPTAY---------------HKAPFTRLALPYTAPH
UniRef90_Q8JUN6_1_211      --------------NPTAY---------------HKAPFTRLALPYTAPH
UniRef90_A1EC01_1_211      --------------NPTAY---------------NKAPYTRLALPYTAPH
UniRef90_A2I8Z5_725_935    --------------NPTAY---------------HKEPLTRLALPYTAPH
UniRef90_B1A0W7_1_211      --------------NPTAY---------------HKQPFTRLALPYTAPH
UniRef90_Q9DJ38_1_212      --------------NPTAY---------------HKEPLTRLALPYTAPH
UniRef90_A1EC10_1_211      --------------NPTAY---------------NKAPFTRLVLPYTAPN
UniRef90_E2FZV2_1_211      --------------NPTAY---------------HKAPLTRLALPYTAPH
UniRef90_I0AYY6_1_211      --------------NPTGY---------------VKAPFTRLALPYIAPH
UniRef90_A7XPU3_1_209      --------------NPTAY---------------QKQPITRLALPYTAPH
UniRef90_Q999Y2_1_211      --------------NPTAY---------------KKAPFTRLALPYTAPH
UniRef90_B2MZR8_1_211      --------------NPTAY---------------NKAPFTRLALPYTAPH
UniRef90_A1EC03_1_211      --------------NPTAY---------------HKAPFTRLALPYTAPH
UniRef90_Q8BEA7_1_208      --------------NPTAY---------------QKKPITRLALPYTAPH
UniRef90_C0LYR0_1_209      --------------NPTAY---------------QKQPITRLALPYTAPH
UniRef90_C0LS35_1_211      --------------NPTAY---------------NKAPFTRLALPYTAPH
UniRef90_I0AZ16_1_210      --------------NPTAY---------------NKAPFTRLALPYTAPH
UniRef90_A4ZPP1_1_210      --------------NPTAY---------------HKAPFTRLALPYTAPH
UniRef90_A0ZXC4_1_211      --------------NPTAY---------------NKAPFTRLALPYTAPH
UniRef90_Q8JUS6_1_211      --------------NPTAY---------------XXXXXXXLALPYTAPH
UniRef90_A1EC12_1_211      --------------NPTAY---------------TKAPFTRLALPYTAPH
UniRef90_F6KYM9_1_211      --------------NPTAY---------------HKAPITRLALPYTAPH
UniRef90_B1A0X1_1_211      --------------NPTVY---------------PKQPFTRLALPYTAPH
UniRef90_I0AZ19_1_211      --------------YPTVY---------------RKEPLTRLALPYTAPH
UniRef90_Q912K6_1_211      --------------NPTAY---------------NKAPFTRLALPYTAPH
UniRef90_A4ZPL9_1_211      --------------NPTAY---------------HKAPFTRLALPYTAPH
UniRef90_Q9YPW9_1_211      --------------NPTAH---------------HKAPLTRLALPYTAPH
UniRef90_A1EC13_1_211      --------------NPTAY---------------NKAPFTRLALPYTAPH
UniRef90_B2MZQ3_1_211      --------------NPTAY---------------HKAPLTRLALPYTAPH
```

```
UniRef90_E2J011_1_211      --------------NPTAY---------------HKAPLTRLALPYTAPH
UniRef90_I0AZ31_1_211      --------------LPTAY---------------NXAPFTRLALPYAAPH
UniRef90_D7RFC4_1_211      --------------NPTAY---------------HKAPLTRLALPYTAPH
UniRef90_E2IZY3_1_211      --------------NPTAY---------------HKAPLTRLALPYTAPH
UniRef90_Q9DJ33_1_211      --------------TXTAY---------------HKAPLTRLALPYTAPH
UniRef90_Q9DJ21_1_192      --------------NPTAY---------------HKALLTRLALPYTAPH
UniRef90_Q0PV54_36_239     --------------NPTAY---------------PKQPFTRLALPYTAPH
UniRef90_C8YS67_1_209      --------------NPTAY---------------HKGPLTRLALPYTAPH
UniRef90_F6KYY3_1_211      --------------NPTAY---------------HKAPLTRLAVPYTAPH
UniRef90_B1A0W4_1_211      --------------NPTAY---------------HKAPFTRLALPYTAPH
UniRef90_B3FHW7_1_211      --------------NPTAY---------------NGTTFTRQALPYSAPH
UniRef90_A0ZXB5_1_211      --------------NPTAY---------------KKAPFTRLALPYTAPH
UniRef90_A1EBZ3_1_211      --------------NPTAY---------------HKEPFTRLALPYTAPH
UniRef90_Q9DJA4_1_210      --------------NPTAY---------------YKAPLTRLALPYTAPH
UniRef90_Q0QED4_407_613    --------------NPTAY---------------HKGPVTRLALPYTAPH
UniRef90_Q8JUU0_1_211      --------------NPTAY---------------NKAPFTRLALPYTAPH
UniRef90_Q9DJF0_19_211     --------------NPTAY---------------HKAXLTRLALPYTAPH
UniRef90_I0AZ37_1_211      --------------HPXAY---------------NKAPFTRLFFPYTGPQ
UniRef90_Q67462_1_211      --------------NPTAY---------------YKKPLTRLALPYTAPY
UniRef90_I0AZ36_1_211      --------------NPSDY---------------NKAPFTRLVXPYXAPH
UniRef90_Q91EM4_1_211      --------------NPXAY---------------HKAXLTXLELPYXAPH
UniRef90_Q0PV56_33_229     --------------NPTAY---------------HKEPFTRLALPYTAPH
UniRef90_C0LYW2_1_209      --------------NPTAY---------------QKQPITRLALPYTAPH
UniRef90_B2MZX2_1_207      --------------NPTAY---------------HKGPLTRLALPYTAPH
UniRef90_Q01124_1_209      --------------NPTAY---------------HKGPLTRLALPYTAPH
UniRef90_E2J004_1_192      --------------PPTAY---------------HKAPLTRLALPYTAPH
UniRef90_Q8JUL9_1_209      --------------XXXXX---------------XXXXXXXXXXPYTAPH
UniRef90_Q6X5M0_1_179      --------------NPTAY---------------HKAPFTRLALPYTAPH
UniRef90_Q2I5Y3_113_321    --------------NPTAY---------------NKAPFTRLALPYTAPH
UniRef90_Q01121_1_189      --------------NPTAY---------------HKGPLTRLALPYTAPH
UniRef90_Q6X5M1_1_179      --------------NPTVY---------------HKAPFTRLALPYTAPH
UniRef90_A3RGT7_2_181      --------------NPTAY---------------HKEPFTRLALPYTAPH
UniRef90_P03310_11_218     --------------NPTAY---------------HKGPLTRLALPYTAPH
UniRef90_B3FHW2_1_211      --------------NPACY---------------HNVPFMLFALPYSAPQ
UniRef90_Q8JUM1_1_211      --------------XXXXX---------------XXXPFTRLALPYTAPH
UniRef90_Q65051_1_200      --------------NPTAY---------------NKAPFTRLALPYTAPH
UniRef90_Q8JUL2_2_187      --------------NPTAY---------------HRAPFTRLALPYTAPH
UniRef90_B2BF63_11_200     --------------NPTAY---------------HKAPLTRLALPYTAPH
UniRef90_Q0PV58_32_197     --------------NPTAY---------------HKEPFTRLALPYTAPH
UniRef90_Q65102_9_211      --------------NPNAY---------------YKKRLTRLALPYTAPH
UniRef90_Q8BED3_1_210      --------------NPTAY---------------QKQPITRLALPYTAPH
UniRef90_A0PG53_1_160      --------------NPTAY---------------HKEPLTRLALPYTAPH
UniRef90_B3FHW0_1_211      --------------NAAVY---------------HKVPFTRFALPYSAPH
UniRef90_B3FHV9_1_211      --------------NAAVY---------------HKVAFTRFAMPFSAPH
UniRef90_B3FHV8_1_211      --------------NAAVY---------------YKVPFTGFALPYTALH
UniRef90_A0PG60_5_160      --------------NPTAY---------------HKAPLTRLALPYTAPH
UniRef90_Q91NQ7_2_163      --------------NPTAY---------------HKAPLTRLALPYTAPH
UniRef90_B3FHV6_1_211      --------------NAAFY---------------HKVPLTLLALPYSAPH
UniRef90_B3FHV7_1_211      --------------NAAVY---------------QKVPFTRFALPYSAPH
UniRef90_A0PG54_1_160      --------------NPTAY---------------HKKPLTRLALPYTAPH
UniRef90_B3FHW1_1_211      --------------NAAVY---------------HKVPFTRFALPFSAPH
UniRef90_Q65068_1_206      --------------XPTAY---------------XXGPVTRLALPYXAPX
UniRef90_Q9YPF0_1_208      --------------XPTAX---------------HXGPLTRLALPXXAPX
UniRef90_Q8BEF3_1_209      --------------NPTAY---------------QKQPTTRLALPYTAPH
UniRef90_B3FHV4_1_211      --------------NAAVY---------------QKVPLTLFALPYTAPH
UniRef90_Q8JUN9_1_211      --------------XXXXX---------------XXXXXXXXXXXXXXXX
UniRef90_Q9YPG1_1_165      --------------NPTAY---------------NKAPFTRLALPYTAPH
UniRef90_A0PG23_5_162      --------------NPTAY---------------HKAPLTRLALPYTAPH
UniRef90_Q9DJ61_1_156      --------------NPTAY---------------HKAXLTRLELPYTAXH
UniRef90_Q65097_1_206      --------------NPTAY---------------HKGXVTXLAXPYTAPH
UniRef90_Q01126_1_208      --------------NPTAY---------------HKGPLTXLAXPYTAPH
UniRef90_Q67461_13_173     --------------NPTAY---------------YKKPLTRLALPYTAPH
UniRef90_Q00280_54_209     --------------NPTAY---------------XXXXXXXXXXXYTAPH
UniRef90_H9AY35_719_926    --------------DGLALQGLDSTCSVGFAGSAGNGSAAVMAIPYNSPM
```

```
2WZR1                      RVLATVYNGDCKYKPTGTAPRENIRGDLATLAARI-ASETH-IP-TTFNY
UniRef90_Q6PMU0_725_943    RCLATSYNGDCKYKPAGTTPRENIRGDLATLAGRI-ASETH-IP-TTFNY
UniRef90_F1AW55_725_943    RLLATTYNGDCKYKPAGQEPRTAIRGDLAVIAERI-ASETH-IP-TSFNY
UniRef90_A0PG93_1_219      RLLATVYNGDCKYKPTEQPPRTNIRGDLAVLAQRI-ASKTH-IP-TSFNY
UniRef90_A0PG86_1_219      RCLATAYNGDCKYKPNSEAPRTHIRGDFTTLAERI-ASETH-IP-TTFNY
```

```
UniRef90_A0PG77_1_219      RCLATAYNGDCKYKPNAEAPRTHIRGDLAVLAERI-ASETH-IP-TTFNY
UniRef90_Q6PMU2_725_943    RVLATVYNGGCKYKPTNEDARTNIRGDLATLAERI-REQSH-IP-TTFNY
UniRef90_Q8V0G8_526_744    RVVATSYNGNCKYQDQSTTPLTHVRGDLATLAERI-AQNAH-IP-TSFNY
UniRef90_A0PG80_1_219      RVLATVYNGDCKYKPINEDTRTNIRGDLATLAARV-REQSH-IP-TTFNY
UniRef90_Q8BDT2_1_218      RVLATTYNGDCKYKAQPVENRE-IRGDLAVLAVRV-AEETH-IP-TTFNY
UniRef90_A0PG94_1_219      RVLATVYNGNCRYKPNTEAPRPTIRGDLATLAARI-AEQAH-IP-TTFNY
UniRef90_C8YS70_1_219      RLLATQYAGDCKYKQQPETPRENIRGDLATLAARI-SSESH-IP-TTFNY
UniRef90_Q6PMT8_726_934    RLLSTVYNGECEYGTTVTA----IRGDRAVLAAKY-ANTRHTLP-STFNF
UniRef90_Q6PMT3_724_939    GVLATVYNGNCKYSETQRV--TSRRGDLAVLAQRVENETTRCLP-TTFNF
UniRef90_B8Y0J7_726_934    RLLSTVYNGECEYKTTVSA----IRGDRXVLANKY-AGVKHALP-STFNF
UniRef90_E2IU95_1_209      RLLATRYNGECKYTDRSPA----IRGDRAVLAAKY-ANSRHTLP-STFNF
UniRef90_Q80JY0_1_209      RLLSTRYNGECKYTQTSGA----IRGDRAVLAAKY-ANTKHELP-STFNF
UniRef90_A0PFY2_1_209      RLLSTVYNGECTYTKTATA----IRGDRAALAAKY-ADNVHTLP-QTFNF
UniRef90_Q80K03_1_209      RLLSTVYNGECRYTERVTA----IRGDRAVLAAKY-ANTRHALP-STFNF
UniRef90_Q1L757_525_734    RLLSTVYNGECVYADTVAP----IRGDRQVLAAKY-SNRKHQLP-STFNY
UniRef90_Q80K16_1_209      RLLSTVYNGECEYRTNVTA----IRGDRQVLAQKY-ASTKHALP-STFNF
UniRef90_Q1L752_527_735    RLLSTVYNGECNYSTSVTP----IRGDRAVLAAKY-ASTKHTLP-STFNF
UniRef90_A0PG17_1_209      RLLSTVYNGECDYNSKVTA----IRGDRQVLQQKY-ASTRHALP-STFNF
UniRef90_Q80JY2_1_209      RLLSTVYNGECEYTKTVSA----IRGDRAVLAAKY-ASGKHTLP-STFNF
UniRef90_Q80JY8_1_209      RLLSTRYNGECKYTQRSTR----IRGDPAVLAAKY-ANTKHELP-STFNF
UniRef90_Q8JVD0_525_739    GVLATVYNGNCKYSKTQHV--VPRRGDLAVLAQRVENETTRCRP-TTFNF
UniRef90_Q80JU2_1_209      RLLSTVYNGECKYDTTVQP----IRGDRAVLANKY-ANRKHTLP-STFNF
UniRef90_A0PFY7_1_209      RLLSTVYNGECDYKKASTA----IRGDRAVLAAKY-ANTKHTLP-STFNF
UniRef90_A0PFY4_1_209      RVLATRYNGECKYTDRVSP----IRGDRAVLAAKY-ADSRHSLP-STFNF
UniRef90_Q80JZ5_1_205      RLLATRYNGECKYTQEARA----IRGDRAVLAAKY-AGAKHALP-STFNF
UniRef90_A0PFZ0_1_209      RLLSTVYNGECKYTEKTIA----IRGDRAVLAQKY-ASTKHALP-STFNF
UniRef90_Q1L748_525_740    QVLATVYNGTCVYRKNAPV--TPRRGDLQALQARVDAERERCIP-TSFNF
UniRef90_Q80JY7_1_209      RLLSTRYNGECKYTQQSTA----IRGDRAVLASKY-SNIKHELP-STFNF
UniRef90_A0PG12_1_209      RLLSTVYNGECAYSKPVSA----IRGDRQALAAKY-ASGRHTLP-STFNF
UniRef90_A0PFY8_1_209      RLLSTVYNGECEYNKTSNP----IRGDRAVLAAKH-KDVAHSLP-STFNF
UniRef90_Q80JZ1_1_209      RLLSTVYNGECTYVHKTQA----IRGDRAVLAQKY-ANTKHSLP-STFNF
UniRef90_Q80JU1_1_209      RLLATHYNGECTYTKKVVP----IRGDRAALRAKY-ADTKHSLP-STFNF
UniRef90_B2MZR9_1_211      RVLATVYNGTCKYSTGT-T---GRQGDLESLAARV----ATQLP-TSFNY
UniRef90_P49303_726_936    RVLATVYNGTGKYSAGG-M---GRRGDLEPLAARV----AAQLP-TSFNF
UniRef90_Q2I5Z0_71_281     RVLATVYNGNCKYGVSPMT---NVRGDLQVLAQKA----ARTLP-TSFNY
UniRef90_Q9DJ49_1_212      RVLATAYNGECRYGTHTTT---NVRGDLQVLAQKA----ARTLP-TSFNY
UniRef90_A1EBZ1_1_211      RVLATVYNGTSKYSATG-A---STRGDLGSLAARV----AEQLP-ASFNF
UniRef90_A1EBZ4_1_211      RVLATVYNRTCKYSATG-G---RMRGDLGTFAARV----AAQLP-ASFNF
UniRef90_I0AZ32_1_211      RVLATVYNGTSRYASSG-S---SGRGDMGSLAARV----ARQLP-SSFNY
UniRef90_A8D5K4_1_211      RVLATVYNGDCKYGKSPVT---NLRGDLQVLTQKA----ARTLP-TSFNY
UniRef90_Q2I5X3_80_290     RVLATVYNGTSKYSTTG-G---RTRGDLGALAARV----ATQLP-ASFNF
UniRef90_I0AYZ5_1_211      RVLATVYNGVNKYAESG-S---NRRGDLGSLAARV----AKRLP-ASFNF
UniRef90_I0AZ39_1_210      RVLATVYNGTSKYSVST-S---GRRGDLGSLAARV----AKHLP-ASFNF
UniRef90_H9T7U2_127_337    RVLATVYNGNCKYGDSPVT---NVRGDLQVLAQKA----ARTLP-TSFNY
UniRef90_Q9DJF9_1_211      RVLATVYNGNCKYAQGPLA---NVRGDLQVLAQKA----ARPLP-TSFNY
UniRef90_I0AYY0_1_211      RVLATVYNGVNKYTVSS-S---GRRGDLGALAARV----ARQLP-TSFNY
UniRef90_Q2I5X1_23_232     RVLATVYNGTNKY-STT-G---SRRGDLGALAARV----AAQLP-ASFNF
UniRef90_B2ZHQ6_1_211      RVLATVYNGSCKYAEGSLT---KVRGDLQVLAQKA----AKPLP-TSFNY
UniRef90_A4ZPM0_1_211      RVLATVYNGTSKYSVTT-S---PRRGDLGSLAARV----AAQLP-ASFNY
UniRef90_E7D638_1_210      RVLATVYNGASKYSVPA-A----RRGDLGSLAARL----AAQLP-ASFNY
UniRef90_Q8JUM5_1_211      RVLATVYNGTSKYSATV-T---DGRGDLGALAAKV----AAQLP-ASFNF
UniRef90_Q8BED5_1_209      RVLATVYNGKTTYGETT-P----RRGDMAALAQRL----MGQLP-TSFNY
UniRef90_A0ZXF2_1_211      RVLATVYNGECKYTNDNAP---NVRGDLQVLARKA----ARSLP-TSFNY
UniRef90_F1BVI2_1_211      RVLATVYNGTSKYSTGS-G---RTRGDLGQLAART----AAQLP-ASFNF
UniRef90_I0AYX9_1_210      RVLATVYNGTNKYS-TA-T---TRRGDLGALAARI----ASQLP-ASFNY
UniRef90_I0AZ13_1_211      RVLATVYNGTSEYAVSG-P---DSRGDTGPLAARA----ANQLP-ASFNY
UniRef90_A4ZPL8_1_211      RVLATVYNGTSKYSTGA-S---PRRGDLGVLAARV----ATQLP-SSFNY
UniRef90_Q6PMY9_724_932    RVLATVYNGKTTYGEQS-T----RHGDLATLAQGV----SNRLP-TSFNY
UniRef90_Q8JUP6_1_211      RVLATVYNGTSKYSATG-E---HRRGDLGQLAARV----AAQLP-ASFNF
UniRef90_B1A0W6_1_210      RVLATVYNGVSKYSTTG-G---GRRGDLGSLAARV----AAQLP-ASFNF
UniRef90_C0LRW1_1_211      RVLATVYNGTSKYSTAG-I---GRRGDLGALAARV----AAQLP-ASFNF
UniRef90_Q8JUN6_1_211      RVLATVYNGTNKYTAPN-T---GRRGDLGPLAARV----AAQLP-ASFNF
UniRef90_A1EC01_1_211      RVLATVYNGTNKYSAAS-G---RTRGDLGQLAARV----AAQLP-ASFNF
UniRef90_A2I8Z5_725_935    RVLATVYNGSSKYGDTSTN---NVRGDLQVLAQKA----ERTLP-TSFNY
UniRef90_B1A0W7_1_211      RVLATVYNGVSRYSTTG-N---GRRGDLGPLAARV----AAQLP-SSFNF
UniRef90_Q9DJ38_1_212      RVLATVYNGNCKYTSVAMA---NGRGDLQVLTQKA----ARPLP-TSFNY
UniRef90_A1EC10_1_211      RLLVTVYNGTNKYSAAS-G---RTRGDLGAVAARI----AAQLP-ASFNF
UniRef90_E2FZV2_1_211      RVLATVYNGSCRYSGAVTP---NVRGDLQVLARRA----APMLP-TSFNF
UniRef90_I0AYY6_1_211      RVLATVYNGTNKYSASA-P---NRRGDLGALAARL----AKQLP-ASFNY
UniRef90_A7XPU3_1_209      RVLATVYNGKTAYGETT-T----RRGDLAALAQRV----SRQLP-PSFNY
UniRef90_Q999Y2_1_211      RVLATVYNGTSEYTVSG-S---NGRGDMAPLAARV----AKTLP-ASFNY
UniRef90_B2MZR8_1_211      RVLATVYNGTSKYTVGG-S---GRRGDMGSLAARV----AEQLP-ASFNY
UniRef90_A1EC03_1_211      RVLATVYNGTNKYSAAS-G---RTRGDLGTVAERT----ATQLP-ASFNF
```

```
UniRef90_Q8BEA7_1_208      RVLATVYNGETTYGETN-P----RRGDLAVLAQRE----SKRLP-TSFNY
UniRef90_C0LYR0_1_209      RVLATVYNGKTAYGAET-P----RRGDLAAIAQRV----NSSLP-TSFNY
UniRef90_C0LS35_1_211      RVLATVYNGTSRYSTTG-G---RTRGDLGALAARV----SAQLP-ASFNF
UniRef90_I0AZ16_1_210      RVLATVYNGTSKYSTSS-P---G-RGDVGPLAARV----VKQLP-ASFNY
UniRef90_A4ZPP1_1_210      RVLATVYHGTSKYSTPG-T----RRGDLGSFAARV----AAQLP-TSFNF
UniRef90_A0ZXC4_1_211      RVLATVYNGTSKYTVSG-S---SRRGNLGSLAARV----AKALP-ASFNY
UniRef90_Q8JUS6_1_211      RVLATVYNGTNKYSTAG-T---GRRGDLGALAARV----AAQLP-ASFNF
UniRef90_A1EC12_1_211      RVLATVYNGTNKYSAAS-G---RTRGDLGQLAARV----AAQLP-ASFNF
UniRef90_F6KYM9_1_211      RVLATVYNGSCKYSSGAVP---NVRGDLRVLAQKA----TRSLP-TSFNY
UniRef90_B1A0X1_1_211      RVLATVYNGVNKYYTAH-S---GRRDDLGSLEARV----AAQLP-ASFNF
UniRef90_I0AZ19_1_211      RVLATVYNGTSKYTTSD-S---SRRGDMGALAARV----AKQLP-ASFNY
UniRef90_Q912K6_1_211      RVLATEYNGTNRYTVSG-S---NRRGDLGSLAARV----AKALP-ASFNY
UniRef90_A4ZPL9_1_211      RVLATVYNGTTKYATES-S---GRRGDLGPLAARV----AAQLP-ASFNY
UniRef90_Q9YPW9_1_211      RVLATVYNGSCKYSDARVS---NVRGDLQVLAQKA----ERALP-TSFNY
UniRef90_A1EC13_1_211      RVLATVYNGTSKYSAPG-L---CTRGDLGYPGARV----AAELP-ASFNF
UniRef90_B2MZQ3_1_211      RVLATVYNGECRYSRNAVP---NLRGDLQVLAQKV----ARTLP-TSFNY
UniRef90_E2J011_1_211      HVLATVYNVTCRYCRAPVT---NVRGDLQVLSQKA----ARTLP-TSFNY
UniRef90_I0AZ31_1_211      RVLATVYNGTSRYAVSG-S---GRRGDLGPLAARV----AKALP-ASFNY
UniRef90_D7RFC4_1_211      RVLATVYNGNCKYAGGPLT---NVRGDLQVLAQKA----ARPLP-TSFNY
UniRef90_E2IZY3_1_211      RVLATVYNGNCKYGGTPVT---NVRGDLQVLAQKA----ARTLP-TSFNY
UniRef90_Q9DJ33_1_211      RVLATVYNGNCRYGRAPVT---NVRGDLQVLAQKA----ARTLP-TSFNY
UniRef90_Q9DJ21_1_192      RVLATVYNGNCKYXDAAVT---NVRGDLQVLAQKA----ARSLP-TSFNY
UniRef90_Q0PV54_36_239     RVLATVYNGVNKYSAAS-G---GRRGDLGSLAARV----AAQLP-ASFNF
UniRef90_C8YS67_1_209      RVLATVYTGTATYATAN-P----RRGDPGQLAATR----SRHLP-ASFNF
UniRef90_F6KYY3_1_211      RVLATVYNGRCEYSKNPAS---NVRGDLQVLAQKA----ARALP-TSFNY
UniRef90_B1A0W4_1_211      RVLATVYNGVNKYSSTS-G---GRRGDLGPLATRV----AAQLP-SSFNF
UniRef90_B3FHW7_1_211      RGLATVCNGTNKYSAGS-G---RRRGDLGPLAARI----AAELP-ASFNF
UniRef90_A0ZXB5_1_211      RVLATVYNGVSKYTANG-S---NRRGDMAALAARV----AKALP-ASFNY
UniRef90_A1EBZ3_1_211      RVLATVYNGTSKYSVSG-S---NRRGDLGSPRSAV----RKQLP-ASFNY
UniRef90_Q9DJA4_1_210      RVLATVYNGNYKYDDTPVT---NVRGDLQVLAQKA----ARTLP-TSFNY
UniRef90_Q0QED4_407_613    RVLATAYTGTTTY-TAS-A-----RGDLAHLTTTH----ARHLP-TSFNF
UniRef90_Q8JUU0_1_211      RVLATVYNGTSKYSAXX-X---XXXXXXXXXXXXX----AAQLP-ASFNY
UniRef90_Q9DJF0_19_211     RVLATVYNGNCKYAEGPLA---NVRGDLQVLAQKA----ARALP-TSFNY
UniRef90_I0AZ37_1_211      RVXGTVYXGVXKYAVGG-S---GRRGDLGSLAARV----ARQLP-ASFXY
UniRef90_Q67462_1_211      RVLATVYNGECTYSSNAVP---NVRGDPNLLEQKA----ARMLP-AYFNY
UniRef90_I0AZ36_1_211      GVXATVYNGVNKYAVGG-S---SRRGDSGPLAARV----ARQLP-ASFKY
UniRef90_Q91EM4_1_211      RVLATVYNGNCKYGESPVT---NVRGDLQVLAQKA----ARTLP-TSFNY
UniRef90_Q0PV56_33_229     RVLATVYNGTNKYAVTG----------------------AKQLP-SSFNF
UniRef90_C0LYW2_1_209      RVLVNSVQRKTTYGGET-S----ERGDFAALTQRL----SNWLP-TSFNY
UniRef90_B2MZX2_1_207      RVLATAYTGTTTY-T-S-T----RGGDLTHLAAAH----ARRLP-TSFNF
UniRef90_Q01124_1_209      RVLATRYTGTTTY-TTS-A----RRGDLAQLAAAH----ARHLP-TSFNF
UniRef90_E2J004_1_192      RVLATVYNGNCKYGGPPVT---NVRGDLQVLAQKA----ARTLP-ISFNY
UniRef90_Q8JUL9_1_209      RVLATVYNGTSKYSARR-S-----RGDLGSLATRV----XTQLP-ASFNY
UniRef90_Q6X5M0_1_179      RVLATVYNGTNKYSTGG-S---TRRGDLGALAARV----AAQLP-ASFNY
UniRef90_Q2I5Y3_113_321    RVLATVYNGTSKYSAG--E---RTRGDLGALAARV----ATQLP-ASFNF
UniRef90_Q01121_1_189      RVLATAYTGTTTY-TTS-A----RRGDLAHLAATH----ARRLP-TSFNF
UniRef90_Q6X5M1_1_179      RVLATVYNGTSKYSTGG-S---SRRGDLGSLAARV----AAQLP-TSFNY
UniRef90_A3RGT7_2_181      RVLATVYNGTNKY-AAT-G---ARRGDLGSLAARV----AAQLP-SSFNF
UniRef90_P03310_11_218     RVLATTYTGTTAY-TAS-A----RRGDLAHLAAAH----ARHLP-TSFNF
UniRef90_B3FHW2_1_211      FVMATGYHRDCLYSPNPII---NVRGDLQVLPQKG----ARTLP-TCFNY
UniRef90_Q8JUM1_1_211      RVLATVYNGTNKYSTAG-T---GRRGDLGALAARV----AAQLP-ASFNF
UniRef90_Q65051_1_200      RVLATVYNGTNKYSTDG-P----RRDDMGSPAARA----AKQLP-ASFNY
UniRef90_Q8JUL2_2_187      RVLATVYNGTSKXXXXX-X---XXXXXXXXXAAKV----AAQLP-ASFNF
UniRef90_B2BF63_11_200     RVLATVYNGNCKYGESHTT---NVRGDLQVLAQKA----ARTLP-TSFNY
UniRef90_Q0PV58_32_197     RVLATVYNGTNKY-AAT-G---ARRGDLGSLAARV----AAQLP-SSFNF
UniRef90_Q65102_9_211      RVLATVYNGECKYSSXAVP---NVRGDLNVLEQKA----ARTLP-TSFNY
UniRef90_Q8BED3_1_210      RVLATVYNGKTTYGETT-P----RRGDMAALAQRL----MGQLP-TSFNY
UniRef90_A0PG53_1_160      RVLATVYNGNCKYGGAPPT---NRRGDLQVLAQKA----ARPLP-TSFNY
UniRef90_B3FHW0_1_211      CVMATGYNGVCLYCQSPIT---NLRGDLQVLPQKG----ARTLP-TSFNY
UniRef90_B3FHV9_1_211      FVMTTGYNGVCLYSPNPRT---NLRGDLRALPQKV----ARTLP-SSFKY
UniRef90_B3FHV8_1_211      FVMATVYNGVCLYSPNPIS---NVRGDLQVLPQKV----AGTLP-TSFNY
UniRef90_A0PG60_5_160      RVLATVYNGNCRYGNAPVN---NVRGDLQVLAQKA----ARTLP-TSFNY
UniRef90_Q91NQ7_2_163      RVLATVYNGSCKYSRVEVP---KVRGDLQVLAQRA----ARTLP-TSFNF
UniRef90_B3FHV6_1_211      LVMGTVYPGNCLYSPNPII---NVRGDLQVLPQKG----ARTLP-SCFNY
UniRef90_B3FHV7_1_211      LVMRTGYHGVCLYSPNPIS---NVRGDLQVLPQKV----SRTLP-TSFNY
UniRef90_A0PG54_1_160      RVLATVYNGNCKYDTAPLT---NVRGDLQVLAQKA----ARTLP-TSFNY
UniRef90_B3FHW1_1_211      CVMGTGYNGVCLYSQNPRT---NLRGDLQVLPQKV----ARALP-TSFNY
UniRef90_Q65068_1_206      RVLATAYTGTTTY-TAS-A-----RGDLAHLTTTH----AXHLP-TSFNF
UniRef90_Q9YPF0_1_208      RVLATTYTGTTTX-TTS-A----RRGDLAHLATAH----ARHLP-TSFXF
UniRef90_Q8BEF3_1_209      RVLATVYNGKTTYGETT-S----RRGDMAVLAQRL----MGRLP-TSFNY
UniRef90_B3FHV4_1_211      RVMRTGYHGVCLYSPNPIT---NVRGDLRVLPPKV----VRTLP-TSFNY
UniRef90_Q8JUN9_1_211      XXXXXXXXXXXKYSTAG-T---GRRGDLGALAARV----AAQLP-ASFNF
UniRef90_Q9YPG1_1_165      RVLATVCNGTNKYSTDG-P----RRGDMGXXXXXA----AKQLP-ASFNY
```

```
UniRef90_A0PG23_5_162     RVLATVYNGNCRYGEVSVT---NVRGDLQVLAQKA----ARTLP-TSFNY
UniRef90_Q9DJ61_1_156     RVLATVYNGNCKYGESSVT---NVRGDLQVLAQKA----ARTLP-TSFNY
UniRef90_Q65097_1_206     XVLATAYTGTTTY-IAS-A-----RGDLAHLKTTH----ARHLP-TSFNF
UniRef90_Q01126_1_208     RVLATEXAGTTTY--TT-T----RRGDLAQLATSR----AXRLP-TSFNF
UniRef90_Q67461_13_173    RVLATVYNGECKYSSNAVP---NVRGDPNVLEQKA----ARTLP-XXXXX
UniRef90_Q00280_54_209    RVLATTYTGTTAY-TTS-V----RRGDLAXLAATH----ARHLP-TSFNF
UniRef90_H9AY35_719_926   RAIPTKYAGTTQYTHVTPA-----------------------RP-GTANF


2WZR1                     GMI----YTQAEVD-----VYLRMKRAELYCPRPVLTHYDHNGRDRYKTT
UniRef90_Q6PMU0_725_943   GRI----YTQADVD-----VYVRMKRAELYCPRPLLTHYDHAGKDRYKTA
UniRef90_F1AW55_725_943   GQL----YTESEVD-----VYVRMKRAELYCPRPLLTHYDHDGKDRYKVA
UniRef90_A0PG93_1_219     GQI----YTESEVD-----VYVRMKRAELYCPRPVLTHYDHGDKDRYKIE
UniRef90_A0PG86_1_219     GRI----YTEAEVD-----VYVRMKRAELYCPRPVLTHYDHQGRDRYKVA
UniRef90_A0PG77_1_219     GRI----YTEADVD-----VYVRMKRAELYCPRPVLTHYDHKGRDRYKVA
UniRef90_Q6PMU2_725_943   GIV----LTEAQVD-----VYVRMKRAELYCPRPLLTTYDHASTDRYKVS
UniRef90_Q8V0G8_526_744   GQI----YTEAEVD-----VYVRMKRAETYCPRPLLTHYDHAGKNRYKTK
UniRef90_A0PG80_1_219     GII----LTEAEVD-----VYVRMKRAELYCPRPVLTTYDHASADRYKVP
UniRef90_Q8BDT2_1_218     GMI----LTESEVD-----VYVRMKRAELYCPRFLLTTYDHDGADRYKTT
UniRef90_A0PG94_1_219     GII----LTEGEVD-----VYVRMKRAELYCPRFVLTMYDHAGQDRRKIR
UniRef90_C8YS70_1_219     GII----ITEAEVD-----VYVRMKRAELYCPRFLLTTYDHQTAERYKTK
UniRef90_Q6PMT8_726_934   GHV----TADQPVD-----VYYRMKRAELYCPRPLLPAYNHADRDRFDAP
UniRef90_Q6PMT3_724_939   GRL----LCEEG-D-----AYYRMKRAELYCPRPLRVRYTHTT-DRYKTP
UniRef90_B8Y0J7_726_934   GFV----TVDKPVD-----VYYRMKRAELYCPRPLLVTYDHANRDRFDAP
UniRef90_E2IU95_1_209     GHV----TADQPVD-----VYYRMKRAELYCPRPLLPAYQHGTRDRFDAP
UniRef90_Q80JY0_1_209     GFV----TADEPVD-----AYYRMKRAELYCPRPLLPAYDHGSRDRFDAP
UniRef90_A0PFY2_1_209     GFV----TVDKPVD-----VYYRMKRAELYCPRPLLPAYDHASRDRFDAP
UniRef90_Q80K03_1_209     GHV----TADKPVD-----VYYRMKRAELYCPRPLLPAYDHANRDRFDSP
UniRef90_Q1L757_525_734   GYV----TADEPVD-----VYYRMKRAEFYCPRALLPAYDHHSRDRFDSP
UniRef90_Q80K16_1_209     GFV----TTDKPVD-----VYYRMKRAELYCPRPLLPAYDHQNRDRFDAP
UniRef90_Q1L752_527_735   GYV----TADAPVD-----VYYRMKRSELYCPRPLLPAYDHQSHDRFDAP
UniRef90_A0PG17_1_209     GFV----TADKPVD-----VYYRMKRAELYCPRPLLPAYDHKNRDRFDAP
UniRef90_Q80JY2_1_209     GFV----TADKPVN-----VYYRMKRAELYCPRPFLPAYDHASRDRFDAP
UniRef90_Q80JY8_1_209     GYV----TADKPVD-----VYYRMKRAELYCPRPLLPGYDHADRDRFDSP
UniRef90_Q8JVD0_525_739   GRL----LCDTG-D-----VYYRMKRAELYCPXALRVRYAHTT-DRYKTK
UniRef90_Q80JU2_1_209     GYV----TADKPVD-----VYYRMKRAELYCPRPLLPAYDHQSRDRFDAP
UniRef90_A0PFY7_1_209     GHV----TADAPVD-----VYYRMKRAELYCPRALLPAYDHVGRDRFDAP
UniRef90_A0PFY4_1_209     GHV----TADRPVD-----VYYRMKRAELYCPRPLLPAYQHNNRDRFDAP
UniRef90_Q80JZ5_1_205     GHV----TADAAVD-----VYYRMKRAELYCPRPLLPAYEHLSRDRF---
UniRef90_A0PFZ0_1_209     GFV----TADKPVD-----VYYRMKRAELYCPRALLPAYTHQDRDRFDAP
UniRef90_Q1L748_525_740   GRL----YAESG-E-----LYLRMKRAELYCPRWIFVRYTHTT-DRYKVP
UniRef90_Q80JY7_1_209     GYV----TADKPVS-----VYYRMKRTELYCPRPLLPAYDHADRGRFDAP
UniRef90_A0PG12_1_209     GFV----TADKPVD-----VYYRMKRAELYCPRALLPAYDHANRDRFDAP
UniRef90_A0PFY8_1_209     GFV----TADKPVD-----VYYRMKRAELYCPRPLLPAYSHNGRDRFDAP
UniRef90_Q80JZ1_1_209     GHV----TADEAVD-----VYYRMKRAELYCPRPLLPAYEHNDRDRYDGP
UniRef90_Q80JU1_1_209     GFV----TADKPVD-----VYFRMKRTELYCPRALLPAYDHAHNDRFDAP
UniRef90_B2MZR9_1_211     GAV----KATKIHE-----LLVRMKRAELYCPRPLL-AVEVSAQDRHKQK
UniRef90_P49303_726_936   GAI----QATTIHE-----LLVRMKRAELYCPRPLL-AVEVSSQDRHKQK
UniRef90_Q2I5Z0_71_281    GAI----KATRVIE-----LLYRMKRAETYCPRPLL-AVH-PSEARHKQK
UniRef90_Q9DJ49_1_212     GAI----KATRVIE-----LLYRIKRAETYCPRPLL-AIH-PSEARHKQK
UniRef90_A1EBZ1_1_211     GAI----RATTIHE-----LLVRMKRAELYCPRPLL-AVEVSSQDRYKQK
UniRef90_A1EBZ4_1_211     GAI----RATNIHE-----LLVRMKRAELYCPRPLL-AVEVTVQDRHKQK
UniRef90_I0AZ32_1_211     GAI----KADTIHE-----LLVRMKRAELYCPRPLL-AVEVFGQDRHKQK
UniRef90_A8D5K4_1_211     GAI----KATRVTE-----LLYRMKRAETYCPRPLL-AIH-PSETRHKQK
UniRef90_Q2I5X3_80_290    GAI----RATDISE-----LLVRMKRAELYCPRPLL-AVEVTAQDRHKQK
UniRef90_I0AYZ5_1_211     GAI----KADTIHE-----LLVRMKRAELYCPRPLL-ALEVSSQDRHKQK
UniRef90_I0AZ39_1_210     GAI----RATNIHE-----LLVRMKRAELYCPRPLL-AVE-ASQDRYKQK
UniRef90_H9T7U2_127_337   GAI----KATRVTE-----LLYRMKRAETYCPRPLL-AIH-PSGARHKQK
UniRef90_Q9DJF9_1_211     GAI----KATRVTE-----LLYRMKRAETYCPRPLL-AIQ-PNEARHKQK
UniRef90_I0AYY0_1_211     GAI----RAQTIHE-----LLVRMKRAELYCPRPLL-AVAVSSQDRHKQK
UniRef90_Q2I5X1_23_232    GAI----RATNIHE-----LLVRMKRAELYCPRPLL-STEVTTQDRHKQR
UniRef90_B2ZHQ6_1_211     GAI----KATQVTE-----LLYRMKRAETYCPRPLL-AVH-PNEARHKQK
UniRef90_A4ZPM0_1_211     GAI----RATTIHE-----LLVRMKRAELYCPRPLL-AVAVSSTDRHKQK
UniRef90_E7D638_1_210     GAV----RATEIQE-----LLVRMKRAELYCPRPLL-AVEVSSQDRHKQK
UniRef90_Q8JUM5_1_211     GAI----RATTIHE-----LLVRMKRAELYCPRPLL-AVKVSSQDRHKQK
UniRef90_Q8BED5_1_209     GAV----KADTITE-----LLIRMKRAETYCPRPLL-ALD-TTQDRRKQK
UniRef90_A0ZXF2_1_211     GAI----KATRVTE-----LLYRMKRAETYCPRPLL-AIH-PTEARHKQK
UniRef90_F1BVI2_1_211     GAI----KADGIHE-----LLVRMKRAELYCPRPLL-AVEVSSHDRHKQK
UniRef90_I0AYX9_1_210     GAI----RAQTIHE-----LLVRMKRAELYCPRPLL-AVEVSSQDRHKQK
UniRef90_I0AZ13_1_211     GAI----KAKVIHE-----LLVRMKRAELYCPRPLL-AVEVSSQDRYKQK
UniRef90_A4ZPL8_1_211     GAL----RAETIQE-----LLVRMKRAELYCPRPLL-PAEVGSADRHKQK
```

```
UniRef90_Q6PMY9_724_932    GAV----KADTITE-----LLIRMKRAETYCPRPLL-ALD-TTQDRRKQE
UniRef90_Q8JUP6_1_211      GAI----QATTIHE-----LLVRMKRAELYCPRPLL-AVEVSFQDRHKQK
UniRef90_B1A0W6_1_210      GAI----RATNIQE-----LLVRMKRAELYCPRPLL-PVEVLSQDRYKQK
UniRef90_C0LRW1_1_211      GAI----RATTIHE-----LLVRMKRAELYCPRPLL-AVEVSTQDRHKQK
UniRef90_Q8JUN6_1_211      GAI----RATTISE-----LLVRMKRAELYCPRPLL-AVEVSANDRHKQK
UniRef90_A1EC01_1_211      GAI----RATAIHE-----LLVRMKRAELYCPRPLL-AVEVSSQDRHKQK
UniRef90_A2I8Z5_725_935    GAI----KATRVTE-----LLYRMKRAETYCPRPLL-AIQ-PSTARHKQK
UniRef90_B1A0W7_1_211      GAI----RATTIHE-----LLVRMKRAELYCPRPLL-AVEVLSQDRHKQK
UniRef90_Q9DJ38_1_212      GAI----KATRLVE-----LLYRMKRAEAYCPRPLL-AIQ-PSEARHKQK
UniRef90_A1EC10_1_211      GAI----KADAIHE-----LLVRMKRAELYCPRPLL-AVEVSSQDRHKQK
UniRef90_E2FZV2_1_211      GAI----KATRVTE-----LLYRMKRAETYCPRPLL-AFH-PSEARHKQK
UniRef90_I0AYY6_1_211      GAI----KADTIHE-----LLVRMKRAELYCPRPLL-AVEVSSQDRHKQK
UniRef90_A7XPU3_1_209      GAV----NAESITE-----LLIRIKRAETYCPRPLL-ALD-TVQERRKQE
UniRef90_Q999Y2_1_211      GAI----KADNIHE-----LLVRMKRAELYCPRPLL-ATEVPSQGRYKQK
UniRef90_B2MZR8_1_211      GAI----KATDIHE-----LLVRMKRAELYCPRPLL-AVEVSSQDRHKQK
UniRef90_A1EC03_1_211      GAI----KATTIHE-----LLVRMKRAELYCPRPLL-AVEVSSQDRHKQK
UniRef90_Q8BEA7_1_208      GAV----KAENITE-----LLIRMKRAETYCPRPLL-ALD-TTHDRRKQE
UniRef90_C0LYR0_1_209      GAV----KAESITE-----LLIRMKRAETYCPRPLL-ALE-TTQDRRKQE
UniRef90_C0LS35_1_211      GAI----RATAISE-----LLVRMKRAELYCPRPLL-AVEVTEQDRHKQK
UniRef90_I0AZ16_1_210      GAI----KATKIHE-----LLVRMKRAELYCPRPLL-AIEVVSQDRHKQK
UniRef90_A4ZPP1_1_210      GAI----RATGIQE-----LLVRMKRAELYCPRPLL-AVEVSSQDRHKQR
UniRef90_A0ZXC4_1_211      GXI----KADXVHE-----LXVRMKRAELYCPRPLL-ALEVSSQDRHKQK
UniRef90_Q8JUS6_1_211      GAI----RATTIEE-----LLVRMKRAELYCPRPLL-AVEVTAQDRHKXX
UniRef90_A1EC12_1_211      GAV----QATTIHE-----LLVRMKRAELYCPRPLL-AVEVSSQDRHKQK
UniRef90_F6KYM9_1_211      GAI----KATQVTE-----LLYRMKRAETYCPRPLL-AIH-PSEARHKQK
UniRef90_B1A0X1_1_211      GAI----RATNIQE-----LLVRMKRAELYCPRPLL-AVEVSSQSRHKQK
UniRef90_I0AZ19_1_211      GAI----KADTIHE-----LLVRMKRAELYCPRPLL-AVEVSSQDRHKQK
UniRef90_Q912K6_1_211      GAI----KADNIHE-----LLVRMKRAELYCPRPLL-AIXLSSQNRHKQR
UniRef90_A4ZPL9_1_211      GAL----KATTIHE-----LLVRMKRAELYCPRPLL-AVEVSSQDRHKQK
UniRef90_Q9YPW9_1_211      GAI----KATRVTE-----LLYRMKRAETYCPRPLL-AIQ-PSDARHKQE
UniRef90_A1EC13_1_211      GAI----KNKSMHE-----LLVSMKRAELYCPRRLQ-TVEVSSQDRHKQK
UniRef90_B2MZQ3_1_211      GAI----KATRVTE-----LLYRMKRAETYCPRPLL-AIH-PTEARHRQK
UniRef90_E2J011_1_211      GAI----KTTLPTE-----LLYRRKRAETYCPRPLL-ATH-PSEARHKQR
UniRef90_I0AZ31_1_211      GAI----KADNIHE-----LLVRMKRAELYCPRPLL-AIEVSSQDRHKQK
UniRef90_D7RFC4_1_211      GAI----KATRVTE-----LLYRMKRAETYCPRPLL-AVH-PDQARHKQK
UniRef90_E2IZY3_1_211      GAI----KATRVTE-----LLYRMKRAETYCPRPLL-AIH-PCEARHKQK
UniRef90_Q9DJ33_1_211      GAI----KATRVIE-----LLYRMKRAETYCPRPLL-ATH-PSEARHKQR
UniRef90_Q9DJ21_1_192      GAI----KATRVTE-----LLYRMKRAE-YCXRPLL--------------
UniRef90_Q0PV54_36_239     GAI----RATSIQE-----LLVRMKRAELYCPRPLL-AVKVSSQDRHKTK
UniRef90_C8YS67_1_209      GAV----KAETITE-----LLVRMKRAELYCPRPIL-PVQ-PTGDRHKQP
UniRef90_F6KYY3_1_211      GAI----KATQVTE-----LLYRMKRAETYCPRPLL-AIH-PTEARHKQK
UniRef90_B1A0W4_1_211      GAI----RATTIHE-----LLVRMKRAELYCPRPLL-AVEVSSQDRHKQK
UniRef90_B3FHW7_1_211      GAI----KNKSMHE-----LLVSMKRAELYCPRTLQ-TVEVSSQDRHKQK
UniRef90_A0ZXB5_1_211      GAI----KATNIHE-----LLVRMKRAELYCPRPLL-AIEVSSQDRHKQK
UniRef90_A1EBZ3_1_211      GAI----KADHVHE-----LLVRMKRAELYCPRPLL-AIEVSSQDRHKQK
UniRef90_Q9DJA4_1_210      GAI----KATRVTE-----LLYRMKRAETYCPRPLL-AIH-PSEARHKQK
UniRef90_Q0QED4_407_613    GAV----KAETITE-----LLVRMKRAELYCPRPIL-PIQ-PTGDRRKQP
UniRef90_Q8JUU0_1_211      GAI----KATAIHE-----LLVRMKRAELYCPRPLL-AIEVSSQDRYKQK
UniRef90_Q9DJF0_19_211     GAI----KATRVTE-----LLYRMKRAETYCPRPLL-AVH-PSEARHKQK
UniRef90_I0AZ37_1_211      GAI----KADTIHE-----ILVRMKRAELYCPRPLL-AIEVSSQDRHKQK
UniRef90_Q67462_1_211      GAI----KATRVTE-----LLYRMKRAETYCPRPLL-AIH-PTDARHKRK
UniRef90_I0AZ36_1_211      GAI----KATTIHE-----ILVRMKRAELYCPRPLL-AIEVSSQDRHKQK
UniRef90_Q91EM4_1_211      GAI----KATRVTE-----LLYRMKRAETYCPRPLL-AIH-PSEXRHKQK
UniRef90_Q0PV56_33_229     GAI----RATTIHE-----LLVRMRRAELYCPRPLL-AMEVSAEGRHKQK
UniRef90_C0LYW2_1_209      GAV----QAETITE-----LLIRMKRAETYCPRPLL-ALD-TTQDRRKQE
UniRef90_B2MZX2_1_207      GAV----KAETITE-----LLVRMKRAELYCPRPIL-PVQ-STGDRHKQP
UniRef90_Q01124_1_209      GAV----KAETVXE-----LLVRMKRAELYCPRPIL-PIQ-PTGDRHKQP
UniRef90_E2J004_1_192      GAI----KGTRVTE-----LLYRMKRAETYCPRPLL--------------
UniRef90_Q8JUL9_1_209      GAI----KAQAIHE-----LLVRMKRAELYCPRPLL-AIEVSSQDRHKQK
UniRef90_Q6X5M0_1_179      GAL----KAQDIHE-----LLVRM--------------------------
UniRef90_Q2I5Y3_113_321    GAI----QATNISE-----LLVRMKRAELYCPRPLL-AVEVTTQDRHKQK
UniRef90_Q01121_1_189      GAV----KAETITE-----LLVRMKRAELYCPRPIL--------------
UniRef90_Q6X5M1_1_179      GAL----RADTIHE-----ILVRM--------------------------
UniRef90_A3RGT7_2_181      GAI----RATTIHE-----LLVRMRRAELYCPRPLL-AMEVSAEGRHKQK
UniRef90_P03310_11_218     GAV----KAETITE-----LLVRMKRAELYCPRPVL-PVQ-PTGDRHKQP
UniRef90_B3FHW2_1_211      GVI----QATRLTE-----LVYRMKRSENYCPRPLL-AIH-PNEARHKQK
UniRef90_Q8JUM1_1_211      GAI----RATTIEE-----LLVRMKRAELYCPRPLL-AVEVTAQDRHKQR
UniRef90_Q65051_1_200      GAI----RADTIHE-----LLVRMKRAELYCPRPLL-AIEVSSQDRHKQR
UniRef90_Q8JUL2_2_187      GAI----RATTIHE-----LLVRMKRAELYCPRPLL-AVEVSSQDRHKQK
UniRef90_B2BF63_11_200     GAI----KATRVTE-----LLYRMKRAETYCPRPLL-AIH-PNEARHKQK
UniRef90_Q0PV58_32_197     GAI-----------------------------------------------
UniRef90_Q65102_9_211      GAI----KASRVTE-----LLYRMKRAETYCPRPLL-AIH-PTDARHKQK
UniRef90_Q8BED3_1_210      GAV----KADTITE-----LLIRMKRAETYCPRPLL-ALD-TTQDRRKQE
```

```
UniRef90_A0PG53_1_160     GAI----KAAQVTE-----LLYRMKRAETYCPRPLL-AIH-PSEARHKQK
UniRef90_B3FHW0_1_211     GAI----QATRVTE-----LLYRMKRAETYCPRPLL-VIH-PNEARHRPQ
UniRef90_B3FHV9_1_211     GAI----QATRVTE-----MVYRMKRSETYCPRPLL-AIH-PNEARHKQK
UniRef90_B3FHV8_1_211     GVI----QATRLSQ-----MVYRMKRSESYCPRRLL-AIH-PNEARHKQK
UniRef90_A0PG60_5_160     GAI----KATRVTE-----LLYRMKRAETYCPRPLL-AIH-PSEARHKQK
UniRef90_Q91NQ7_2_163     GAI----KATRVTE-----LLYRMKRAETYCPRPLL-AVH-PSETRHKQK
UniRef90_B3FHV6_1_211     GVI----QATRLTE-----LVYRMKRSENYCPRPLL-AIH-PNEARHKQK
UniRef90_B3FHV7_1_211     GVI----QATRVTE-----LVYRMKRSENYCPRPLL-AIH-PNEARHKQK
UniRef90_A0PG54_1_160     GAI----KATRVTE-----LLYRMKRAETYCPRPLL-AVH-PTEARHKQK
UniRef90_B3FHW1_1_211     GAI----QATRVTE-----LLYRMKRVETYCARHLL-AIH-PNEARHKQK
UniRef90_Q65068_1_206     GAV----KAETIXE-----LLVRMKRAELYCPRPIL-PIQ-PTGXRHKQX
UniRef90_Q9YPF0_1_208     GAV----XAETVTE-----LLVRMKRAELYCPRPIL-PIQ-PTGDRHKQP
UniRef90_Q8BEF3_1_209     GAV----KAETITE-----LLIRMKRAETYCPRPLL-ALD-TTQDRRXQK
UniRef90_B3FHV4_1_211     GVI----QATRLTE-----LVYRMKRSENYCPRPLL-AIH-PNEARHKQK
UniRef90_Q8JUN9_1_211     GAI----RATTIEE-----LLVRMKRAELYCPRPLL-AVEVTAQDRHKQR
UniRef90_Q9YPG1_1_165     GAI-----------------------------------------------
UniRef90_A0PG23_5_162     GAI----KATRVSE-----LLYRMKRAETYCPRPLL-AIH-PSETRHKQK
UniRef90_Q9DJ61_1_156     GAI----KATRVTE-----LLYRMKRAXTYCPRPLL-AIH-PSEARXKQK
UniRef90_Q65097_1_206     XAV----KAETIXE-----LLVRXKRAXLYCPRPIL-PIX-PTDXRHXQP
UniRef90_Q01126_1_208     GAV----KADTVTE-----LLVRMKRAELYCPRPIL-PIQ-PXGDRHKQL
UniRef90_Q67461_13_173    XXX----XXTRVTE-----LLYRMKRAETYCPRPLL-AIH-PTDARHKQK
UniRef90_Q00280_54_209    GAV----KAETITE-----LLVRMKRAELYCPRPVL-PVQ-PAGDRHKQP
UniRef90_H9AY35_719_926   GLVFVVGESGVSFR-----ILYRLKRTELYC
```

```
2WZR1                     LVKPAKQ
UniRef90_Q6PMU0_725_943   ITKPVKQ
UniRef90_F1AW55_725_943   LVAPAKQ
UniRef90_A0PG93_1_219     LVKPAKQ
UniRef90_A0PG86_1_219     LTKPAKQ
UniRef90_A0PG77_1_219     LTKPAKQ
UniRef90_Q6PMU2_725_943   LVAPEKQ
UniRef90_Q8V0G8_526_744   LVAPAKQ
UniRef90_A0PG80_1_219     LVAPEKQ
UniRef90_Q8BDT2_1_218     LVAPEKQ
UniRef90_A0PG94_1_219     LVAPAKQ
UniRef90_C8YS70_1_219     LVAPEKQ
UniRef90_Q6PMT8_726_934   I------
UniRef90_Q6PMT3_724_939   LVKPDKQ
UniRef90_B8Y0J7_726_934   I------
UniRef90_E2IU95_1_209     I------
UniRef90_Q80JY0_1_209     I------
UniRef90_A0PFY2_1_209     I------
UniRef90_Q80K03_1_209     I------
UniRef90_Q1L757_525_734   I------
UniRef90_Q80K16_1_209     I------
UniRef90_Q1L752_527_735   I------
UniRef90_A0PG17_1_209     I------
UniRef90_Q80JY2_1_209     I------
UniRef90_Q80JY8_1_209     I------
UniRef90_Q8JVD0_525_739   LVAPDKQ
UniRef90_Q80JU2_1_209     I------
UniRef90_A0PFY7_1_209     I------
UniRef90_A0PFY4_1_209     I------
UniRef90_Q80JZ5_1_205     -------
UniRef90_A0PFZ0_1_209     I------
UniRef90_Q1L748_525_740   LTKPEKQ
UniRef90_Q80JY7_1_209     I------
UniRef90_A0PG12_1_209     I------
UniRef90_A0PFY8_1_209     I------
UniRef90_Q80JZ1_1_209     I------
UniRef90_Q80JU1_1_209     I------
UniRef90_B2MZR9_1_211     IIAPAKQ
UniRef90_P49303_726_936   IIAPAKQ
UniRef90_Q2I5Z0_71_281    IVAPVKQ
UniRef90_Q9DJ49_1_212     IVAPTKQ
UniRef90_A1EBZ1_1_211     IIAPAKQ
UniRef90_A1EBZ4_1_211     IIAPAKQ
UniRef90_I0AZ32_1_211     IIAPAKQ
UniRef90_A8D5K4_1_211     IVAPVKQ
UniRef90_Q2I5X3_80_290    IIAPAKQ
```

UniRef90_I0AYZ5_1_211	I	I	A	P	A	K	Q
UniRef90_I0AZ39_1_210	I	I	A	P	A	K	Q
UniRef90_H9T7U2_127_337	I	V	A	P	V	K	Q
UniRef90_Q9DJF9_1_211	I	V	A	P	E	K	Q
UniRef90_I0AYY0_1_211	I	I	A	P	A	K	Q
UniRef90_Q2I5X1_23_232	I	I	A	P	A	K	Q
UniRef90_B2ZHQ6_1_211	I	V	A	P	V	K	Q
UniRef90_A4ZPM0_1_211	I	I	A	P	A	K	Q
UniRef90_E7D638_1_210	I	I	A	P	A	K	Q
UniRef90_Q8JUM5_1_211	I	I	A	P	A	K	Q
UniRef90_Q8BED5_1_209	I	I	A	P	E	K	Q
UniRef90_A0ZXF2_1_211	I	V	A	P	V	K	Q
UniRef90_F1BVI2_1_211	I	I	A	P	A	K	Q
UniRef90_I0AYX9_1_210	I	I	A	P	A	K	Q
UniRef90_I0AZ13_1_211	I	I	A	P	A	K	Q
UniRef90_A4ZPL8_1_211	I	I	A	P	A	K	Q
UniRef90_Q6PMY9_724_932	I	I	A	P	E	K	Q
UniRef90_Q8JUP6_1_211	I	I	A	P	A	K	Q
UniRef90_B1A0W6_1_210	I	I	A	P	A	K	-
UniRef90_C0LRW1_1_211	I	I	A	P	A	K	Q
UniRef90_Q8JUN6_1_211	I	I	A	P	A	K	Q
UniRef90_A1EC01_1_211	I	I	A	P	A	K	Q
UniRef90_A2I8Z5_725_935	I	V	A	P	A	K	Q
UniRef90_B1A0W7_1_211	I	I	A	P	A	K	Q
UniRef90_Q9DJ38_1_212	I	V	A	P	V	K	Q
UniRef90_A1EC10_1_211	I	I	A	P	A	K	Q
UniRef90_E2FZV2_1_211	I	V	A	P	V	K	Q
UniRef90_I0AYY6_1_211	I	I	A	P	A	K	Q
UniRef90_A7XPU3_1_209	I	I	A	P	E	K	Q
UniRef90_Q999Y2_1_211	I	I	A	P	A	K	Q
UniRef90_B2MZR8_1_211	X	I	A	P	A	K	Q
UniRef90_A1EC03_1_211	I	I	A	P	A	K	Q
UniRef90_Q8BEA7_1_208	I	I	A	P	E	K	Q
UniRef90_C0LYR0_1_209	I	I	A	P	E	K	Q
UniRef90_C0LS35_1_211	I	I	A	P	A	K	Q
UniRef90_I0AZ16_1_210	I	I	A	P	A	K	Q
UniRef90_A4ZPP1_1_210	I	I	A	P	A	K	Q
UniRef90_A0ZXC4_1_211	I	I	A	P	E	K	Q
UniRef90_Q8JUS6_1_211	I	I	A	P	A	K	Q
UniRef90_A1EC12_1_211	I	M	A	P	E	K	Q
UniRef90_F6KYM9_1_211	I	V	A	P	V	K	Q
UniRef90_B1A0X1_1_211	I	I	A	P	A	K	Q
UniRef90_I0AZ19_1_211	I	I	A	P	T	K	Q
UniRef90_Q912K6_1_211	I	I	A	P	A	K	Q
UniRef90_A4ZPL9_1_211	I	I	A	P	A	R	Q
UniRef90_Q9YPW9_1_211	I	V	A	S	A	K	Q
UniRef90_A1EC13_1_211	I	N	A	P	A	K	Q
UniRef90_B2MZQ3_1_211	I	V	A	P	V	K	Q
UniRef90_E2J011_1_211	I	V	A	S	A	K	Q
UniRef90_I0AZ31_1_211	I	I	A	P	A	K	Q
UniRef90_D7RFC4_1_211	I	V	A	P	V	K	Q
UniRef90_E2IZY3_1_211	I	V	A	P	V	K	Q
UniRef90_Q9DJ33_1_211	I	I	A	P	A	K	Q
UniRef90_Q9DJ21_1_192	-	-	-	-	-	-	-
UniRef90_Q0PV54_36_239	-	-	-	-	-	-	-
UniRef90_C8YS67_1_209	L	I	A	P	A	K	Q
UniRef90_F6KYY3_1_211	I	V	A	P	L	K	Q
UniRef90_B1A0W4_1_211	I	I	A	P	A	K	Q
UniRef90_B3FHW7_1_211	I	N	A	P	A	K	Q
UniRef90_A0ZXB5_1_211	I	I	A	P	A	K	Q
UniRef90_A1EBZ3_1_211	I	I	A	P	A	K	Q
UniRef90_Q9DJA4_1_210	I	V	X	P	X	K	-
UniRef90_Q0QED4_407_613	L	V	A	P	A	K	Q
UniRef90_Q8JUU0_1_211	I	I	A	P	A	K	Q
UniRef90_Q9DJF0_19_211	I	V	A	P	V	K	Q
UniRef90_I0AZ37_1_211	I	I	A	P	A	K	Q
UniRef90_Q67462_1_211	I	V	A	P	V	K	R
UniRef90_I0AZ36_1_211	I	I	A	P	A	K	Q
UniRef90_Q91EM4_1_211	V	V	A	P	V	K	Q
UniRef90_Q0PV56_33_229	I	I	A	P	A	K	Q
UniRef90_C0LYW2_1_209	I	I	A	P	E	R	Q
UniRef90_B2MZX2_1_207	L	I	A	P	A	K	Q
UniRef90_Q01124_1_209	L	I	A	P	A	K	Q

UniRef90_E2J004_1_192	-	-	-	-	-	-	-
UniRef90_Q8JUL9_1_209	I	I	A	P	A	K	Q
UniRef90_Q6X5M0_1_179	-	-	-	-	-	-	-
UniRef90_Q2I5Y3_113_321	I	I	A	P	A	K	Q
UniRef90_Q01121_1_189	-	-	-	-	-	-	-
UniRef90_Q6X5M1_1_179	-	-	-	-	-	-	-
UniRef90_A3RGT7_2_181	I	I	A	P	A	K	Q
UniRef90_P03310_11_218	L	I	A	P	A	K	Q
UniRef90_B3FHW2_1_211	I	V	A	P	A	K	Q
UniRef90_Q8JUM1_1_211	I	I	A	P	A	K	Q
UniRef90_Q65051_1_200	I	I	A	P	A	K	Q
UniRef90_Q8JUL2_2_187	I	I	A	P	A	K	Q
UniRef90_B2BF63_11_200	I	V	A	P	V	K	Q
UniRef90_Q0PV58_32_197	-	-	-	-	-	-	-
UniRef90_Q65102_9_211	I	V	A	A	V	K	Q
UniRef90_Q8BED3_1_210	I	I	A	P	E	K	Q
UniRef90_A0PG53_1_160	I	V	A	P	-	-	-
UniRef90_B3FHW0_1_211	I	V	P	T	V	K	Q
UniRef90_B3FHV9_1_211	I	V	A	P	A	K	Q
UniRef90_B3FHV8_1_211	I	V	A	P	A	K	Q
UniRef90_A0PG60_5_160	I	V	A	P	-	-	-
UniRef90_Q91NQ7_2_163	I	V	A	P	M	K	Q
UniRef90_B3FHV6_1_211	I	V	A	P	A	K	Q
UniRef90_B3FHV7_1_211	I	V	A	P	E	K	Q
UniRef90_A0PG54_1_160	I	V	A	P	-	-	-
UniRef90_B3FHW1_1_211	V	V	V	T	V	K	Q
UniRef90_Q65068_1_206	L	V	A	P	A	K	-
UniRef90_Q9YPF0_1_208	L	I	A	P	A	K	-
UniRef90_Q8BEF3_1_209	I	X	T	P	E	X	Q
UniRef90_B3FHV4_1_211	I	V	A	P	A	K	Q
UniRef90_Q8JUN9_1_211	I	I	A	P	A	K	Q
UniRef90_Q9YPG1_1_165	-	-	-	-	-	-	-
UniRef90_A0PG23_5_162	I	E	A	P	V	K	-
UniRef90_Q9DJ61_1_156	I	V	A	P	V	K	Q
UniRef90_Q65097_1_206	L	V	A	P	A	K	-
UniRef90_Q01126_1_208	X	V	A	P	E	X	Q
UniRef90_Q67461_13_173	I	V	A	P	V	K	Q
UniRef90_Q00280_54_209	X	I	A	P	A	K	Q
UniRef90_H9AY35_719_926							

1	2	3	4	5	6	7	8	9
Variable			Average			Conserved		

X - Insufficient data - the calculation for this site was performed on less than 10% of the sequences.

Figure 2S

```
2WZR2                        DKKTEETTLLEDRIVTTSHGTTTSTTQSSVGVTYGYALTDKFLPGPN-T-
UniRef90_F1AW55_285_503      DKKTEETTLLEDRILTTSHGTTTSTTQSSVGITYGYASSDKFVPGPN-T-
UniRef90_Q6PMU0_285_503      DKKTEETTLLEDRILTTSHGTTTSTTQSSVGVTRGYADSDKFLPGPN-T-
UniRef90_Q8V0G8_86_304       DKKTEETTLLEDRILTTSHGTTTSTTQSSVGVTYGYAASDQFLPGPN-T-
UniRef90_Q6PMU2_285_503      DKKTEETTLLEDRILTTSHGTTTSTTQSSVGVTYGYAASDKFLPGPN-T-
UniRef90_Q6PMT8_285_503      DKKTEETTLLEDRIVTTRHGTTTSTTQSSVGITYGYADADSFRPGPN-T-
UniRef90_A2I8Z5_287_504      DKKTEETTLLEDRILTTRNGHTTSTTQSSVGVTYGYATAEDLVSGPN-T-
UniRef90_Q1L754_86_304       DKKTEETTLLEDRIVTTRHGTTTSTTQSSVGVTYGYADSDSFHPGPN-T-
UniRef90_B8Y0J7_285_503      DKKTEETTLLEDRILTTRHGTTTSTTQSSVGVTLGYADADAFRPGPN-T-
UniRef90_Q1L757_86_304       DKKTEETTLLEDRILTTRHGTTTTTTQSSVGVTYGYADSDRFVPGPN-T-
UniRef90_P49303_287_504      DKKTEETTLLEDRILTTRNGHTTSTTQSSVGVTYGYSTQEDHVSGPN-T-
UniRef90_A6XP89_287_504      DKKTEETTLLEDRILTTRNGHTTSTTQSSVGVTYGYAVAEDAVSGPN-T-
UniRef90_Q6PMT3_285_502      DKKTEETTHLEDRILTTRHNTTTSTTQSSVGVTYGYASADRFLPGPN-T-
UniRef90_Q1L752_86_304       DKKTEETTLLEDRILTTRHGTTTSTTQSSVGVTFGYADADSFTAGPN-T-
UniRef90_Q1L748_86_303       DKKTEETTLLEDRILTTRHGTTTSTTQSSVGVTYGYASADKFLPGPN-T-
UniRef90_P15072_287_504      DKKTEETTLLEDRILTTRNGQTTSTTQSSVGVTFGYATAEDSTSGPN-T-
UniRef90_Q8JVD0_86_303       DKRTEETTLLEDRILTTRHNTTTFTTQSSVGVTHGYASADRFLPGPN-T-
UniRef90_H9AY35_273_496      DHKTEETTKLEDRILVTRSGPTTKTTQSSVGVVQGYGG----PSPQSTK
UniRef90_B0Z3W9_300_527      DKKTEETTKLEDRIMTTRHHTDITTTQSTVGITHGYAHSE--LDQVNAA-
UniRef90_Q91LX8_289_537      DQDTEETTRQPDRIVTTLDGNTSRTTQSSVGILRGYNYTPGKHVNP-SS-
UniRef90_Q66776_291_513      DQDTEDVTRQPDRIVTVLDGNTSRTTQSSVGILRGYNYAPGKHTQP-SS-
UniRef90_Q100Y4_72_320       DQETEDVTRQPDRIVTVLDGNTSRTTQSSVGILRGYNYKPGKHVPP-SS-
UniRef90_Q2XP17_290_516      DKKTEETTNIEDRIETTVVGVTIINSQGSVGTTYCYSRPDSKAP---ST-
UniRef90_P12296_138_384      DQNTEEMENLSDRVSQDTAGNTVTNTQSTVGRLVGYGTV-HDGEHP-AS-
UniRef90_Q02472_148_405      DENTEEMENLSDRVDSDKAGNSATNTQSSVGRLHGYGAT-HRGDHP-AS-
UniRef90_A4ZKN2_144_403      DQNTEEMTNLSDRVSSDTAGNTATNTQSTVGRLFAFGSR-HKGKHP-AS-
UniRef90_C3U5A3_174_435      DQDTEEMTNLSDRVASDTAGNTATNTQSTVGRLFGYASR-HRGKHP-AS-
UniRef90_P08544_148_405      DQNTEEMENLSDRVASDKAGNSATNTQSTVGRLCGYGKS-HHGEHP-AS-
UniRef90_E3TMG9_144_405      DQNTEEMTNLSDRVSSDTAGNTATNTQSTVGRLFGFGQR-HKGKHP-AS-
UniRef90_G9FTJ7_144_402      DQDTEEMTNLSDRVSSDTAGNTATNTQSTVGRLFAFGSR-HKGKHP-AS-
UniRef90_B3VTU7_149_406      DQNTEEMENLSDRVDSDKAGNSAVNTQSSVGRLCGYGMH-HKGKHP-AS-
UniRef90_Q155Z9_151_421      DHNTEEMENSADRVTTQTAGNTAINTQSSLGVLCAYVEDPTKSDPP-SS-
UniRef90_G1C9J1_14_204       -----------DRIMQLTMGNSTITTQEAANAVVAYGVWPDFAPGL-GE-
UniRef90_I1YLT5_69_326       DPKTENTTNMEDRILTRRHGNTAISSQAAEGVLYGYGMESDKSDPTSCG-
UniRef90_G0Y2E2_141_365      ---TVEEAGFSDRIMQITSGNSTITTQEAANAIVGYGVWPEIDQGV-GQ-
UniRef90_G0Y2E0_150_361      -----------DRIMQLTAGNSTITTQEAASAVVAYGVWPTHDVGV-GE-
UniRef90_Q8B552_195_378      -----------------------------------------TYNIDAGE-
UniRef90_B5A1N0_195_378      -----------------------------------------SYNIDAGE-
UniRef90_Q2LKZ0_129_301      --------------------------------------------------
UniRef90_Q65481_129_301      --------------------------------------------------
UniRef90_G1DFA4_229_430      DPDTEESTELPDRITEDTAGNTNITTQSSVGTLVAYHQL--RSKHPVTS-
UniRef90_H9LG14_129_301      --------------------------------------------------
UniRef90_H6VLP2_125_335      -----------DRIMQITSGNSTITTQEAVQAIVAYGEWPEYEVGI-GE-
UniRef90_B9VIN1_70_329       -PSAENSTNMGDRILTLREGNTAISSQATEGILHGYNKETDTSQPTSCG-
UniRef90_G1JYY8_171_367      -----------DRILSITTGNSTITTQEAASAIVGYATWPEYYAGA-GE-
UniRef90_Q8QUZ6_80_281       -----------DRVAQLTMGNSTITTQEAANITVAYGEWPSYLSDLDAT-
UniRef90_I1YLT7_70_335       -PQREKQNIQEDRIFTRNAGNTSVNSQASEGVLQAYGKESDNTDPTSCG-
UniRef90_F5B9S5_80_301       -----------DRVAQLTLGNSTITTQEAANITVAYGEWPGYLSDFDAT-
UniRef90_G0Y2E4_163_340      -------------------------------------------VGV-GH-
UniRef90_B8XTQ1_73_321       ----EGATYLEDRLLTRRAGNTAVNSQAAEGVLLAYGKESDKTCPTSCG-
UniRef90_H9C6N6_131_322      --------------------------------------------------
UniRef90_I1YLT4_70_334       -PRVETTTNQEDRILTRNAGNTSINSQASEGVLHAYGRESDDTDPTSCG-
UniRef90_B8XTQ0_73_332       ----ESSRDAEDRVITRQAGNTTVNSQASEGVLCAYGVDSLGTWPTSCG-
UniRef90_B9V442_80_329       -----------DRIIQITRGDSTITSQDVANAVVGYGVWPHYLTPQDAT-
UniRef90_D2IW00_80_316       -----------DRIIQITRGNSTITSQDVANAVVGYGVWPQYLPDDDAS-
UniRef90_Q2LL04_129_301      --------------------------------------------------
UniRef90_I1YLT6_69_335       NPRTENSTYLEDRVLTRTAGNTSINSQAAEGVLNAYAKESDQTCPTSCG-
UniRef90_G5DC73_80_330       -----------DRIIQITRGDSTITSQDVANAVVAYGVWPHYLSSGDAT-
UniRef90_A5GZE4_80_318       -----------DRIIQITRGDSTITSQDVANAVVGYGVWPHYLTSRDAT-
UniRef90_D9D8F3_70_328       -PRVENSTYQEDRILSRVAGNTSINSQASEGVLCAYAHETDGSDPTSCG-
UniRef90_G0ZNT5_127_322      --------------------------------------------------
UniRef90_B9V459_80_317       -----------DRIIQITRGDSTMTSQDVANAVVGYGVWPHYLTAQDAT-
UniRef90_A8S322_129_323      --------------------------------------------------
UniRef90_C7DUC7_127_322      --------------------------------------------------
UniRef90_G8IJE2_80_317       -----------DRIIQITRGDSTITSQDVANAVVAYGVWPHYLSSKDAT-
UniRef90_Q2LKZ8_129_301      --------------------------------------------------
UniRef90_B8XTP8_70_327       -PRTETGMMMEDRVLSRTAGNTSVNSQAAEGVLQAYGTETDSNSPTSCG-
UniRef90_H2E685_80_301       -----------DRVAQLTLGNSTITTQEAANITVAYGEWPSYLSDKDAT-
UniRef90_Q5YJ95_199_371      --------------------------------------------------
```

```
UniRef90_Q9IM09_80_305     -----------DRVAQLTIGNSTITTQEAANVVVAYGQWPEYLDSKDAT-
UniRef90_A5GZE9_80_317     -----------DRIIQITRGDSTITSQDVANAVVGYGVWPHYLTPQDAT-
UniRef90_Q66790_80_316     -----------DRVRSITLGNSTITTQESANVVVAYGRWPKYLEDDQAT-
UniRef90_A5GZD8_131_315    --------------------------------------------------
UniRef90_P12915_129_301    --------------------------------------------------
UniRef90_Q5XLP5_80_317     -----------DRIIQITRGDSTITSQDVANAVVGYGVWPHYLTADDAS-
UniRef90_G0Z2U8_195_360    --------------------------------------------------
UniRef90_A1E4A3_131_303    --------------------------------------------------
UniRef90_G0Y2E3_141_369    ---TVEELGYSDRIMQITSGNSTITTQEAVNAVVAYGVWPSIDEGV-GQ-
UniRef90_B9V482_80_298     -----------DRVQQITLGNSTITTQEAANAVVCYAEWPEYLPDKDAS-
UniRef90_B8XTP9_157_328    --------------------------------------------------
UniRef90_F8V2V4_80_316     -----------DRVRSITLGNSTITTQECANVVVGYGVWPDYLSDEEAT-
UniRef90_A1E4A2_131_303    --------------------------------------------------
UniRef90_Q8V5Z9_80_307     -----------DRVAQLKLGNTSITTQEAANITVGYGEWPTFLSNVDAN-
UniRef90_G8IJE1_80_316     -----------DRIIQITRGDSTITSQDVANAVVGYGVWPHYLSPEDAT-
UniRef90_P12916_80_317     -----------DRIIQITRGDSTITSQDVANAVVGYGVWPHYLTPQDAT-
UniRef90_P03303_80_315     -----------DRVQQITLGNSTITTQEAANAVVCYAEWPEYLPDVDAS-
UniRef90_B8XTQ4_69_329     KPKVENDTNMEDRVITLKAGNTIVNSQASEGVLHGYGIGTNTQRPSSCG-
UniRef90_Q9YLJ1_78_316     ---------FSDRVRSLTLGNSTITTQESANVVVGYGRWPDYLADDQAT-
UniRef90_B9V466_80_318     -----------DRIIQITRGDSTITSQDVANAVIGYGIWPHYLSAEDAT-
UniRef90_Q05ED6_131_323    --------------------------------------------------
UniRef90_B5BSU7_131_316    --------------------------------------------------
UniRef90_A5GZF1_80_316     -----------DRIIQITRGDSTITSQDVANAVVGYGVWPHYLTSQDAT-
UniRef90_B9V453_80_313     -----------DRIIQITRGDTTITSQDIANAVVGYGVWPTYLDSKDAS-
UniRef90_Q05EE3_80_323     -----------DRVRSITLGNSTITTQESANVVVSYGEWPRYLKDDEAT-
UniRef90_A5GZE7_80_317     -----------DRIIQITRGDSTITSQDVANAVVGYGVWPHYLSATDAT-
UniRef90_B9V485_80_315     -----------DRVQQITLGNSTITTQEAANAVVCYAEWPEYLPDKDAS-
UniRef90_G8IJH5_80_318     -----------DRIIQITRGDSTITSQDVANAVVGYGVWPHYLTAQDAT-
UniRef90_B9V435_80_318     -----------DRIIQITRGDSTITSQDVANAVVGYGVWPHYLTPQDAT-
UniRef90_Q8V398_80_292     -----------DRVLQLTLGNSTITTQEAANIVVGYGKWPEHLKPIDAN-
UniRef90_Q82081_159_315    --------------------------------------------------
UniRef90_Q6W9F6_159_316    --------------------------------------------------
UniRef90_Q03053_159_316    --------------------------------------------------
UniRef90_A5GZD6_131_315    --------------------------------------------------
UniRef90_Q2YEN8_80_316     -----------DRVRSLTLGNSTITTQESANVVVGYGRWPEYLKDEEAT-
UniRef90_G8HH17_117_311    ---TVEECGFSDRIMQITSGNSTITTQEAVNAVVAYGCWPSFDSGA-GE-
UniRef90_A7KC08_80_331     -----------DRIMQITRGDSTITSQDVANAVVAYGVWPHYLTPQDAT-
UniRef90_Q6W9G1_131_314    --------------------------------------------------
UniRef90_G8IQ64_80_315     -----------DRVQQITLGNSTITTQEAANAVVCYAEWPEYLPDNDAS-
UniRef90_I0IY47_80_323     -----------DRVLQLTLGNSTITTQEAANAVVGYGEWPTLLNSNEAN-
UniRef90_G5DC75_80_317     -----------DRIIQITRGDSTITSQDVANAVVGYGVWPHYLSGTDAT-
UniRef90_Q05ED2_80_316     -----------DRVRSITLGNSTITTQESANVVVAYGKWPDYLKDEEAT-
UniRef90_Q5VAK8_80_316     -----------DRVRSITLGNSTITTQECANVVVGYGVWPSYLKDNEAV-
UniRef90_A7KC11_80_330     -----------DRIIQITRGDSTITSQDVANAVVGYGVWPHYLTPQDAT-
UniRef90_B5BSU6_131_316    --------------------------------------------------
UniRef90_Q05ED0_80_316     -----------DRVRSLTLGNSTITTQESANVVIGYGVWPNYLSDDQAT-
UniRef90_Q5VAL5_159_315    --------------------------------------------------
UniRef90_A4UHT9_129_315    --------------------------------------------------
UniRef90_H8Y6P9_127_322    --------------------------------------------------
UniRef90_P29813_80_316     -----------DRVRSITLGNSTITTQESANVVVGYGRWPEYLKDNEAT-
UniRef90_P32537_131_305    --------------------------------------------------
UniRef90_G5D5E2_159_315    --------------------------------------------------
UniRef90_Q9YLG5_80_319     -----------DRVRSITLGNSTITTQECANVVVGYGTWPRYLSDKEAT-
UniRef90_B8XTQ3_70_330     -PKVENSLYTEDRLLTRKAGNTSVNSQAAEGVLQGYGHESDFSNPTSCG-
UniRef90_A7KC13_88_321     -------------------GNSTITTQEAANAIVAYAEWPSFLKDDDAS-
UniRef90_O41174_80_282     -----------DRVAQLTLGNSTITTQEAANITVAYGEWPSYLSDLDAT-
UniRef90_A5GZF9_80_318     -----------DRIIQITRGDSTITSQDVANAVVAYGVWPHYLTPQDAT-
UniRef90_G8IJE5_80_295     -----------DRIIQITRGDSTITSQDVANAVVGYGVWPHYLTPQDAT-
UniRef90_Q9QCF0_72_316     ---TAEECGYSDRVRSITLGNSTITTQECANVVVGYGTWPDYLHDDEAT-
UniRef90_G5DC71_157_324    --------------------------------------------------
UniRef90_A8S330_157_325    --------------------------------------------------
UniRef90_Q9WN78_80_316     -----------DRVRSITLGNSTITTQECANVVVGYGVWPTYLSDHEAT-
UniRef90_A0MHB7_86_316     -------------------GSSTITTQDSVNTIVAYGEWPSYLSDLDAS-
UniRef90_O89280_159_323    --------------------------------------------------
UniRef90_G8IJG2_80_318     -----------DRIIQITRGNSTITSQDIANAVVAYGVWPEYLPSQDAS-
UniRef90_F6M9Y3_80_316     -----------DRIIQITRGNSTITSQDIANAVVGYGVWPQYLSDADAN-
UniRef90_A5GZE0_80_317     -----------DRIIQITRGDSTITSQDVANAVVGYGVWPHYLTSQDAT-
UniRef90_Q7T7N9_80_323     -----------DRVLQLTLGNSTITTQEAANAVVAYGEWPRFINDQEAN-
UniRef90_A7YF31_159_315    --------------------------------------------------
UniRef90_H9X8D4_80_317     -----------DRIIQITRGDSTITSQDVANAVVGYGVWPHYLTPQDAT-
UniRef90_G9K146_80_316     -----------DRIIQITRGDSTITSQDVANAVVGYGVWPHYLTPQDAT-
UniRef90_G9K138_159_330    --------------------------------------------------
UniRef90_G0YPI0_80_292     -----------DRVAQLTVGNSTITTQEAANIVVGYGEWPNYCPDTDAT-
```

```
UniRef90_O91734_159_316   --------------------------------------------------
UniRef90_B9V473_80_315    -----------DRIIQITRGNSTITSQDIANAVVAYGVWPQYLSDADAN-
UniRef90_Q9WHH4_131_316   --------------------------------------------------
UniRef90_Q2L970_80_316    -----------DRVRSITLGNSTITTQESANVVVGYGVWPDYLSDEEAT-
UniRef90_Q80GI2_159_316   --------------------------------------------------
UniRef90_Q05ED1_131_316   --------------------------------------------------
UniRef90_B9V440_80_317    -----------DRIIQITRGDSTITSQXVANAVVGYGVWPHYLTPEDAT-
UniRef90_G1CSA8_80_313    -----------DRVAQLTVGNSTITTQEAANITVGYGEWPEYCPDTDAT-
UniRef90_B1PZZ4_80_316    -----------DRVRSITLGNSTITTQESANVVVAYGRWPEYLKDDEAT-
UniRef90_B9V457_80_316    -----------DRIIQITRGNSTITSQDIANAVVAYGVWPHYLSDTDAN-
UniRef90_D8VCT6_80_323    -----------DRVLQLTLGNSTITTQEAANAVIGYGEWPQFLNSADAN-
UniRef90_G8IRN4_129_324   --------------------------------------------------
UniRef90_Q80GI1_159_316   --------------------------------------------------
UniRef90_I1XV41_80_323    -----------DRVMQLTLGNSTITTQEAANAVVAYGEWPSFLNPQEAN-
UniRef90_P21404_159_316   --------------------------------------------------
UniRef90_P23008_135_293   --------------------------------------------------
UniRef90_A5GZE2_80_315    -----------DRIIQITRGDSTITSQDVANAVVGYGVWPHYLTPEDAT-
UniRef90_Q6W9F4_159_316   --------------------------------------------------
UniRef90_B3SRP0_80_317    -----------DRVAQLTVGNSTITTQEAANITVGYGVWPEYSKDTEAT-
UniRef90_Q6JKS0_159_312   --------------------------------------------------
UniRef90_G8IJG6_157_325   --------------------------------------------------
UniRef90_D4AH62_131_316   --------------------------------------------------
UniRef90_A5GZD9_129_321   --------------------------------------------------
UniRef90_Q66331_80_318    -----------DRVRSITLGNSTITTQECANVVVGYGVWPDYLKDSEAT-
UniRef90_Q6W9E8_159_323   --------------------------------------------------
UniRef90_P04936_159_330   --------------------------------------------------
UniRef90_A7KC07_80_326    -----------DRIIQITRGDSTITSQDIANAVVAYGVWPHYLSAEDAN-
UniRef90_Q66806_80_316    -----------DRVRSITLGNSTITTQESANVVVGYGVWPEYLRDEEAT-
UniRef90_A5GZF5_80_318    -----------DRIIQITRGNSTITSQDIANAVVGYGVWPEYLTDDDAS-
UniRef90_Q05ED4_159_315   --------------------------------------------------
UniRef90_G8IRN3_129_329   --------------------------------------------------
UniRef90_Q82122_80_315    -----------DRIIQITRGDSTITSQDVANAVVGYGVWPHYLTPQDAT-
UniRef90_Q8V601_80_317    -----------DRVAQLTVGNSTITTQEAANITVGYGVWPEYSKDTEAT-
UniRef90_B9V433_129_314   --------------------------------------------------
UniRef90_G5EN04_80_318    -----------DRVAQLTVGNSTITTQEAANIVLSYGEWPEYCPSTDAT-
UniRef90_Q8V613_159_316   --------------------------------------------------
UniRef90_Q6W9F9_80_316    -----------DRVRSMTLGNSTITTQESANVVVAYGEWPHYLSDDEAT-
UniRef90_H9C6N7_159_315   --------------------------------------------------
UniRef90_B9V434_80_314    -----------DRIIQITRGDTTITSQDIANAVVAYGVWPTYLNSKDAT-
UniRef90_A5GZF6_80_318    -----------DRIIQITRGNSTITSQDVANAVVAYGVWPEYLSAQDAS-
UniRef90_A5GZF2_80_318    -----------DRLMQITRGDSTITSQDIANAVVGYGVWPSYLTPDDAT-
UniRef90_C7DUC8_80_317    -----------DRIMQITRGDSTITSQDVANAVVGYGVWPHYLSAKDAT-
UniRef90_G5CZJ4_127_322   --------------------------------------------------
UniRef90_B9V477_80_318    -----------DRIIQITRGNSTITSQDIANAVVAYGVWPEYLSSDDAS-
UniRef90_G8IJH3_88_314    -------------------GNSTITTQEAANAIVGYGEWPSFLDDKDAS-
UniRef90_Q8UY33_159_317   --------------------------------------------------
UniRef90_P07210_80_318    -----------DRLIQITRGDSTITSQDTANAVVAYGVWPSYLTPDDAT-
UniRef90_Q66577_131_323   --------------------------------------------------
UniRef90_G8IJF0_127_318   --------------------------------------------------
UniRef90_E7CRL8_159_323   --------------------------------------------------
UniRef90_Q995Z1_80_314    -----------DRVRSITLGNSTITTQECANVVVGYGVWPTYLSDTEAT-
UniRef90_C6ZEZ9_129_315   --------------------------------------------------
UniRef90_Q5G9Z5_159_312   --------------------------------------------------

2WZR2                     ---NGLETRVEQAERFFKHKLFDW--------------TLDQQFGT--TY
UniRef90_F1AW55_285_503   ---NGLETRVEQAERFFKHKLFDW--------------TLEQKFGT--TH
UniRef90_Q6PMU0_285_503   ---NGLETRVEQAERFFKHKLFDW--------------TTDQQFGV--AH
UniRef90_Q8V0G8_86_304    ---NGLETRVEQAERFFKQKMFDW--------------TTEQSFGT--TH
UniRef90_Q6PMU2_285_503   ---NGLETRVEQAERYFKQKLFDW--------------DTTQKFGV--TH
UniRef90_Q6PMT8_285_503   ---SGLETRVQQAERFFKEKLFDW--------------TSEKPFGT--LH
UniRef90_A2I8Z5_287_504   ---SGLETRVVQAERFFKTHLFDW--------------VTSDSFGR--CH
UniRef90_Q1L754_86_304    ---SGLETRVEQAERFFKEKLFDW--------------TSDKPFGT--LH
UniRef90_B8Y0J7_285_503   ---SGLETRVQQAERFFKEKLFDW--------------TSDKPFGT--LY
UniRef90_Q1L757_86_304    ---SGLETRVRQAERIFKEKLFDW--------------TSDKPFGT--LH
UniRef90_P49303_287_504   ---SGLETRVVQAERFFKKYLFDW--------------TPDKAFGH--LE
UniRef90_A6XP89_287_504   ---SGLETRVQQAERFFKKHLFDW--------------TPNLAFGH--CH
UniRef90_Q6PMT3_285_502   ---SGLESRVEQAERFFKEKLFTW--------------TASQEFAH--VH
UniRef90_Q1L752_86_304    ---SGLETHVPQAERFFKEKLFDW--------------TSDKPFGT--TC
UniRef90_Q1L748_86_303    ---SGLDTRVEHAERFFKEKLFTW--------------TANQEYAH--VH
UniRef90_P15072_287_504   ---SGLETRVHQAERFFKMALFDW--------------VPSQNFGH--MH
```

```
UniRef90_Q8JVD0_86_303     ---SGLETRVEQAERFFKEKLLTW--------------TAAQEYAH--VH
UniRef90_H9AY35_273_496    TLAAGLAVDVPSAEKFYDLGTWDW--------------NSTHPAGR--TM
UniRef90_B0Z3W9_300_527    ---SGTHEHVDHVSRIYMKKLFTW--------------SMSDATGY--YH
UniRef90_Q91LX8_289_537    -AQDTPSKHEQSVECGFTFKLTKW--------------TAVASTWD--HL
UniRef90_Q66776_291_513    -AQDTPSKAEQSVERGFTFQLAQW--------------ETSRNIWD--HL
UniRef90_Q100Y4_72_320     -AQDKPSRAEQSVERGFTFKLTKW--------------ENSRHVWD--HL
UniRef90_Q2XP17_290_516    -VSDPVTRLGPTLSRHYTFKVGEW--------------PHSQSHGH--AW
UniRef90_P12296_138_384    -CADTASEKILAVERYYTFKVNDW--------------TSTQKPFE--YI
UniRef90_Q02472_148_405    -CADTATDKVLAAERYYTIDLATW--------------TTAQTTFS--HI
UniRef90_A4ZKN2_144_403    -CADTATDKVLAAERYYTLKLVTW--------------TTTQKPFD--HV
UniRef90_C3U5A3_174_435    -CADRATDKVLAAERYYTIKLATW--------------DTTLKAFD--HI
UniRef90_P08544_148_405    -CADTATDKVLAAERYYTIDLASW--------------TTSQEAFS--HI
UniRef90_E3TMG9_144_405    -CADTATDKVLAAERYYTIKLASW--------------TKTQESFD--HI
UniRef90_G9FTJ7_144_402    -CADTATDKVLAAERYYTFKIATW---------------STQKAFD--HV
UniRef90_B3VTU7_149_406    -CADTATDKVLSAERYYTIDLATW--------------TTTLSTFS--HI
UniRef90_Q155Z9_151_421    -STDQPTTTFTAIDRWYTGRLNSW--------------TKAVKTFS--FQ
UniRef90_G1C9J1_14_204     -AVDQSTTPGTAVDRFYTLDSFAW--------------T---ANNF-SGI
UniRef90_I1YLT5_69_326     ---DAPSRATPATSRSFVIPLQDW--------------LSSIDPYQ--YQ
UniRef90_G0Y2E2_141_365    -AIDMQTIPGPSCERFYTLDSLKW--------------T---RTGL--GY
UniRef90_G0Y2E0_150_361    -AVDMQTTPGPAVERFYTLDSWYW--------------T---KGSAQEGR
UniRef90_Q8B552_195_378    -ALDLDTRPGPSCDRFYTFDSLNW--------------Q----NTQYGEW
UniRef90_B5A1N0_195_378    -ALDLDTKPGPSCDRFYTFDALSW--------------G----STEYGEW
UniRef90_Q2LKZ0_129_301    ------------AERFYTLPSVQW--------------T---NSFK--GH
UniRef90_Q65481_129_301    ------------AERFYTLPSVQW--------------T---TTFK--GR
UniRef90_G1DFA4_229_430    -CADKPTIGGPAMERNFVQYIGTW--------------ETAQVEYQ--CR
UniRef90_H9LG14_129_301    ------------AERFYTLPSVQW--------------T---PTFK--GR
UniRef90_H6VLP2_125_335    -ALDKQSEPGPACDRFYTLDSWQW--------------T---NSSR--GY
UniRef90_B9VIN1_70_329     ---DRASKATPATQRSFIIPLKDW--------------TSSQNSYS--AL
UniRef90_G1JYY8_171_367    -SVDQFSRPGPASDRFYTLRSVNW--------------T---KNMD--PI
UniRef90_Q8QUZ6_80_281     -AVDKTTKPGVACDRFYTLPSKKW--------------E---QNSI--GW
UniRef90_I1YLT7_70_335     ---DKPSRGTAATDRSFVIQLKGW--------------TREQAAYD--AQ
UniRef90_F5B9S5_80_301     -AVDKTTKPGVACDRFYTLPGKKW--------------E---ASSL--GW
UniRef90_G0Y2E4_163_340    -AIDKETKPGPAVERFYTLDSIYW--------------N---QDWK--GR
UniRef90_B8XTQ1_73_321     ---DEPSEGTAATDRSFVIQLQPW--------------VKTNQAYT--AQ
UniRef90_H9C6N6_131_322    --------------RFYTLESKTW--------------T---NSSK--GW
UniRef90_I1YLT4_70_334     ---DEPSRGTAATDRSFVIQLKEW--------------TKTQASYD--AQ
UniRef90_B8XTQ0_73_332     ---DKPSEGTDATDRNFVFQLLPW--------------AQGQKSYD--AQ
UniRef90_B9V442_80_329     -AIDKPSRPDTSSNRFYTLESKMW--------------T---SDSK--GW
UniRef90_D2IW00_80_316     -AIDKPTHPDTSSNRFYTLESKEW--------------K---SDSK--GW
UniRef90_Q2LL04_129_301    ------------AERFYTLRSKAW--------------E---ANSP--GW
UniRef90_I1YLT6_69_335     ---DKPSEGTPATDRGFVVQLKPW--------------AKTNAAYD--AQ
UniRef90_G5DC73_80_330     -AIDKPSRPDTSSNRFYTLKSITW--------------S---RSSK--GW
UniRef90_A5GZE4_80_318     -AIDKPSQPDTSSNRFYTLESKTW--------------T---SDSK--GW
UniRef90_D9D8F3_70_328     ---DTPSKGTAATDRSFIVQLQQW--------------STTQAAYD--AQ
UniRef90_G0ZNT5_127_322    ------------ADRFYTLESVRW--------------T---QNSL--GW
UniRef90_B9V459_80_317     -AIDKPTQPDTSSNRFYTLESKLW--------------D---ETSK--GW
UniRef90_A8S322_129_323    --------------RFYTLTSVDW--------------T---TTSK--GW
UniRef90_C7DUC7_127_322    ------------ADRFYTLDSVQW--------------T---SDSK--GW
UniRef90_G8IJE2_80_317     -AIDKPSRPDTSSNRFYTLKSITW--------------S---SASK--GW
UniRef90_Q2LKZ8_129_301    ------------AERFYTLRSKPW--------------Q---SDSP--GW
UniRef90_B8XTP8_70_327     ---DDPSKGTHATDRAFVIQLLPW--------------KQTTNSYF--AQ
UniRef90_H2E685_80_301     -AVDKPTQPGVSCDRFYTLPGKKW--------------A---TEDR--GW
UniRef90_Q5YJ95_199_371    -----------AVDRFYTFDSLQW--------------T----NTQVGEW
UniRef90_Q9IM09_80_305     -AVDKPTQPDVASNRFYTLKTVSW--------------E---KSST--GW
UniRef90_A5GZE9_80_317     -AIDKPTQPDTSSNRFYTLESKTW--------------V---ETSK--GW
UniRef90_Q66790_80_316     -AEDQPTQPDVATCRFYTLESVQW--------------E---ANSA--GW
UniRef90_A5GZD8_131_315    --------------RFYTLESKTW--------------T---NSSK--GW
UniRef90_P12915_129_301    ------------ADAFYTLRSKPW--------------Q---ADSK--GW
UniRef90_Q5XLP5_80_317     -AIDKPTQPDTSSNRFYTLESKVW--------------K---RDSK--GW
UniRef90_G0Z2U8_195_360    -----------AVDRFYTFDSLQW--------------T----NTQVGEW
UniRef90_A1E4A3_131_303    --------------RFYTLKSVKW--------------E---AGST--GW
UniRef90_G0Y2E3_141_369    -AIDMQTVPGPSCERFYTFSSQSW--------------T---TTTV--GY
UniRef90_B9V482_80_298     -DVNKTSKPDTSVCRFYTLDSKDW--------------S---ANSK--GW
UniRef90_B8XTP9_157_328    --------------------------------------------------
UniRef90_F8V2V4_80_316     -AEDQPTQPDVATCRFYTLNSVKW--------------E---MQSA--GW
UniRef90_A1E4A2_131_303    --------------RFYTLKSVKW--------------E---GTSK--GW
UniRef90_Q8V5Z9_80_307     -AVDAPTHPDVSVNRFYTLKAVXW--------------E---KTST--GW
UniRef90_G8IJE1_80_316     -AIDKPTRPDTSSNRFYTLESKIW--------------M---NDSK--GW
UniRef90_P12916_80_317     -AIDKPTQPDTSSNRFYTLESKHW--------------N---GDSK--GW
UniRef90_P03303_80_315     -DVNKTSKPDTSVCRFYTLDSKTW--------------T---TGSK--GW
UniRef90_B8XTQ4_69_329     ---DDPSIATHCIERGFTINLADW--------------DKSKESWQ--AL
UniRef90_Q9YLJ1_78_316     -AEDQPTQPDVATCRFYTLESVSW--------------Q---SGSA--GW
UniRef90_B9V466_80_318     -AIDKPTQPDTSSNRFYTLESKIW--------------H---TNSK--GW
```

```
UniRef90_Q05ED6_131_323    --------------RFYTLDSVDW--------------Q---NNSA--GW
UniRef90_B5BSU7_131_316    --------------RFYTMESVNW--------------E---SNSY--GW
UniRef90_A5GZF1_80_316     -AIDKPSRPDTSSNRFYTLESKEW--------------S---ANSK--GW
UniRef90_B9V453_80_313     -AIDKPTQPDTSANRFYTLESKEW--------------T---PDSK--GW
UniRef90_Q05EE3_80_323     -AEDKPTQPDVATCRFYTLESINW--------------E---KGSA--GW
UniRef90_A5GZE7_80_317     -AIDKPTQPDTSSNRFYTLESKVW--------------T---STSQ--GW
UniRef90_B9V485_80_315     -DVNKTSKPDTSVCRFYTLDSKTW--------------N---NTSK--GW
UniRef90_G8IJH5_80_318     -AIDKPSQPDTSSNRFYTLESKLW--------------T---NESK--GW
UniRef90_B9V435_80_318     -AIDKPSHPDTSSNRFYTLESKTW--------------S---NESK--GW
UniRef90_Q8V398_80_292     -SIDAPTKPDVAVNRFYTLKAKKW--------------E---KNSS--GW
UniRef90_Q82081_159_315    --------------------------------------------------
UniRef90_Q6W9F6_159_316    --------------------------------------------------
UniRef90_Q03053_159_316    --------------------------------------------------
UniRef90_A5GZD6_131_315    --------------RFYTLDSKTW--------------Q---QDSK--GW
UniRef90_Q2YEN8_80_316     -AEDQPTQPDVATCRFYTLESVQW--------------E---KNSA--GW
UniRef90_G8HH17_117_311    -AIDKLTDPGPSVDRFYTLDSIEW--------------S---TTTQ--GY
UniRef90_A7KC08_80_331     -AIDKPSRPDTSSNRFYTLESKTW--------------T---GTSK--GW
UniRef90_Q6W9G1_131_314    --------------RFYTLESVDW--------------S---ANSN--GW
UniRef90_G8IQ64_80_315     -DVNKTSKPDTSVCRFYTLESKTW--------------Q---NNSK--GW
UniRef90_I0IY47_80_323     -SVDAPTVPDTSSDRFYTLDSVDW--------------E---KASR--GW
UniRef90_G5DC75_80_317     -AIDKPSRPDTSSNRFYTLESKVW--------------T---PTSQ--GW
UniRef90_Q05ED2_80_316     -AEDQPTQPDVATCRFYTLDSVEW--------------S---KNSQ--GW
UniRef90_Q5VAK8_80_316     -AEDQPTQPDVATCRFYTLDSVQW--------------Q---KTSN--GW
UniRef90_A7KC11_80_330     -AIDKPTQPDTSSNRFYTLESKIW--------------T---TDSK--GW
UniRef90_B5BSU6_131_316    --------------RFYTLDSIQW--------------Q---KTSP--GW
UniRef90_Q05ED0_80_316     -AEDQPTQPDVATCRFYTLESVKW--------------D---KASA--GW
UniRef90_Q5VAL5_159_315    --------------------------------------------------
UniRef90_A4UHT9_129_315    --------------RFYTLKSVTW--------------E---VGSS--GW
UniRef90_H8Y6P9_127_322    ------------ADRFYTLKSVEW--------------G---ATSI--GW
UniRef90_P29813_80_316     -AEDQPTQPDVATCRFYTLESVTW--------------E---RDSP--GW
UniRef90_P32537_131_305    --------------RFYTLKSKKW--------------E---SNST--GW
UniRef90_G5D5E2_159_315    --------------------------------------------------
UniRef90_Q9YLG5_80_319     -AEDQPTQPDVATCRFYTLSSVQW--------------Q---RESA--GW
UniRef90_B8XTQ3_70_330     ---DAPSKAVPATSRGYTVHLKDW--------------TSTIYAYY--AQ
UniRef90_A7KC13_88_321     -DVNKPTRPDTSACRFYTLASKTW--------------S---RESK--GW
UniRef90_O41174_80_282     -AVDKTTKPGVSCDRFYTLPGKKW--------------E---ATTK--GW
UniRef90_A5GZF9_80_318     -AIDKPSQPETSSNRFYTLESKTW--------------D---SSSK--GW
UniRef90_G8IJE5_80_295     -AIDKPSKPDTSSNRFYTLESQTW--------------T---NESK--GW
UniRef90_Q9QCF0_72_316     -AEDQPTQPDVATCRFYTLESIQW--------------Q---KTSD--GW
UniRef90_G5DC71_157_324    --------------------------------------------------
UniRef90_A8S330_157_325    --------------------------------------------------
UniRef90_Q9WN78_80_316     -AVDQPTQPDVATCRFYTLESVKW--------------E---SSSA--GW
UniRef90_A0MHB7_86_316     -SVDKPTHPETSADRFYTLDSVQW--------------G---GSSK--GW
UniRef90_O89280_159_323    --------------------------------------------------
UniRef90_G8IJG2_80_318     -AIDKPTHPDTSSNRFYTLESKNW--------------T---ATSK--GW
UniRef90_F6M9Y3_80_316     -AIDKPTHPDTSSNRFYTLDSKLW--------------T---NTSK--GW
UniRef90_A5GZE0_80_317     -AIDKPTRPDTSSNRFYTLESQLW--------------T---NTSK--GW
UniRef90_Q7T7N9_80_323     -PVDAPTEPDASANRFYTLDSVDW--------------G---ADSK--GW
UniRef90_A7YF31_159_315    --------------------------------------------------
UniRef90_H9X8D4_80_317     -AIDKPTQPDTSSNRFYTLESKHW--------------T---NTSK--GW
UniRef90_G9K146_80_316     -AIDKPSRPDTSSNRFYTLASKEW--------------D---YNSK--GW
UniRef90_G9K138_159_330    --------------------------------------------------
UniRef90_G0YPI0_80_292     -AVDKPTRPDVSVNRFYTLSTKSW--------------T---QNSR--GW
UniRef90_O91734_159_316    --------------------------------------------------
UniRef90_B9V473_80_315     -AIDKPSHPDTSSNRFYTLDSKLW--------------E---NGSK--GW
UniRef90_Q9WHH4_131_316    --------------RFYTLDSVEW--------------Q---KNSP--GW
UniRef90_Q2L970_80_316     -AEDQPTQPDVATCRFYTLDSVSW--------------M---KESQ--GW
UniRef90_Q80GI2_159_316    --------------------------------------------------
UniRef90_Q05ED1_131_316    --------------RFYTLDSVSW--------------E---ETSQ--GW
UniRef90_B9V440_80_317     -AIDKPSQPDTSTNRFYTLESKVW--------------D---SESK--GW
UniRef90_G1CSA8_80_313     -AVDKPTRPDVSVNRFYTLPAKLW--------------TKEGTGSK--GW
UniRef90_B1PZZ4_80_316     -AEDQPTQPDVATCRFYTLESVTW--------------E---KDSP--GW
UniRef90_B9V457_80_316     -AIDKPSHPDTSSDRFYTLDSKLW--------------S---NESK--GW
UniRef90_D8VCT6_80_323     -PVDAPTLPDSSSNRFYTLESVEW--------------Q---SSSR--GW
UniRef90_G8IRN4_129_324    --------------RFYTLESVTW--------------N---GSSQ--GW
UniRef90_Q80GI1_159_316    --------------------------------------------------
UniRef90_I1XV41_80_323     -PVDAPTMPDASSNRFYTLDSVDW--------------E---KTSR--GW
UniRef90_P21404_159_316    --------------------------------------------------
UniRef90_P23008_135_293    --------------------------------------------------
UniRef90_A5GZE2_80_315     -AIDKPSHPDTSSNRFYTLDSKLW--------------S---GDSK--GW
UniRef90_Q6W9F4_159_316    --------------------------------------------------
UniRef90_B3SRP0_80_317     -AVDKPTRPDVSVNRFYTLSAKLW--------------Q---TDSK--GW
UniRef90_Q6JKS0_159_312    --------------------------------------------------
```

```
UniRef90_G8IJG6_157_325   --------------------------------------------------
UniRef90_D4AH62_131_316   --------------RFYTLESVEW--------------Q---GSSA--GW
UniRef90_A5GZD9_129_321   ------------ACRFYTLDSKTW--------------T---NLSK--GW
UniRef90_Q66331_80_318    -AEDQPTQPDVATCRFYTLDSVQW--------------Q---KTSP--GW
UniRef90_Q6W9E8_159_323   --------------------------------------------------
UniRef90_P04936_159_330   --------------------------------------------------
UniRef90_A7KC07_80_326    -AIDKPTRPDTSSNRFYTLESKQW--------------M---ENSK--GW
UniRef90_Q66806_80_316    -AQDQPTQPDVATCRFYTLESVMW--------------E---KSSA--GW
UniRef90_A5GZF5_80_318    -AIDKPTHPDTSSNRFYTLESKHW--------------E---NNSK--GW
UniRef90_Q05ED4_159_315   --------------------------------------------------
UniRef90_G8IRN3_129_329   --------------RFYTLASVDW--------------T---QSSK--GW
UniRef90_Q82122_80_315    -AIDKPTQPDTSSNRFYTLDSKMW--------------N---STSK--GW
UniRef90_Q8V601_80_317    -AVDKPTKPDVSVNRFYTLSAKLW--------------Q---SNSK--GW
UniRef90_B9V433_129_314   ------------ACRFYTLDSKMW--------------T---QGSK--GW
UniRef90_G5EN04_80_318    -AVDKPTRPDVSVNRFYTLSTKSW--------------K---TEST--GW
UniRef90_Q8V613_159_316   --------------------------------------------------
UniRef90_Q6W9F9_80_316    -AEDQPTQPDVATCRFYTLDSVTW--------------Q---KNSN--GW
UniRef90_H9C6N7_159_315   --------------------------------------------------
UniRef90_B9V434_80_314    -AIDKPTQPDTSTNRFYTLESREW--------------R---SDSK--GW
UniRef90_A5GZF6_80_318    -AIDKPSHPDTSSNRFYTLESKNW--------------T---NTSK--GW
UniRef90_A5GZF2_80_318    -AIDKPTQPDTSSNRFYTLDSRTW--------------T---SASL--GW
UniRef90_C7DUC8_80_317    -AIDKPTQPDTSSNRFYTLDSKIW--------------K---PGSK--GW
UniRef90_G5CZJ4_127_322   ------------ADRFYTLDSVNW--------------T---STSL--GW
UniRef90_B9V477_80_318    -AIDKPTHPDTSSNRFYTLESKQW--------------D---TNSK--GW
UniRef90_G8IJH3_88_314    -DVNKPTRPDTSACRFYTLASKTW--------------S---QSSK--GW
UniRef90_Q8UY33_159_317   --------------------------------------------------
UniRef90_P07210_80_318    -AIDKPTQPDTSSNRFYTLDSRSW--------------T---SASS--GW
UniRef90_Q66577_131_323   --------------RFYTLESVQW--------------Q---ENSA--GW
UniRef90_G8IJF0_127_318   ------------ADRFYTLRSVNW--------------G---ATSK--GW
UniRef90_E7CRL8_159_323   --------------------------------------------------
UniRef90_Q995Z1_80_314    -AEDQPTQPDVATCRFYTLESVNW--------------Q---KESA--GW
UniRef90_C6ZEZ9_129_315   --------------RFYTLESVDW--------------K---SGSK--GW
UniRef90_Q5G9Z5_159_312   --------------------------------------------------
```

```
2WZR2                     VLELP----T------DHKGIYGQLVDSHAYIRNGWDVQVSATATQFNGG
UniRef90_F1AW55_285_503   VLELP----T------DHKGIYGQLVDSHSYIRNGWDVQVSATATQFNGG
UniRef90_Q6PMU0_285_503   ILELP----T------DHKGIYGQLVDSHSYIRNGWDVEVSATATQFNGG
UniRef90_Q8V0G8_86_304    VLELP----T------DHKGIYGQLVDSHAYIRNGWDVEVSATATLFNGG
UniRef90_Q6PMU2_285_503   VLELP----T------DHKGVYGQLVDSYTYMRNGWDVQVSATATQFNGG
UniRef90_Q6PMT8_285_503   VLELP----K------DHKGIYGSLTDAYTYMRNGWDVQVTATSTQFNGG
UniRef90_A2I8Z5_287_504   LLELP----T------DHKGVYGSLTDSYAYMRNGWDVEVTAVGNQFNGG
UniRef90_Q1L754_86_304    ILELP----K------DHKGIYGSLTESYAYMRNGWDVQVSATSTQFNGG
UniRef90_B8Y0J7_285_503   VLELP----K------DHKGIYGKLTDSYTYMXNGWDVQVSATSTQFNGG
UniRef90_Q1L757_86_304    VLELP----K------DHKGIYGQLTEAYTYMRNGWDVQVTATSTQFNGG
UniRef90_P49303_287_504   KLELP----T------DHKGVYGHLVDSFAYMRNGWDVEVSAVGNQFNGG
UniRef90_A6XP89_287_504   YLELP----T------EHKGVYGSLMDSYAYMRNGWDIEVTAVGNQFNGG
UniRef90_Q6PMT3_285_502   LLELP----T------DHKGIYGAMVESHAYVRNGWDVQVSATSTQFNGG
UniRef90_Q1L752_86_304    VLELP----K------DHKGIYGKLNDSYAYMRNGWDVQVSATSTQFNGG
UniRef90_Q1L748_86_303    CLELP----T------DHKGVYGQLVDSHTYIRNGWDVQVTATSTQSNGG
UniRef90_P15072_287_504   KVVLP----H------EPKGVYGGLVKSYAYMRNGWDVEVTAVGNQFNGG
UniRef90_Q8JVD0_86_303    LLELP----V------DHKGIYGAMLDSHAYVRNGWDVQVSATSTQFNGG
UniRef90_H9AY35_273_496   RYPLPS---S------LRKGAFANLSKTYVLMQNGWEVVVTAQGTLSHGG
UniRef90_B0Z3W9_300_527   AYPLPDLILT------EARE-YHNLLKSYALYRNGWEVHVSVVSTMYHSG
UniRef90_Q91LX8_289_537   AIPLPMS-----PGLFKNSGMYKNFVETHYLIKNGWKVQVQSNSSQFHSG
UniRef90_Q66776_291_513   TIPLPMC-----PGLIKVSGMYKAFIETHAYIKNGWKIQVQCNASQFHSG
UniRef90_Q100Y4_72_320    TIPLPMC-----PGLMKISGMYKSFVETHYLIKNGWKVQVQCNASQFHSG
UniRef90_Q2XP17_290_516   ICPLP-------GDKLKKMGSFHEVVKSHHLVKNGWDVVVQVNASFAHSG
UniRef90_P12296_138_384   RIPLPHVL----SG--EDGGVFGATLRRHYLVKTGWRVQVQCNASQFHAG
UniRef90_Q02472_148_405   RVPLPHAL----AG--EHGGVFGATLRRHYLAKCGWRVQVQCNASQFHAG
UniRef90_A4ZKN2_144_403   RIPLPHVL----AG--KQGGVFGSTLRRHYLCKCGWRIQVQCNASQFHAG
UniRef90_C3U5A3_174_435   RVPFPHIL----SG--KEGGVFGATLRRHYLCKCGWRIQVQCNASQFHAG
UniRef90_P08544_148_405   RIPLPHVL----AG--EDGGVFGATLRRHYLCKTGWRVQVQCNASQFHAG
UniRef90_E3TMG9_144_405   RVPLPHAL----AG--ENGGVFSSTLRRHYLCKCGWRIQVQCNASQFHAG
UniRef90_G9FTJ7_144_402   RVPFPHVL----SG--SKGGVFGSTLRRHYLCKCGWRIQVQCNASQFHAG
UniRef90_B3VTU7_149_406   RIPLPHVL----AG--EDGGVFGSTLRRHYLCKCGWRIQVQCNASQFHAG
UniRef90_Q155Z9_151_421   AVPLPGAFLSRQGG--LNGGAFTATLHRHFLMKCGWQVQVQCNLTQFHQG
UniRef90_G1C9J1_14_204    CYRLPGVL--------SDMGMFGQNLRYHYLMRCGFLCHVQINATKFHQG
UniRef90_I1YLT5_69_326    IYRTSQLRKE------SVGNLYSKSLNSHSYTKTGFEVTLQVNTSPFHAG
UniRef90_G0Y2E2_141_365   GIKLPAAL--------TDLGVFGQNCQFHFLCHTGYCVHVQVNASKFHQG
UniRef90_G0Y2E0_150_361   AYKLPAAL--------TDLGVFGQNCQFHFLCRSGFCVHVQVNATKFHQG
```

```
UniRef90_Q8B552_195_378   TCPLPGAL--------MDTGVFGQNLRFHYLYRSGFCVHVQVNASSFHQG
UniRef90_B5A1N0_195_378   ALPLPGGL--------SDMGVFGQNLKFHYLYRSGFCVNVQCNASKFHQG
UniRef90_Q2LKZ0_129_301   YWKLPDAL--------SELGLFGQNLQFHYLYRGGWVIHVQCNATKFHQG
UniRef90_Q65481_129_301   FWKLPDAL--------SQLGLFGQNLQFHYLYRGGWAIHVQCNATKFHQG
UniRef90_G1DFA4_229_430   YLPLPHGL--------EEMGVFETTAIRHYTMKCGWKIQVQLNTSHFHAG
UniRef90_H9LG14_129_301   YWKLPDAL--------SDLGLFGQNLQYHYLYRGGWAIHVQCNATKFHQG
UniRef90_H6VLP2_125_335   GYDLPGCL--------TDIGIFGQNCTYHFLMRSGFCIHVQLNASKFHQG
UniRef90_B9VIN1_70_329    VYRTSHMSKD------AVGNLFSKNMNTHAYTKTGWTATLQINTSPFHSG
UniRef90_G1JYY8_171_367   IVPFPGAL--------QDIGVFGQNLKFHFLYNGGACFHVQCNASQFHQG
UniRef90_Q8QUZ6_80_281    EWKLPDAL--------TELGVFGQNSQYHFLSRCGWAIHVQANATRFHQG
UniRef90_I1YLT7_70_335    WVKLTQELRKE-----HKGNVFAKNLKTHTFVKAGYEVTLQINTSPFHTG
UniRef90_F5B9S5_80_301    EWKLPDAL--------TQLGVFGQNCQFHYLSRCGWTIHVQCNATKFHQG
UniRef90_G0Y2E4_163_340   VIRLPGAL--------TDLGMFGQNCQFHFLMRSGFCVHVQVNASKFHQG
UniRef90_B8XTQ1_73_321    WVRLTQELRQD-----HKGNVFAKNLKSHAFAKLGFEVTVQVNTSPFHCG
UniRef90_H9C6N6_131_322   CWKLPDAL--------KEMGVFGQNMFFHALGRTGYTIHVQCNATKFHSG
UniRef90_I1YLT4_70_334    WVRLTQELRKE-----IKGNVFAKNLKTHTFVKAGYEVTLQVNTSPFHCG
UniRef90_B8XTQ0_73_332    WIRITKDLRQD-----NKGNVFAKNMKSHAYMKAGYEVTLQVNTSPFHCG
UniRef90_B9V442_80_329    WWKLPDAL--------KNMGIFGENMFYHFLGRSGYTVHVQCNASKFHQG
UniRef90_D2IW00_80_316    WWKLPDAL--------KNMGIFGENLFYHFLGRAGYTVHVQCNASKFHQG
UniRef90_Q2LL04_129_301   YWKLPDAL--------NNTGMFGQNAQFHYIYRGGWAVHVQCNATKFHQG
UniRef90_I1YLT6_69_335    WYRITDQLKID-----ERGNIFTKNMKSHAYLKAGYEVTLQVNTSPFHCG
UniRef90_G5DC73_80_330    WWKLPDAL--------RDMGIFGENMFYHYLGRSGYTVHVQCNATKFHQG
UniRef90_A5GZE4_80_318    WWKLPDAL--------KEMGVFGQNMYYHFLGRSGYTVHVQCNSSKFHQG
UniRef90_D9D8F3_70_328    WVRLTQELRAD-----NKGNVFAKNLKTHSYAKMGFEVTLQVNTSPFHCG
UniRef90_G0ZNT5_127_322   WWKLPDCL--------KDMGLFGQNMYHHSMGRTGYIIHVQCNATKFHSG
UniRef90_B9V459_80_317    WWKLPDAL--------KDMGIFGENMFYHFLGRSGYTVHVQCNASKFHQG
UniRef90_A8S322_129_323   WWKLPDCL--------KDMGIFGQNLYHHALGRSGYIIHTQCNATKFNSG
UniRef90_C7DUC7_127_322   WWKLPDAL--------KDMGLFGQNMYYHSMGRSGYIVHVQCNATKFHSG
UniRef90_G8IJE2_80_317    WWKLPDAL--------RDMGIFGENMFYHYLGRSGYTVHVQCNATKFHQG
UniRef90_Q2LKZ8_129_301   YWKLPDAL--------NNTGMFGQNAQFHYIYRGGWAVHVQCNATKFHQG
UniRef90_B8XTP8_70_327    WVRLTQKLSNN-----LHGNVMAKNIKSHAFAKMGFEVMLQANTSPFHNG
UniRef90_H2E685_80_301    EWKLPDAL--------TELGVFGQNCQYHYLARCGWIIHVQCNATKFHQG
UniRef90_Q5YJ95_199_371   SLPLPGGL--------MDTGVFGQNLRFHYLSRMGFCVHVQCNASKFHQG
UniRef90_Q9IM09_80_305    YWKFSDCL--------ASVGLFGQNVQYHYLGRYGLAVHVQCNASKFHQG
UniRef90_A5GZE9_80_317    WWKLPDAL--------KDMGIFGENMFYHFLGRSGYTVHVQCNASKFHQG
UniRef90_Q66790_80_316    WWKFPEAL--------KDMGLFGQNMYYHYLGRAGYTIHVQCNASKFHQG
UniRef90_A5GZD8_131_315   CWKLPDAL--------KDMGVFGQNMFFHALGRTGYTIHVQCNATKFHSG
UniRef90_P12915_129_301   YWKLPDAL--------NNTGMFGQNAQFHYIYRGGWAVHVQCNATKFHQG
UniRef90_Q5XLP5_80_317    WWKLPDAL--------KDMGIFGENMYYHFLGRSGYTVHVQCNASKFHQG
UniRef90_G0Z2U8_195_360   SLPLPGGL--------MDVGVFGQNLRFHYLSRMGFCVHVQCNASKFHQG
UniRef90_A1E4A3_131_303   WWKLPDAL--------NNIGMFGQNVQHHYLYRSGFLIHVQCNATKFHQG
UniRef90_G0Y2E3_141_369   GLKLPLAL--------TDMGVFGQNCQYHFLCHSGFCVHVQVNASKFHQG
UniRef90_B9V482_80_298    CWKLPDAL--------KDMGVFGQNMFFHSLGRTGYTIHVQCNATKFHSG
UniRef90_B8XTP9_157_328   --------LQN-----NKANVFAKNLKAHSYLRAGYEVTLQVNTSPFHIG
UniRef90_F8V2V4_80_316    WWKFPDAL--------SEMGLFGQNMQYHYLGRSGYTIHVQCNASKFHQG
UniRef90_A1E4A2_131_303   WWKLPDAL--------NQTGMFGQNVQYHYLYRSGFLCHVQCNATKFHQG
UniRef90_Q8V5Z9_80_307    YWKLPDCL--------NQLGVFGQNAQYHYLYRSGFAVHVQCNASKFHQG
UniRef90_G8IJE1_80_316    WWKLPDAL--------KDMGVFGQNMYYHFLGRSGYTIHVQCNASKFHQG
UniRef90_P12916_80_317    WWKLPDAL--------KEMGIFGENMYYHFLGRSGYTVHVQCNASKFHQG
UniRef90_P03303_80_315    CWKLPDAL--------KDMGVFGQNMFFHSLGRSGYTVHVQCNATKFHSG
UniRef90_B8XTQ4_69_329    VYRLSDHLKDD-----TVGNMFSKTLGTHAYTKCGYRVSLQINTSPFHSG
UniRef90_Q9YLJ1_78_316    WWKFPEAL--------KDMGLFGQNMYYHYLGRAGYTIHVQCNASKFHQG
UniRef90_B9V466_80_318    WWKLPDAL--------KDMGIFGENMYYHYLGRSGYTVHVQCNASKFHQG
UniRef90_Q05ED6_131_323   WWKFPNAL--------KDMGLFGQNMHYHYLGRTGYTVHVQCNASKFHQG
UniRef90_B5BSU7_131_316   WWKFPEAL--------KNMGLFGQNMYYHYLGRAGYTIHVQCNASKFHQG
UniRef90_A5GZF1_80_316    WWKLPDAL--------KDMGIFGENMFYHFLGRSGYTVHVQCNASKFHQG
UniRef90_B9V453_80_313    WWKLPDAL--------KDMGVFGENMFYHALGRSGYLIHVQCNASKFHSG
UniRef90_Q05EE3_80_323    WWKFPIAL--------KDMGLFGQNMLYHYLGRAGYTIHVQCNASKFHQG
UniRef90_A5GZE7_80_317    WWKLPDAL--------KDMGIFGENMFYHYLGRSGYTVHVQCNASKFHQG
UniRef90_B9V485_80_315    CWKLPDAL--------KDMGVFGQNMFFHSLGRSGYTIHVQCNATKFHSG
UniRef90_G8IJH5_80_318    WWKLPDAL--------KDMGVFGQNMYYHFLGRSGYTVHVQCNSSKFHQG
UniRef90_B9V435_80_318    WWKLPDAL--------KDMGIFGQNMYYHFLGRSGYTVHVQCNSSKFHQG
UniRef90_Q8V398_80_292    MWKLPDCL--------NDTGVFGQNCWYHFLYRGGWAVHVQCNASKFHQG
UniRef90_Q82081_159_315   -------------------GVFGQNMFYHSLGRTGYTIHVQCNATKFHSG
UniRef90_Q6W9F6_159_316   -------------------GLFGQNMLYHYLGRTGYTIHVQCNASKFHQG
UniRef90_Q03053_159_316   -------------------GLFGQNMQYHYLGRAGYTVHVQCNASKFHQG
UniRef90_A5GZD6_131_315   CWKLPDAL--------KDMGVFGQNMFYHTLGRTGYTIHVQCNATKFHSG
UniRef90_Q2YEN8_80_316    WWKFPEAL--------KDMGLFGQNMLYHYLGRAGYTIHVQCNASKFHQG
UniRef90_G8HH17_117_311   YYNLPGCL--------TNLGMFGQNCAYHYLMRSGFCVHIQVNASKFHQG
UniRef90_A7KC08_80_331    WWKLPDAL--------KEMGIFGENMFYHFLGRSGYTVHVQCNASKFHQG
UniRef90_Q6W9G1_131_314   WWKFPEAL--------KDMGLFGQNMLYHYLGRAGYTIHVQCNASRFHQG
UniRef90_G8IQ64_80_315    CWKLPDAL--------KDMGVFGQNMFFHSLGRSGYTIHVQCNATKFHSG
UniRef90_I0IY47_80_323    WWKLPDAL--------KDMGMFGQNMYHHYLGRAGYTIHVQCNASKFHQG
```

```
UniRef90_G5DC75_80_317    WWKLPDAL--------KDMGIFGENMFYHYLGRSGYTVHVQCNASKFHQG
UniRef90_Q05ED2_80_316    WWKFPEAL--------KDMGLFGQNMLYHYLGRTGYTIHVQCNASKFHQG
UniRef90_Q5VAK8_80_316    WWKFPDAL--------KDMGLFGQNMLYHYLGRAGYTIHVQCNASRFHQG
UniRef90_A7KC11_80_330    WWKLPDAL--------KDMGIFGQNMYYHFLGRSGYTIHVQCNASKFHQG
UniRef90_B5BSU6_131_316   WWKFPDAL--------KDMGLFGQNMYYHYLGRAGYTVHVQCNASKFHQG
UniRef90_Q05ED0_80_316    WWKFPEAL--------KDMGLFGQNMYYHYLGRSGYTIHVQCNASRFHQG
UniRef90_Q5VAL5_159_315   -------------------GLFGQNMHYHYLGRAGYTIHVQCNASKFHQG
UniRef90_A4UHT9_129_315   WWKLPDCL--------RDMGVFGQNMYHHAMGRSGYIIHTQCNATKFHSG
UniRef90_H8Y6P9_127_322   WWKLPDCL--------KDMGIFGQNMYYHSFGRSGFLIHVQCNATKFHTG
UniRef90_P29813_80_316    WWKFPDAL--------KDMGLFGQNMYYHYLGRAGYTLHVQCNASKFHQG
UniRef90_P32537_131_305   WWKLPDAL--------NQIGMFGQNVQYHYLYRSGFLCHVQCNATKFHQG
UniRef90_G5D5E2_159_315   -------------------GVFGQNMFFHSLGRSGYTIHVQCNATKFHSG
UniRef90_Q9YLG5_80_319    WWKFPDAL--------SDMGLFAQNMMYHYLGRTGYTIHVQCNASKFHQG
UniRef90_B8XTQ3_70_330    LYRISDRIKDP-----AIGNLFSRNMDAHSFTKTGYEVMLQVNTSPFHSG
UniRef90_A7KC13_88_321    CWKLPDAL--------KDMGIFGQNMFYHSQGRTGYTIHVQCNATKFHSG
UniRef90_O41174_80_282    EWKLPDAL--------TELGVFGQNCQFHFLYRCGWSIHVQCNATKFHQG
UniRef90_A5GZF9_80_318    WWKLPDAL--------KDMGIFGQNMYYHFLGRSGYTVHVQCNASKFHQG
UniRef90_G8IJE5_80_295    WWKLPDAL--------KDMGIFGENMFYHFLGRSGYTVHVQCNASKFHQG
UniRef90_Q9QCF0_72_316    WWKFPEAL--------KDMGLFGQNMHYHYLGRSGYTIHVQCNASKFHQG
UniRef90_G5DC71_157_324   -------------------GIFGQNMYHHAMGRSGYIIHTQCNATKFHSG
UniRef90_A8S330_157_325   -------------------GMFGQNMYYHALGRSGYIFHVQCNATKFHSG
UniRef90_Q9WN78_80_316    WWKFPEAL--------SDMGLFGQNMQYHYLGRAGYTIHVQCNASKFHQG
UniRef90_A0MHB7_86_316    WWKLPDCL--------KNMGIFGQNMYYHAMGRSGYIIHTQCNATKFHSG
UniRef90_O89280_159_323   -------------------GLFGQNMQYHYLGRSGYTIHVQCNASKFHQG
UniRef90_G8IJG2_80_318    WWKLPDAL--------KNMGIFGENLFYHFLGRAGYTVHVQCNASKFHQG
UniRef90_F6M9Y3_80_316    WWKLPDAL--------KNMGIFGENMFYHFLGRAGYTVHVQCNASKFHQG
UniRef90_A5GZE0_80_317    WWKLPDAL--------KDMGIFGENMFYHFLGRSGYTVHVQCNASKFHQG
UniRef90_Q7T7N9_80_323    WWKLPDAL--------KDMGMFGQNMYYHYLGRAGYTVHVQCNASKFHQG
UniRef90_A7YF31_159_315   -------------------GLFGQNMHYHYLGRSGYTIHVQCNASKFHQG
UniRef90_H9X8D4_80_317    WWKLPDAL--------KDMGIFGENMYYHFLGRSGYTVHVQCNASKFHQG
UniRef90_G9K146_80_316    WWKLPDAL--------KDMGIFGENMFYHFLGRSGYTVHVQCNASKFHQG
UniRef90_G9K138_159_330   -------------------GIFGENMFYHFLGRSGYTVHVQCNASKFHQG
UniRef90_G0YPI0_80_292    YWKFPDVL--------NDVGVFGQNAQYHYLYRSGFCVHVQCNASKFHQG
UniRef90_O91734_159_316   -------------------GLFGQNMYYHYLGRAGYTIHVQCNASKFHQG
UniRef90_B9V473_80_315    WWKLPDAL--------KNMGIFGENMFYHFLGRAGYTVHVQCNASKFHQG
UniRef90_Q9WHH4_131_316   WWKFPDAL--------NDMGLFGQNMLYHYLGRAGYTIHVQCNASKFHQG
UniRef90_Q2L970_80_316    WWKFPDAL--------RDMGLFGQNMQYHYLGRSGYTIHVQCNASKFHQG
UniRef90_Q80GI2_159_316   -------------------GLFGQNMYYHYLGRAGYTIHVQCNASRFHQG
UniRef90_Q05ED1_131_316   WWRFPDAL--------QDMGLFGQNMLYHYLGRTGYTIHVQCNASKFHQG
UniRef90_B9V440_80_317    WWKLPDAL--------KDMGVFGQNMYYHFLGRSGYTIHVQCNASKFHQG
UniRef90_G1CSA8_80_313    YWKFPDVL--------TEKGVFGQNAQFHFLYRSGFCIHVQCNASKFHQG
UniRef90_B1PZZ4_80_316    WWKFPDAL--------KDMGLFGQNMYYHYLGRAGYTIHVQCNASKFHQG
UniRef90_B9V457_80_316    WWKLPDAL--------KDMGIFGENMFYHFLGRTGYTIHVQCNASKFHQG
UniRef90_D8VCT6_80_323    WWKLPDAL--------KDMGMFGQNMYYHYLGRAGYTVHVQCNASKFHQG
UniRef90_G8IRN4_129_324   WWKLPDCL--------KDMGIFGQNMYHHAMGRSGYIIHTQCNATKFHSG
UniRef90_Q80GI1_159_316   -------------------GMFGQNMFYHYLGRAGYTIHVQCNASRFHQG
UniRef90_I1XV41_80_323    WWKLPDAL--------KDMGMFGQNMYHHYLGRAGYTVHVQCNASKFHQG
UniRef90_P21404_159_316   -------------------GLFGQNMQYHYLGRAGYTIHLQCNASKFHQG
UniRef90_P23008_135_293   -------------------GIFGENMYYHFLGRSGYTVHVQCNASKFHQG
UniRef90_A5GZE2_80_315    WWKLPDAL--------RDMGVFGQNMYYHFLGRSGYTVHVQCNSSKFHQG
UniRef90_Q6W9F4_159_316   -------------------GLFGQNMYYHYLGRAGYTIHVQCNASRFHQG
UniRef90_B3SRP0_80_317    YWKFPDVL--------TETGVFGQNAQYHYLYRSGFCIHVQCNASKFHQG
UniRef90_Q6JKS0_159_312   -------------------GVFGQNAQYHYLYRSGFCIHVQCNASKFHQG
UniRef90_G8IJG6_157_325   -------------------GIFGQNMYYHSMGRSGYIIHTQCNATKFNSG
UniRef90_D4AH62_131_316   WWKFPEAL--------KDMGLFGQNMHYHYLGRAGYTIHVQCNASKFHQG
UniRef90_A5GZD9_129_321   CWKLPDAL--------KDMGIFGQNMFFHSQGRTGYTIHVQCNATKFHSG
UniRef90_Q66331_80_318    WWKLPDAL--------SNLGLFGQNMQYHYLGRTGYTDHVQCNASKFHQG
UniRef90_Q6W9E8_159_323   -------------------GLFGQNMYYHYLGRSGYTLHVQCNASRFHQG
UniRef90_P04936_159_330   -------------------GIFGENMFYHYLGRSGYTIHVQCNASKFHQG
UniRef90_A7KC07_80_326    WWKLPDAL--------KDMGIFGENMFYHFLGRAGYTIHVQCNASKFHQG
UniRef90_Q66806_80_316    WWKFPDAL--------ADMGLFGQNMLYHYLGRSGYTIHVQCNASKFHQG
UniRef90_A5GZF5_80_318    WWKLPDAL--------KSMGIFGENMFYHHLGRAGYTIHVQCNASKFHQG
UniRef90_Q05ED4_159_315   -------------------GLFGQNMYYHYLGRAGYTIHVQCNASKFHQG
UniRef90_G8IRN3_129_329   WWKIPDCL--------KDMGMFGQNMFHHALGRSGYIIHTQCNATKFHSG
UniRef90_Q82122_80_315    WWKLPDAL--------KDMGIFGENMFYHFLGRSGYTVHVQCNASKFHQG
UniRef90_Q8V601_80_317    YWKFPDVL--------TETGVFGQNAQFHYLYRSGFCIHVQCNASKFHQG
UniRef90_B9V433_129_314   CWKLPDAL--------KDMGIFGQNMFFHSQGRTGYTIHVQCNATKFHSG
UniRef90_G5EN04_80_318    YWKFPDVL--------NDTGVFGQNAQFHYLYRSGFCMHVQCNASKFHQG
UniRef90_Q8V613_159_316   -------------------GLFGQNMLYHYLGRAGYTFHVQCNASRFHQG
UniRef90_Q6W9F9_80_316    WWKFPDAL--------KDMGLFGQNMYYHYLGRAGYTIHVQCNASKFHQG
UniRef90_H9C6N7_159_315   -------------------GIFGENMFYHFLGRAGYTVHVQCNASKFHQG
UniRef90_B9V434_80_314    WWKLPDAL--------KDMGVFGENMFYHSLGRAGYMIHVQCNASKFHSG
UniRef90_A5GZF6_80_318    WWKLPDAL--------KNMGIFGENLFYHFLGRAGYTVHVQCNASKFHQG
```

```
UniRef90_A5GZF2_80_318    WWKLPDAL--------KNMGIFGENMFYHFLGRSGYTIHVQCNSSKFHQG
UniRef90_C7DUC8_80_317    WWKLPDAL--------KEMGIFGENMHYHYLGRSGYTVHVQCNASKFHQG
UniRef90_G5CZJ4_127_322   WWKLPDCL--------REMGLFGQNMYYHSMGRTGYIIHVQCNATKFHSG
UniRef90_B9V477_80_318    WWKLPDAL--------KSMGIFGENMFYHHLGRAGYTIHVQCNASKFHQG
UniRef90_G8IJH3_88_314    CWKLPDAL--------KDMGIFGQNMFYHSQGRTGYTIHVQCNATKFHSG
UniRef90_Q8UY33_159_317   -------------------GVFGQNAQFHYLYRSGFCIHVQCNASKFHQG
UniRef90_P07210_80_318    WWKLPDAL--------KNMGIFGENMFYHFLGRSGYTIHVQCNSSKFHQG
UniRef90_Q66577_131_323   WWKFPDAL--------SNMGLFGQNMLYHYLGRSGYTIHVQCNASKFHQG
UniRef90_G8IJF0_127_318   WWKFPDAL--------SEMGVFGQNMYYHAMGRSGYIIHTQCNATKFHSG
UniRef90_E7CRL8_159_323   -------------------GLFGQNMYYHYLGRSGYTVHVQCNASKFHQG
UniRef90_Q995Z1_80_314    WWKFPDAL--------RDMGLFGQNMLYHYLGRSGYTIHVQCNASKFHQG
UniRef90_C6ZEZ9_129_315   WWKFPDAL--------KDMGMFGQNMYHHSMGRFGALIHVQCNATKFHSG
UniRef90_Q5G9Z5_159_312   -------------------GVFGQNAQFHYLYRSGFCVHVQCNASKFHQG


2WZR2                     CLLVAMVPEL----------------------------------------
UniRef90_F1AW55_285_503   CLLVAMVPEL----------------------------------------
UniRef90_Q6PMU0_285_503   CLLVAMVPEL----------------------------------------
UniRef90_Q8V0G8_86_304    CLLVAMVPEL----------------------------------------
UniRef90_Q6PMU2_285_503   CLLVAMVPEL----------------------------------------
UniRef90_Q6PMT8_285_503   SLLVAMVPEL----------------------------------------
UniRef90_A2I8Z5_287_504   CLLVAMVPEL----------------------------------------
UniRef90_Q1L754_86_304    SLLVAMVPEL----------------------------------------
UniRef90_B8Y0J7_285_503   SLLVAMVPEL----------------------------------------
UniRef90_Q1L757_86_304    SLLVAMVPEL----------------------------------------
UniRef90_P49303_287_504   CLLVAMVPEW----------------------------------------
UniRef90_A6XP89_287_504   CLLVALVPEL----------------------------------------
UniRef90_Q6PMT3_285_502   TLLVAMVPEL----------------------------------------
UniRef90_Q1L752_86_304    SLLVAMVPEL----------------------------------------
UniRef90_Q1L748_86_303    ALLVALVPEL----------------------------------------
UniRef90_P15072_287_504   CLQAALVPEM----------------------------------------
UniRef90_Q8JVD0_86_303    TLLVAMVPEL----------------------------------------
UniRef90_H9AY35_273_496   MLLVAMVPEL----------------------------------------
UniRef90_B0Z3W9_300_527   CLIVAMVPEF----------------------------------------
UniRef90_Q91LX8_289_537   CLLVVMVPEYVAE------------QQRDFRGS---------WQDKDSDG
UniRef90_Q66776_291_513   CLLVAMIPEYLST------------AQQDFLGS---------WRDKTTDS
UniRef90_Q100Y4_72_320    CLLVVMVPEYVAT------------SQTDFRGS---------WQDKENDN
UniRef90_Q2XP17_290_516   ALCVAAVPEY------------------KHTHE---------KALKWSEL
UniRef90_P12296_138_384   SLLVFMAPEY---------------PT--------------------LDV
UniRef90_Q02472_148_405   SLLVFLAPEF---------------YTGTGVAT---------SGQEPNKV
UniRef90_A4ZKN2_144_403   SLLVFMAPEF---------------DTSSYSAV---------DDPIGEEP
UniRef90_C3U5A3_174_435   SLLIFMAPEF---------------DTSPYESE---------TEPDKNKV
UniRef90_P08544_148_405   SLLVFMAPEF---------------YTGKGTKT---------GTMEPSDP
UniRef90_E3TMG9_144_405   SLLVFMAPEF---------------DTSNHSTE---------VEPRADTA
UniRef90_G9FTJ7_144_402   SLLVFMAPEF---------------DTSPYNAT---------TEPTKAVP
UniRef90_B3VTU7_149_406   SLLVFMAPEF---------------YTGHTPAT---------GVTEPATP
UniRef90_Q155Z9_151_421   ALLVAMVPETTLDV----------KPDGKAKSL---------QELNEEQW
UniRef90_G1C9J1_14_204    CVMVVAIPECEYID----T-NVTGLDDTYTPGA---------QWRA----
UniRef90_I1YLT5_69_326    LVGLFMVPEFVRYTSP-SLTWENMLTK-FN--L-FKN-----TNIYTQDA
UniRef90_G0Y2E2_141_365   MLLVCAIPDCKVDG----R---DNAT-VYNLEA---------KDLE----
UniRef90_G0Y2E0_150_361   MLLVAAIPDCQVTE----H----GMNSAYPLSA---------VDFV----
UniRef90_Q8B552_195_378   CLLVAMVPEHQ--------------------------------TPKQQNN
UniRef90_B5A1N0_195_378   MLLVAMVPEHQ--------------------------------TPTQLAN
UniRef90_Q2LKZ0_129_301   TLLVVATPEHKIQ-----------SAESPAFAR---------TNPGEQGA
UniRef90_Q65481_129_301   TLLVVAIPEHKIQ-----------SQQTPEFAK---------TNPGEDGY
UniRef90_G1DFA4_229_430   CLGVFAVPEA-----------------------------------QWVGK
UniRef90_H9LG14_129_301   TLLVVAIPEHKIQ-----------AQQTPDFTR---------TNPGENGA
UniRef90_H6VLP2_125_335   MMLVCAIPECQSSS----G---ERSNSIFQLQE---------RDFA----
UniRef90_B9VIN1_70_329    LIGLFMVPEFVRKISD-DLEWIDLVAAGFNTTL-FKD-----SNTYNQDS
UniRef90_G1JYY8_171_367   ALLVAAVPEAVKLADD-FDPDQTHRPEVYPYQS---------KLGA----
UniRef90_Q8QUZ6_80_281    CLLVVAVPEHQLG-----------SKLNPEFGT---------TMPGKEGR
UniRef90_I1YLT7_70_335    MIGLFLVPKFTRMDLM-TWAWQNFHRK---LFFSLKV-----PNSMTPRV
UniRef90_F5B9S5_80_301    CLLVVAVPEHQLG-----------YTENIEFTQ---------AMPGKNGR
UniRef90_G0Y2E4_163_340   MLLVAMVPEAQHPT----IGTNDNAA----LSD---------ETLA----
UniRef90_B8XTQ1_73_321    MLGLFLVPEFTRPGPV-NLEWKDLTEK---SVI-INN-----TDIYKPQT
UniRef90_H9C6N6_131_322   CLLVVVIPEHQLASHT-E------GNVSVKYKY---------THPGETGI
UniRef90_I1YLT4_70_334    LVGLFLVPEFTRYGPE-NLGWQNLTTR---LSL-VKN-----SNLYETQN
UniRef90_B8XTQ0_73_332    LIGLFLVPEFTRPGPV-NLEWKNLADR---RPI-MSD-----TSIWTPQN
UniRef90_B9V442_80_329    TLIVVMIPEHQLASAS-T------GNVTAGYNL---------THPGEQGR
UniRef90_D2IW00_80_316    ALVVAAIPEHQLAYIG-S------ENVSVGYKH---------THPGESGR
UniRef90_Q2LL04_129_301   TLLVVAIPEHQIA-----------TQEQPDFGR---------TMPGDDGG
```

```
UniRef90_I1YLT6_69_335     LVGLFMVPEWTRYGPTGEISWTNLLTR---LTL-VKN-----NDLYEPQT
UniRef90_G5DC73_80_330     TLVVAMIPEHQIASLQ-H------GDVNVGYNF---------THPGEEGR
UniRef90_A5GZE4_80_318     TLIVAMIPEHQLAAAE-K------GNVTAGYNL---------THPGETGR
UniRef90_D9D8F3_70_328     LVGLFLVPEFTRFGPD-NLTWQDITTR---LSL-VNN-----PSLYDPQN
UniRef90_G0ZNT5_127_322    CLMVAVVPEHQLAFIG-A------DGTKVKYSQ---------THPGERGH
UniRef90_B9V459_80_317     TLLVAMVPEHQLASAS-R------GNVTAGYNY---------THPGELGR
UniRef90_A8S322_129_323    CLIVAVVPEHQLAYIG-E------ANVNVGYDH---------THPGEGGH
UniRef90_C7DUC7_127_322    CLMVAVIPEHQLSYIG-G------DGTNVKYKH---------THPGEAGH
UniRef90_G8IJE2_80_317     TLIVAMIPEHQIASLQ-H------GNVNVGYKF---------THPGEEGR
UniRef90_Q2LKZ8_129_301    TLLVVAIPEHQIA-----------TQEQPDFGR---------TMPGETGG
UniRef90_B8XTP8_70_327     ILGLFLVPEFVRKGEI-TDEWIDLTPT---SSL-VSN-----TELYNPQT
UniRef90_H2E685_80_301     CLLVLAVPDHQLG-----------TTYNPDFNN---------TMPGKAGR
UniRef90_Q5YJ95_199_371    ALIVAMIPEHQ--------------------------------TPTQVSN
UniRef90_Q9IM09_80_305     TLLVLAIPEWEIGVS---------NADRASFNL---------TNPDKNGH
UniRef90_A5GZE9_80_317     TLLVAMVPEHQLASAN-I------GNVTVGYNY---------THPGESGR
UniRef90_Q66790_80_316     CLLVVCVPEAEMGCAK--------PDENVDATN---------LTNGENTC
UniRef90_A5GZD8_131_315    CLLVVVVPEHQLASHT-Q------GNVSVKYKY---------THPGEQGI
UniRef90_P12915_129_301    TLLVLAIPEHQIA-----------TQEQPAFDR---------TMPGSEGG
UniRef90_Q5XLP5_80_317     TLLIAMVPEHQLASAN-Y------GNVTAGYNY---------THPGEAGR
UniRef90_G0Z2U8_195_360    ALLVAMVPEHQ--------------------------------TPQQVSN
UniRef90_A1E4A3_131_303    ALLVVAIPEHQRGAYN--------TNTSPEFND---------IMKGEEGG
UniRef90_G0Y2E3_141_369    LLLVCAIPDCQVQG----RLEGDSVTGLYTLES---------KDFE----
UniRef90_B9V482_80_298     CLLVVVIPEHQLASHD-G------GTVSVKYKF---------THPGEQGI
UniRef90_B8XTP9_157_328    LIGLFLVPEFTRPGPE-NLEWRDLTEM---KRI-LND-----TNIYNSQT
UniRef90_F8V2V4_80_316     CLLVVCVPEAEMGCTN--------VENAPAYGD---------LCGGETAK
UniRef90_A1E4A2_131_303    ALLVVAIPEHQLGKYN--------TGTSASFDD---------VMKGKSGG
UniRef90_Q8V5Z9_80_307     ALLVVAIPEWTQGRGL-TSS----TNTYMEYSQ---------TNPGKNGG
UniRef90_G8IJE1_80_316     TLIVAAIPEHQLASAE-K------GNVNTGYNY---------THPGETGR
UniRef90_P12916_80_317     TLLVAMIPEHQLASAK-N------GSVTAGYNL---------THPGEAGR
UniRef90_P03303_80_315     CLLVVVIPEHQLASHE-G------GNVSVKYTF---------THPGERGI
UniRef90_B8XTQ4_69_329     LIGLFLVPECCIPASL-NMDWIDLKTQ---LPL-LTS-----SSHYQGLG
UniRef90_Q9YLJ1_78_316     CLLVVCVPEAEMGCAD--------VTSVVTALN---------LINGEDAH
UniRef90_B9V466_80_318     TLLVVMIPEHQLANAN-S------AKAGAGYNY---------THPGERGR
UniRef90_Q05ED6_131_323    CLLVVCVPEAEMGCAD--------VERTVVEES---------LSKSESPC
UniRef90_B5BSU7_131_316    CLLVACVPEAEMGCAD--------TTTVVTQDS---------LSNGDVPM
UniRef90_A5GZF1_80_316     TLIVAMIPEHQLASAT-T------GNATAGYNL---------THPGEGGR
UniRef90_B9V453_80_313     TLLVVAIPEHQLAYIG-T------GNVTVGYKH---------THPGETGR
UniRef90_Q05EE3_80_323     CLLVVCVPEAEMGCAK--------TDYTVTEDA---------LSKGEEPC
UniRef90_A5GZE7_80_317     TLLVVMIPEHQLASAH-T------GNVTAGYNF---------THPGEKGR
UniRef90_B9V485_80_315     CLLVVVIPEHQLASHQ-G------GNVSVKYTL---------THPGEAGI
UniRef90_G8IJH5_80_318     TLIVAMIPEHQLAAAE-K------GNVTAGYNL---------THPGERGR
UniRef90_B9V435_80_318     TLIVAMIPEHQLAAAE-R------GNVTAGYNF---------THPGEPGR
UniRef90_Q8V398_80_292     ALLVFLAPDFTRGAPT-TTTTG--TIPNVSYSD---------TMPGEDGT
UniRef90_Q82081_159_315    CLLVVVIPEHQLASHE-G------GTVSVKYKY---------THPGDRGI
UniRef90_Q6W9F6_159_316    CLLVVCVPEAEMGCAT--------VTNTLNATA---------LCNGDTAM
UniRef90_Q03053_159_316    CLLVVCVPEAEMGCAT--------LANKPDQKS---------LSNGETAN
UniRef90_A5GZD6_131_315    CLLIVVIPEHQLASHD-G------GTVSVKYKF---------THPGDQGI
UniRef90_Q2YEN8_80_316     CLLVVCVPEAEMGCSQ--------TDKEVAAMN---------LTKGETAH
UniRef90_G8HH17_117_311    TLMIVAVPECQFPG----EPTAEFGTIPDTLRT---------EFWK----
UniRef90_A7KC08_80_331     TLIVAMIPEHQLASAT-T------GNVRAGYNL---------THPGESGR
UniRef90_Q6W9G1_131_314    CLLVVCVPEAEMGSAV--------PGKALLASD---------LTGAEEPM
UniRef90_G8IQ64_80_315     CLLVVVIPEHQLASHK-G------GNVSVKYKY---------THPGEGGI
UniRef90_I0IY47_80_323     TLFVAAIPEFMMASNA-AD-----GTGGITYER---------ANPGEAGG
UniRef90_G5DC75_80_317     TLLVVMIPEHQLAAAN-T------GNVAAGYNF---------THPGEKGR
UniRef90_Q05ED2_80_316     CLLVVCVPEAEMGCAM--------KDGKVSAES---------LTKGESAC
UniRef90_Q5VAK8_80_316     ALLVVCVPEAEMGCAT--------IDRTVNATA---------LCEGDKAK
UniRef90_A7KC11_80_330     TLIVAAIPEHQLASAH-K------GNVNTGYNY---------THPGETGR
UniRef90_B5BSU6_131_316    CLLVVCVPEAEMGGRP--------TGQAFLNEE---------LTKGEVAC
UniRef90_Q05ED0_80_316     CLLVVCVPEAEMGNFN--------IQGALTAAD---------LTNGETAH
UniRef90_Q5VAL5_159_315    CLLVVCVPEAEMGSAT--------VGQAFEHTA---------LTNQDTPH
UniRef90_A4UHT9_129_315    CLLVAVVPEHQLAYIG-G------DNTRVKYKH---------THPGELGH
UniRef90_H8Y6P9_127_322    CLIVAVIPEHQLSYVG-A------ADVKVRYDH---------THPGEDGH
UniRef90_P29813_80_316     CLLVVCVPEAEMGCSQ--------VDGTVNEHG---------LSEGETAK
UniRef90_P32537_131_305    TLLIVAIPEHQIGKKG--------TGTSASFAE---------VMKGAEGG
UniRef90_G5D5E2_159_315    CLLVVVIPEHQLASHE-G------GNVSVKYKL---------THPGEGGI
UniRef90_Q9YLG5_80_319     CLLVVCVPEAEMGCTN--------KENTPLFEK---------LCGQDNAK
UniRef90_B8XTQ3_70_330     LIGLFLVPELVRSTGS-DLEWMVMTQK---LSL-YQPPAEEIAHSHYSTQ
UniRef90_A7KC13_88_321     TLLVVIIPEHQLASAE-G------GNVSVFYDQ---------THPGERGI
UniRef90_O41174_80_282     TLLVVAVPDHQLG-----------TTYQPEFDN---------VMPGKAGR
UniRef90_A5GZF9_80_318     TLIVAMIPEHQLASAR-H------GNLTAGYNF---------THPGENGR
UniRef90_G8IJE5_80_295     TLLVAMIPEHQLATAV-G------GSVSAGYNY---------THPGEKGR
UniRef90_Q9QCF0_72_316     CLLVVCVPEAEMGCAT--------VENEVSAAA---------LSSGETAK
UniRef90_G5DC71_157_324    CLLVAVVPEHQLAYIG-G------DNTQVNYEH---------THPGERGH
```

```
UniRef90_A8S330_157_325   LLLVAIVPEHQLAYIG-G------TNANVGYNH---------THPGEGGH
UniRef90_Q9WN78_80_316    CLLVVCVPEAEMGAAT--------TDHAMNHTK---------LSNIGQAM
UniRef90_A0MHB7_86_316    CLLVAVVPEHQLAYIG-V------D-AQVSYKH---------THPGERGH
UniRef90_O89280_159_323   CLLVVCVPEAEMGCSN--------LNNAPLAAD---------LSAGEVAR
UniRef90_G8IJG2_80_318    TLIVAAIPEHQLAFIS-K------GNVTVGYNY---------THPGEQGR
UniRef90_F6M9Y3_80_316    TLVIAAIPEHQLAYIG-S------GNVSVGYKQ---------THPGENGR
UniRef90_A5GZE0_80_317    TLLIATIPEHQLATAV-G------GSVSAGYNY---------THPGEKGR
UniRef90_Q7T7N9_80_323    TLFVAAIPEYMMASNS-GT-----NTGGIIYEF---------ANPGEAGG
UniRef90_A7YF31_159_315   CVLVVCVPEAEMGCAD--------IDSTFEHTA---------LTKQELPY
UniRef90_H9X8D4_80_317    TLLVAMIPEHQLASAK-H------GSVTVGYKL---------THPGEAGR
UniRef90_G9K146_80_316    TLIVAMIPEHQLASAT-T------GNATAGYNL---------THPGETGR
UniRef90_G9K138_159_330   TLIVAMIPEHQLASAT-Y------GNVAAGYKL---------THPGETGR
UniRef90_G0YPI0_80_292    ALLVAMVPEFVIASKS-ERSKPNDPAQYPAFSV---------TNPGKNGR
UniRef90_O91734_159_316   CILVVCVPEAEMGSAQ--------TSGVVNYEH---------ISKGEIAS
UniRef90_B9V473_80_315    TLVVAAIPEHQLAYIG-S------GNVSVGYRH---------THPGEQGR
UniRef90_Q9WHH4_131_316   CLLVVCVPEAEMGCTQ--------HDKTVNQEH---------LSQGERAM
UniRef90_Q2L970_80_316    CLLVVCVPEAEMGAAK--------INEKINREH---------LSKGEVAN
UniRef90_Q80GI2_159_316   CLLVVCVPEAEMGSST--------PGQAFTVEE---------LTKGEEAM
UniRef90_Q05ED1_131_316   CILVVCVPEAEMGCSK--------VDDTVSASE---------LSKGERAC
UniRef90_B9V440_80_317    TLIVAAIPEHQLASAT-K------GNVTAGYNY---------THPGEMGR
UniRef90_G1CSA8_80_313    TLLVACLPEYVNARAS-TNDTNA-TAPHPPYAV---------TQPGPNGK
UniRef90_B1PZZ4_80_316    CLMVVCVPEAEMGCSQ--------IDGTVNEHS---------LSEGETAK
UniRef90_B9V457_80_316    TLVVAAIPEHQLAFIG-K------GNVSVGYRH---------THPGENGR
UniRef90_D8VCT6_80_323    ALFVAAIPEFMMASNS-AD-----QTGGITYEK---------ANPGEAGG
UniRef90_G8IRN4_129_324   CLLVAVVPEHQLAYIG-G------SNAQVSYEH---------THPGERGH
UniRef90_Q80GI1_159_316   CLLVVCVPEAEMGSSQ--------VGQNFPASA---------LTKGERAS
UniRef90_I1XV41_80_323    TLFVAAIPEFMMASNS-AD-----STGGITYEK---------ANPGEAGG
UniRef90_P21404_159_316   CLLVVCVPEAEMGGAV--------VGQAFSATA---------MANGDKAY
UniRef90_P23008_135_293   TLLVAMIPEHQLASAK-H------GSVTAGYKL---------THPGEAGR
UniRef90_A5GZE2_80_315    TLIVAMIPEHQLASAE-K------GNVTAGYKL---------THPGEAGR
UniRef90_Q6W9F4_159_316   CLLVVCVPEAEMGSHT--------PGTAFASDA---------VSKGEVAL
UniRef90_B3SRP0_80_317    TLLVAAIPEYVVARAS-NEQDPN-TAPHPNYNV---------TQPGPGGK
UniRef90_Q6JKS0_159_312   ALLVAAVPEMVLGANS-SDPKPN-TAKHPEFAT---------TMPGANGG
UniRef90_G8IJG6_157_325   CLLVVVVPEHQLAYIG-G------TKASVSYNH---------THPGESGH
UniRef90_D4AH62_131_316   CLLVVCVPEAEMGCSN--------VDRVVTASA---------LTSGERAS
UniRef90_A5GZD9_129_321   CLLVVVIPEHQLASIQ-G------GSVSVYYEK---------THPGEKGI
UniRef90_Q66331_80_318    CLLVVCVPEAEMGCAT--------LDNTPSSAE---------LLGGDTAK
UniRef90_Q6W9E8_159_323   CLMVVCVPEAEMGCAD--------ITRVVNAAA---------LSGGELAK
UniRef90_P04936_159_330   TLIVALIPEHQIASAL-H------GNVNVGYNY---------THPGETGR
UniRef90_A7KC07_80_326    TLMVVAIPEHQLAYIG-T------GNVTVGYKH---------THPGESGR
UniRef90_Q66806_80_316    CLLVVCVPEAEMGSAQ--------LDKTLNHTK---------LSNTEHAS
UniRef90_A5GZF5_80_318    TLIVVAIPEHQLAYIG-G------GNVTVGYNH---------THPGESGR
UniRef90_Q05ED4_159_315   CLLVVCVPEAEMGCAD--------VQGVVTAEE---------LTKGEVAS
UniRef90_G8IRN3_129_329   LLLVAVVPEHQLAYVG-G------TNANVGYRH---------THPGETGH
UniRef90_Q82122_80_315    TLLVVMIPEHQLATVN-K------GNVNAGYKY---------THPGEAGR
UniRef90_Q8V601_80_317    TLLVAAIPEYVVARAS-DDAKPN-TAPHPNYAV---------TQPGPGGE
UniRef90_B9V433_129_314   CLLVVVIPEHQLASAE-G------GNVSVLYDK---------THPGEKGI
UniRef90_G5EN04_80_318    ALLVAAIPEFVVAASS-PATKPNGQGLYPDFAH---------TNPGKNGQ
UniRef90_Q8V613_159_316   CLLVVCVPEAEMGCSN--------VNNQPSFEQ---------LANGDTAH
UniRef90_Q6W9F9_80_316    CLLVVCVPEAEMGCAK--------TDGALVATS---------LTNGEMAH
UniRef90_H9C6N7_159_315   TLVVAAIPEHQLAYIG-S------GNVSVGYKH---------THPGETGR
UniRef90_B9V434_80_314    TLLVVAIPEHQLSYLG-T------GNVTVGYRH---------THPGENGR
UniRef90_A5GZF6_80_318    TLIVAAIPEHQLAFIK-S------GNVTVGYKY---------THPGEQGR
UniRef90_A5GZF2_80_318    LLIVAAIPEHQLASAV-K------GNVSVGYKH---------THPGEQGR
UniRef90_C7DUC8_80_317    TLLILMIPEHQLANAN-A------TNADAGYNY---------THPGERGR
UniRef90_G5CZJ4_127_322   CLMVAVIPEHQMAYIG-A------GGTNVRYDQ---------THPGEAGH
UniRef90_B9V477_80_318    TLIVVAIPEHQLAYVG-G------GNVTVGYNH---------THPGETGR
UniRef90_G8IJH3_88_314    TLLVVVIPEHQLASAK-G------GNVSVYYDK---------THPGENGI
UniRef90_Q8UY33_159_317   TLLVVAIPEFTVARSD-TSTNPA-TAKHPSYAA---------AQPGKNGK
UniRef90_P07210_80_318    LLIVAAIPEHQLASAT-S------GNVSVGYNH---------THPGEQGR
UniRef90_Q66577_131_323   RPIVVCVPEAEMGCSK--------VDGVVNAQG---------LSKGETPI
UniRef90_G8IJF0_127_318   AIIVAVVPEHQLAYIG-G------TKANVSYGH---------THPGENGH
UniRef90_E7CRL8_159_323   ALGVFAIPEYCLAGDS-D------KQRYTSYAN---------ANPGERGG
UniRef90_Q995Z1_80_314    ALLVVCVPEAQMGSAK--------VKEAFTHEA---------LSKGEIAK
UniRef90_C6ZEZ9_129_315   CLLIMVVPEHQLAYIG-A------EGVKVRYEH---------THPGEKGH
UniRef90_Q5G9Z5_159_312   ALLVVAIPEYVQGRSS-NSTTPA-SAPHPTYQA---------VQPGKNGK
```

```
2WZR2                     --------------------------------CKLDDREKY------QLT
UniRef90_F1AW55_285_503   --------------------------------CKLTDREKY------QLT
UniRef90_Q6PMU0_285_503   --------------------------------CKLEDREKY------QLT
```

```
UniRef90_Q8V0G8_86_304   --------------------------------CKLEQREKY------QLT
UniRef90_Q6PMU2_285_503  --------------------------------CKLDEREKY------QLT
UniRef90_Q6PMT8_285_503  --------------------------------CSLRDREEF------QLS
UniRef90_A2I8Z5_287_504  --------------------------------CSITKREQY------QLT
UniRef90_Q1L754_86_304   --------------------------------CSPREREEF------QLT
UniRef90_B8Y0J7_285_503  --------------------------------CSLKSXEEF------QLT
UniRef90_Q1L757_86_304   --------------------------------CSLREREEF------QLG
UniRef90_P49303_287_504  --------------------------------KELTPREKY------QLT
UniRef90_A6XP89_287_504  --------------------------------KTLDTRQKY------QLT
UniRef90_Q6PMT3_285_502  --------------------------------HSLDKRDVS------QLT
UniRef90_Q1L752_86_304   --------------------------------SSIRDREEF------QPT
UniRef90_Q1L748_86_303   --------------------------------KSLDKRCVS------QLT
UniRef90_P15072_287_504  --------------------------------GDISDREKY------QLT
UniRef90_Q8JVD0_86_303   --------------------------------HTLDKRDVS------QLT
UniRef90_H9AY35_273_496  --------------------------------NFKDTSDSQ-PPDYRQYT
UniRef90_B0Z3W9_300_527  --------------------------------VKENTNGSTYNPTFAQLT
UniRef90_Q91LX8_289_537  LPGEWKWQSYDALP------------------------RHLPPQ------QLT
UniRef90_Q66776_291_513  TPGTWVWNTYEAFP------------------------PGFPPQ------QIT
UniRef90_Q100Y4_72_320   LPGAWMWQTYDARP------------------------STLPPQ------QLT
UniRef90_Q2XP17_290_516  EEPAYTYQ---------------------------------------QLS
UniRef90_P12296_138_384  FAMDNRWSKDN-LP---NGTRTQTNRKG---PFAMDHQNFW------QWT
UniRef90_Q02472_148_405  FLMDTTWQEPQAAP---TGFRYDGKNGF----FTLNHQNYW------QWT
UniRef90_A4ZKN2_144_403  FKVDTTWQTGS--L---RGHSYEDKSTQTLRPLALNHQNYW------QWT
UniRef90_C3U5A3_174_435  FTMDTTWQSGTKCL---RGHSFESKTNQSLRPLSLNHQNTW------QWT
UniRef90_P08544_148_405  FTMDTEWRSPQGAP---TGYRYDSRTGF----FATNHQNQW------QWT
UniRef90_E3TMG9_144_405  FKVDANWQKHTQIL---TGHAYVNTTTKVNVPLALNHQNFW------QWT
UniRef90_G9FTJ7_144_402  FQMDTQWQSGK--L---LGHSYESTTLQGLRPLALNHQNYW------QWT
UniRef90_B3VTU7_149_406  FTMDSSWQTPQQNP---VGFRYDGRTGY----FALNHQNYW------QWM
UniRef90_Q155Z9_151_421  VEMSDDYRTGKNMPFQSLGTYYRPPNWT----WGPNFINPY------QVT
UniRef90_G1C9J1_14_204   ------------------------------------------QYPIHQLT
UniRef90_I1YLT5_69_326   D----ITDNLDEKN-SFDLA----------------------DITPEQMF
UniRef90_G0Y2E2_141_365  ------------------------------------------DYPRFQLT
UniRef90_G0Y2E0_150_361  ------------------------------------------NYPKHQLT
UniRef90_Q8B552_195_378  DFQYYETKEFCGTEYPTE-----------------------------QLT
UniRef90_B5A1N0_195_378  GWAYREGEKYCGKQYPTE-----------------------------QLT
UniRef90_Q2LKZ0_129_301  AY--Q-------------FP----------------FTFED-GTALGNAL
UniRef90_Q65481_129_301  EF--Q-------------FP----------------FTLED-GSALGNAL
UniRef90_G1DFA4_229_430  FTMSTEWE---GLP---------------------TDMRPE------SFF
UniRef90_H9LG14_129_301  EF--N-------------FN----------------FTLED-GSALGNAL
UniRef90_H6VLP2_125_335  ------------------------------------------DYPLAQLT
UniRef90_B9VIN1_70_329   A----LVGKIDDKN-TFDLA----------------------STTPEQFF
UniRef90_G1JYY8_171_367  ------------------------------------------GLPLAQLT
UniRef90_Q8QUZ6_80_281   NL--E-------------YA----------------YNFED-GTSIANSL
UniRef90_I1YLT7_70_335   M----VLPDLDTKVVLLIMA----------------------DFTPEQFL
UniRef90_F5B9S5_80_301   DI--T-------------DA----------------FNLED-GTSLANSL
UniRef90_G0Y2E4_163_340  ------------------------------------------EIPVPQLT
UniRef90_B8XTQ1_73_321   Y----AGDRAFDTEGSFDMG----------------------DFTPEQFM
UniRef90_H9C6N6_131_322  NLSDA-VA----TGGASHDP----------------VYSMN-GTLVGNLL
UniRef90_I1YLT4_70_334   Y----GAGTPWNEAGSFDLA----------------------DFTPEQFL
UniRef90_B8XTQ0_73_332   YGPGTNSTRKFDDENSFDMG----------------------DMTPEQFF
UniRef90_B9V442_80_329   DVG-I-TRVEDLLKQPSDDS----------------WLNFD-GTLLGNIT
UniRef90_D2IW00_80_316   TL-ND-RENNN-SQQPTDES----------------WLNCD-GTLLGNIT
UniRef90_Q2LL04_129_301  TF--Q-------------EP----------------FWLED-GTCLGNAL
UniRef90_I1YLT6_69_335   Y----TSPSTLIEDYSFDLA----------------------DFTPEQMM
UniRef90_G5DC73_80_330   EVKNT-TR-EHLQQQPTEEH----------------WLNFD-GTLLGNIT
UniRef90_A5GZE4_80_318   DVGIT-VR-EHPNKQPSDDN----------------WLNFD-GTLLGNIT
UniRef90_D9D8F3_70_328   Y----AGDRALDTDGSFDMA----------------------DFSPEQFM
UniRef90_G0ZNT5_127_322  TIEET-T--IRNRFNPDENP----------------FYLCN-GTLFGNIQ
UniRef90_B9V459_80_317   VVGQA-RV-QTNDNQPSDDN----------------WLNFD-GTFLGNLL
UniRef90_A8S322_129_323  VIGSN-V--RRDNKQPDEDP----------------FFNCN-GTLLGNIT
UniRef90_C7DUC7_127_322  RIGAR-T--DRGDHNPDENP----------------FYLCN-GTLFGNIQ
UniRef90_G8IJE2_80_317   EVRTN-RT-EYLGQQPTEEY----------------WLNFD-GTLLGNIT
UniRef90_Q2LKZ8_129_301  TF--Q-------------EP----------------FWLED-GTSLGNAL
UniRef90_B8XTP8_70_327   Y-----ANFPFDAKHSFDYS----------------------DITPEQFM
UniRef90_H2E685_80_301   TI--T-------------EP----------------FDMDD-GTSLANAL
UniRef90_Q5YJ95_199_371  GFAYRDGVNYQGNNYPTE-----------------------------QLT
UniRef90_Q9IM09_80_305   TMTGQ-------------EA----------------YCLHN-GTNIHSSL
UniRef90_A5GZE9_80_317   DVGQD-R--RSNDKQPSDDN----------------WLNFD-GTLLGNLL
UniRef90_Q66790_80_316   ELTAGAAP---AEKGKVQTA----------------VCNATMGVAVGNLT
UniRef90_A5GZD8_131_315  DLDSV-AE----TGGASHDP----------------VYSMN-GTLIGNLL
UniRef90_P12915_129_301  TF--Q-------------EP----------------FWLED-GTSLGNSL
UniRef90_Q5XLP5_80_317   DVGQQ-R--TNNEKQPSDDN----------------WLNFD-GTLLGNLL
UniRef90_G0Z2U8_195_360  EFVYRNNANYQGNNYPTE-----------------------------QLT
```

```
UniRef90_A1E4A3_131_303   TF--N-------------HP----------------YVLDD-GTSLACAT
UniRef90_G0Y2E3_141_369   ------------------------------------------DYPAPQLT
UniRef90_B9V482_80_298    DLGTS-GE----RTGPAKDA----------------VYNMD-GTLIGNLT
UniRef90_B8XTP9_157_328   L-PGSFS---FDSDHSFDLG----------------------DFTPEQFL
UniRef90_F8V2V4_80_316    SFEQNAAT---GET-AVQTA----------------VCNAGMGVGVGNLT
UniRef90_A1E4A2_131_303   VF--N-------------HP----------------YVLDD-GTSLACSL
UniRef90_Q8V5Z9_80_307    TF--N-------------HP----------------YLLDN-GTAVSMAL
UniRef90_G8IJE1_80_316    DVGKD--R-ESTRYQPSDDN----------------WLNFD-GTLLGNIT
UniRef90_P12916_80_317    VVGQQ-R--DANLRQPSDDS----------------WLNFD-GTLLGNLL
UniRef90_P03303_80_315    DLSSA-NE----VGGPVKDV----------------IYNMN-GTLLGNLL
UniRef90_B8XTQ4_69_329    L---STGQGTISEKGSIDAA----------------------GTIPQQLF
UniRef90_Q9YLJ1_78_316    TFSPSEAT---AEAGKVQTA----------------VCNAGMGVAVGNLT
UniRef90_B9V466_80_318    QVGDP-RE-NITAAHPSDDN----------------WLNFD-GTMLGNVL
UniRef90_Q05ED6_131_323   EFTPSSVT---GEN-AVQTA----------------VCNAAMGVAVGNLT
UniRef90_B5BSU7_131_316   KFSNQKET---SAN-KVQNA----------------VWNAGMGVGVGNLT
UniRef90_A5GZF1_80_316    DVSLS-NR-ADLSKQPSDDA----------------WLNFD-GTLLGNIT
UniRef90_B9V453_80_313    VIATT-TDKQ--TRQPSFDS----------------WLNCN-GTLLGNAL
UniRef90_Q05EE3_80_323    LFTESESS---ETH-GVQTA----------------VHNAAMGIAAGNLT
UniRef90_A5GZE7_80_317    TVGDR-R--DHHDKQPSDDN----------------WLNFD-GTSLGNAL
UniRef90_B9V485_80_315    DLSKA-EV----DKGPSNNP----------------VYSMN-GTLLGNLL
UniRef90_G8IJH5_80_318    TIGIP-DR-LHPNKQPSDDN----------------WLNFD-GTLLGNIT
UniRef90_B9V435_80_318    EVGKP-NR-QYTSKQPSDDN----------------WLNFD-GTLLGNIT
UniRef90_Q8V398_80_292    TF--R-------------NP----------------LFLDN-GTMLQQAT
UniRef90_Q82081_159_315   DLDTV-EV----AGGPTSDA----------------IYNMD-GTLLGNLL
UniRef90_Q6W9F6_159_316   NFTKTRAN---TTN-GVQTA----------------IHNAGMGVATGNLT
UniRef90_Q03053_159_316   TFDSQNTT---GQT-AVQAN----------------VINAGMGVGVGNLT
UniRef90_A5GZD6_131_315   DLSTA-EE----TNGPVKNP----------------VYNMD-GTLLGNLL
UniRef90_Q2YEN8_80_316    KFEPTTTT---GEH-TVQSI----------------VCNAGMGVGVGNLT
UniRef90_G8HH17_117_311   ------------------------------------------QYPRSQLT
UniRef90_A7KC08_80_331    NVDPI-TR-DNPTKQPSDDS----------------WLNFD-GTLLGNIT
UniRef90_Q6W9G1_131_314   EFKNTDTT------TGVQAN----------------VINAGMGVGVGNLT
UniRef90_G8IQ64_80_315    DLSTP-QD----EEGPVKDP----------------VYSMN-GTLLGNLL
UniRef90_I0IY47_80_323    KFQKN-------------FTPS-STAPAKNSFAPVDWLLGC-GVMAGNIT
UniRef90_G5DC75_80_317    EVGSP-R--ETHPNHPSDDS----------------WLNFD-GTLLGNAL
UniRef90_Q05ED2_80_316    TFSEAEAT---SENGKVQNA----------------VCNAAMGVGVGNLT
UniRef90_Q5VAK8_80_316    EFTAGAVS---SQN-GVQTA----------------VYNAAMGVAVGNLT
UniRef90_A7KC11_80_330    DVGTE-VR-DTANYQPSDDA----------------WLNFD-GTLLGNIT
UniRef90_B5BSU6_131_316   VFTANKEE---GTSKSVQAA----------------VCNAGMGVGVGNLT
UniRef90_Q05ED0_80_316    KFTTDPAQ---GTGNGVQTA----------------VCNAAMGVGVGNLT
UniRef90_Q5VAL5_159_315   TFTEGAST---GR--AVQTA----------------VCNAGMGVGVGNLT
UniRef90_A4UHT9_129_315   KIGSN-S--ERGDNQPDENP----------------FFNCN-GTLLGNLT
UniRef90_H8Y6P9_127_322   TFKAP-T--ERGQQNPDENP----------------FYLCN-GTLLGNIT
UniRef90_P29813_80_316    KFSSTSTN---GTN-TVQTI----------------VTNAGMGVGVGNLT
UniRef90_P32537_131_305   VF--E-------------QP----------------YLLDD-GTSLACAL
UniRef90_G5D5E2_159_315   DLSTN-VE----ADGPVKDP----------------VYNMN-GTLLGNLL
UniRef90_Q9YLG5_80_319    EFTREGPTISKGAT-DVQTA----------------VCNAGMGVGVGNLT
UniRef90_B8XTQ3_70_330    T----ITPN---KNVSFDLA----------------------DMTAEQMM
UniRef90_A7KC13_88_321    DLGTV-IE----K-GPMTDP----------------IYNMD-GTLLGNVL
UniRef90_O41174_80_282    EV--K-------------YP----------------YNFED-GTSLANSL
UniRef90_A5GZF9_80_318    NVGQL-RT-EYLDKQPSDDN----------------WLNFD-GTLLGNIT
UniRef90_G8IJE5_80_295    DVGKL-NR-ESNQRQPSDDN----------------WLNFD-GTLLGNLT
UniRef90_Q9QCF0_72_316    YFAKTGAT---GTH-TVQSI----------------VTNAGMGVGVGNLT
UniRef90_G5DC71_157_324   TIGSN-S--DRGNNQPDENP----------------FFNCN-GTLLGNLT
UniRef90_A8S330_157_325   EIGKN-T--ERNDKQPDEDP----------------LFNCN-GTLLGNLT
UniRef90_Q9WN78_80_316    EFSAGKST---DQT-GPQTA----------------VHNAGMGVAVGNLT
UniRef90_A0MHB7_86_316    EIGRN-T--NRDDNQPDENP----------------FFNCN-GTLLGNLT
UniRef90_O89280_159_323   QFTVEPAN---GQN-QVQTA----------------VHNAAMGVAVGNLT
UniRef90_G8IJG2_80_318    EIGTF-GPSSTLDRQPSDEN----------------WLNCD-GTLLGNIT
UniRef90_F6M9Y3_80_316    TIDDT-RVNTM-SNQPTDES----------------WLNCD-GTLLGNIT
UniRef90_A5GZE0_80_317    DVGKM-DR-ERNDRQPSDDS----------------WLNFD-GTLLGNLT
UniRef90_Q7T7N9_80_323    RFSST-------------FTPD-TQAPGKNKFAPLDWLLGC-GVMAGNIT
UniRef90_A7YF31_159_315   EFTANPVT---GK--VVQTA----------------VCNAGMGVGVGNLT
UniRef90_H9X8D4_80_317    DVSQE-R--DTSLKQPSDDS----------------WLNFD-GTLLGNLL
UniRef90_G9K146_80_316    DVSPK-VR-ENPDKQPSDDA----------------WLNFD-GTLLGNIT
UniRef90_G9K138_159_330   DVSLS-RT-ENRDKQPSDDN----------------WLNFD-GTLLGNIT
UniRef90_G0YPI0_80_292    EF--T-------------DP----------------YVLDA-GIPLSQAL
UniRef90_O91734_159_316   RFTTTTT----AEDHGVQAA----------------VWNAGMGVGVGNLT
UniRef90_B9V473_80_315    VI-GT-RENRN-TNQPTDES----------------WLNCD-GTLLGNIT
UniRef90_Q9WHH4_131_316   SFKT-ETT---GEEHSVQAA----------------VWNAGLGVGAGNLT
UniRef90_Q2L970_80_316    TFTGTKSS---NTN-DVQQA----------------VFNAGMGVAVGNLT
UniRef90_Q80GI2_159_316   EFTAGNHQ---GTTASVQKA----------------VCNAGMGVGVGNLT
UniRef90_Q05ED1_131_316   AFTSGKAT---AAAGTIQTA----------------VFNAGMGVGVGNLT
UniRef90_B9V440_80_317    DVGKI-GR-ENNGKQPSDDN----------------WLNFD-GTLLGNIT
```

```
UniRef90_G1CSA8_80_313     EL--T-------------FP----------------YVLDA-GIPINQLL
UniRef90_B1PZZ4_80_316     KFAATSTN---GTN-TVQSI----------------VTNAGMGVGVGNLT
UniRef90_B9V457_80_316     TLNGS-RVDGN-SKQPTEES----------------WLNCD-GTLLGNIT
UniRef90_D8VCT6_80_323     KFQAS-------------FTPS-AEDGKKNTFAPVDWLLGC-GVMAGNIT
UniRef90_G8IRN4_129_324    EIGSN-T--NRDRNQPDEDP----------------FFNCN-GTLLGNLT
UniRef90_Q80GI1_159_316    VFTSGEHQ---STEKGVQTA----------------VCNAGMGVGVGNLT
UniRef90_I1XV41_80_323     KFQPT-------------FTPS-SEDGKKNTFAPVDWLLGC-GVMAGNVT
UniRef90_P21404_159_316    EFTSATQS---DQT-KVQTA----------------IHNAGMGVGVGNLT
UniRef90_P23008_135_293    DVSQE-R--DASLRQPSDDS----------------WLNFD-GTLLGNLL
UniRef90_A5GZE2_80_315     DVSIT-NR-DHVSRQPSDEN----------------WLNFD-GTLLGNIT
UniRef90_Q6W9F4_159_316    QFTETGSG---GTTAGVQTA----------------VCNAGMGVAVGNLT
UniRef90_B3SRP0_80_317     EI--A-------------FP----------------YLLDN-GTALSQAL
UniRef90_Q6JKS0_159_312    TF--E-------------NP----------------YIMDM-GVPLSQAL
UniRef90_G8IJG6_157_325    EIGKNTT--NRGNHEPDENP----------------FFSCN-GTLLGNLT
UniRef90_D4AH62_131_316    TFQATINS---NQN-DVQNV----------------VHNAAMGVGVGNLT
UniRef90_A5GZD9_129_321    DLNES-DG----D-GPMLDP----------------LYMMD-GTLIGNSL
UniRef90_Q66331_80_318     EFADKPVA--SGSNKLVQRV----------------VYNAGMGVGVGNLT
UniRef90_Q6W9E8_159_323    EFKEAPTT---GQN-EVQAA----------------AHNAGMGVAVGNLT
UniRef90_P04936_159_330    EVKAE-TR-LNPDLQPTEEY----------------WLNFD-GTLLGNIT
UniRef90_A7KC07_80_326     IIANN-ADNN--TRQPSDDN----------------WLNCD-GTLMGNLP
UniRef90_Q66806_80_316     TFGSMNSN---EAG-AVQNV----------------VHNAGMGVGVGNLT
UniRef90_A5GZF5_80_318     RIGGF-DGTER-NRQPSDEN----------------WLNCN-GTLLGNLT
UniRef90_Q05ED4_159_315    VFSNTNTT---GQG-KVQNQ----------------VCNAGMGVGVGNLT
UniRef90_G8IRN3_129_329    TITGNTA--NRSSGEPDDDP----------------FFNCN-GTLLGNLT
UniRef90_Q82122_80_315     EVGTQ----VENEKQPSDDN----------------WLNFD-GTLLGNLL
UniRef90_Q8V601_80_317     EL--A-------------FP----------------YLLDN-GTALSQAP
UniRef90_B9V433_129_314    DLSEA-DS----T-GPMKDP----------------LYMMD-GTLIGNSL
UniRef90_G5EN04_80_318     EF--R-------------DP----------------YVLDA-GIPLSQAL
UniRef90_Q8V613_159_316    VFTEDAAT---GEQ-GVQTA----------------VCNAAMGVGVGNLT
UniRef90_Q6W9F9_80_316     EFTESAAP---STQGNVQTA----------------VCNAAMGVGVGNLT
UniRef90_H9C6N7_159_315    II-GS-RDNNM-TGQPTDES----------------WLNCD-GTLLGNIT
UniRef90_B9V434_80_314     EIAEV-ANRN--ERQPSYDS----------------WLNCN-GTLLGNAL
UniRef90_A5GZF6_80_318     EIGTF-GENSVLDRQPSDEN----------------WLNCD-GTLLGNIT
UniRef90_A5GZF2_80_318     EISSE-RR-TDDDRRPSDDS----------------WLNFD-GTLLGNLP
UniRef90_C7DUC8_80_317     EVGSD-R--VAHANLPSDDN----------------WLNFD-GTLLGNAL
UniRef90_G5CZJ4_127_322    TIMAS-K--QWGEHNPDENP----------------FYNLN-GTLFGNIQ
UniRef90_B9V477_80_318     RIGGY-ENVPR-NRQPSDEN----------------WLNCN-GTLLGNLT
UniRef90_G8IJH3_88_314     DLSVV-DE----L-GPKTDP----------------VYNMD-GTLLGNAL
UniRef90_Q8UY33_159_317    EF--S-------------IP----------------YVLDS-GVPLSQAL
UniRef90_P07210_80_318     EVVPS-RT-SSDNKRPSDDS----------------WLNFD-GTLLGNLP
UniRef90_Q66577_131_323    EFERTSTT---AEVGVVQKA----------------VYNAGMGVGVGNLT
UniRef90_G8IJF0_127_318    EIRGPDSGHDRGNNNPDEDP----------------LFNCN-GTLLGNIT
UniRef90_E7CRL8_159_323    KFYSQ-------------FNKDNAVTSPKREFCPVDYLLGC-GVLLGNAF
UniRef90_Q995Z1_80_314     EF--TPSD---ATD-GVQTA----------------VHNAAMGVAVGNLT
UniRef90_C6ZEZ9_129_315    TLKGS-Q--DRSTNNPDDNP----------------FYMCN-GTLLGNGM
UniRef90_Q5G9Z5_159_312    EL--A-------------FP----------------YVLDA-GIPLSEAL
```

```
2WZR2                      LFPHQFLNPRTNTTAHIQVPYLGVDRHD-----------------QGTRH
UniRef90_F1AW55_285_503    LFPHQFLNPRTNTTAHIQVPYLGVDRHD-----------------QGVRH
UniRef90_Q6PMU0_285_503    LFPHQFLNPRTNTTAHIQVPYLGVDRHD-----------------QGMRH
UniRef90_Q8V0G8_86_304     LFPHQFLNPRTNTTAHIQVPYLGVDRHD-----------------QGHKH
UniRef90_Q6PMU2_285_503    LFPHQYINPRTNTTAHIQVPYLGVDRHD-----------------QGKRH
UniRef90_Q6PMT8_285_503    LYPHQFINPRTNTTAHIQVPYLGVNRHD-----------------QGKRH
UniRef90_A2I8Z5_287_504    LFPHQFINPRTNMTAHITVPYLGVNRYD-----------------QYKVH
UniRef90_Q1L754_86_304     LYPHQFINPRTNTTAHIQVPYLGVNRHD-----------------QGKRH
UniRef90_B8Y0J7_285_503    LYPHQFINPRTNTTAHIQVPYLGVNRHD-----------------QGKRH
UniRef90_Q1L757_86_304     LYPHQFINPRTNTTAHIQVPHLGVNRHD-----------------QGKRH
UniRef90_P49303_287_504    LFPHQFISPRTNMTAHIVVPYLGVNRYD-----------------QYKKH
UniRef90_A6XP89_287_504    LFPHQFINPRTNMTAHINVPFVGVNRYD-----------------QYVLH
UniRef90_Q6PMT3_285_502    LFPHQYINPRTNTTAHIVVPYVGVNRHD-----------------QVQMH
UniRef90_Q1L752_86_304     LYPHQFINPRTNTTAHIQVPYLGVNRHD-----------------QGKRH
UniRef90_Q1L748_86_303     LFPHQFINPRTNTTAHLVVPYIGVNRHD-----------------QVNQH
UniRef90_P15072_287_504    LYPHQFINPRTNMTAHITVPYVGVNRYD-----------------QYKQH
UniRef90_Q8JVD0_86_303     LFPHQHINPRTNTTAHIVVPYVGVNRHD-----------------QVKMH
UniRef90_H9AY35_273_496    VYPHQMINPRTNTTAHIRVPYVGAFSME-----------------DVNKH
UniRef90_B0Z3W9_300_527    LYPHQLINLRTNTTASIRVPYVGATDMD-----------------DHRLH
UniRef90_Q91LX8_289_537    LYPHQFLNLRTNTTVDIEVPYMNFVPAS-----------------APVMH
UniRef90_Q66776_291_513    LFPHQFLNLRTNTTVDLEVPYTNFAPSS-----------------SPTLH
UniRef90_Q100Y4_72_320     LFPHQFLNLRTNTTVDLEVPYMNCVPSS-----------------SPKMH
UniRef90_Q2XP17_290_516    VFPHQLLNLRTNSSVHLVMPYIGPGPTT-----------------NLTLH
```

```
UniRef90_P12296_138_384   LYPHQFLNLRTNTTVDLEVPYVNIAPTS-----------------SWTQH
UniRef90_Q02472_148_405   VYPHQILNLRTNTSVDLEVPYVNVAPTS-----------------SWTQH
UniRef90_A4ZKN2_144_403   VYPHQILNLRTNTTCDLEVPYVNICPTS-----------------SWTQH
UniRef90_C3U5A3_174_435   IYPHQILNLRTNTTCDIEVPYVNICPTS-----------------SWTQH
UniRef90_P08544_148_405   VYPHQILNLRTNTTVDLEVPYVNVAPSS-----------------SWTQH
UniRef90_E3TMG9_144_405   TYPHQILNLRTNTTCDLEVPYVNVCPTS-----------------SWTQH
UniRef90_G9FTJ7_144_402   VYPHQILNLRTNTTCDLEVPYVNICPTS-----------------SWTQH
UniRef90_B3VTU7_149_406   VYPHQILNLRTNTSVDLEVPFTNIAPTS-----------------SWTQH
UniRef90_Q155Z9_151_421   VFPHQILNARTSTSVDINVPYIGETPTQ-----------------SSETQ
UniRef90_G1C9J1_14_204    LFPHQFINLRTNNSATLVLPYVNPCPME-----------------NAASH
UniRef90_I1YLT5_69_326    LYPHQLINPKETTIATVRVPYINAAPQN-----------------DPNVH
UniRef90_G0Y2E2_141_365   LYPHQFINLRTNNSATLILPYVAASPAE-----------------SPLTH
UniRef90_G0Y2E0_150_361   LYPHQLINLRTNNSATLILPYVSASPAE-----------------SPLTH
UniRef90_Q8B552_195_378   LFPHQFLNLRTNNSVTIVYPYTNCTPSG-----------------FGLSH
UniRef90_B5A1N0_195_378   LFPHQFINLRTNNSATIIYPYTNVTPAS-----------------FGLAH
UniRef90_Q2LKZ0_129_301   IYPHQWVNLRTNNSATLVLPYVNALPMD-----------------SGIRH
UniRef90_Q65481_129_301   IYPHQWINLRTNNSATLILPYVNALPMD-----------------SGIRH
UniRef90_G1DFA4_229_430   LYPHQLINCRTNTSVDLILPYLNFVPTS-----------------AYGYH
UniRef90_H9LG14_129_301   IYPHQWINLRTNNSATLILPYVNAIPMD-----------------SGIRH
UniRef90_H6VLP2_125_335   LFPHQLINLRTNNSATLILPYVNASPSE-----------------NPLSH
UniRef90_B9VIN1_70_329    LYPHQLINPKETTMATVSVPYVNVAPSS-----------------DPSVH
UniRef90_G1JYY8_171_367   VFPHQIINLRTNNSATILMPYVGPTPAT-----------------FGLTH
UniRef90_Q8QUZ6_80_281    IYPHQWINLRTNNSATLVLPYMNAVPMD-----------------SAIRH
UniRef90_I1YLT7_70_335    LYPHHFINPKDTNIATVRVPYISVAPTN-----------------DSTVH
UniRef90_F5B9S5_80_301    IYPHQWINLRTNNSATIVLPYINCIPMA-----------------SAIRH
UniRef90_G0Y2E4_163_340   LFPHQLINLRTNNSATIIYPYTNCTPAE-----------------NALTH
UniRef90_B8XTQ1_73_321    LFPHQLINPKDNNIATVRVPYINIAPTN-----------------DPTVH
UniRef90_H9C6N6_131_322   IFPHQFINLRTNNTATLIVPYINSVPMD-----------------SMTRH
UniRef90_I1YLT4_70_334    LYPHQFINPKDTNIATVRVPYISVAPTN-----------------DSTVH
UniRef90_B8XTQ0_73_332    LFPHQLINPKDTNVAIVRVPYVNIAPSN-----------------DTTVH
UniRef90_B9V442_80_329    IFPHQFINLRSNNSATIIVPYVNAVPMD-----------------SMPRH
UniRef90_D2IW00_80_316    IFPHQFINLRSNNSATLILPYVNAVPMD-----------------SMVRH
UniRef90_Q2LL04_129_301   IYPHQWINLRTNNSATLILPYVNAIPMD-----------------SAIRH
UniRef90_I1YLT6_69_335    LFPHQFINPKDTNIATVRVPYVNAAPTN-----------------DPTVH
UniRef90_G5DC73_80_330    IFPHQFINLRSNNSATIIVPYVNAVPMD-----------------SMRSH
UniRef90_A5GZE4_80_318    IFPHQFINLRSNNSATIIVPYVNAVPMD-----------------SMLRH
UniRef90_D9D8F3_70_328    LFPHQFINPKDTNIATVRVPYVNIAPTS-----------------DTTVH
UniRef90_G0ZNT5_127_322   IFPHQMINLRTNNSATIIVPYINCLPMD-----------------SMLRH
UniRef90_B9V459_80_317    IFPHQFINLRTNNSATIIVPYVNAVPMD-----------------SMLRH
UniRef90_A8S322_129_323   IFPHQLINLRTNNSSTIVVPYINCVPMD-----------------NMLRH
UniRef90_C7DUC7_127_322   IYPHQMINLRTNNSATIVVPYINCLPMD-----------------NMLRH
UniRef90_G8IJE2_80_317    IFPHQFINLRSNNSATIIVPYVNAVPMD-----------------SMRSH
UniRef90_Q2LKZ8_129_301   IYPHQWINLRTNNSATLILPYVNAIPMD-----------------SAIRH
UniRef90_B8XTP8_70_327    IFPHQLINPKDTNVATVRVPYINIAPTN-----------------DTTVH
UniRef90_H2E685_80_301    IYPHQWINVRTNNSATLVLPYINSIPMD-----------------SPIRH
UniRef90_Q5YJ95_199_371   IFPHQIINLRTNNSATIVYPYTNCTPAG-----------------FGLAH
UniRef90_Q9IM09_80_305    VFPHQFINLRTNNCATLVLPYVGATPLD-----------------TPIKH
UniRef90_A5GZE9_80_317    IFPHQFINLRSNNSATIIVPYVNAVPMD-----------------SMLRH
UniRef90_Q66790_80_316    IFPHQWINLRTNNCATIVMPYINSVPMD-----------------NMFRH
UniRef90_A5GZD8_131_315   IFPHQFINLRTNNTATIIVPYINSVPMD-----------------SMTRH
UniRef90_P12915_129_301   IYPHQWINLRTNNSATLILPYVNAIPMD-----------------SAIRH
UniRef90_Q5XLP5_80_317    IFPHQFINLRSNNSATIIVPYVNAVPMD-----------------SMLRH
UniRef90_G0Z2U8_195_360   VFPHQIINLRTNNSATIVYPYTNCTPAG-----------------FGLAH
UniRef90_A1E4A3_131_303   IFPHQWINLRTNNSATIVLPWMNAAPMD-----------------FPLRH
UniRef90_G0Y2E3_141_369   LYPHQMINLRTNNSATLILPYVSASPAE-----------------SPLTH
UniRef90_B9V482_80_298    IFPHQFINLRTNNTATIIVPYINSVPID-----------------SMTRH
UniRef90_B8XTP9_157_328   LFPHQLINPKDNNIATVRVPYVNIAPTS-----------------DTTVH
UniRef90_F8V2V4_80_316    IYPHQWINLRTNNSATIVMPYINSVPMD-----------------NMFRH
UniRef90_A1E4A2_131_303   IFPHQWINLRTNNSATIMLPWMNCAPMD-----------------FALRH
UniRef90_Q8V5Z9_80_307    IYPHQWINLRTNNCATIVLPYVNNVPSD-----------------CALKH
UniRef90_G8IJE1_80_316    IFPHQFINLRSNNSATLILPYVNAVPMD-----------------SMLRH
UniRef90_P12916_80_317    IFPHQFINLRSNNSATLIVPYVNAVPMD-----------------SMLRH
UniRef90_P03303_80_315    IFPHQFINLRTNNTATIVIPYINSVPID-----------------SMTRH
UniRef90_B8XTQ4_69_329    IYPHQLINPKDTNIASVEVPYVNCAPTS-----------------DPMIH
UniRef90_Q9YLJ1_78_316    IFPHQWINLRTNNCATIVMPYINSVPMD-----------------NMFRH
UniRef90_B9V466_80_318    IFPHQFINLRSNNSATIIVPYVNAVPMD-----------------SMLRH
UniRef90_Q05ED6_131_323   IFPHQWINLRTNNSATIVIPYINSVPMD-----------------NMFRH
UniRef90_B5BSU7_131_316   IYPHQWINLRTNNSATIVMPYINSVPMD-----------------TMYRH
UniRef90_A5GZF1_80_316    VYPHQLINLRTNNSATIIVPYVNAVPMD-----------------SMVRH
UniRef90_B9V453_80_313    IFPHQFINLRTNNAATLILPYVNATPMD-----------------SMLRH
UniRef90_Q05EE3_80_323    IFPHQWINLRTNNSATIIVPYINSVPMD-----------------NMYRH
UniRef90_A5GZE7_80_317    IFPHQFINLRSNNSATIIVPYVNAVPMD-----------------SMLRH
UniRef90_B9V485_80_315    IFPHQFINLRTNNTATIVVPYINSVPID-----------------SMTRH
```

```
UniRef90_G8IJH5_80_318    IFPHQFINLRSNNSATIIVPYVNAVPMD-----------------SMLSH
UniRef90_B9V435_80_318    IYPHQFINLRSNNSATIIVPYVNAVPMD-----------------SMLRH
UniRef90_Q8V398_80_292    VFPHQWINLRTNNSATIIVPYINNIPAD-----------------SPVRH
UniRef90_Q82081_159_315   IFPHQFINMRTNNTATIVVPYINSVPID-----------------SMTRH
UniRef90_Q6W9F6_159_316   IFPHQWINLRTNNSATIVMPYINSVPMD-----------------NMYRH
UniRef90_Q03053_159_316   IFPHQWINLRTNNSATIVMPYINSVPMD-----------------NMFRH
UniRef90_A5GZD6_131_315   IFPHQFINLRTNNTATIIVPYMNSVPMD-----------------SMTRH
UniRef90_Q2YEN8_80_316    IYPHQWINLRTNNCATIVMPYINSVPMD-----------------NMFRH
UniRef90_G8HH17_117_311   IFPHQFINLRTNNSSTLILPYVNATPAE-----------------NALTH
UniRef90_A7KC08_80_331    IFPHQFINLRSNNSATIIVPYVNAVPMD-----------------SMLRH
UniRef90_Q6W9G1_131_314   IFPHQWINLRTNNSATIVMPYINSVPMD-----------------NMYRH
UniRef90_G8IQ64_80_315    IFPHQFINLRTNNTATIVVPYINSVPID-----------------SMTRH
UniRef90_I0IY47_80_323    VYPHQIINLRTNNCATLVLPYVNSTSTD-----------------SMVKH
UniRef90_G5DC75_80_317    IFPHQFINLRSNNSATIIVPYVNAVPMD-----------------SMLRH
UniRef90_Q05ED2_80_316    IYPHQWINLRTNNSATIVMPYINSVPMD-----------------NMYRH
UniRef90_Q5VAK8_80_316    IFPHQWINLRTNNSATIVMPYINSVPMD-----------------NMYRH
UniRef90_A7KC11_80_330    IFPHQYINLRSNNSATLILPYVNAVPMD-----------------SMLRH
UniRef90_B5BSU6_131_316   IYPHQWINLRTNNCATIVMPYINCVPMD-----------------NMNRH
UniRef90_Q05ED0_80_316    IFPHQWINLRTNNCATIVMPYINSVPMD-----------------NMFRH
UniRef90_Q5VAL5_159_315   IFPHQWINLRTNNSATIVIPYINSVPMD-----------------NMFRH
UniRef90_A4UHT9_129_315   IFPHQLINLRTNNSSTIVVPYINCTPMD-----------------SMLRH
UniRef90_H8Y6P9_127_322   AYPHQLINLRTNNSCTIVVPYINAVPMD-----------------NMLRH
UniRef90_P29813_80_316    IYPHQWINLRTNNCATIVMPYINNVPMD-----------------NMFRH
UniRef90_P32537_131_305   VYPHQWINLRTNNSATIVLPWMNSAPMD-----------------FALRH
UniRef90_G5D5E2_159_315   IFPHQFINLRTNNTATLVVPYINSVPID-----------------SMTRH
UniRef90_Q9YLG5_80_319    IFPHQWINLRTNNSATIVMPYINSVPMD-----------------SMIRH
UniRef90_B8XTQ3_70_330    LFPHQLINPKDTNVATVRVPYVNVAPTS-----------------DPRVH
UniRef90_A7KC13_88_321    IFPHQFINLRTNNTATIVTPYINSVPMD-----------------SMTRH
UniRef90_O41174_80_282    IYPHQWINLRTNNSATLVLPYANAIPMD-----------------SPIRH
UniRef90_A5GZF9_80_318    IFPHQFINLRSNNSATIIVPYVNAVPMD-----------------SMLRH
UniRef90_G8IJE5_80_295    IFPHQFINLRSNNSATIIVPYVNAVPMD-----------------SMLRH
UniRef90_Q9QCF0_72_316    IFPHQWINLRTNNSATIVMPYINSVPMD-----------------NMFRH
UniRef90_G5DC71_157_324   IFPHQLINLRTNNSSTIVVPYINCVPMD-----------------SMLRH
UniRef90_A8S330_157_325   IFPHQLINLRTNNSATIVVPYINCVPMD-----------------SMLRH
UniRef90_Q9WN78_80_316    IYPHQWINLRTNNSATIVMPYINSVPMD-----------------NMYRH
UniRef90_A0MHB7_86_316    IFPHQLINLRTNNSSSIVVPYINCTPMD-----------------SMLRH
UniRef90_O89280_159_323   IFPHQWINLRTNNSATIVMPYINSVPMD-----------------NMFRH
UniRef90_G8IJG2_80_318    VYPHQYINLRSNNSATLILPYVNAVPMD-----------------SMVRH
UniRef90_F6M9Y3_80_316    IYPHQFINLRSNNSATLILPYVNAVPMD-----------------SMLRH
UniRef90_A5GZE0_80_317    IFPHQFINLRSNNSATIIVPYVNAVPMD-----------------SMLRH
UniRef90_Q7T7N9_80_323    VFPHQIINLRTNNCATLVLPYVNSVVTD-----------------SMAKH
UniRef90_A7YF31_159_315   IFPHQWINLRTNNSATIVIPYINSVPMD-----------------NMFRH
UniRef90_H9X8D4_80_317    IFPHQFINLRSNNSATIIVPYVNAVPMD-----------------SMLRH
UniRef90_G9K146_80_316    IYPHQLINLRSNNSATIIVPYVNAVPMD-----------------SMLRH
UniRef90_G9K138_159_330   IFPHQFINLRSNNSATIIVPYVNAVPMD-----------------SMLRH
UniRef90_G0YPI0_80_292    IFPHQWINLRTNNCATIIMPYINAVPFD-----------------SAINH
UniRef90_O91734_159_316   IFPHQWINLRTNNSATIVMPYVNSVPMD-----------------NMYRH
UniRef90_B9V473_80_315    IFPHQFINLRSNNSATLILPYVNAVPMD-----------------SMIRH
UniRef90_Q9WHH4_131_316   IFPHQWINLRTNNSATIVMPYINCVPMD-----------------NMFRH
UniRef90_Q2L970_80_316    IFPHQWINLRTNNCATIVMPYINSVPMD-----------------NMFRH
UniRef90_Q80GI2_159_316   IFPHQWITSRTNNCATIVMPYINCVPMD-----------------TMYRH
UniRef90_Q05ED1_131_316   IYPHQWINLRTNNSATIVMPYINCVPMD-----------------NMFRH
UniRef90_B9V440_80_317    IFPHQFINIRSNNSATLILPYVNAVPMD-----------------SMLRH
UniRef90_G1CSA8_80_313    IAPHQWINLRTNNCATIVMPYINAVPYD-----------------SALNH
UniRef90_B1PZZ4_80_316    IFPHQWINLRTNNCATIVMPYINNVPMD-----------------NMFRH
UniRef90_B9V457_80_316    IYPHQFINLRSNNSATLILPYVNAVPMD-----------------SMLRH
UniRef90_D8VCT6_80_323    VFPHQIINLRTNNCATLVLPYVNSSATD-----------------SMAKH
UniRef90_G8IRN4_129_324   IFPHQLINLRTNNSSTIVVPYINCVPMD-----------------SMLRH
UniRef90_Q80GI1_159_316   VFPHQWINLRTNNCATIVMPYINSIPMD-----------------NMFRH
UniRef90_I1XV41_80_323    VFPHQIINLRTNNCATLVLPYVNSTSTD-----------------SMAKH
UniRef90_P21404_159_316   IYPHQWINLRTNNSATIVMPYINSVPMD-----------------NMFRH
UniRef90_P23008_135_293   IFPHQFINLRSNNSATLIVPYVNAVPMD-----------------SMLRH
UniRef90_A5GZE2_80_315    IFPHQFINLRTNNSATLIVPYVNAVPMD-----------------SMLRH
UniRef90_Q6W9F4_159_316   IYPHQWINLRTNNCATIVMPYINCVPMD-----------------NMFRH
UniRef90_B3SRP0_80_317    IYPHQWINLRTNNCATIVMPYINCLPFD-----------------SALNH
UniRef90_Q6JKS0_159_312   VFPHQWINLRTNNCATIIVPYINAVPFD-----------------SAINH
UniRef90_G8IJG6_157_325   IFPHQLINLRTNNSSTIVVPYINCTPMD-----------------NMLRH
UniRef90_D4AH62_131_316   IFPHQWINLRTNNSATIVMPYINSVPMD-----------------NMFRH
UniRef90_A5GZD9_129_321   IFPHQFINLRTNNTATIVVPYINSVPMD-----------------SMTRH
UniRef90_Q66331_80_318    IFPHQWINLRTNNSATIVMPYTNSVPMD-----------------NMFRH
UniRef90_Q6W9E8_159_323   IYPHQWINLRTNNSATIVMPYINSVPMD-----------------NMHRH
UniRef90_P04936_159_330   IFPHQFINLRSNNSATIIAPYVNAVPMD-----------------SMRSH
UniRef90_A7KC07_80_326    IFPHQFINLRTNNSATLILPYMNCVSMD-----------------SMLRH
```

```
UniRef90_Q66806_80_316    IYPHQWINLRTNNCATIVMPYINSVPMD-----------------NMFRH
UniRef90_A5GZF5_80_318    IFPHQFINLRSNNSATLILPYVNAVPMD-----------------SMLRH
UniRef90_Q05ED4_159_315   IFPHQWINLRTNNSATIVMPYINSVPMD-----------------NMFRH
UniRef90_G8IRN3_129_329   IFPHQLINLRTNNSSTIVVPYINCVPMD-----------------NMLKH
UniRef90_Q82122_80_315    IFPHQFINLRSNNSATLIVPYVNAVPMD-----------------SMVRH
UniRef90_Q8V601_80_317    IYPHQWINLRTNNCATIVMPYINCLPFD-----------------SALNH
UniRef90_B9V433_129_314   IFPHQFINLRTNNTATIVVPYINSVPMD-----------------SMTRH
UniRef90_G5EN04_80_318    VFPHQWINLRTNNCATIIMPYVNALPFD-----------------SALNH
UniRef90_Q8V613_159_316   IYPHQWINLRTNNSATIVMPYINCVPMD-----------------NMQRH
UniRef90_Q6W9F9_80_316    IYPHQWINLRTNNSATIVMPYINSVPMD-----------------NMYRH
UniRef90_H9C6N7_159_315   IFPHQFINLRSNNSATLILPYVNAVPMD-----------------SMVRH
UniRef90_B9V434_80_314    IFPHQFINLRTNNAATLILPYVNATPMD-----------------FMLRH
UniRef90_A5GZF6_80_318    VYPHQYINLRSNNSATLILPYVNAVPMD-----------------SMVRH
UniRef90_A5GZF2_80_318    IYPHQYINLRTNNSATLILPYINAVPMD-----------------SMLRH
UniRef90_C7DUC8_80_317    IFPHQFINLRSNNSATLIVPYVNAVPMD-----------------SMLRH
UniRef90_G5CZJ4_127_322   IFPHQMINLRTNNSATIVVPYINCLPMD-----------------NMLRH
UniRef90_B9V477_80_318    IFPHQFINLRSNNSATLILPYVNAVPMD-----------------SMLRH
UniRef90_G8IJH3_88_314    IFPHQFINLRTNNTATLVMPYINSVPMD-----------------SMTRH
UniRef90_Q8UY33_159_317   IYPHQWINLRTNNCATIVMPYINAVPYD-----------------SALNH
UniRef90_P07210_80_318    IYPHQYINLRTNNSATLILPYVNAVPMD-----------------SMLRH
UniRef90_Q66577_131_323   IFPHQWINLRRNNSATIVIPYINCVPMD-----------------NMFRH
UniRef90_G8IJF0_127_318   IFPHQIINLRTNNSSTIIVPYINCTPMD-----------------SMLKH
UniRef90_E7CRL8_159_323   VYPHQIINLRTNNSATIVLPYVNAMAID-----------------SMVKH
UniRef90_Q995Z1_80_314    IFPHQWINLRTNNSATIVMPYINAVPMD-----------------NMYRH
UniRef90_C6ZEZ9_129_315   LYPHQMINLRTNNSATIVIPYINCVPMD-----------------NMLRH
UniRef90_Q5G9Z5_159_312   IFPHQWINLRTNNCATIVMPYINAVPYD------------
```

```
2WZR2                     KAWTLVVMVLAPYTNDQTIGST--KAEVYVNIAPTNVYVAGEKPVKQ
UniRef90_F1AW55_285_503   KAWTLVVMVVAPYTNDQTIGST--KAEVYVNIAPTNVYVAGEKPVKQ
UniRef90_Q6PMU0_285_503   KAWTLVVMVVAPYTNDQTIGSN--KAEVYVNIAPTNVYVAGEKPAKQ
UniRef90_Q8V0G8_86_304    KPWTLVVMVVAPYTNDQTIGST--KAEVYVNIAPTNVYVAGEKPVKQ
UniRef90_Q6PMU2_285_503   KAWTLVVMVVAPYTNDQTIGSS--KAEVYVNIAPTNVYVAGERPAKE
UniRef90_Q6PMT8_285_503   QAWSLVVMVLTPLTTEAQMNSG--TVEVYANIAPTNVFVAGEKPAKQ
UniRef90_A2I8Z5_287_504   KPWTLAVMVVAPLTV-NNEGAP--QIKVYANIAPTNVHVAGELPSKE
UniRef90_Q1L754_86_304    QAWSLVVMVLTPLTTEAQMNSG--TVEVYANIAPTNVYVAGEMPAKQ
UniRef90_B8Y0J7_285_503   QAWSLVVMVLTPLTTEAQMNSG--TVEVYANIAPTNVVVAGELPGKQ
UniRef90_Q1L757_86_304    QAWSLVVMVLTPLTTEAQMNSS--TVEVYASIAPTNVFVAGEKPSKQ
UniRef90_P49303_287_504   KPWTLVVMVVSPLTT-NTVSAG--QIKVYANIAPTHVHVAGELPSKE
UniRef90_A6XP89_287_504   KPWTLVVMVVAPLTV-KTGGSE--QIKVYMNAAPTYVHVAGELPSKE
UniRef90_Q6PMT3_285_502   KAWTLVVAVMAPLTT-SNMGQD--NVEVYANIAPTNVYVAGERPSKQ
UniRef90_Q1L752_86_304    QAWSLVVMVLTPLTTEAQMNSG--TVEVYANIAPTNVYVAGELPGKQ
UniRef90_Q1L748_86_303    KAWTLVVAVMAPLTT-GTMGSD--NVEVYANIAPTNVYVSGEKPAKQ
UniRef90_P15072_287_504   RPWTLVVMVVAPLTT-NTAGAQ--QIKVYANIAPTNVHVAGELPSKE
UniRef90_Q8JVD0_86_303    KAWTLVVAVLAPLTT-SNMGQD--NVRCTQTSPPTNVYVAGEKPTKQ
UniRef90_H9AY35_273_496   NAYTLVLVVISPLTT---GGHAKTVVKVKVTVAPVDVKVVGETPERQ
UniRef90_B0Z3W9_300_527   SVWSLVVGVVVPMTI-SNGGSDLTSLDVRASFTPLNVKVAGPMPNKQ
UniRef90_Q91LX8_289_537   CPWTLLIMVISPLRY-GAGSAP--DVQLTMTITPTDFVANG------
UniRef90_Q66776_291_513   CPWTLVIVVVSP-----------------------------------
UniRef90_Q100Y4_72_320    CPWTLMIMVVTPLRY-SSGAAT--DVQITATITPTDFVANG------
UniRef90_Q2XP17_290_516   NPWTIVILILSELTGPGQ------TVPVTMSVAPIDAMVNGPLP---
UniRef90_P12296_138_384   ASWTLVIAVVAPLTY-STGAST--SLDITASIQPVRPVFNG------
UniRef90_Q02472_148_405   ANWALVVAVLTPLQY-STGAAT--DVAITVSLQPVNPVFNG------
UniRef90_A4ZKN2_144_403   ANWTIVVAVLSPLQY-AAGSAT--EVDITLSIQPVKPVFNG------
UniRef90_C3U5A3_174_435   ANWTLVVAVLTPLQC-PTGSAP--DVEITASIQPVKPVFNG------
UniRef90_P08544_148_405   ANWTLVVAVLSPLQY-ATGSSP--DVQITASLQPVNPVFNG------
UniRef90_E3TMG9_144_405   ANWTLVIAVLTPLQY-SQGSAT--TIEITASIQPVKPVFNG------
UniRef90_G9FTJ7_144_402   ANWVLVVAVLSPLQY-SSGSSP--NVDITVSVQPIKPVFNG------
UniRef90_B3VTU7_149_406   ANWTLVVAVLTPLQY-AAGSAT--DVQITASIQPVKPVFNG------
UniRef90_Q155Z9_151_421   NSWTLLVMVLVPLDY-KEGATT--DPEITFSVRPTSPYFNG------
UniRef90_G1C9J1_14_204    NYWTLAIL---------------------------------------
UniRef90_I1YLT5_69_326    NVWSVIVMVISPLQYSNGAAPN---VAMTLTITPIDTVFNG------
UniRef90_G0Y2E2_141_365   NNWTVYVTYIVELSYMNGSS-T--EVPITVSVAPMSCSFSG------
UniRef90_G0Y2E0_150_361   NNWTIYITPVVPLDYVTGSS-T--NVPVTISIAP-------------
UniRef90_Q8B552_195_378   NFVTLVIRIVVPLSYSTGAS-T--QIPITVSVAP-------------
UniRef90_B5A1N0_195_378   NFVTLIVRVVVPLSYNTGAS-T--QVPITVSVAP-------------
UniRef90_Q2LKZ0_129_301   NNWTLSVIPIVPLE-YAAGATT--YVPITVTIAP-------------
UniRef90_Q65481_129_301   KNWTLAVIPLVDLA-YADGSTT--FVPITVTIAP-------------
UniRef90_G1DFA4_229_430   CPWTLVFMVLTP-----------------------------------
UniRef90_H9LG14_129_301   NNWTLAVIPVVDLA-YATGSTT--YVPITITIAP-------------
UniRef90_H6VLP2_125_335   NFWTVFITPVVPLSYNTGAT-T--SIPVTVSIAP-------------
```

```
UniRef90_B9VIN1_70_329    NIWTVVVMVLSPLQIGSGASPN---VQMTLTLTPTGSVFNG------
UniRef90_G1JYY8_171_367   NFFSLVIVPIA------------------------------------
UniRef90_Q8QUZ6_80_281    SNWSLMVMPVA------------------------------------
UniRef90_I1YLT7_70_335    TVWTAVVMVIAPLNYSNGASPT---VGMVMTITPVNSVFNGLRHTAQ
UniRef90_F5B9S5_80_301    NYWSLLVIPVVPLE-HATGTTP--FVAVTVTIAP-------------
UniRef90_G0Y2E4_163_340   NFVSLYIVPIVPLKFEKGAS-T--TIPVTVSISP-------------
UniRef90_B8XTQ1_73_321    TVWTAVVMVLVPLNFSEGASPT---VSITMTITP-------------
UniRef90_H9C6N6_131_322   NNVSLLVIPVVPLTNPTGMS-T--TIPITVTIAPMNTEFSG------
UniRef90_I1YLT4_70_334    TVWTAVVMVIAPLNYVTGASPT---VGMVMTITPVNSVFNGLRHTAQ
UniRef90_B8XTQ0_73_332    TVWTAVVMVIVPLNFSAGASPT---VSMTMSIAPLNSVFNG------
UniRef90_B9V442_80_329    NNWSLVIIPICPLESDGQTP-----VPITISISPMCAEFSGARAKSQ
UniRef90_D2IW00_80_316    NNWSIVIIPVSPLQ-TIEGT-P--YVPITLSISP-------------
UniRef90_Q2LL04_129_301   SNWTLAIIPVAPLK-YAADTTP--LVPITVTIAP-------------
UniRef90_I1YLT6_69_335    TIWTAVVMVICPLNFSNGASPI---VNMTLTITPVNSVFNGLRQYAQ
UniRef90_G5DC73_80_330    NNWSLVIIPICPLETMSTIT----TVPITISISPMCAEFSGARARSQ
UniRef90_A5GZE4_80_318    NNWSLVIIPICPLEVSSPGS-N--SIPITVSISP-------------
UniRef90_D9D8F3_70_328    TVWTAVVMVIVPLYYSAGASPT---VNMTMTITPVNSVFNG------
UniRef90_G0ZNT5_127_322   NNLTLLIIPLVPLRTGNTGG-T--TLPITVTIAPDKSEFSG------
UniRef90_B9V459_80_317    NNWSLLIIPVSPLKSD-QAS-V--RVPITISISP-------------
UniRef90_A8S322_129_323   NNLSLVIIPIVPLRAANGV--T--KVPITISIAPDKSEFSGAR----
UniRef90_C7DUC7_127_322   NNVTLGIIPLVPLRSGNTGR-T--TLHITVTIAPDKSELSG------
UniRef90_G8IJE2_80_317    NNWSLVIIPICPLETMSTIT----TVPITISISP-------------
UniRef90_Q2LKZ8_129_301   SNWTLAVIPVAPLK-YAAETTP--LVPITVTIAP-------------
UniRef90_B8XTP8_70_327    TVWTAVVMVLVPLNFSSGASPT---VSLTLTITPINSVFNG------
UniRef90_H2E685_80_301    SNWSLMIIPIVPLK-SATGTTP--FVGITVTIAP-------------
UniRef90_Q5YJ95_199_371   NFVTLVIRVLVPLRYNDGAS-T--FVPITVSVAPMCSMFAG------
UniRef90_Q9IM09_80_305    NVWSLVVIPVVPLD-YTTGATT--QVPITITMAP-------------
UniRef90_A5GZE9_80_317    NNWSLLIIPVSPLKSDASAT-S--KVPITISISP-------------
UniRef90_Q66790_80_316    YNFTLMVIPFVPLT-SMGGS-T--YVPITVTIAP-------------
UniRef90_A5GZD8_131_315   NNVSLLVIPVVPLVNPVGMS-T--IIPVTVTIAP-------------
UniRef90_P12915_129_301   SNWTLAIIPVAPLK-YAAETTP--LVPITVTIAP-------------
UniRef90_Q5XLP5_80_317    NNWSLLIIPVSPLEADTSAT-A--IVPITVSISP-------------
UniRef90_G0Z2U8_195_360   NFVTLVIRVLVPLKYNDGAS-P--FVPITVSVAP-------------
UniRef90_A1E4A3_131_303   NQWTLAIIPVVPL--GTRTMSS--MVPITVSIAP-------------
UniRef90_G0Y2E3_141_369   NIWTVYLVPVVPLEYAAGAS-T--SLPITVSVAPMSCSFSG------
UniRef90_B9V482_80_298    NNISLMVIPIVPLS---------------------------------
UniRef90_B8XTP9_157_328   NIWTAVVMVVSPLDFATGASPQ---VGMVLTITPVNSVFNG------
UniRef90_F8V2V4_80_316    NNFTLMIIPFAPLDYVTGAS-S--YIPITVTVAP-------------
UniRef90_A1E4A2_131_303   NQWTLAIIPVVPL--NTSGGTT--MVPITVSIAP-------------
UniRef90_Q8V5Z9_80_307    NNWTLAVIPISPLN-YDAGATP--LVPITITIAP-------------
UniRef90_G8IJE1_80_316    NNWSLVIIPICPLETNGGDT----TVPITISISP-------------
UniRef90_P12916_80_317    NNWSLVIIPISPLRSETTSS-N--IRPITVSISP-------------
UniRef90_P03303_80_315    NNVSLMVIPIAPLTVPTGAT-P--SLPITVTIAP-------------
UniRef90_B8XTQ4_69_329    NIWTALVVVIAPLQSNASASPT---VAMSMTVTPVGAVFNG------
UniRef90_Q9YLJ1_78_316    YNFTLMVIPFAPLA-SQGGS-T--YVPITITIAP-------------
UniRef90_B9V466_80_318    NNWSLVIIPVSKLQADNTGS-V--VVPITISISP-------------
UniRef90_Q05ED6_131_323   QNFTLMIIPFAPLEVSGGIT-P--SVPITVTIAPMDAEYNG------
UniRef90_B5BSU7_131_316   QNFTLMIIPFVPLTVNAAAS-N--QVPITVTVAP-------------
UniRef90_A5GZF1_80_316    NNWSLVIIPICPLESLPSST-----VPITISISP-------------
UniRef90_B9V453_80_313    NNWSLLIVPVSELR---GDT----SIPITVSISP-------------
UniRef90_Q05EE3_80_323    QNFTLMIIPFAPLEVQAGTA-T--AVPITVTVAPMQAEYNG------
UniRef90_A5GZE7_80_317    NNWSLVIIPISPLKSDPSAS-A--LVPITISISP-------------
UniRef90_B9V485_80_315    NNVSLLIIPISPLTAPTNSP-N--TLPVTVTIAP-------------
UniRef90_G8IJH5_80_318    NNWSLVIIPICPLEVSTTGT-N--GIPITVSISP-------------
UniRef90_B9V435_80_318    NNWSLVIIPICPLQSSGTES-S--TIPITISISP-------------
UniRef90_Q8V398_80_292    NNWTLCVVPVVPLD---------------------------------
UniRef90_Q82081_159_315   NNVSLMVVPIAPLNAPTGSS-P--TLPVTVTIAP-------------
UniRef90_Q6W9F6_159_316   HNFTLMIIPFAPLDFATGAS-S--YVPITVTIAP-------------
UniRef90_Q03053_159_316   NNFTLMIIPFAPLSYSTGAT-T--YVPITVTVAP-------------
UniRef90_A5GZD6_131_315   NNVSLMVIPIAPLAAPTGSA-T--TLPITVTIAP-------------
UniRef90_Q2YEN8_80_316    YNFTLMVIPFAPLDYTAQAS-E--YVPVTVTIAP-------------
UniRef90_G8HH17_117_311   SYWT-------------------------------------------
UniRef90_A7KC08_80_331    NNWSLVIIPICPLENSTEVP-N--AVPITISISPMCAEFSGARARSQ
UniRef90_Q6W9G1_131_314   YNFTLMVIPFAKLSVIGSNL-N--SVPITVTVAP-------------
UniRef90_G8IQ64_80_315    NNVSLLVIPIAPLVAPTGSL-Q--TLPITVTIAP-------------
UniRef90_I0IY47_80_323    NNWGIVVMPFVPLD-YDEGATT--KVPITLTVAP-------------
UniRef90_G5DC75_80_317    NNWSLVIIPISPLKAENATS-V--IVPITISISP-------------
UniRef90_Q05ED2_80_316    YNFTLMVIPFAPLNYS-NTT-T--FVPITITVAP-------------
UniRef90_Q5VAK8_80_316    HNFTLMVIPFVPLDYSTGAS-T--YVPVTVTVAP-------------
UniRef90_A7KC11_80_330    NNWSLVIIPICPLETQSQDT----TVPITISISPMYAEFSGARARSQ
UniRef90_B5BSU6_131_316   YNFTLMVIPFVPLD-SNGGT-T--YVPITVTVAP-------------
UniRef90_Q05ED0_80_316    YNFTLMVIPFVPLE-TMGGT-P--YVPITVTIAP-------------
UniRef90_Q5VAL5_159_315   YNFTLMIIPFAPLAYSTGAT-T--YVPITVTIAP-------------
```

```
UniRef90_A4UHT9_129_315   NNVSLVIIPICPLRAPATAP-S--TLPITISIAP-------------
UniRef90_H8Y6P9_127_322   NNLTLMIVPIVPLKPGLSGS-P--SLPITITIAPNKSEFSG------
UniRef90_P29813_80_316    HNFTLMIIPFVPLDYSSDSS-T--YVPITVTVAP-------------
UniRef90_P32537_131_305   NNWTLAVIPVCPLAGGTGNTNT--YVPITISIAP-------------
UniRef90_G5D5E2_159_315   NNVSLMIIPIAPLIVPTNAS-P--TLPITVTIAP-------------
UniRef90_Q9YLG5_80_319    NNFTLMIIPFVPLDYVNGSS-P--YIPITVTVAP-------------
UniRef90_B8XTQ3_70_330    NIWTAVIMVVTPLKYADGASPT---VQMTLTITPIDTVFNG------
UniRef90_A7KC13_88_321    NNLSLLVIPVVPITANTGSP-P--SIPVTVTIAPMNLELSG------
UniRef90_O41174_80_282    SSWSLLVIPVVP-----------------------------------
UniRef90_A5GZF9_80_318    NNWSLVIIPICELQVDTGVP-A--TVPITISISP-------------
UniRef90_G8IJE5_80_295    NNWSLVII---------------------------------------
UniRef90_Q9QCF0_72_316    YNFTLMIIPFVPLDFTAEAS-T--YVPITVTVAP-------------
UniRef90_G5DC71_157_324   NNVSLVVIPICPLRKSGNVS-Q--TLPITISIAPDKSEFSGAR----
UniRef90_A8S330_157_325   NNISLVIIPLVPLRSGSTQTIN--RLPITISIAPDKSEFSGAR----
UniRef90_Q9WN78_80_316    YNFTLMVIPFAKLEHSPQAS-T--YVPITVTVAP-------------
UniRef90_A0MHB7_86_316    NNVSLVIIPICPLKTPSGAP-R--TLPITVSIAPDR-----------
UniRef90_O89280_159_323   NNFTLMIIPFAKLAYSDGAS-T--FVPITVTIAPMNAEYNG------
UniRef90_G8IJG2_80_318    NNWSIVIIPISKLE-TEGGT-T--IVPITLSISP-------------
UniRef90_F6M9Y3_80_316    NNWSIVIIPVSPLD-S-QAT-G--NVPITISISP-------------
UniRef90_A5GZE0_80_317    NNWSLVIIPICELKCDTTAA----TVPITISISP-------------
UniRef90_Q7T7N9_80_323    NNWGIVVIPFVKLA-YQNGATT--KVPITVTIAP-------------
UniRef90_A7YF31_159_315   YNFTLMIIPFAPLKYDTGAT-P--YVPITVTVAP-------------
UniRef90_H9X8D4_80_317    NNWSLVIIPICSLRSETSST-N--IVPITVSISP-------------
UniRef90_G9K146_80_316    NNWSLVIIPICPLESMPAST-----VPITISISP-------------
UniRef90_G9K138_159_330   NNWSLVIIPICPLEANGVQP----VVPITISISPMCAEFSGARAKSQ
UniRef90_G0YPI0_80_292    NNCGLIVVPVAP-----------------------------------
UniRef90_O91734_159_316   HNFTLMIIPFVPLDFSAGAS-T--YVPITVTVAP-------------
UniRef90_B9V473_80_315    NNWSIVIIPISPLE-A-NVT-D--PVPITISISP-------------
UniRef90_Q9WHH4_131_316   QNFTLMLIPFVPLDYSDGAS-S--YVPITVTVAP-------------
UniRef90_Q2L970_80_316    YNFTLMIIPFAKLDYAAGSS-T--YIPITVTVAP-------------
UniRef90_Q80GI2_159_316   YNFTLMVIPFVKLR-SDGGT-T--YVPITVTIAP-------------
UniRef90_Q05ED1_131_316   YNFTLMLIPFAPMK-SVGGT-S--YVPITITIAP-------------
UniRef90_B9V440_80_317    NNWSLVIIPICPLESQGTEG----VVPITVSISP-------------
UniRef90_G1CSA8_80_313    CNFGLFVIPVAPLN-YSDGATT--AIPITVTIAP-------------
UniRef90_B1PZZ4_80_316    HNFTLMIIPFVPLDYSSDSS-T--YVPVTVTVAP-------------
UniRef90_B9V457_80_316    NNWSLVIIPVSPLA-S-EST-D--KVPITISISP-------------
UniRef90_D8VCT6_80_323    NNWGIVILPFVPLQ-YEDGAST--AIPITVTVAP-------------
UniRef90_G8IRN4_129_324   NNVSLVIIPVCPLRTSGNAP-R--TLPVTISIAPDKSEFSGAR----
UniRef90_Q80GI1_159_316   YNFTLMVIPFVPLD-VSGGT-P--YVPITVTIAP-------------
UniRef90_I1XV41_80_323    NNWGIVVLPFVPLD-YDSGATP--NVPITVTIAP-------------
UniRef90_P21404_159_316   YNFTLMVIPFVKLDYADTAS-T--YVPITVTVAP-------------
UniRef90_P23008_135_293   NNWCLVIIPISPLRSETTSS-N--IVPITVSISP-------------
UniRef90_A5GZE2_80_315    NNWSLVIIPICQLEVVGGVS-N--TVPITIS---------------
UniRef90_Q6W9F4_159_316   YNFTLMIIPFVPLA-EMGGT-T--YVPITVTVAP-------------
UniRef90_B3SRP0_80_317    CNFGLVVIPVAPLA-YSAGATT--AIPITLSIAPLNSEFAG------
UniRef90_Q6JKS0_159_312   NNFTLVVIPISPLK-YCTGATT--AIPITVTIAPLN-----------
UniRef90_G8IJG6_157_325   NNVSLLIIPICPLRTSNGAP-T--TLPITISIAPDKSEFSGAK----
UniRef90_D4AH62_131_316   YNFTLMIIPFAKLEYVSGAS-S--VVPITVTIAP-------------
UniRef90_A5GZD9_129_321   NNLSLMVIPIVNITATSGAA-P--SISITVTIAPMYLELSG------
UniRef90_Q66331_80_318    NNVTLMVIPFVPLDYCPGST-T--YVPITVTIAP-------------
UniRef90_Q6W9E8_159_323   YNFTLMIIPFVPLDYSDGAS-T--YVPITITIAPMDAEYNG------
UniRef90_P04936_159_330   NNWSLVIIPICPLETSSAIN----TIPITISISPMCAEFSGARAKRQ
UniRef90_A7KC07_80_326    NNWSLLIIPVCPLQVDAQAT-R--FVPITISISPMSAEFSGAR----
UniRef90_Q66806_80_316    YNFTLMVIPFAQLDYAPSAS-T--HVPITVTVAP-------------
UniRef90_A5GZF5_80_318    NNWSLVIIPVCPLEVAQGSE-Q--YVPITISISP-------------
UniRef90_Q05ED4_159_315   YNFTLMIIPFAKLDYTATA--S--YVPITVTVAP-------------
UniRef90_G8IRN3_129_329   NNLTLVIVPLVPLRFGNSGA-N--TLPITISIAPDKSEFSGARQKSQ
UniRef90_Q82122_80_315    NNWSLVIIPVCQLQSN-NIS-N--IVPITVSISP-------------
UniRef90_Q8V601_80_317    CNFGLIVIPVAPLA-YSSGATS--AIPITLSIAPLNSEFAG------
UniRef90_B9V433_129_314   NNLSLMVIPIVDITATSGTT-P--SIPVTITIAP-------------
UniRef90_G5EN04_80_318    SNFGLVVIPISPLK-YCNGATT--EVPITLTIAPLNSEFSG------
UniRef90_Q8V613_159_316   NNLTLMIIPFVELDYSAGAT-T--FVPITVTVAP-------------
UniRef90_Q6W9F9_80_316    YNFTLMIIPFVALDFN-NTA-S--NVPITITVAP-------------
UniRef90_H9C6N7_159_315   NNWSIVIIPISPLQ-A-DIA-D--PVPITISISP-------------
UniRef90_B9V434_80_314    NNWSLLIVPISQLR---GDT-N--SIPITISISP-------------
UniRef90_A5GZF6_80_318    NNWSIVIIPISKLV-SVGGT-A--YVPITISISP-------------
UniRef90_A5GZF2_80_318    NNWSLVIIPICPLQVESSGI-P--TVPITVSISP-------------
UniRef90_C7DUC8_80_317    NNWSLVIIPICELLVDANAS-A--VVPITISISP-------------
UniRef90_G5CZJ4_127_322   NNLTLLIIPIVKLRHGTTGT-T--TLPITVTIAPDKSEFSG------
UniRef90_B9V477_80_318    NNWSLVIIPVCPLEVAVGSE-Q--YVPITISISP-------------
UniRef90_G8IJH3_88_314    NNLSLLVIPVIPITATSGAA-H--SVPITVTIAP-------------
UniRef90_Q8UY33_159_317   CNFGLAIIPVAPLD-YNAGATT--AIPITVTIAPMNSEFAG------
UniRef90_P07210_80_318    NNWSLVIIPICPLQVQPGGT-Q--SIPITVSISP-------------
```

```
UniRef90_Q66577_131_323   N N F T L M V I L F A P L K - S S G G T - N - - Y V S I T I T V A P M D A E Y N G - - - - - -
UniRef90_G8IJF0_127_318   N N V S L V I I P I V P L R V N G T G P - T - - T I P I T V S V A P - - - - - - - - - - - - -
UniRef90_E7CRL8_159_323   N N W G I A I L P L S P L D - F A Q D S S V - - E I P I T V T I A P - - - - - - - - - - - - -
UniRef90_Q995Z1_80_314    Y N F T L M I I P F A K L E Y A S G S S - T - - V V P I T V T V A P - - - - - - - - - - - - -
UniRef90_C6ZEZ9_129_315   N N F S L V I V P I V P L R P G N S G A - P - - I L P I T V T I A P - - - - - - - - - - - - -
UniRef90_Q5G9Z5_159_312
```

1	2	3	4	5	6	7	8	9

Variable Average Conserved

x - Insufficient data - the calculation for this site was performed on less than 10% of the sequences.

Figure 3S

```
2WZR3                       -----GILPVAVSDGYGGFQNTDPKTSDPVYGHVYNPARTLYPGRFTNLL
UniRef90_F1AW55_504_724     -----GILPVAVSDGYGGFQNTDPKTSDPIYGHVYNPARTGYPGRYTNLL
UniRef90_Q6PMU0_504_724     -----GILPVAVSHGYGGFQNTDPKTSDPVYGHVYNPARTGLPGRFSNLL
UniRef90_Q6PMU2_504_724     -----GIVPVAVADGYGGFQNTDPKTSDPIYGHVYNAPRTGYPGRYTNLL
UniRef90_Q8V0G8_305_525     -----GIFPVAAAAGYGGFQNTDPKTSDPIYGHVYNPARTGYPGRFTNLL
UniRef90_Q6PMT3_503_723     -----GIIPVACNDGYGGFQNTDPKTADPIYGLVSNAPRTAFPGRFTNLL
UniRef90_Q1L748_304_524     -----GIIPVACNDGYGGFQNTDPKTADPIYGLVVNPPRNAFPGRFTNLM
UniRef90_Q6PMT8_504_725     -----GIIPVACSDGYGGFQNTDPKTADPIYGYVYNPSRNDCHGRYSNLL
UniRef90_B8Y0J7_504_725     -----GIVPVAAADGYGGFQNTDPKTADPIYGYVYNPSRNDCHGRFSNLM
UniRef90_Q1L757_305_524     -----GIVPVACADGYGGFQNTDPKTADPIYGHVYNPPRNDCCGRFTNFM
UniRef90_Q1L752_305_526     -----GIVPVACSDGYGGFQNTDPKTADPIYGHVYTPSRQDCHGRFSNLL
UniRef90_Q1L754_305_526     -----GILPVACADGYGGFQNTDPKTADPIYGHVYNPSRDGCHGRFSNLM
UniRef90_P49303_505_725     -----GIVPVACSDGYGGLVTTDPKTADPVYGMVYNPPRTNYPGRFTNLL
UniRef90_Q6PMY9_505_723     -----GIVPVACVDGYGNMVTTDPKTADPVYGKVFNPPRTNLPGRFTNFL
UniRef90_A2I8Z5_505_724     -----GIFPVACSDGYGGLVTTDPKTADPVYGKVFNPPRNLLPGRFTNLL
UniRef90_P15072_505_723     -----GIFPVACSDGYGNMVTTDPKTADPAYGKVYNPPRTALPGRFTNYL
UniRef90_Q8JVD0_304_524     -----GIVPVACHDGYGGFQNTDPKTADPIYGLVSNPPRTAFPGRFTNLL
UniRef90_H9AY35_497_713     -----GLVPLANHIGYGGFKTTAPLTADPVCGNVYNPPRQDMPGRFTNFM
UniRef90_B0Z3W9_528_746     -----GIVPVAAKAGYSGFCTTSPITADPVYGKVVNPPRRHIPGRFTNFL
UniRef90_Q2XP17_533_745     ------------------FMSSVPDNSTPLYPKVVVPPRQ-VPGRFTNFI
UniRef90_Q100Y4_324_550     --AVSEGIPGT-QPYDRQFLSAEPSAPPPVYTPSWLPDRSFIPGKFTDFL
UniRef90_Q91LX8_542_768     ---VSEGIPGT-QPYDRQFLSTEPSAPPPLYTPSWLPDRSFIPGKFTDFL
UniRef90_A4ZKN2_415_639     -------IPVTVREHAGTFYSTTPDNTVPVYGKTVATPSDYMCGEFTDLL
UniRef90_E3TMG9_417_639     -------FPVTVREHAGTFFSTTPDTTVPVYGNTISTPFDYMCGKFTDLL
UniRef90_P08544_417_642     -------IPVTVREHKGCFYSTNPDTTVPIYGKTISTPSDYMCGEFSDLL
UniRef90_B3VTU7_418_646     -------IPVTVREHQGTFYSTNPDTTVPIYGKTIATPSDYMCGEFSDLI
UniRef90_Q66776_543_769     --AVAQGIPGT-QPYDRQFVSSEPSAPPPVYTPSWLPERSFIPGKFTDFL
UniRef90_G9FTJ7_414_638     -------IPVTIREHAGTFYSTTPDTTVPIYGKTIATPSDYMCGEFDDLL
UniRef90_Q02472_417_641     -------VAVTVREHQGSFYSTNPDTTVPIYGKTIVTPSDYMCGEFTDLL
UniRef90_P12296_396_619     -------IPVTIREHAGTWYSTLPDSTVPIYGKTPVAPANYMVGEYKDFL
UniRef90_C3U5A3_447_672     -------FPGTIREHAGTFYSTTPDTTVPVYGKTISTPANYMCGEFTDLL
UniRef90_G1DFA4_460_684     PQTITQGFAGHIRENQAMYASTIPDETTPVYGLGFNPNTDFVPGEVHNFL
UniRef90_Q155Z9_448_665     ------------------FLSTLPDDTVPAYGNVRTPPVNYLPGEITDLL
UniRef90_B9VIN1_352_561     ------------------FSSTVPDVTEPCYGMTVVPPKDFMPAPITDLV
UniRef90_G8HZ08_715_947     -------VPTFQVPGSGQFVSTLRNAGYPLYAHFEPTHSFENPGRFVNLL
UniRef90_B8XTQ3_353_566     ------------------FSSTVPDVTEPCYGMTVNPPKDYMPEPVGDLV
UniRef90_D9D8F3_345_562     ---------------FDQFNTTVPLRTEPCYGMTVTPPVDYMPPPVEDLM
UniRef90_I1YLT4_348_565     ------------------FNTTVPLRTEPCYGMTLTPPLDYMPPPIEDLA
UniRef90_B8XTP9_335_563     -----GPIPTRPFHNFNQFNTTVPLRTEPCYGMTLTPPVDYMPKPIDDLV
UniRef90_I1YLT7_349_565     ------------------FNTTVPLITEPCYGMTLTPPTDYMPKPIEDLA
UniRef90_I1YLT6_336_558     -----GPIPVRTFHNSSQFATTVPLRAEPCYGMTVTPPTDYMPDPIDDLV
UniRef90_B8XTP8_334_562     -----GPIPVRPFHNFQQFSTTVPLRTEPCYGMTVTPPVDYMPLPITDLV
UniRef90_B8XTQ0_353_567     ------------------FSTTVPLRTEPCYGMTVTPPVDYMPLPITDLV
UniRef90_Q5YJ95_381_603     -------FPVRQVPGSQQFMTTQRDSGIPIYPEFEKTHGFKLPGRVTNLL
UniRef90_B8XTQ4_341_566     -------IPVRMTQNSGQFSTTLPARMEPCYGLTPNPTRDFLPPVVEDLL
UniRef90_I1YLT5_349_558     ------------------FSSTVPDVTEPAYGMTVAPPRDYMPPPVDDLA
UniRef90_F6IA11_421_636     -------VPTFDIPGSGQFCTTLANTGYPAFPIWTPSEGFPLPGRMTNLI
UniRef90_G0Z2U8_412_599     ------------------------------------------PGRVTNLL
UniRef90_Q5YJA7_377_599     -------FPIRQVPGSQQFMTTQRDSGIPIYPEFEKTHSFKLPGRVTNLL
UniRef90_G0Y2E4_357_574     -------VPTFQIPGSGQFVTTIKNDGFPALPHYQETPLHFIPGEVTNLL
UniRef90_Q8B552_395_615     -------IPVRQIPGSSQFLTVQRDAGVPLYPEFQKTARFTLPGRVTNYM
UniRef90_A7KC13_333_552     -------LPTTLTPGSEQFLTTDDRQSPSALPNYEPTPLIHIPGRVTNLL
UniRef90_A5GZD6_337_514     -------LPTTTLPGSGQFLTTDDRQSPSALPNYEPTPKIHIPGEVKNLL
UniRef90_G5CZJ8_367_551     ------------------------------------------PGRVVNLL
UniRef90_G8HH17_373_575     ------------------------NPGYPLYPEYQKTPPHHIPGRVTNLM
UniRef90_G0Y2E3_380_601     -------IPVFEVPGSGQFMTTLRNSGYPCLPDYEETPSHHIPGKVVNLL
UniRef90_B9V482_337_556     -------LPTTTLPGSGQFLTTDDRQSPSALPNYEPTPRIHIPGQVRNLL
UniRef90_A5GZF6_334_555     -------LPVMLTPGSEQFLTTDDAQSPSALPYFHPTKEIFIPGRVTNLS
UniRef90_A7KC10_335_556     -------LPVMLTPGSEQFLTTDDNQSPSALPWFHPSKEIFIPGRVTNLA
UniRef90_G8IJG2_334_555     -------LPVMLTPGSAQFLTTDDAQSPSALPYFHPTKEIFIPGRVNNLS
UniRef90_D2IW00_332_553     -------LPVMLTPGSGQFLTTDDAQSPSALPYFHPTKEIFIPGRVTNLS
UniRef90_A5GZD9_331_550     -------LPTVLTPGSEQFLTTDDRQSPSAMPNYEPTPLIHIPGEVRNLL
UniRef90_A7KC07_334_555     -------LPVMITPGSGQFLTTDDAQSPSALPYFHPTKEIFIPGQVKNLI
UniRef90_Q672R3_805_1000    -------------------------------------HSFNLPGRVRNLL
UniRef90_B5A1N0_416_617     ----------------------------PVYPEFEKTHGFRLPGRVTNFL
UniRef90_B9V433_331_550     -------LPAVLTPGSEQFLTTDDRQSPSAMPNYEPTPLIHIPGEVKNLL
UniRef90_Q82081_333_552     -------LPTTTLPGSGQFLTTDDRQSPSALPSYEPTPRIHIPGKVRNLL
UniRef90_Q5XLP5_336_557     -------LPVYMTPGSGQFLTTDDLQSPSALPWYHPTKEIFIPGQVRNLI
UniRef90_B9V457_333_554     -------LPVMLTPGSAQFLTTDDTQSPSALPYFHPTKEIFIPGKVSNLS
```

```
UniRef90_G1JYY8_404_619   -------IPVFQVPGSSQFLTTLRNDGYPIYPEFSTTHSYPLPGRVRNLL
UniRef90_F6N707_334_556   -------LPVMLTPGSAQFLTTDDTQSPSALPYFHPTKEIFIPGKVNNLS
UniRef90_G5D5D9_333_552   -------LPTTTLPGSGQFLTTDDRQSPSALPNYEPTPRIHIPGKVRNLL
UniRef90_Q82122_332_553   -------LPVYVTPGSGQFMTTDDMQSPCALPWYHPTKEIFIPGEVKNLI
UniRef90_G5D5E2_333_552   -------LPTTTLPGSGQFLTTDDRQSPSALPNYEPTPRIHIPGQVKNLL
UniRef90_A5GZF5_335_556   -------LPVFITPGSAQFLTTDDTQSPSALPYFHPTKEIFIPGEVKNLS
UniRef90_G0Y2E2_378_600   -------VPVFEVPGSGQFVTTIRNSGFPILPDYEETPNHGIPGRVRNLM
UniRef90_P03303_333_552   -------LPTTTLPGSGQFLTTDDRQSPSALPNYEPTPRIHIPGKVHNLL
UniRef90_P07210_338_559   -------LPVMLTPGSGQFLTTDDTQSPSAFPYFHPTKEIFIPGQVRNLI
UniRef90_F6M9Y3_333_554   -------LPVMLTPGSAQFLTTDDTQSPSALPYFHSTKEIFIPGKVSNLS
UniRef90_B9V459_336_557   -------LPVYVTPGSGQFMTTDDLQSPCALPWYHPTKEIFIPGEVRNLI
UniRef90_B9V477_333_554   -------LPVFLTPGSAQFLTTDDTQSPSALPYFHPTKEIFIPGEVKNLS
UniRef90_H9C6N6_333_552   -------LPTLTTPGSGQFLTTDDRQSPSAMPNYEPTPPIHIPGEVHNLL
UniRef90_A5GZE7_336_557   -------LPVYVTPGSGQFMTTDDLQSPSALPWYHPTKEILIPGEVKNLI
UniRef90_B9V485_333_552   -------LPTTTLPGSGQFLTTDDRQSPSALPCYEPTPKIHIPGEVKNLL
UniRef90_G0Y2E0_419_588   ------------------------------------------PGRVTNLL
UniRef90_G9K138_332_516   -------LPVMVTPGSGQFMTTDDYQSPSALPWFHPTKEISIPGQVRNLI
UniRef90_A5GZE9_336_557   -------LPVYITPGSGQFMTTDDLQSPSALPWYHPTKEIFIPGEVKNLI
UniRef90_B9V434_332_553   -------LPVMLTPGSGQFLTTDDMQSPSVLPYFHPTQEIFIPGEVKNLV
UniRef90_P04936_332_552   -------LPVFITPGSGQFLTTDDFQSPCALPWYHPTKEISIPGEVKNLV
UniRef90_C7DUC8_336_557   -------LPIYMTPGSGQFMTTDDFQSPSALPWYHPTKEILIPGEVKNLI
UniRef90_G8IQ74_332_548   -------LPVRMPSGSNQFMTTEDEQSPNILPSFHSTKQIHIPGQIKTVM
UniRef90_I2CMI9_334_554   -------LPVRMPSGSQQFMTTEDEQSPNMLPGYHSSKKIHIPGMITNVL
UniRef90_B9V466_338_522   -------LPTYITPGSGQFMTTDDFQSPSALPWYHPTKEILIPGEVKNLI
UniRef90_A5GZF9_333_554   -------IPTMLTPGSGQFMTTDDFQSPSALPWYHPTKEISIPGQVRNLI
UniRef90_G8IJE2_332_552   -------LPVLVTPGSGQFLTTDDYQSPCALPWYHPTKEIFIPGEVKNLV
UniRef90_H8Y6Q0_168_390   -------LPVRLASGTQQFMTTEDEQSANLLPGFHPTPVMHIPGEITNLM
UniRef90_G0YPI0_327_543   -------IPVEMKPGTNQFLTTDDGVSAPILPGFDPTPVIHIPGEVSSIL
UniRef90_B9V473_333_554   -------LPVMLTPGSAQFLTTDDAQSPSALPYFHPTKEIFIPGEVKNLS
UniRef90_B5BSU6_368_556   ------------------------------------------PGEVHNLM
UniRef90_A8S322_365_548   ------------------------------------------PGRITNIL
UniRef90_G8IQ62_334_555   -------IPTMLTPGSGQFMTTDDFQSPSALPWYHPTKEISIPGQVRNLI
UniRef90_B9V453_331_552   -------LPVMLTPGSGQFLTTDDMQSPSVLPYFHSTQEIFIPGEVKNLI
UniRef90_G9K146_333_520   -------LPVFITPGSGQFMTTDDFQSPSALPWFHPTREISIPGQVKNLV
UniRef90_F6M9Z0_330_551   -------LPVMLTPGSGQFMTTDDFQSPCALPWYHPTKEISIPGQVRNLI
UniRef90_P12916_334_555   -------LPVYITPGSGQFMTTDDMQSPCALPWYHPTKEISIPGEVKNLI
UniRef90_G5EN04_327_500   -------FPTELKPGTNQFLTTDDGTSPPILPGFEPTPLIHIPGEFTSLL
UniRef90_C7DUC6_357_551   ---------------------------------FHPSKKIHIPGMITNIL
UniRef90_Q9WN78_367_554   ------------------------------------------PGQVRNLM
UniRef90_G0YPI1_326_499   -------LPVEIKPGSYQFLTTDDEVSAPILPGFQPTPEIHIPGEVRNLL
UniRef90_G0YPH9_326_499   -------VPVEMKPGTNQFLTTDDGTSAPILPGFHPTPTIHIPGEVHSLL
UniRef90_Q8UY33_326_552   -------IPVELKPGSNQFLTTDDEVSAPILPGFHPTPEIHIPGEVKNLL
UniRef90_A5GZE0_334_555   -------LPTLLTPGSGQFMTTDDYQSPSALPWYHPTKEISIPGQVQNLI
UniRef90_G0ZNT7_334_555   -------LPVRMPSGAQQFMTTEDEQSPNILPGYHSSKKIHIPGKITSVL
UniRef90_A7KC11_332_515   -------LPVLMTPGSGQFMTTDDYQSPSALPWFHPTKEIFIPGQVQNLS
UniRef90_G1JYY7_238_455   -------VPTFNVPGSGMFMTTLRNAGFPIYPEFSKTHSFPLPGRVRNLL
UniRef90_H6UKU5_326_499   -------VPMELKPGSNQFLTTDDEVSAPILPGFNATPNIYIPGEVTNLL
UniRef90_Q03053_367_554   ------------------------------------------PGQVNNLM
UniRef90_Q995Z1_365_551   ------------------------------------------PGEVKNLM
UniRef90_B9V442_331_555   -------LPVMLTPGSGQFMTTDDFQSPSALPWFHPTKEISIPGQVTNLI
UniRef90_G1CSA8_329_506   -------LPAELKPGTNQFLTTDNGVSAPILPGFHPTPAIHIPGEVHNLL
UniRef90_A5GZE4_333_554   -------LPTMMTPGSGQFMTTDDFQSPCALPWFHPTKEISIPGEVKNLI
UniRef90_G8IJF4_357_552   ---------------------------------FHPSKKIQIPGKITSVL
UniRef90_A5GZF2_336_558   -------LPVMLTPGSGQFLTTDDTQSPSAFPYFHPTKEIFIPGQVRNLI
UniRef90_A5GZF1_333_554   -------LPVFITPGSGQFMTTDDFQSPSALPWFHPTREISIPGQVKNFV
UniRef90_Q5U904_326_555   -------LPAELKPGTNQFLTTDDGVSAPILPGFHPTPEMHIPGEVKNLL
UniRef90_Q6JKR7_325_551   -------IPAELXPGTNQFLTTEDGTSAPILPGFHPTQVIHIPGEVRNLL
UniRef90_G8IJF0_360_553   ---------------------------------FHPSKKIHIPGMITNVM
UniRef90_A1E4A3_319_512   -------VPTYLLPGSGQFLTTDDHSSAPVLPCFNPTPEMHIPGQVRNML
UniRef90_Q8QUZ6_317_541   -------LPVTNTPGSYQFMTTDEDSSACVLPDFNPTQAIHIPGEVTNLQ
UniRef90_Q2LL04_324_545   ------------GPGSYQFMTSDEDSTPCILPDFQPTPEIFIPGEVTNLL
UniRef90_F6M9Y7_364_585   -------LPAMLTPGSGQFMTTDDFQSPCALPWFHPTKEISIPGQVRNLI
UniRef90_Q8V601_326_552   -------LPTELKPGTNQFLTTDDGVAAPILPGFHPTPEMFIPGEVKNLL
UniRef90_Q6JKS0_326_499   -------IPVELKPGTNQFLTTDNEVSVPILPGFQPTPTIHIPGEVNNLL
UniRef90_B3SRP1_326_550   -------LPVELKPGSNQFLTTDDGVSAPILPGFHPTPLIHIPGEVNNLL
UniRef90_G5CZJ4_357_550   ---------------------------------YSPTKEIHIPGKITNML
UniRef90_B5BSU7_367_555   ------------------------------------------PGEVKNLM
UniRef90_G8IRN3_333_554   -------LPVRLASGTQQFMTTEDEQSANLLPGFHPTPTMHIPGEITNIM
UniRef90_A5GZE2_333_554   -------LPVMMTPGSGQFMTTDDYQSPSALPWFHPTKEISIPGEVKNLV
UniRef90_A7KC08_333_554   -------LPVMITPGSGQFLTTDDFQSPSALPWFHPTKEISIPGQVRNLV
UniRef90_H9BP13_325_500   -------LPMELKPGTNQFLTTDDGVSAPILPGFHPTPVIHIPGEVRNLL
UniRef90_Q80GI2_332_555   -------LPVLNTPGSNQFLTTDDYQSPCAMPEYDVTPPLHIPGEVSNLM
UniRef90_B9V435_333_554   -------LPVLTTPGSGQFMTTDDFQSPSALPWFHPTKEISIPGQVRNLI
```

```
UniRef90_Q5G9Z5_326_554   -------LPAELKPGTNQFLTTDDGVSAPILPGFHPTPEIHIPGEVTNLL
UniRef90_Q9YLG5_370_557   ------------------------------------------PGRVHNLM
UniRef90_G8IJE5_332_553   -------LPVLLTPGSGQFMTTDDFQSPSALPWYHPTKEISIPGQVRNLV
UniRef90_Q8V614_326_499   -------LPVELKPGTNQFLTTDDDDAPPVLPGFQATPEMHIPGEVTNLL
UniRef90_Q5GA06_355_555   ---------------------------------YHPTQEIHIPGEVKNLL
UniRef90_G8IJG6_332_548   -------IPMRLPSGSNQFMTTEDEQSPNILPNFHPTKKIHIPGEIKTIM
UniRef90_Q05ED6_367_555   ------------------------------------------PGEVRNLM
UniRef90_P23008_310_531   -------LPVYITPGSGQFMTTDDMQSPCALPWYHPTKEISIPGEVKNLI
UniRef90_Q8JRP9_365_551   ------------------------------------------PGEVHNLM
UniRef90_Q05EE3_367_555   ------------------------------------------PGEVKNLM
UniRef90_Q8V604_326_552   -------LPVVLTPGSTQFLTTNDGVSAPILPGFHPTPAIHIPGEVKNIL
UniRef90_C7DUC7_331_549   -------IPTRMPSGSQQFMTTEDEQSPNILPEYSPTKLMHIPGEITNVL
UniRef90_P12915_324_545   ------------GPGSYQFMTTDEDCSPCILPDFQPTLEIFIPGKVNNLL
UniRef90_G8IJE1_331_509   -------LPVLMTPGSGQFMTTDDYQSPSALPWFHSTKEIFIPGQVHNLS
UniRef90_A4UHT9_332_548   -------LPVRMPSGANQFMTTEDEQSPNILPSFHSTKQIHIPGEIKTIM
UniRef90_Q9QCF0_367_554   ------------------------------------------PGEVKNLM
UniRef90_Q9QF31_325_500   -------IPTELKPGTNQFLTTDDGVSAPILPGFHPTPPIHIPGEVHNLL
UniRef90_Q65900_325_551   -------IPTELKPGTNQFLTTDDGVSAPILPGFHPTPAIHIPGEVRNLL
UniRef90_E5D8F2_334_553   -------LPTRLPSGSQQFMTTEDEQSPNILPGFHPSKKIHIPGMITNVM
UniRef90_A1E4A2_319_512   -------VPTYLLPGSSQFMTTDDHSSAPAFPDFSPTPEIHIPGRVHNML
UniRef90_B9V440_332_553   -------LPVMITPGSGQFMTTDDYQSPSALPWFHSTKEISIPGQVRNLA
UniRef90_Q6W9F4_332_507   -------LPVLATPGSDQFLTSDDYQSPCAMPEYDVTPPMRIPGEVRNLM
UniRef90_G8IJH5_359_554   ---------------------------------FHPTKEISIPGEVKNLI
UniRef90_B1PZZ4_333_555   -------LPVMITPGSNQFLTSDDFQSPTAMPQFDVTPELNIPGEVQNLM
UniRef90_O89280_367_554   ------------------------------------------PGQVNNLM
UniRef90_P32537_321_545   -------VPTCLLPGSNQFLTTDDHSSAPAFPDFSPTPEMHIPGQVHSML
UniRef90_G0YPI2_326_499   -------IPAELRPGTNQFLTTDDDTAAPILPGFTPTPTIHIPGEVHSLL
UniRef90_Q9YLJ1_368_556   ------------------------------------------PGEVRNIM
UniRef90_Q2YEN8_367_554   ------------------------------------------PGEVHNLM
UniRef90_A7YF31_331_554   -------LPALLTPGSNQFLTSDDFQSPSAMPQFDPTPLMHIPGEVHNLM
UniRef90_Q65481_317_544   -------LPTMYTPGSGQFLTTDDFQSPSILPKFKATPEIEIPGRVLNIL
UniRef90_Q2LKZ0_317_544   -------IPTLYTPGSGQFLTTDDFQTPCMLPKFQPTPVIDIPGEVKNFL
UniRef90_Q05ED0_367_555   ------------------------------------------PGEVRNLM
UniRef90_Q8V5Z9_323_547   -------LPAQLTPGSTQFVTTDNDSSQPLIPNFDETTPIHIPGEVRNML
UniRef90_Q6W9F9_367_555   ------------------------------------------PGEVRNLM
UniRef90_H8Y6P9_356_549   ---------------------------------FSPTKQIHIPGKIDSIL
UniRef90_G5DC71_332_548   -------IPTRMPSGSNQFMTTEDEQSPNILPSFHSTKQMHIPGQIKTIM
UniRef90_C6ZEZ9_341_560   -------IPTRIPAGSQQFMTTEDEQSPNILPEYSPTKEIHIPGKIDNML
UniRef90_Q05ED4_367_554   ------------------------------------------PGEVKNLM
UniRef90_H6VBU4_358_522   --------------------------------------------------
UniRef90_Q8V398_325_495   -------LPVKATPGSYQFLTTSDDCSEGLLPNFHSTPEIFIPGQVNNLL
UniRef90_D8VCT6_342_524   -------LPVLNTPGSNQFLTTDNFQSPCIVPNYDVTAPIHIPGEVRNMM
UniRef90_D9YV24_514_704   -----------------------------------NPPTTYVPGRTTNYM
UniRef90_Q66577_367_555   ------------------------------------------PGEVHNLM
UniRef90_A0MHB7_357_547   ---------------------------------FSPTKQIHIPGQIKTIM
UniRef90_E9LS20_360_544   ---------------------------------FHPTKKIHIPGLVTNIM
UniRef90_Q9WHH4_367_554   ------------------------------------------PGEVKNLM
UniRef90_G0ZNT5_357_550   ---------------------------------YTPTKQIFIPGEITNML
UniRef90_Q5YJA3_454_673   ------------------FSSTQPYTAAPVYGKIPASNPSYMPGEFLDYV
UniRef90_H9U0H0_456_678   ------------------FSSTQPYQGPPVYGKISASDPSYMPGEFNDYV
UniRef90_Q6W9E8_367_554   ------------------------------------------PGQVKNLM
UniRef90_Q80GI1_367_556   ------------------------------------------PGEVRNIM
UniRef90_C7B298_377_565   ------------------------------------------PGEVKNMM
```

```
2WZR3                     DVAEACPTLLD----------------F-NG-VPYVQTQS-------NSG
UniRef90_F1AW55_504_724   DVAEACPTFLD----------------F-NG-VPYVQTQN-------NSG
UniRef90_Q6PMU0_504_724   DVAEACPTLLD----------------F-SG-RPYVITQA-------NSG
UniRef90_Q6PMU2_504_724   DVAEACPTFLD----------------F-NG-VPYVTTQN-------NSG
UniRef90_Q8V0G8_305_525   DVAEACPTLLD----------------F-NG-VPYVTTQN-------NSG
UniRef90_Q6PMT3_503_723   DVAEACPTFLD----------------F-DG-TPYVKTRH-------NSG
UniRef90_Q1L748_304_524   DVAEACPTFLD----------------F-DG-TPYVKTKH-------NSG
UniRef90_Q6PMT8_504_725   DVAEACPTFLN----------------F-DG-KPYVVTK--------NNG
UniRef90_B8Y0J7_504_725   DVAEACPTLLN----------------F-DG-KPYVVTR--------NSG
UniRef90_Q1L757_305_524   DAAEACPTLLN----------------F-DG-KPYVVTK--------NNG
UniRef90_Q1L752_305_526   DVAEACPTLLN----------------F-DG-KPYVVTK--------SSG
UniRef90_Q1L754_305_526   DVAEACPTLLN----------------F-DG-KPYVVTG--------NSG
UniRef90_P49303_505_725   DVAEACPTFLC----------------FDDG-KPYVVTR--------TDE
UniRef90_Q6PMY9_505_723   DVAEACPTFLR----------------F-GE-VPFVKTV--------NSG
UniRef90_A2I8Z5_505_724   DVAEACPTFLH----------------FDGD-VPYVTTK--------TDS
UniRef90_P15072_505_723   DVAEACPTFLM----------------F-EN-VPYVSTR--------TDG
```

```
UniRef90_Q8JVD0_304_524    GRRCSVPNFLW----------------TSTG-HRTLRPST-------TVA
UniRef90_H9AY35_497_713    HVAQVCPTFGR----------------IGSGSVPYFLTT--------QDS
UniRef90_B0Z3W9_528_746    DVADACPTMAR----------------F-TS-KPSITTVS-------GAS
UniRef90_Q2XP17_533_745    DVAKQTYSFCS----------------ISGK-PYFEVTNT-------SGD
UniRef90_Q100Y4_324_550    QVAV-IPTLAE-------VSV-AS---YKPV-PSFSVSNV-------MQE
UniRef90_Q91LX8_542_768    QVAV-IPTMAE-------VSVGTN---HKPI-PSFSVGNT-------LED
UniRef90_A4ZKN2_415_639    SLCK-IPTFLG-------NVSSTQ-----RL-PYFSATNT-------VPN
UniRef90_E3TMG9_417_639    SLCK-IPTFLG-------NLDSNK----KRI-PYFSATNS-------TPA
UniRef90_P08544_417_642    ELCK-LPTFLG-------NP-NTN---NKRY-PYFSATNS-------VPA
UniRef90_B3VTU7_418_646    ELCK-LPTFLG-------NPANTSPP-GGRY-PYFSATNS-------VPI
UniRef90_Q66776_543_769    QVAV-IPTLAE-------VSV-QN---YKPV-PSFPVSNV-------LQT
UniRef90_G9FTJ7_414_638    SLCK-IPTFLG-------NPATDQ-----RL-PYISASNT-------VGD
UniRef90_Q02472_417_641    ELCK-LPTFLG-------NLSNDT-----RV-PFFTATNS-------VPT
UniRef90_P12296_396_619    EIAQ-IPTFIG-------NKVPNA------V-PYIEASNT-------AVK
UniRef90_C3U5A3_447_672    SLCK-IPTFVG-------NNLDSK-----WN-PYFSSTNS-------VNE
UniRef90_G1DFA4_460_684    EWTR-TPCLMS-------NVVDDTIR------GYFTASNT-------RSD
UniRef90_Q155Z9_448_665    QLAR-IPTLMAFERVPEPVPASDT-----YV-PYVAVPTQ-------FDD
UniRef90_B9VIN1_352_561    SLAK-VPTFCT-------V--------TENK-PYFEVNNN-------KSD
UniRef90_G8HZ08_715_947    EVAM-IGTFAD------VGQTQA----ASEE-IPDGTAITTKMNAVVGNL
UniRef90_B8XTQ3_353_566    SLAK-VGSLVT-------VSYPS----NSDQ-PYFEISNT-------KKD
UniRef90_D9D8F3_345_562    SLAQ-VPCFLS-------VAFSDNSI--KGF-PYFAVSNT-------EQG
UniRef90_I1YLT4_348_565    DLVK-IPSFCS-------VSFTESSSDEKGF-PYFSVSNG-------TQG
UniRef90_B8XTP9_335_563    SLVK-VPSFIT-------NSGSDTPT-GRSF-PYFSVSSS-------TQG
UniRef90_I1YLT7_349_565    DLVR-VPSFCT-------VPF-RNTFRHKSF-PYFAVSNG-------DPG
UniRef90_I1YLT6_336_558    SVAK-VPSFVC-------I--------TDSI-PYFAVSNA-------TQG
UniRef90_B8XTP8_334_562    ELAK-VPSFVT-------VANSDTTS-ERSF-PYFSVSNT-------EQG
UniRef90_B8XTQ0_353_567    SLSK-VPSFLT-------VAQGDTTK-QRN--PYFAVSNG-------TQG
UniRef90_Q5YJ95_381_603    QVAQ-VGTFLK------FRNNVN----DASG-VYLNLDVT--------QG
UniRef90_B8XTQ4_341_566    SIAK-VPCFLL-------ADEDT----TKQK-PYFLISNA-------SST
UniRef90_I1YLT5_349_558    SLAK-VPSFVT-------V--------TVDK-PYFEVTNA-------KST
UniRef90_F6IA11_421_636    SVAR-IPTFLQ------GTGGNT----YGFS-VQNAIPAD----------
UniRef90_G0Z2U8_412_599    QVAQ-VGTFLK------FSNNTN----NESG-VYLNLDIT--------QG
UniRef90_Q5YJA7_377_599    QVAQ-VGTFLK------FSNNVN----DASR-VYLNLDIT--------QG
UniRef90_G0Y2E4_357_574    EVCQ-IDTFAN------LGTS---------E-LSFTIDVS-------NQT
UniRef90_Q8B552_395_615    QVAR-VPTLMK------MPNTMS----DGT---YLAVDVT--------QN
UniRef90_A7KC13_333_552    ELVQ-VDTMIP------INNVTDL----DTI-EKYRIPLM-------QNQ
UniRef90_A5GZD6_337_514    EIIQ-VDTLIP------MNNTHTK----DEV-KSYLIPLV-------ANQ
UniRef90_G5CZJ8_367_551    EIAQ-VDSLIP------INNIANN----DNL-ERYRIPLT-------PNV
UniRef90_G8HH17_373_575    EVAE-VDTLCN------LSDT---------E-VLY-INAT-------GSA
UniRef90_G0Y2E3_380_601    EVMS-VDTFCN------CSDS-D----TQTK-MYFTLNV---------NI
UniRef90_B9V482_337_556    EIIQ-VDTMIP------MNKTKVN----DEV-GNYLIPLI-------QGQ
UniRef90_A5GZF6_334_555    EICQ-IDSMIP------INNTHGV---KNTV-NMYTVQVQ-------EQT
UniRef90_A7KC10_335_556    EICQ-VDTLIP------VNNVQAS---KNNV-SMYTVMLS-------TQT
UniRef90_G8IJG2_334_555    EICQ-IDSMIP------VNNTHAV---KNSV-NMYTVEVR-------SQA
UniRef90_D2IW00_332_553    EICQ-VDSMIP------INNTVAS---HKRV-SMYAVRVG-------VQT
UniRef90_A5GZD9_331_550    EIAQ-VDTLIP------INNAEGI----TGL-ETYRIPLY-------QNR
UniRef90_A7KC07_334_555    EMCQ-VDTLIP------VNNVQAS---KNNI-SMYTVQLT-------TQT
UniRef90_Q672R3_805_1000   EVAQ-IPTLVT------GLKEGT----EGWH-RPWLVNIE-------QSM
UniRef90_B5A1N0_416_617    EVAR-VGTIVA------MQSTSN----QSSQ-LYYTLDLT--------QN
UniRef90_B9V433_331_550    EIAQ-VDTLIP------LNNTTNT----TGL-GMYRIPLV-------QNM
UniRef90_Q82081_333_552    EIIQ-VGTLIP------MNNTGTN----DNV-TNYLIPLH-------ADR
UniRef90_Q5XLP5_336_557    EMCQ-VDTMIP------INNTNER---IGNV-NMYTVSLT-------SQT
UniRef90_B9V457_333_554    EICQ-VDSMIP------VNNTTPV---NNSI-GMYTVRVE-------VQT
UniRef90_G1JYY8_404_619    EVAR-VGTFCD------FKGK-----------SSITIDVG-------K--
UniRef90_F6N707_334_556    EICQ-VDSMIP------INNIIAN---KNSI-NLYTVRVE-------VQT
UniRef90_G5D5D9_333_552    EIIQ-VDTLIP------MNNVHTS----DEV-NSYLIPLT-------ANR
UniRef90_Q82122_332_553    EMCQ-VDTLIP------INSTQSN---IGNV-SMYTVTLS-------PQT
UniRef90_G5D5E2_333_552    EIIQ-VDTLIP------MNNTHNR----DEV-KSYLIPLQ-------PNR
UniRef90_A5GZF5_335_556    EICQ-VDSMIP------INNVEQH---KNSV-DMYSVVLT-------KQT
UniRef90_G0Y2E2_378_600    EVCG-VDTFCN------CATSTT----TDEQ-LSFSVSI---------ND
UniRef90_P03303_333_552    EIIQ-VDTLIP------MNNTHTK----DEV-NSYLIPLN-------ANR
UniRef90_P07210_338_559    EMCQ-VDTLIP------VNNTQEN---VRSV-NMYTVDLR-------TQV
UniRef90_F6M9Y3_333_554    EICQ-VDSMIP------INNTIAM---NNKI-GMYTVEVG-------VQT
UniRef90_B9V459_336_557    EMCQ-VDTLIP------INNTFAN---LRNV-NKYAVSLF-------SQT
UniRef90_B9V477_333_554    EICQ-VDSMIP------VNNVQGN---KNSV-NMYSIILD-------KQT
UniRef90_H9C6N6_333_552    EIIQ-VDTLIP------MNNTGAR----DTM-DEYRIPLE-------VNK
UniRef90_A5GZE7_336_557    EMCQ-VDSLIP------INNTTAN---LRKV-NMYTIQLA-------NQT
UniRef90_B9V485_333_552    EMIQ-VDTLIP------MNNTTAT----DEV-GNYLIPLN-------VNK
UniRef90_G0Y2E0_419_588    EVMQ-VDTFCN------PVNS------TNAA-VSFTLDVS-------NNS
UniRef90_G9K138_332_516    EMCQ-VDTLIP------INNLEQN---IRNV-NMYSVVLN-------QET
UniRef90_A5GZE9_336_557    EMCQ-VDTLIP------INNTASN---IRNV-SMYTVQLT-------SQV
UniRef90_B9V434_332_553    ELCQ-VDTMVP------LNNINAS---RNRI-DMYALPLT-------RQP
UniRef90_P04936_332_552    EICQ-VDSLVP------INNTDTY---INSE-NMYSVVLQ-------SSI
```

```
UniRef90_C7DUC8_336_557   EMCQ-VDTSIP------INNTTQN---LRKI-NMYTIQLE-------NQT
UniRef90_G8IQ74_332_548   DMAR-VDSFMP------INNIPSQ---IQKV-GIYNIQVS-------AAS
UniRef90_I2CMI9_334_554   HMAR-VDSFMP------INNIRTE---IGKV-SIYNIPIL-------RKT
UniRef90_B9V466_338_522   EMCQ-VDTLIP------INNTDNN---LRKV-NLYTVELT-------NQP
UniRef90_A5GZF9_333_554   ELCE-VDTLIP------INNTSEN---LKTT-NMYTVYLE-------RRS
UniRef90_G8IJE2_332_552   GICQ-VDSLIP------LVXXXXX---XTSE-AMYSIKLQ-------SSV
UniRef90_H8Y6Q0_168_390   HMAR-VDSFMP------INNISNH---INKV-PIYNVPIL-------KKS
UniRef90_G0YPI0_327_543   ELCQ-VETILE------VNNVTD----VQGV-SRLLIPVK-------AQE
UniRef90_B9V473_333_554   EICQ-VDSIIP------INNVEAR---KNSI-SMYTVEVG-------VKT
UniRef90_B5BSU6_368_556   EIAE-VDSVVP------VNNL-AGT--INTI-DAYGIPVS-------ASN
UniRef90_A8S322_365_548   HMAM-VDSFMP------LNNIETN---VGQI-SIYNVQVT-------KKT
UniRef90_G8IQ62_334_555   EMCE-VDTLIP------INNVGTN---VKNV-NMYTVLLE-------RQN
UniRef90_B9V453_331_552   ELCQ-VDTMVP------LNNLHIN---KNKI-GMYALPLT-------RQN
UniRef90_G9K146_333_520   ELCQ-VDTLIP------INSTTAN---NDNT-SIYTVTLE-------KQT
UniRef90_F6M9Z0_330_551   ELCQ-VDTLIP------INNLEQN---VNNT-DMYSVVLN-------NET
UniRef90_P12916_334_555   EMCQ-VDTLIP------VNNVGTN---VGNI-SMYTVQLG-------NQM
UniRef90_G5EN04_327_500   DLCQ-IETILE------VNNTTS----TTGV-SRLLIPVR-------AQN
UniRef90_C7DUC6_357_551   HMAR-VDSFMP------INNIRNH---EGKI-SIYNITVV-------RKT
UniRef90_Q9WN78_367_554   EIAE-VDSVVP------VDNT-EEH--VNSI-EAYRIPVR-------PQT
UniRef90_G0YPI1_326_499   ELCQ-VETILE------VNNTTD----THGM-SRLLIPVS-------AQT
UniRef90_G0YPH9_326_499   ELCQ-IETMLE------VNNTTR----AVEL-DRLRIPVS-------AQS
UniRef90_Q8UY33_326_552   EIAQ-VESILE------VNNLKTNE--NDPM-RRLLIPVS-------VQS
UniRef90_A5GZE0_334_555   ELCQ-VATMIP------INNTNTN---IRDK-SMYTIRLG-------GET
UniRef90_G0ZNT7_334_555   HMAR-VDSFMP------INNVESE---IGKV-SIYNVTVT-------KKA
UniRef90_A7KC11_332_515   ELCQ-VDTIIP------INNISSN---TTSV-NMYSLVLT-------KER
UniRef90_G1JYY7_238_455   EVAQ-VGTFAS------FGKS-----------HKLVINVG-------KRT
UniRef90_H6UKU5_326_499   ELCQ-IETMLE------VNNVTD----VDGM-QRLLIPVS-------AQT
UniRef90_Q03053_367_554   EIAE-VDSVVP------VNNT-EGK--VSSI-EAYQIPVQ-------SNS
UniRef90_Q995Z1_365_551   EIAE-VDSLVP------VNNT-SRL--TRSI-EAYGIPVR-------TGL
UniRef90_B9V442_331_555   ELCQ-VDTLIP------VNNTETNG--VTNI-NMYTVTIN-------REV
UniRef90_G1CSA8_329_506   EIAQ-VESILE------VNNLTSLETPSTLM-QRLLIPVE-------AKS
UniRef90_A5GZE4_333_554   ELCQ-VDTMIP------INNVGNN---TKSV-DMYTVVLQ-------RQD
UniRef90_G8IJF4_357_552   HMAR-VDSFMP------INNIKAQ---EGSV-NMYHITVT-------RKG
UniRef90_A5GZF2_336_558   EMCQ-VDTLIP------INNTGNN---VRSA-NMYTVDLT-------TQ-
UniRef90_A5GZF1_333_554   ELCQ-IDTLIP------INSVTGN---TNNT-SIYTVTLT-------RET
UniRef90_Q5U904_326_555   EICQ-VESILE------VNNLTTNKAASQLM-TRLLIPVE-------AQT
UniRef90_Q6JKR7_325_551   ELCQ-VETMLE------VNNLESNE--NDPM-QRLLIPVQ-------SQS
UniRef90_G8IJF0_360_553   HMAR-VDSFIP------LNNIEGE---TTKP-SVYCVTVP-------KKT
UniRef90_A1E4A3_319_512   EVVQ-VESMME------INNTEN----AVGM-ERLKVDIS-------ALT
UniRef90_Q8QUZ6_317_541   ALCQ-VESMLE------INNTPD----QQRN-GRLWLTVG-------AQS
UniRef90_Q2LL04_324_545   QIAQ-VESIVE------ANNVAD----ATGV-ERYVIPVS-------VQD
UniRef90_F6M9Y7_364_585   ELCQ-VDTLIP------INNLQAN---VTNL-DMYSVVLN-------REI
UniRef90_Q8V601_326_552   EIAQ-VESILE------VNNLTTNE--TNPM-ERLLIPVS-------VQS
UniRef90_Q6JKS0_326_499   ELCQ-VESILE------VNNVTG----QEGI-NRLLIPVS-------TQS
UniRef90_B3SRP1_326_550   QLCQ-IESILE------VNNVPS----ASQM-QRLLIPVS-------VQS
UniRef90_G5CZJ4_357_550   HIAQ-VESFLP------LNNIDRH---VGSI-DIYGVTIT-------GTS
UniRef90_B5BSU7_367_555   EIAE-VDSLVP------VNNV-PEK--VNSL-EAFQIPVS-------TGS
UniRef90_G8IRN3_333_554   HMAR-VDSFMP------INNTSER---VD-V-PIYNITVT-------RKS
UniRef90_A5GZE2_333_554   EMCQ-VDTLIP------INNTPGN---VNTI-NMYTVLLE-------RKD
UniRef90_A7KC08_333_554   ELCQ-VDTLIP------IDSVEAN---IKST-NMYTVTLT-------RET
UniRef90_H9BP13_325_500   ELCQ-IDTIME------VNNLTTNE--ATPM-ERLRIPVQ-------VQT
UniRef90_Q80GI2_332_555   EIAE-VDSVVP------VNNL-NDK--VNTI-ETFGIPVS-------ASD
UniRef90_B9V435_333_554   EMCQ-VDTIIP------INNTGTN---TTSI-NMYTVQLE-------RTA
UniRef90_Q5G9Z5_326_554   EICQ-VESIIE------VNNLSTLETSSTAM-QRLSFEVS-------VQS
UniRef90_Q9YLG5_370_557   EIAE-VDSVVP------LNNI-QDN--LRKM-DIYRVQVS-------SQT
UniRef90_G8IJE5_332_553   ELCQ-VDTLIP------INNTEAN---IRNK-NMYTVQLG-------GEP
UniRef90_Q8V614_326_499   ELCQ-VESILE------VNNTTT----ATGV-ERLLIPVS-------VQS
UniRef90_Q5GA06_355_555   EICR-VESILE------VNNLTTNE--ANPM-ERLLIPVS-------VQS
UniRef90_G8IJG6_332_548   DMAR-VDSFMP------INNIQSQ---LQDV-SVYNVQVT-------PQA
UniRef90_Q05ED6_367_555   EIAE-VDSVVP------VNNL-SKN--VNTM-EAYQIPVN-------TGN
UniRef90_P23008_310_531   EMCQ-VDTLIP------VNNVGNN---VGNV-SMYTVQLG-------NQT
UniRef90_Q8JRP9_365_551   EIAE-VDSLVP------VNNT-ARN--TRSI-EAYGIPVR-------AGV
UniRef90_Q05EE3_367_555   EVAE-VDSVVP------VNNN-PRD--VNSI-RAYQIPVS-------TGT
UniRef90_Q8V604_326_552   EIMQ-VESILE------VNNLNSNE--STPM-NRLVIPVS-------VQS
UniRef90_C7DUC7_331_549   HMAQ-VEPFIP------INNIQAQ---IGRI-SMYTIPVT-------RKA
UniRef90_P12915_324_545   EIAQ-VESILE------ANNREG----VEGV-ERYVIPVS-------VQD
UniRef90_G8IJE1_331_509   ELCQ-VDTIIP------INYTSAN---TTSV-NMYTVVLD-------KGE
UniRef90_A4UHT9_332_548   DMAR-VDSFMP------INNV-NQ---SHTL-GVYYITIS-------NVS
UniRef90_Q9QCF0_367_554   EIAE-VDSVVP------VNNT-QDS--VYNM-DVYKIPVS-------GGN
UniRef90_Q9QF31_325_500   EICR-VETILE------VNNLKTNE--TTPM-QRLCFPVS-------VQS
UniRef90_Q65900_325_551   EICR-VETILE------VNNLQSNE--TTPM-QRLCFPVS-------VQS
UniRef90_E5D8F2_334_553   HMAR-VDSFIP------INNIQGE---VGKV-SMYYITVT-------KKT
UniRef90_A1E4A2_319_512   ELVQ-VESMME------INNVAD----ASGM-ERLRVEIS-------VQT
```

```
UniRef90_B9V440_332_553   ELCQ-VDTIIP------INHTTAT---TSSV-NMYTVVLD-------KKT
UniRef90_Q6W9F4_332_507   EIAE-VDSVIP------INNV-SDN--LQST-RVYQIPVS-------AAN
UniRef90_G8IJH5_359_554   ELCQ-VDSMIP------INNVTDK---IRSV-DMYTVVLQ-------RQD
UniRef90_B1PZZ4_333_555   EIAE-VDSVVP------VNNV-EGK--LDTM-DIYRIPVQ-------SGN
UniRef90_O89280_367_554   EIAE-VDSVVP------VNNT-ETN--VNGM-DAYRIPVQ-------SNM
UniRef90_P32537_321_545   EIVQ-IESMME------INNVND----ASGV-ERLRVQIS-------AQS
UniRef90_G0YPI2_326_499   ELCR-VETILE------VNNTTE----ATGL-TRLLIPVS-------SQN
UniRef90_Q9YLJ1_368_556   EIAE-VDSVVP------VNNM-SSK--VKTI-EAYQIPVS-------VGT
UniRef90_Q2YEN8_367_554   EIAE-VDSVVP------VNNT-QNN--LQSM-EAYHIEVN-------TGN
UniRef90_A7YF31_331_554   EIAE-VDSVIP------INNT-TGK--CKTM-EAYQVAVR-------ANN
UniRef90_Q65481_317_544   EIVQ-VESLVE------INNVDQ----VNGV-ARYRIPLS-------VQD
UniRef90_Q2LKZ0_317_544   EVVQ-VESLVE------INNVES----AEGV-ARYRIPLN-------VQD
UniRef90_Q05ED0_367_555   EIAE-VDSVVP------MNNI-PAN--EKKM-ETYQIWVE-------ARD
UniRef90_Q8V5Z9_323_547   EICQ-VESILE------VNNVTG----VNGM-SRLLIPIS-------VQS
UniRef90_Q6W9F9_367_555   EIAE-VDSVMP------INNI-EGN--LRNM-KAYRVQVS-------TGN
UniRef90_H8Y6P9_356_549   HMAM-IESFIP------INNIPDH---IGKV-AIYSIQIT-------RKV
UniRef90_G5DC71_332_548   DMAR-VDSFMP------INNIPSQ---IQEV-GVYNITVS-------GTS
UniRef90_C6ZEZ9_341_560   HIAM-VDSLIP------INNQRSH---TGQV-AMYNVQVS-------KKS
UniRef90_Q05ED4_367_554   EIAE-VDSVVP------VNNT-ARN--IHSM-EAYRIPVQ-------CFN
UniRef90_H6VBU4_358_522   --------------------------------------------------
UniRef90_Q8V398_325_495   ELVQ-IETMMT------INNVAAQ---SAGV-ERISIPVS-------VQS
UniRef90_D8VCT6_342_524   ELAE-IDSVIP------MNAV-AGK--VNTM-EAYPIPLN-------QGT
UniRef90_D9YV24_514_704   GLAN-QPGLMA------------------------LVDIG-------MDS
UniRef90_Q66577_367_555   EIAE-VDSVVP------VNNT-STH--MEGT-DAFQIKVT-------AGN
UniRef90_A0MHB7_357_547   DMAR-VDSFMP------INNLQSQ---LQDV-GVYNITLT-------GTS
UniRef90_E9LS20_360_544   HIAR-VDSFIP------LNNIEGQ---VGRV-SMYYVPVT-------KKV
UniRef90_Q9WHH4_367_554   EIAE-VDSVVP------INNV-EAA--ISSM-EAYRVEVN-------TSK
UniRef90_G0ZNT5_357_550   HIAQ-VESFIP------LNNTEGH---LGKM-DIYRVTVT-------QKA
UniRef90_Q5YJA3_454_673   QLAQ-IPSLVK----------------NREI-RVSSVDKT----------
UniRef90_H9U0H0_456_678   QLAQ-IPSLVK----------------NREI-RVSSVEQT----------
UniRef90_Q6W9E8_367_554   EIAE-VDSVVP------LNNI-GSA--VNTL-EAYRVAVA-------SMA
UniRef90_Q80GI1_367_556   EIAE-IDSVVP------VNNVDEDK--VKSI-EAYRIPMN-------VVG
UniRef90_C7B298_377_565   EIAE-VDSLIP------MNAV-PGK--ANTM-DMYRIGLD-------SNA


2WZR3                     -----SKVLACFDLA-FG-HKNMKNTY--MSGLAQYFAQYSGTLNLHFMY
UniRef90_F1AW55_504_724   -----SKVLTCFDLA-FG-HKNFKNTF--MSGLAQYYTQYSGTLNLHFMY
UniRef90_Q6PMU0_504_724   -----SKVLTLFDLA-FG-HKNLKNTF--MSGLAQYYTQYSGTLNLHFMY
UniRef90_Q6PMU2_504_724   -----SKVMACFDLA-FG-HKNLKNTF--LSGLAQYYTQYSGTLNLHFMY
UniRef90_Q8V0G8_305_525   -----SKVLTCFDLA-FG-HKNMKNTY--LSGLAQYYAQYSGTLNLHFMY
UniRef90_Q6PMT3_503_723   -----SKILAHIDLA-FG-HKSFKNTY--LAGLAQYYAQYSGSINLHFMY
UniRef90_Q1L748_304_524   -----SKIMTHIDLA-FG-HKSFKNTY--LAGLAQYFAQYSGTLNLHFMY
UniRef90_Q6PMT8_504_725   -----DKVMTYFDVA-FT-HKVHKNTF--LAGLADYYTQYQGSLNYHFMY
UniRef90_B8Y0J7_504_725   -----DKVMTAFDVA-FT-HKVHKNTF--LAGLADYYTQYSGSLNYHFMY
UniRef90_Q1L757_305_524   -----DKVMTRFDVA-FT-HKVHKNTF--LAGLADYYTQYSGSLNYHFMY
UniRef90_Q1L752_305_526   -----DKVMAAFDVA-FT-HKVHKNTF--LAGLADYYTQYTGSLNYHFMY
UniRef90_Q1L754_305_526   -----NKVMTAFDVA-FT-HKVHRNTF--LAGLADYYTQYSGSLNYHFMY
UniRef90_P49303_505_725   -----QRLLAKFDLS-LA-AKHMSNTY--LSGIAQYYAQYSGTINLHFMF
UniRef90_Q6PMY9_505_723   -----DRLLAKFDMS-LA-AGHMSNTY--LAGLAQYYTQYSGTMNIHFMF
UniRef90_A2I8Z5_505_724   -----DRVLAQFDLS-LA-AKHMSNTF--LAGLAQYYAQYSGTINLHFMF
UniRef90_P15072_505_723   -----QRLLAKFDVS-LA-AKHMSNTY--LAGLAQYYTQYTGTINLHFMF
UniRef90_Q8JVD0_304_524   -----ARYSHTLTWH-LG-TRSFKNTY--VAGLAQYYAQYSGSLNLHFMY
UniRef90_H9AY35_497_713   -----DELLSTIDVS-FT-SFEMSSTF--LAGLAQFYAQYRGTINVHFTF
UniRef90_B0Z3W9_528_746   -----ERLLATIDVS-LV-SHEMSFTY--LAGLSSLYAQYRGSINMHCIY
UniRef90_Q2XP17_533_745   ------EPLFQMDVS-LS-AAELHGTY--VASLSSFFAQYRGSLNFNFIF
UniRef90_Q100Y4_324_550   ------KPLVNTDLT-FT-SMTFRNTY--LASLAQHFTQYRGSLCLDLLF
UniRef90_Q91LX8_542_768   ------RPLLNTDLT-FT-SMTFRNTY--LSALALNYTQYRGSICVDFLF
UniRef90_A4ZKN2_415_639   -----N-ALVTYQVS-LS-CSCMANSM--LASVARNFNQYRGSLNYLFVF
UniRef90_E3TMG9_417_639   -----T-PLVTYQVT-LS-CSCMANSM--LAAVARNFNQYRGSLNYLFVF
UniRef90_P08544_417_642   -----T-SMVDYQVA-LS-CSCMANSM--LAAVARNFNQYRGSLNFLFVF
UniRef90_B3VTU7_418_646   -----T-ALASYQVA-LS-CSCMSNSM--LAAVARNFNQYRGSLNFLFVF
UniRef90_Q66776_543_769   ------KPLVNTDLT-FT-SMTFRNTY--VSALSLQYTQYRGSICMDLLF
UniRef90_G9FTJ7_414_638   -----Q-PLATYQVT-LS-CSCMANSM--LAAVSRNFNQYRGSLNYLFVF
UniRef90_Q02472_417_641   -----E-SLVEYQVT-LS-CSCMSNSM--LASVARNFNQYRGSLNFLFVF
UniRef90_P12296_396_619   -----TQPLAVYQVT-LS-CSCLANTF--LAALSRNFAQYRGSLVYTFVF
UniRef90_C3U5A3_447_672   -----NVSLVTYQVT-LS-SINLANTM--LSSVARNFNQYRGSLNFLFVF
UniRef90_G1DFA4_460_684   ------EPLLQMDVT-LA-SDHMRYTA--LGQMSFRYAQYRGSINVMLTF
UniRef90_Q155Z9_448_665   ------RPLISFPIT-LS-DPVYQNTL--VGAISSNFANYRGCIQITLTF
UniRef90_B9VIN1_352_561   ------APVFQSNVV-LS-DPHYAHTL--VAGLGRYFGNYRGSLQFTLIS
UniRef90_G8HZ08_715_947   VT---KTKVFSLDIS-LS-AKYLANTY--VGMLAKMFQQYRGSLKYTFTY
UniRef90_B8XTQ3_353_566   ------TPLFKCNVL-LS-DPHFQHTL--VSNLGKYFANYRGSLQFSFIS
```

```
UniRef90_D9D8F3_345_562    ------RKLFSSGVV-LS-DPHYQHTL--LSNLARFFCNYRGSIQFDFVA
UniRef90_I1YLT4_348_565    ------TQLFISGVV-LS-DPHYQHTL--LSNMAQFFCNYRGSIQFDFVA
UniRef90_B8XTP9_335_563    ------EKLFSSGVV-LS-DKHYQHTL--LSNLADFFCNYRGSLQFDLVA
UniRef90_I1YLT7_349_565    ------TNVFMASVV-LS-DPHYQHTL--LSNMAQYFCNYRGSLQFDFVA
UniRef90_I1YLT6_336_558    ------SKLIRMNVV-LS-DPHFQHTL--LASLAEHFCNYRGSLQVTLLS
UniRef90_B8XTP8_334_562    ------RNLFKSSVV-LS-DLHYQHTL--VANLARYFCNYRGSLQFDFIA
UniRef90_B8XTQ0_353_567    ------SVLFSTGVI-LS-DPHYQHTL--VSNIAKYFCNYRGSIQFDFVA
UniRef90_Q5YJ95_381_603    NQ---TARIAAIDVS-MI-SPHLATTY--LSRLAQMYANYRGSVVFEFMF
UniRef90_B8XTQ4_341_566    -----QTAVFEMNVI-LS-EYALQRTF--VSMFGKFFCNYRGSIQITAWA
UniRef90_I1YLT5_349_558    ------GPLFKSNVV-LS-DLHYQHTL--VSQIGTFFGNYRGSLQFDLIA
UniRef90_F6IA11_421_636    -----NTTLYSLDVS-LL-ATEMEKTY--IGRLARMFAFYKGSIVLRFTY
UniRef90_G0Z2U8_412_599    NQ---TARIAAIDVS-MK-AGHLITTY--LSRLARMYANYRGSIVFEFMF
UniRef90_Q5YJA7_377_599    AQ---TARIAAIDVS-MV-STHLSTTY--LSRLAQMYANYRGSIVFEFMF
UniRef90_G0Y2E4_357_574    SV---GGKIKDFDMS-LN-STVLSTTY--LSKCARWYTHYRGSVNLTFMF
UniRef90_Q8B552_395_615    IR---TQAIGKWDMS-LI-GDLFKPTY--LGRLAKMYANYRGSVIIEFMF
UniRef90_A7KC13_333_552    ----QEQQVFGFSLF-LG-GGVLKTTL--LGEIAQYYTHWAGSIRISFMY
UniRef90_A5GZD6_337_514    ----QNQKIFGTDLY-IG-DGVFKTTL--LGEIAQYYTHWSGSLKISLMY
UniRef90_G5CZJ8_367_551    ----QEKQVFGFSIF-LG-GTVLKTTL--LGEIAQYYTHWAGSLRISFMY
UniRef90_G8HH17_373_575    PL---GENLGTWDLS-LQ-GNLLSPTY--LGRLSRFYTHYRGSINLTFMF
UniRef90_G0Y2E3_380_601    TE---DKPIRAWDMD-LA-SKLFTTTY--IGRVTKWFSQHRGSIRLTFLY
UniRef90_B9V482_337_556    ----KNEQIFGTNLY-IG-DGVFKTTL--LGEIVQYYTHWSGSLRISLMY
UniRef90_A5GZF6_334_555    -T--PAKEVFAIPVD-VA-SQPLATTL--IGEIASYYTHWTGSVRFSFMF
UniRef90_A7KC10_335_556    -Q--TAQEIFAIPVD-VA-SQPLATTL--IGEIASYYTHWTGSLRFSFMF
UniRef90_G8IJG2_334_555    -S--LAAEVFAIPVD-IA-SQPLATTL--IGEIASYYTHWTGSLRFSFMF
UniRef90_D2IW00_332_553    -A--PAREVFAIPVD-VA-SQPLATTL--IGEIASYYTHWTGSIRFSFMF
UniRef90_A5GZD9_331_550    ----QGQQVFGFRLY-LG-DGVLKTTM--LGEICQYFTHWAGSLRLSFMY
UniRef90_A7KC07_334_555    -D--VAKEVFAIPVD-VA-SQPLATTM--LGELSSYFTHWTGSLRFSFMF
UniRef90_Q672R3_805_1000   NS---GAQIFQMDMS-LM-SVNFESTY--LGQLARMYAQYRGDVIITLTF
UniRef90_B5A1N0_416_617    IQ---TTVLQTIDVS-LL-SDQMQTTY--LSRLAKQYANYRGSILLEFIF
UniRef90_B9V433_331_550    ----QGEQVFGFRLY-LG-DGVLKTTL--LGELCQYFTHWAGSLRLSFMY
UniRef90_Q82081_333_552    ----QNEQIFGTKLY-IG-DGVFKTTL--LGEIAQYYTHWSGSLRISLMY
UniRef90_Q5XLP5_336_557    -NT--AEQIFAIKVD-IA-SQPLSSTL--IGEIASYYTHWTGSLRFSFMF
UniRef90_B9V457_333_554    -D--FKKEIFAIPVD-VA-SQPLSTTL--IGEIANYYTHWTGSLRFSFMF
UniRef90_G1JYY8_404_619    AD---ENPIINWNLD-LN-GNELKDTY--LGRLCTMYANFRGSIRLSFTF
UniRef90_F6N707_334_556    GN--LATEIFAIPVD-IA-AQPLATTL--IGEIASYYTHWTGSIRFSFMF
UniRef90_G5D5D9_333_552    ----QNDQIFGTKLF-IG-DGVFKTTL--LGEIAQYYTHWSGSLRISLMY
UniRef90_Q82122_332_553    -KL--AEEIFAIKVD-IA-SHPLATTL--IGEIASYFTHWTGSLRFSFMF
UniRef90_G5D5E2_333_552    ----QGEMVFGTKLF-IG-DGVFKTTL--LGEIVQYYTHWAGSLRFSLMY
UniRef90_A5GZF5_335_556    -N--PAEEIFAIPVD-VA-SQPLATTL--IGEIASYYTHWTGSIRISFMF
UniRef90_G0Y2E2_378_600    SG---DGPIMEWDMD-LS-AVLFNTTY--LGRCSRWFSQHRGSIRLTFMF
UniRef90_P03303_333_552    ----QNEQVFGTNLF-IG-DGVFKTTL--LGEIVQYYTHWSGSLRFSLMY
UniRef90_P07210_338_559    -D--LAKEVFSIPVD-IA-SQPLATTL--IGELASYYTHWTGSLRFSFMF
UniRef90_F6M9Y3_333_554    -E--LKAEIFAIPVD-VA-SQPLATTL--IGEIASYYTHWTGSLRFSFMF
UniRef90_B9V459_336_557    -DF--AKEIFAIKVD-VA-SQPLSSTL--LGEICSYYTHWTGSLRFSFMF
UniRef90_B9V477_333_554    -T--SAGEVFAIPVD-IA-SQPLATTL--IGEIASYYTHWTGSIRFSFMF
UniRef90_H9C6N6_333_552    ----VNEQIFGTRLF-IG-DGVFKTTL--FGEIAQYYTHWSGSIRISLMY
UniRef90_A5GZE7_336_557    -NM--AEEIFAIKVD-IA-SQPLSNTI--IGEIASYFTHWTGSLRFSFMF
UniRef90_B9V485_333_552    ----QNEQIFGTNLF-IG-DGVFKTTL--LGEIAQYYTHWAGSLRFSLMY
UniRef90_G0Y2E0_419_588    VS---NAAIAHWDMD-MG-SVLFSTTY--MARCAKWYSQHRGGIRVTFMF
UniRef90_G9K138_332_516    -TT--AKEIFAIKVD-IA-SQPLATTM--IGEIASYFTHWTGSLRFSFLF
UniRef90_A5GZE9_336_557    -NL--AQEIFAIKVD-VA-SQPLSSTL--LGEICSYYTHWTGSLRFSFMF
UniRef90_B9V434_332_553    -QS--AVKLFTIPID-IA-SPPLNTTL--LGEIASYYTHWTGSIRLSFMF
UniRef90_P04936_332_552    -NA--PDKIFSIRTD-VA-SQPLATTL--IGEISSYFTHWTGSLRFSFMF
UniRef90_C7DUC8_336_557    -EM--AKEIFAIKVD-IA-SQPLANTM--IGEIASYFTHWAGSLRFSFMF
UniRef90_G8IQ74_332_548    ---T-GK-ILTLPLD-MS-HTVFATTL--LGEVLNYFSNWSGSVKLTFFC
UniRef90_I2CMI9_334_554    --NN-DGLILSIPLD-MS-NSLFATTL--LGEVLNYFTNWSGSITLTFMC
UniRef90_B9V466_338_522    -TP--AQKIFSIKVD-IA-SQPLANTM--LGQIASYYTHWTGSLRFSFMF
UniRef90_A5GZF9_333_554    -DL--AQEIFAIRVD-IA-SQPLATTL--IGEIASYYTHWTGSLRFSFMF
UniRef90_G8IJE2_332_552    -DP--DLKIFSLRTD-IA-SQPLATTL--IGEISNYFTHWTGSLRFSFMF
UniRef90_H8Y6Q0_168_390    SGTQ-PDLILTIPLK-MD-DTLFSTTL--LGEVLSYFSNWSGSITLSFMC
UniRef90_G0YPI0_327_543    TV---DTLCASFQVD-PGRDGPWQSTL--IGQLCRYYTQWSGSLEITFMF
UniRef90_B9V473_333_554    -T--LAEEVFAIPVD-VA-SQPLATTL--IGEISSYYTHWTGSIRFSFMF
UniRef90_B5BSU6_368_556    TSR--DEAIFAFQLD-PGHSSVLSRTL--LGEILNYYAHWSGSLKFTFLF
UniRef90_A8S322_365_548    ---A-DKPIVVIPMD-IS-NTLFSTTL--LGEVLNYFANWSGSISITFMC
UniRef90_G8IQ62_334_555    -DL--AQEIFAIRVD-IA-SQPLATTL--IGEIASYYTHWTGSLRFSFMF
UniRef90_B9V453_331_552    -TP--AAELFAMPVD-IT-SSPLATTL--LGEIASYYTNWTGSIRLSFMF
UniRef90_G9K146_333_520    -SL--AQEIFAIKVD-IA-SNPLATTL--IGEIASYFTHWTGSVRFSFLF
UniRef90_F6M9Z0_330_551    -EL--AKEIFAIKVD-IA-SQPLATTL--IGEIASYYTHWTGSLRFSFLF
UniRef90_P12916_334_555    -DM--AQEVFAIKVD-IT-SQPLATTL--IGEIASYYTHWTGSLRFSFMF
UniRef90_G5EN04_327_500    NV---DQLCASFQVD-PGRNGPWQSTM--VGQICRYYTQWSGSLKVTFMF
UniRef90_C7DUC6_357_551    --NN-GGLILTIPLE-MN-NTLFSTTL--LGEVLNYFTNWSGSITLTFMC
UniRef90_Q9WN78_367_554    NSG--EQ-VFGFQLQ-PGYDSVLKHTL--LGEILNYYANWSGSMKLTFMY
UniRef90_G0YPI1_326_499    AA---DKLCASFRVD-PGRSGPWESTL--LGQICRYYTQWSGSLEVTFMF
```

UniRef90_G0YPH9_326_499	AV---DTLCAAFRVD-PGRDGPWQSTM--VGQLCRYYTQWSGSLKITFMF
UniRef90_Q8UY33_326_552	AV---DQLCASFRVD-PGRAGPWEATL--VGQLCRYYTQWSGSLEVTFMF
UniRef90_A5GZE0_334_555	-DI--KEAIFAMRVD-IA-SQPLATTL--IGEIASYYTHWTGSLRFSFMF
UniRef90_G0ZNT7_334_555	P-NS-DGLILSIPLD-MS-NSLFSTTL--LGEILNYFTNWSGSITLTFMC
UniRef90_A7KC11_332_515	-P---GHEVFAMKVD-IA-SQPLATTI--IGEISSYYTHWTGSLRFSFMF
UniRef90_G1JYY7_238_455	SD---TDPITTWDLN-LA-GDSLRDTY--LGRLVKHYTNFRGSLVCSFIY
UniRef90_H6UKU5_326_499	EA---DKLCASFKVD-PGRDGPWEATT--VGQICRYYTQWSGSLEVTFMF
UniRef90_Q03053_367_554	TNG--SQ-VFGFPLI-PGASSVLNRTL--LGEILNYYTHWSGSIKLTFMF
UniRef90_Q995Z1_365_551	VS---QQ-VFGFKLQ-PGYDSVFKHTL--LGEILNYYANWSGSIKLTFMF
UniRef90_B9V442_331_555	-NITPAKEIFAIKVD-IA-SQPLATTL--IGEIANYYTHWTGSIRFSFLF
UniRef90_G1CSA8_329_506	SP---DTLCASFKVD-PGRDGPWQSTL--VGQICKYYTQWSGSLEVTFMF
UniRef90_A5GZE4_333_554	-QL--MQEVFAIRVD-VA-SQPLATTL--IGEISSYYTQWTGSLRFSFMF
UniRef90_G8IJF4_357_552	--TN-NGLILTIPLE-MS-NTLFSTTL--LGEILNYFTNWSGSVTLTFMC
UniRef90_A5GZF2_336_558	-DHPYAQEVFAIPVD-VA-SHPLATTY--LGEMSSCFTHWTGSLRFSFMF
UniRef90_A5GZF1_333_554	-SL--AQEIFAIKVD-IA-SDPLATTL--IGEIASYYTHWTGSIRFSFLF
UniRef90_Q5U904_326_555	AV---DALCAAFKVD-PGRDGPWQSTL--VGQICRYYTQWSGSLEVTFMF
UniRef90_Q6JKR7_325_551	EK---GKLCAVFRAD-PGLDGPWQSTL--LGQLCRYYTQWSGSLKLTFMF
UniRef90_G8IJF0_360_553	---N-DNVILVLPLE-MD-NTLFSTTL--LGEILNYFANWSGSITITFMC
UniRef90_A1E4A3_319_512	DV---DQLLFNIPLD-IQLDGPLRNTL--VGNISRYYTHWSGSLEMTFMF
UniRef90_Q8QUZ6_317_541	NP---DQQLFALEVT-FADGSIMSKTL--CGIITSYYTQWSGSLEFTFMF
UniRef90_Q2LL04_324_545	AL---DSQIYVLKLE-LGGSGPLSSTL--LGTLAKHFTQWSGSVEITCMF
UniRef90_F6M9Y7_364_585	-DP--AKEVFAIKVD-IA-SQPLATTL--IGEISSYYTHWTGSLRFSFLF
UniRef90_Q8V601_326_552	EV---DKLCASFRTD-PGRDGPWQSTL--VGQLCRYYTQWSGSLEVTFMF
UniRef90_Q6JKS0_326_499	DK---DTLCASLRVD-PGRDGPWQSTL--VGQICRYYTQWSGSLEITFMF
UniRef90_B3SRP1_326_550	SV---DQLCASFRVD-PGRDGPWQSTI--VGQLCRYYTQWSGSLEVTFMF
UniRef90_G5CZJ4_357_550	---D-NKLIVAIPLQ-MD-NTLFATTL--LGEVLNYYANWSGSVRVTFMC
UniRef90_B5BSU7_367_555	HSG--EGAIFAFQLD-PGSGSVLKHTL--LGEILNYYAHWSGSLRFTFLF
UniRef90_G8IRN3_333_554	SSRQ-EDLILVLPLK-MD-DTVFATTL--LGEVLNYFSNWSGSITLSFIC
UniRef90_A5GZE2_333_554	-DL--MQEVFSIRVD-IA-SQPLATTM--IGEIASYYTHWTGSLRFSFMF
UniRef90_A7KC08_333_554	-DS--AKEIFAIKVD-IA-SHPLATTL--IGEIASYYTHWTGSLRFSFLF
UniRef90_H9BP13_325_500	QS---GELCAAFKAD-PGLDGPWQSTM--VGQLCRYYTQWSGSLKITFMF
UniRef90_Q80GI2_332_555	SAQ--TTAIFTFQLD-PGHSPVLRRTL--LGEVLNYYAHWSGSIKLTFLF
UniRef90_B9V435_333_554	-DL--MSEVFAINVD-IA-SHPLATTM--LGEIASYYTHWTGSLRFSFMF
UniRef90_Q5G9Z5_326_554	AV---DQLCAAFRVD-PGRDGPWQSTL--VGQICKYYTQWSGSLEVTFMF
UniRef90_Q9YLG5_370_557	SQG--AQ-VFGFSLQ-PGASSVLQRTL--LGEILNYYTHWSGSLKLTFVF
UniRef90_G8IJE5_332_553	-DP--TTPVFTIRVD-IA-SQPLATTL--IGEISSYYTHWTGSVRFSFMF
UniRef90_Q8V614_326_499	NV---DELCCAFRVD-PGRNGPWESTI--IGQICRYYSQWSGSLEITFMF
UniRef90_Q5GA06_355_555	AV---DSLCAAFRVD-PGRDGPWQSTL--LGQLCRYYTQWSGSLEITFMF
UniRef90_G8IJG6_332_548	---E-GK-ILVIPLD-MS-NTLFATTL--LGEVLNYYSNWSGSIKLSFMC
UniRef90_Q05ED6_367_555	HPK--TKAIFSFPLD-PGNSSVMNRTL--LGEILNYYAHWSGSIKITFMF
UniRef90_P23008_310_531	-GM--AQKVFSIKVD-IT-STPLATTL--IGEIASYYTHWTGSLRFSFMF
UniRef90_Q8JRP9_365_551	TS---QQ-VFGFRVQ-PGYDSVFKHTL--LGEVLNYYANWSGSIKLTFMF
UniRef90_Q05EE3_367_555	HAS--SKAIFSFELD-PGNGTVLSKTL--LGEILNYYAHWSGSVKFTFLF
UniRef90_Q8V604_326_552	SV---DQLCASFRVD-PGRAGPWESTL--VGQYCKYYTQWSGSLEVTFMF
UniRef90_C7DUC7_331_549	---S-NDLIVVIPLQ-MD-NTLFATTL--LGEILNYYANWSGSIRVTFMC
UniRef90_P12915_324_545	AL---DAQIYALRLE-LGGSGPLSSSL--LGTLAKHYTQWSGSVEITCMF
UniRef90_G8IJE1_331_509	-P---GREVFAMRVD-IA-THPLATTI--IGEIANYYTHWTGSLRFSLMF
UniRef90_A4UHT9_332_548	---S-ERKILAIPLD-MS-NTLFSTTL--MGEVLNYYSNWSGSIKLTFFC
UniRef90_Q9QCF0_367_554	QLS--TQ-VFGFQMQ-PGLNSVFKRTL--LGEILNYYAHWSGSVKLTFVF
UniRef90_Q9QF31_325_500	KT---GELCAAFRAD-PGRDGPWQSTI--LGQLCRYYTQWSGSLEVTFMF
UniRef90_Q65900_325_551	KT---GELCAVFRAD-PGRNGPWQSTI--LGQLCRYYTQWSGSLEVTFMF
UniRef90_E5D8F2_334_553	---V-TERILVLPLE-MS-NTLFATTL--LGEVLNYYANWSGSITITFMC
UniRef90_A1E4A2_319_512	DM---DALLFNIPLD-IQLDGPLRSTL--LANIARYYTHWSGSMEMTFMF
UniRef90_B9V440_332_553	-SL--AEEVFAIKVD-IA-SQPLATTL--IGEMSSYYTHWTGSLRFSFMF
UniRef90_Q6W9F4_332_507	SSQ--TESVFAFQLD-PGHGSVLNRTL--LGEILNYYAHWSGSLKFTFLF
UniRef90_G8IJH5_359_554	-EL--MQEVFAIRVD-VA-SQPLATTL--IGEISSYYTQWTGSXRFSFMF
UniRef90_B1PZZ4_333_555	HQS--TQ-VFGFQVQ-PGLDNVFKHTL--LGEILNYYAHWSGSIKLTFVF
UniRef90_O89280_367_554	DTG--GQ-VFGFPLQ-PGASSVFQRTL--LGEILNYYTHWSGSIKLTFMF
UniRef90_P32537_321_545	DM---DQLLFNIPLD-IQLEGPLRNTL--LGNISRYYTHWSGSLEMTFMF
UniRef90_G0YPI2_326_499	KA---DELCAAFMVD-PGRIGPWQSTL--VGQICRYYTQWSGSLKVTFMF
UniRef90_Q9YLJ1_368_556	TVR--GDAIFSFQLN-PGNSPVLNRTL--LGEIINYYAHWSGSIKLTFLF
UniRef90_Q2YEN8_367_554	HQG--DK-IFAFQIQ-PGLDSVFKRTL--LGEVLNYYAHWSGSIKLTFTF
UniRef90_A7YF31_331_554	SRS--KDPIFSFQVD-PGFSEVLNRTL--LGEMLNYYAHWSGSIKLTFLF
UniRef90_Q65481_317_544	DM---DQQVMALRVD-PGTSGPLQSTL--LGVFSRYYTQWSGSIEFTFMF
UniRef90_Q2LKZ0_317_544	AM---DGQIMALRVD-PGIDGPMQSTL--LGVLHRYYAQWSGSLDFTFMF
UniRef90_Q05ED0_367_555	HET--TEAVFKFQLD-PGNDSVLNRTL--LGEILNYYAHWSGSIKFTFLF
UniRef90_Q8V5Z9_323_547	DL---DQQIASFRVD-PGADGPWQSTL--LGNICRYYNQWSGSLEITFMF
UniRef90_Q6W9F9_367_555	HSG--TEPIFSFQMD-PGSSTVMRHTL--TGEMLNYYAHWSGSVKLTFLF
UniRef90_H8Y6P9_356_549	---N-DRNLLILPLE-MD-NTLFSCTL--LGEVLNYFSNWSGSITITFMC
UniRef90_G5DC71_332_548	---T-ER-ILTIPLD-IS-NTIFSTTL--LGEVLNYFSNWSGSIKLTFFC
UniRef90_C6ZEZ9_341_560	---D-TDLILAMPLQ-MD-NTLFATTL--LGEVLNYFGNWSGSIKVTFMC
UniRef90_Q05ED4_367_554	TQG--GQ-VFGFEMQ-PGKETVFKRTL--VGEILNYYAHWSGSLKLTLTF
UniRef90_H6VBU4_358_522	--------LYAFPVR-LG-NSALRTTY--LGNMARFFSQYTGELKFSLMC

```
UniRef90_Q8V398_325_495    DV---DAQIAAFAVN-PGGDGVFQSTY--LGQICRYFNNWSGSIEITFMF
UniRef90_D8VCT6_342_524    PTK--DQSIFSLSLS-PASDERLSRTL--LGEILNYYTHWAGSIKFTFLF
UniRef90_D9YV24_514_704    PV---GTKLLCQKIDPISLTTPISGTLTPLGQTLTHFSQWRGTLDIDLVF
UniRef90_Q66577_367_555    VQD--KSAIFSFQLN-PGNSTVLRRTL--LGEILNYYAHWSGSIKLTFLF
UniRef90_A0MHB7_357_547    ---N-NR-ILAIPLD-MS-NTLFSTTL--MGEILNYFSNWSGSIKLTFLC
UniRef90_E9LS20_360_544    ---N-DERILVLPLE-MN-NTLFSTTL--LGEVLNYFTNWSGSITITFMC
UniRef90_Q9WHH4_367_554    HAG--DQ-VFGFKLG-PGSDAVLNRTL--LGEIMNYYTHWSGSIKLTFVF
UniRef90_G0ZNT5_357_550    ---D-NGLIVVIPLQ-MD-NTLFATTL--LGEVLNYYGNWSGSVRITFMC
UniRef90_Q5YJA3_454_673    ------GPIFQMPLR-PV-NEETTNTM--LGSVTRMFAQWRGSLVIRCVF
UniRef90_H9U0H0_456_678    ------NHIFSMPLR-PV-CEETMNTM--LGSVTRMFSQWRGSMVIRCVF
UniRef90_Q6W9E8_367_554    SSG--QQ-VFGFQLQ-PGSSSVLMRTL--LGEILNYYTHWSGSIKLTFVF
UniRef90_Q80GI1_367_556    DSE--TSALFSFQLD-PGRSTVLQRTL--LGEILNYYAHWSGSIKFTFMF
UniRef90_C7B298_377_565    SDL--NNPIFCLSLS-PASDQRLQFT
```

```
2WZR3                      TGPTNNKAKYMVAYIPPG--THPLPETPEMASHCYHAEWDTGLNSTFTFT
UniRef90_F1AW55_504_724    TGPTNNKAKYMVAYIPPG--TTPLPGTPETASHCYHAEWDTGLNSTFTFT
UniRef90_Q6PMU0_504_724    TGPTNNKAKYMVAYIPPG--THPLPETPEMASHCYHAEWDTGLNSTFTFT
UniRef90_Q6PMU2_504_724    SGPTNNKAKYMVAYIPPG--THPLPNTPEQASHCYHAEWDTGLNSTFTFT
UniRef90_Q8V0G8_305_525    TGPTNNKAKYMVAYIPPS--TNPLPTTPEAASHCYHAEWDTGLNSTFTFT
UniRef90_Q6PMT3_503_723    TGPTQSKARFMVAYIPPG--TSPVPDTPEKAAHCYHSEWDTGLNSKFTFT
UniRef90_Q1L748_304_524    TGPTQSKARFMVVYVPPG--TEPVPNTPEKAAHCYHSEWDTGLNSKFTFT
UniRef90_Q6PMT8_504_725    TGPTHHKAKFMVAYIPPGIETDKLPKTPEDAAHCYHSEWDTGLNSQFTFA
UniRef90_B8Y0J7_504_725    TGPTHHKAKFMVAYVPPGVSTEQLPKTPEDAAHCYHAEWDTGLNSSFSFA
UniRef90_Q1L757_305_524    TGPTHHKAKFMVAYIPPG--TDPLPETPEDAAHCHHAEWDTGLNSTFTFA
UniRef90_Q1L752_305_526    TGPTHHKAKFMVAYVPPGIAVAQLPKTPEDASHCYHSEWDTGLNSSFTFA
UniRef90_Q1L754_305_526    TGPTHHKAKFMVAYVPPGISASALPRTPEDAAHCYHSEWDTGLNSQFTFA
UniRef90_P49303_505_725    TGSTDSKARYMVAYVPPG--VETPPDTPEKAAHCIHAEWDTGLNSKFTFS
UniRef90_Q6PMY9_505_723    TGPTDAKARYMVAYIPPG--MT-PPTDPERAAHCIHSEWDTGLNSKFTFS
UniRef90_A2I8Z5_505_724    TGPTDAKARYMAAYAPPG--ME-PPKTPEAAAHCIHAEWDTGLNSKFTFS
UniRef90_P15072_505_723    TGPTDAKARYMVAYVPPG--MD-APDNPEEAAHCIHAEWDTGLNSKFTFS
UniRef90_Q8JVD0_304_524    TGPTQSKARFMVAYIPPG--TD-LFRHSRGSSTLLHSEWDTGLNSKFTFT
UniRef90_H9AY35_497_713    TGPVNEKARFRVVFVPPG--TT-TPNTAADASLLIHSDWDSGLNSEFVFP
UniRef90_B0Z3W9_528_746    TGFVSDKAKFLLVFVPPG--AD-PPTTLSEAQHCITLEWDTGLNSETVFN
UniRef90_Q2XP17_533_745    TGAAATKAKFLVAFVPPH--TA-APKTRDEAMACIHAVWDVGLNSAFSFN
UniRef90_Q100Y4_324_550    TGSAMAQGKFVVCYVPPG--R--EPTSLDEAMQGTYSIWDLGLNSSFKFV
UniRef90_Q91LX8_542_768    TGTAMAQGKFVVAYTPPG--R--EPKTLDEAMQGTYAIWDLGLNSSFKFV
UniRef90_A4ZKN2_415_639    TGSAMTKGKFLISYTPPG--AG-KPTTRDQAMQATYAIWDLGLNSSYNFT
UniRef90_E3TMG9_417_639    TGSAMTKGKFLISYTPPG--AG-EPKTLDQAMQATYAIWDLGLNSSYNFT
UniRef90_P08544_417_642    TGAAMVKGKFLIAYTPPG--AG-KPTTRDQAMQSTYAIWDLGLNSSFNFT
UniRef90_B3VTU7_418_646    TGTAVTKGKFLIAYTPPG--AG-KPTTREQAMQATYAIWDLGLNSSYNFT
UniRef90_Q66776_543_769    TGSAMCQGKFVVCYVPPG--R--EPQSLDEAMQGTYSIWDLGLNSSFKFV
UniRef90_G9FTJ7_414_638    TGSAMAKGKFLISYTPPG--AG-KPKDREQAMQATYAIWDLGLNSSYNFT
UniRef90_Q02472_417_641    TGSAMTKGKFLIAYTPPG--AG-KPTTRDQAXQSTYAIWDLGLNSSYNFT
UniRef90_P12296_396_619    TGTAMMKGKFLIAYTPPG--AG-KPTSRDQAMQATYAIWDLGLNSSYSFT
UniRef90_C3U5A3_447_672    TGSAMVKGKFLISXTPPG--AD-KPKNRNQAMQATYAIWDLGLNSSYNFT
UniRef90_G1DFA4_460_684    TGAAMVKGKFLLAYTPPG--AE-RPSTIEEAMQATYAIWDLGLQSSYDFT
UniRef90_Q155Z9_448_665    CGPMMARGKFLLSYSPPN--GT-QPQTLSEAMQCTYSIWDIGLNSSWTFV
UniRef90_B9VIN1_352_561    ATTSMTRGKLLCCYTPPG--AD-KPTTVEQAMMSTYAIWDLGLQSTFEFV
UniRef90_G8HZ08_715_947    CGSSMVTGKLLIAYTPPG--GT-EPTDREQAMLGTHMVWDIGLQSSVSFT
UniRef90_B8XTQ3_353_566    ATTGMTKGKLLLSYTPPG--SG-APTTLEQAMNGTYSIWDIGLQSTFNFV
UniRef90_D9D8F3_345_562    ATTALTRGKLLIAYSPPG--AN-EPTSIDQAMMGTYAIWDLGLQSTFNFV
UniRef90_I1YLT4_348_565    ATTAMTRGKLLLSYTPPG--AG-KPTTIDQAMMGTYSIWDLGLQSTFNFV
UniRef90_B8XTP9_335_563    VTTAMTRGKLLLAYTPPG--AG-EPTTIDQAMMGTYTIWDLGLQSTVNFV
UniRef90_I1YLT7_349_565    ATTAMTRGKLLISYTPPG--AN-QPESIDQAMMGTYSIWDLGLQSTFNFV
UniRef90_I1YLT6_336_558    CCTAMTRGKLLVAYTPPG--AG-APESIDQAMQGTYTIWDLGLQSSLNFT
UniRef90_B8XTP8_334_562    ATTAMTRGKLLISYTPPG--AG-EPQSIDQAMMGTYAIWDLGLQSTFNFV
UniRef90_B8XTQ0_353_567    ATTAMTRGKLLMAYTPPG--AN-KPTTIDQAMMGTFSIWDLGLQSTFNFV
UniRef90_Q5YJ95_381_603    CGSQMATGKLLIAYTPPG--GS-SPTTRTDAMLATHVIWDIGLQSTCKLV
UniRef90_B8XTQ4_341_566    AVTAMTRGKLLFSYTPPG--AG-KPQNIKQAMMGTYTIWDLGLQSTLNFT
UniRef90_I1YLT5_349_558    CTTAMTRGKLLLAYTPPG--AN-APETIEQAMMATYSIWDLGLQSTFSFV
UniRef90_F6IA11_421_636    TGPKQSSGKLLIAYTPPG--GA-RPTSREEAMLGTNIIWDFGLQSTCTFV
UniRef90_G0Z2U8_412_599    CGSQMATGKLLIAYTPPG--GS-SPTTRTDAMLATHVIWDIGLQSTCKLV
UniRef90_Q5YJA7_377_599    CGSQMATGKLLIAYTPPG--GS-SPATRTDAMLATHVVWDLGLQSTCKLV
UniRef90_G0Y2E4_357_574    CGSAMATGKILIAYTPPG--GD-APATRKDAMLATHVVWDLGLQSSVTFT
UniRef90_Q8B552_395_615    CGSKMATGKFLVAYTPPG--GS-SPATREEAMLGTHVIWDIGLQSTCKFV
UniRef90_A7KC13_333_552    TGPALSSAKLLLAYTPPG--AP-GPTNRKDAMLGTHIIWDIGLQSTIVMN
UniRef90_A5GZD6_337_514    TGPALSSAKLILAYTPPG--AP-GPETRKDAMLGTHVVWDIGLQSTIVMT
UniRef90_G5CZJ8_367_551    TGPALSSAKLLLAYTPPG--AP-GPKTRKDAMLGTHLVWDIGLQSTVVMN
UniRef90_G8HH17_373_575    CGSAMATGKFLISYTPPG--GD-APRTRQDAMLATHVVWDVGLQSSCSLV
UniRef90_G0Y2E3_380_601    TGSAMSTGKLLIAYTPPG--GS-EPKTRKDAMLGTHVVWDIGLQSSITMV
```

```
UniRef90_B9V482_337_556     TGPALSSAKLLIAYTPPG--AQ-GPTTKKDAXLGTHVVWDIGLQSTIVMT
UniRef90_A5GZF6_334_555     CGTANTSLKLLIAYTPPG--VP-KPTSRKDAMLSTHVVWDVGLQSTMSMV
UniRef90_A7KC10_335_556     CGTANTTLKLLIAYTPPG--VP-KPDTRKKAMLGTHLVWDVGLQSTVSLV
UniRef90_G8IJG2_334_555     CGTANTSLKLLIAYTPPG--VP-KPDSRQKAMLSTHVVWDVGLQSTVSLV
UniRef90_D2IW00_332_553     CGTANTSLKLLVAYTPPG--VP-KPDSRTKAMLGTHVVWDVGLQSTVSMV
UniRef90_A5GZD9_331_550     TGPALSSAKLLIAYTPPG--TA-GPADRRTAMLGTHVVWDIGLQSTVVLN
UniRef90_A7KC07_334_555     CGTANTTLKLLIAYTPPG--VP-KPDTRKKAMLGTHLVWDVGLQSTVSMI
UniRef90_Q672R3_805_1000    CGTAMTTGKILIAYTPPG--AN-PPADRTEAMLGTHVVWDIGLQSSVTMA
UniRef90_B5A1N0_416_617     CGSQMATGKLLIAYTPPG--GS-RPTTRQEASLGTNIIWDLGLQSTCKFP
UniRef90_B9V433_331_550     TGPALSSAKLLIAYTPPG--AQ-GPTKRKEAMLGTHVVWDIGLQSTVVLN
UniRef90_Q82081_333_552     TGPALSSAKIILAYTPPG--TR-GPEDRKKAMLGTHVVWDIGLQSTIVMT
UniRef90_Q5XLP5_336_557     CGTANTTLKLLLAYTPPG--ID-KPTTRKQAMLGTHIVWDVGLQSTVSLV
UniRef90_B9V457_333_554     CGTASTSLKLLIAYTPPG--VD-KPNTRTKAMLGTHVVWDVGLQSTVSMV
UniRef90_G1JYY8_404_619     AGSHMATGKLLLCYSPPG--AN-PPKTRDEAMLGTSLLWDIGLQSTVNFV
UniRef90_F6N707_334_556     CGTANTKVKLLIAYTPPG--VE-KPTSRTKAMLGTHLVWDVGLQSTVSMV
UniRef90_G5D5D9_333_552     TGPALSSAKLILAYTPPG--AQ-GPTNRKDAMLGTHVVWDIGLQSTIVMT
UniRef90_Q82122_332_553     CGTANTTLKVLLAYTPPG--IG-KPRSRKEAMLGTHVVWDVGLQSTVSLV
UniRef90_G5D5E2_333_552     TGPALSSAKLILSYTPPG--AN-GPTTRKEAMLGTHVVWDIGLQSTIVMT
UniRef90_A5GZF5_335_556     CGSANTSLKLLIAYTPPG--VP-KPTSRKEAMLGTHLVWDVGLQSTASMV
UniRef90_G0Y2E2_378_600     CGSAMTTGKLLLAYTPPG--GA-APATRKEAMLGTHVVWDIGLQSSITMV
UniRef90_P03303_333_552     TGPALSSAKLILAYTPPG--AR-GPQDRREAMLGTHVVWDIGLQSTIVMT
UniRef90_P07210_338_559     CGSASSTLKLLIAYTPPG--VG-KPKSRREAMLGTHLVWDVGLQSTASLV
UniRef90_F6M9Y3_333_554     CGTANTSLKLLIAYTPPG--VA-KPTTRTMAMLGTHLVWDVGLQSTVSMV
UniRef90_B9V459_336_557     CGTANTTLKVLIAYTPPG--IK-KPETRKQAMLGTHIVWDVGLQSTVSLV
UniRef90_B9V477_333_554     CGSANTSLKLLIAYTPPG--VP-KPTSRREAMLGTHLVWDVGLQSTASMV
UniRef90_H9C6N6_333_552     TGPALSSAKLILAYTPPG--TE-GPKTRREAMLGTHVVWDIGLQSTVVMN
UniRef90_A5GZE7_336_557     CGTANTTLKLLLAYTPPG--IK-KPTNRKQAMLGTHIVWDIGLQSTVSLV
UniRef90_B9V485_333_552     TGPALSSAKLILAYTPPG--VA-GPKNRKEAMLGTHVVWDIGLQSTIIMN
UniRef90_G0Y2E0_419_588     CGSAMATGKLLLAFTPPG--GS-APATRRDAMLGTHEIWDLGLQSSITLV
UniRef90_G9K138_332_516     CGTANTTLKLLVAYTPPG--IE-KPQNRKDAMLGTHVVWDVGLQSTISMV
UniRef90_A5GZE9_336_557     CGTANTTLKLLLAYTPPG--IK-KPENRKQAMLGTHIVWDVGLQSTISLV
UniRef90_B9V434_332_553     CGSANSSLKLLIAYTPPG--AP-EPTKRKEAMLGTHIVWDVGLQSTCSLI
UniRef90_P04936_332_552     CGTANTTVKLLLAYTPPG--IA-EPTTRKDAMLGTHVIWDVGLQSTISMV
UniRef90_C7DUC8_336_557     CGTANTTLKLLLAYTPPG--IG-KPRDRKQAMLGTHIVWDVGLQSTVSLV
UniRef90_G8IQ74_332_548     VCNSFSTGKFLIAYTPPG--AG-IPETRKDAMLGTHVVWDLGLQSSCHLI
UniRef90_I2CMI9_334_554     VCDSFSTGKFLLAYTPPG--GA-HPANRKQAMLGVHVVWDLGLQSSCTMV
UniRef90_B9V466_338_522     CGTANTTLKLLIAYTPPG--IG-EPRDRKQAMLGTHIVWDVGLQSTVSLV
UniRef90_A5GZF9_333_554     CGSASATLKLLIAYTPPG--IA-KPTTRKQAMLGTHVVWDVGLQSTISMI
UniRef90_G8IJE2_332_552     CGTANTTVKLLLAYTPPG--ID-EPRTRKDAMLGTHVIWDIGLQSTISMV
UniRef90_H8Y6Q0_168_390     VCDSFSTGKFLIAYTPPG--GG-HPSDRKEAMLGVHVIWDLGLQSTCTMV
UniRef90_G0YPI0_327_543     TGSFMATGKMLIAYTPPG--SA-QPTTRELAMLGTHIIWDFGLQSSVTLV
UniRef90_B9V473_333_554     CGTANTSLKLLIAYTPPG--VG-KPQNRTQAMLGTHMIWDVGLQSTVSMV
UniRef90_B5BSU6_368_556     CGSAMATGKILLSYSPPG--AK-VPEKRKDAMLGTHIIWDLGLQSSCVLC
UniRef90_A8S322_365_548     VCDSFSTGKFLLAYTPPG--AA-LPPDRKTAMLGVHVIWDLGLQSSCTIV
UniRef90_G8IQ62_334_555     CGSASTTLKLLLAYTPPG--IA-KPTTRKDAMLGTHVVWDVGLQSTISMI
UniRef90_B9V453_331_552     CGSANTSLKLLLAYTPPG--VS-KPTSRREAMLGTHLVWDVGLQSTCSLV
UniRef90_G9K146_333_520     CGTANTTLKLLVAYTPPG--IR-KPTSRKEAMLGTHVVWDVGLQSTMSMV
UniRef90_F6M9Z0_330_551     CGSANTTLKLLIAYTPPG--IS-KPTKRKEAMLGTHVVWDVGLQSTMSMV
UniRef90_P12916_334_555     CGTANTTLKLLLAYTPPG--ID-KPATRKDAMLGTHVVWDVGLQSTISLV
UniRef90_G5EN04_327_500     TGSFMATGKMLIAYTPPG--SA-QPATREAAMLGTHIVWDFGLQSSVTLV
UniRef90_C7DUC6_357_551     VCDSFSTGKFLLAYTPPG--GA-HPKDRKEAMLGVHVIWDLGLQSSCTMV
UniRef90_Q9WN78_367_554     CGAAMATGKFLIAYSPPG--AG-VPGSRKDAMLGTHVIWDVGLQSSCVLC
UniRef90_G0YPI1_326_499     TGSFMATGKMLIAYTPPG--GE-QPKTRDVAMLGTHVIWDFGLQSSVTLV
UniRef90_G0YPH9_326_499     TGSFMATGKMLVAYTPPG--GA-QPASRELAMLGTHVIWDFGLQSSVTLV
UniRef90_Q8UY33_326_552     TGSFMATGKMLIAYTPPG--GA-QPTSREVAMLGTHVIWDFGLQSSVTMI
UniRef90_A5GZE0_334_555     CGSAFTTLKLLIAYTPPG--ID-VPSTRKQAMLGTHVIWDVGLQSTVSMV
UniRef90_G0ZNT7_334_555     VCDSFSTGKFLLAYTPPG--GA-HPKTRKEAMLGVHVIWDLGLQSSCTMV
UniRef90_A7KC11_332_515     CGSAGTTLKLLIAYTPPG--ID-KPTKRKDAMLGTHVVWDVGLQSTISMI
UniRef90_G1JYY7_238_455     TGSAMSTGKILVCYSPPG--AG-VPQSRTEAMLGTNVLWDLGLQSTLEFN
UniRef90_H6UKU5_326_499     TGSFMATGKMLIAYTPPG--GQ-QPATRDIAMLGTHVIWDFGLQSSVTLV
UniRef90_Q03053_367_554     CGSAMATGKFLLAYSPPG--AG-APTTRKEAMLGTHVIWDVGLQSSCVLC
UniRef90_Q995Z1_365_551     CGSAMTTGKLILAYSPPG--AG-VPGTRKDAILGTHVVWDLGLQSSCVLC
UniRef90_B9V442_331_555     CGTANTTLKLLVAYTPPG--IK-KPENRKDAMLGTHVVWDVGLQSTISMV
UniRef90_G1CSA8_329_506     TGSFMATGKMLIAYTPPG--GA-QPTTRDLAMLGTHVIWDFGLQSSITLV
UniRef90_A5GZE4_333_554     CGTANTTLKLLIAYTPPG--IV-KPRNRKEAMLGTHIVWDVGLQSTISMI
UniRef90_G8IJF4_357_552     VCDSFSTGKFLLAYTPPG--GA-HPKNRKEAMLGVHIIWDLGLQSSCTMV
UniRef90_A5GZF2_336_558     CGSACTTLKLLIAYTPPG--IA-KPTSRKEAMLGTHLVWDVGLQSTASLV
UniRef90_A5GZF1_333_554     CGSANTTLKLLIAYTPPG--IR-KPKNRKEAMLGTHVVWDVGLQSTISMV
UniRef90_Q5U904_326_555     TGSFMATGKMLIAYTPPG--AP-QPANRRIAMLGTHVIWDFGLQSSVTLV
UniRef90_Q6JKR7_325_551     TGSFMATGKMLIAYTPPG--GV-TPASRMDAMLGTHIIWDFGLQSSVSLV
UniRef90_G8IJF0_360_553     VCDAFSTGKFLLAYTPPG--GR-LPLSRKEAMLGVHVIWDLGLQSSCTIV
UniRef90_A1E4A3_319_512     CGSFMATGKLILCYTPPG--GS-CPTTRETAMLGTHIVWDFGLQSSVTLI
UniRef90_Q8QUZ6_317_541     TGSFMTTGKLLLAYSPPG--GS-APTTRDDAMLGTHVVWDFGLQSSITLV
```

```
UniRef90_Q2LL04_324_545   TGTFMTTGKVLLAYTPPG--GD-MPRNREEAMLGTHVIWDFGLQSSITLV
UniRef90_F6M9Y7_364_585   CGSANTTLKLLVAYTPPG--IA-KPTDRKAAMLGTHVVWDVGLQSTISMV
UniRef90_Q8V601_326_552   TGSFMATGKLLIAYTPPG--AP-QPATRAQAMLGTHIVWDFGLQSSATLV
UniRef90_Q6JKS0_326_499   TGSFMATGKMLIAYSPPG--SA-QPTTRDLAMLGTHVVWDFGLQSSITLV
UniRef90_B3SRP1_326_550   TGSFMATGKLLIAYTPPG--GT-QPTSRAVAMLGTHVIWDFGLQSSITLV
UniRef90_G5CZJ4_357_550   VCDSFSTGKFLLAYTPPG--GR-LPRDRKEAMLGTHVVWDLGLQSSCTMV
UniRef90_B5BSU7_367_555   CGTAMTTGKLLIAYSPPG--AN-VPENRKDAMLGTHMVWDVGLQSSCVLC
UniRef90_G8IRN3_333_554   VCDSFSTGKFLIAYTPPG--GA-LPTDRKDAMLGVHVIWDLGLQSTCTIA
UniRef90_A5GZE2_333_554   CGTANTTLKLLLAYTPPG--IA-KPSNRKEAMLGTHVVWDVGLQSTISMI
UniRef90_A7KC08_333_554   CGTACTTLKLLIAYTPPG--IR-KPENRREAMLGTHVVWDVGLQSTISMV
UniRef90_H9BP13_325_500   TGSFMATGKMLIAYTPPG--GS-LPANRMQAMLGTHVIWDFGLQSSVTLV
UniRef90_Q80GI2_332_555   CGSAMATGKILLAYSPPG--AG-VPTKRKDAMLGTHVIWDIGLQSSCVLC
UniRef90_B9V435_333_554   CGSASTTLKLLLAYTPPG--IA-KPISRRDAMLGTHVVWDVGLQSTISMV
UniRef90_Q5G9Z5_326_554   TGSFMATGKMLIAYTPPG--GP-KPANRETAMLGTHVIWDFGLQSSVTLV
UniRef90_Q9YLG5_370_557   CGSAMATGKFLLAYSPPG--AG-VPPDRKKAMLGTHVIWDVGLQSSCVLC
UniRef90_G8IJE5_332_553   CGSALTTLKLLIAYTPPG--IR-VPRNRKEAMLGTHLIWDVGLQSTVSMV
UniRef90_Q8V614_326_499   TGSFMATGKMLIAYTPPG--GA-QPSNRTTAMLGTHVIWDFGLQSSVTLV
UniRef90_Q5GA06_355_555   TGSFMATGKMLIAYTPPG--GA-QPTSRSLAMLGTHVIWDFGLQSSVTLV
UniRef90_G8IJG6_332_548   VCDSFSTGKFLIAYTPPG--AG-LPTDRKQAMLGVHTIWDLGLQSTCNMV
UniRef90_Q05ED6_367_555   CGTAMATGKILLSYSPPG--AS-VPESRTQAMLGTHVIWDLGLQSSCVLC
UniRef90_P23008_310_531   CGTANTTLKLLLAYTPPG--ID-EPTTRKDAMLGTHVVWDVGLQSTISLV
UniRef90_Q8JRP9_365_551   CGSAMTTGKLILAYSPPG--AG-VPDSRKNAMLGTHVVLDLGLQSSCVLC
UniRef90_Q05EE3_367_555   CGSAMATGKILLAYSPPG--AS-VPKDRKQAMLGTHVIWDLGLQSSCVLC
UniRef90_Q8V604_326_552   TGSFMATGKMLIAYTPPG--GA-QPANRRTAMLGTHVIWDFGLQSSVTLV
UniRef90_C7DUC7_331_549   VCDSFGTGKSLMAYTPPG--GK-LPTSRKEAMLGVHVIWDLGLQSSCTMV
UniRef90_P12915_324_545   TGTFMTTGKVLLAYTPPG--GD-MPRNREEAMLGTHVVWDFGLQSSITLV
UniRef90_G8IJE1_331_509   CGTAGTTLKLLVAYTPPG--IA-KPTNRKDAMLGTHVVWDVGLQSTISMI
UniRef90_A4UHT9_332_548   VCDSFSTGKFLIAYTPPG--AD-IPESRRDAMLGTHVVWDLGLQSSCHLV
UniRef90_Q9QCF0_367_554   CGSAMALAKFLLAYSPPG--AD-PPKSRKEAMLGTHVIWDIGLQSSCVLC
UniRef90_Q9QF31_325_500   AGSFMATGKMLIAYTPPG--GN-VPADRITAMLGTHVIWDFGLQSSVTLV
UniRef90_Q65900_325_551   AGSFMATGKMLIAYTPPG--GG-VPADRLTAMLGTHVIWDFGLQSSVTLV
UniRef90_E5D8F2_334_553   VCDAFSTGKFLVAYTPPG--GK-LPEDRKQAMLGVHIIWDLGLQSSCTIV
UniRef90_A1E4A2_319_512   CGSFMATGKVILCYTPPG--GS-CPTDRESAMLGTHIVWDFGLQSSITLV
UniRef90_B9V440_332_553   CGTAGTTLKLLLAYTPPG--IA-KPQKRKDAMLGTHVVWDVGLQSTISMI
UniRef90_Q6W9F4_332_507   CGSAMATGKLLLAYSPPG--AD-VPKSRKDAMLGTHIVWDLGLQSSCVLC
UniRef90_G8IJH5_359_554   CGTANTTLKLLIAYTPPG--IA-KPKSRKEAMLGTHIVWDVGLQSTISMI
UniRef90_B1PZZ4_333_555   CGSAMATGKFLLAYAPPG--AN-APKSRKDAMLGTHIIWDVGLQSSCVLC
UniRef90_O89280_367_554   CGSAMATGKFLLAYSPPG--AG-APKSRKDAMLGTHVIWDVGLQSSCVLC
UniRef90_P32537_321_545   CGSFMTTGKLIICYTPPG--GS-SPTDRMQAMLATHVVWDFGLQSSITII
UniRef90_G0YPI2_326_499   TGSFMATGKMLVAYSPPG--SA-QPANRETAMLGTHVIWDFGLQSSVSLV
UniRef90_Q9YLJ1_368_556   CGSAMATGKLLLAYSPPG--AS-VPTSRKDAMLGTHIIWDLGLQSSCVLC
UniRef90_Q2YEN8_367_554   CGSAMATGKLLLAYSPPG--AD-VPASRKQAMLGTHIIWDIGLQSSCVLC
UniRef90_A7YF31_331_554   CGSAMATGKLLLAYSPPG--AS-VPTTRKDAMLGTHIIWDLGLQSSCVLC
UniRef90_Q65481_317_544   CGTFMSTGKVIIAYTPPG--GT-APTSRREAMLGTHIVWDFGLQSSITLV
UniRef90_Q2LKZ0_317_544   CGTFMTTGKVIIAYTPPG--GD-QPTARRQAMLGTHVVWDFGLQSSITLV
UniRef90_Q05ED0_367_555   CGSAMATGKLLIAYSPPG--AN-PPTDRKQAMLGTHIIWDLGLQSSCVLC
UniRef90_Q8V5Z9_323_547   TGSFMATGKILIGYSPPG--GS-QPATREDAMLGTYVVWDFGLQSSVTLA
UniRef90_Q6W9F9_367_555   CGSAMATGKLLLAYSPPG--AS-VPTSRKDAMLGTHIIWDVGLQSSCVLC
UniRef90_H8Y6P9_356_549   VCDSFSTGKFLVAYTPPG--GK-PPTDRKDAMLGVHIIWDLGLQSSCSIV
UniRef90_G5DC71_332_548   VCDSFSTGKFLIAYTPPG--AD-IPTKRKDAMLGTHVIWDLGLQSSCHLV
UniRef90_C6ZEZ9_341_560   VCDSFSSGKFLVAYTPPG--GG-VPTNRKEAMLGTHLIWDLGLQSSCTLV
UniRef90_Q05ED4_367_554   CGSAMATGKFLVAYSPPG--AN-PPKNRKDAMLGTHIIWDVGLQSSCVLC
UniRef90_H6VBU4_358_522   CANSMTKGRLLISFLPGV--VT-RPNNINQAMLGSYVVYDVGLNSTFEFP
UniRef90_Q8V398_325_495   CGSFMATGKILIAYTPPG--GS-APTDRKSAMLGTHIIWDFGLQSSATLV
UniRef90_D8VCT6_342_524   CGSMMATGKILISYSPPG--AA-PPTTRKDAMLGTHVIWDIGLQSSVTLV
UniRef90_D9YV24_514_704   SGSQQNTGRILVSTTPPV--AG-CPATMEAAMRNTCYIWDLTTSASISIT
UniRef90_Q66577_367_555   CGSAMATGKLLLAYSHAG--AS-VPKSRRDAMLGTHVIWDVGLQSSCVLC
UniRef90_A0MHB7_357_547   VCDSFSTGKFLIAYTPPG--AS-IPANRTDAMLGTHIIWDLGLQSSCHMV
UniRef90_E9LS20_360_544   VCDSFSTGKFLLAYTPPG--GA-HPKDRKQAMLGVHVIWDLGLQSSCTMV
UniRef90_Q9WHH4_367_554   CGSAMTTGKFLLSYAPPG--AG-APTKRKDAMLGTHVVWDVGLQSSCVLC
UniRef90_G0ZNT5_357_550   VCDSFSTGKFLMAYTPPG--GR-LPADRKEAMLGTHVVWDLGLQSSCSMV
UniRef90_Q5YJA3_454_673   VGNQMQTCRLLVAWTPQGFNAPV-PSKMAEAMQGHYVIYDTGVDSSVDLV
UniRef90_H9U0H0_456_678   VGNQMQNCRLVMAWTPQGFGTTEAPRTMEQAMQGHYVIYDTGIDSSVDLV
UniRef90_Q6W9E8_367_554   CGSAMATGKFLLAYSPPG--AG-KPMSRKDAMLGTHVVWDVGLQSSCVLC
UniRef90_Q80GI1_367_556   CGSAMATGKILLSYSPPG--AK-APSSRKDAMLGTHVIWDIGLQSSCVLC
UniRef90_C7B298_377_565

2WZR3                     VPYFSAADYA--YT-----------YADE---PEQASVQGWVGVYQITDT
UniRef90_F1AW55_504_724   VPYVSAADFA--YT-----------YSNE---PEQTSVQGWVGVYQITDT
UniRef90_Q6PMU0_504_724   VPYVSAADFA--YT-----------YSDE---PEQASVQGWVGVYQVTDT
```

```
UniRef90_Q6PMU2_504_724   VPYVSGADFA--YT-----------HADE---PEQASVQGWVGVYQITDT
UniRef90_Q8V0G8_305_525   VPYISAADYA--YT-----------YADE---PEQASVQGWIGVYQVTDT
UniRef90_Q6PMT3_503_723   VPYMSAADFA--YT-----------YCDE---PEQASAQGWVTLYQITDT
UniRef90_Q1L748_304_524   VPYVSGADYA--YT-----------YCDE---PETTSAQGWVTLYQITDT
UniRef90_Q6PMT8_504_725   VPYVSASDFS--YT-----------HTDT---PAMATTNGWVAVYQVTDT
UniRef90_B8Y0J7_504_725   VPYISASDFT--YT-----------HTDT---PAMATTNGWVVVLQVTDT
UniRef90_Q1L757_305_524   VPYMSASDYT--FT-----------HTDT---PAMATTNGWVAVYQVTDT
UniRef90_Q1L752_305_526   VPYISSADFS--YT-----------HTDT---PAMATTNGWVVVLQVTDT
UniRef90_Q1L754_305_526   VPYVSASDFS--YT-----------HTDT---PAMATTNGWVVVLQVTDT
UniRef90_P49303_505_725   IPYVSAADYA--YT-----------ASDV---AETTNVQGWVCIYQITHG
UniRef90_Q6PMY9_505_723   IPYLSAADYA--YT-----------ASDV---AETTSVQGWVCIYQITHG
UniRef90_A2I8Z5_505_724   IPYLSAADYA--YT-----------ASDV---AETTNVQGWVCLFQITHG
UniRef90_P15072_505_723   IPYISAADYA--YT-----------ASHE---AETTCVQGWVCVYQITHG
UniRef90_Q8JVD0_304_524   GQPTWRADFA--YT-----------YCDE---PEQASAQGWVTVYQITDT
UniRef90_H9AY35_497_713   VPYVSPTPYT--TT-----------HGTD---ADQATVNGWIQIYQL-DA
UniRef90_B0Z3W9_528_746   IPYISQTYYT--ST-----------HSST---ADIGNVSGRVQIYQVT-A
UniRef90_Q2XP17_533_745   VPYSSPSDFM--AV-----------YS-A--DATVVNVSGWLQVYALTAL
UniRef90_Q100Y4_324_550   IPYISASAYR--FT-----------HEDD--RPSVVNAVGWLQIYQLTTL
UniRef90_Q91LX8_542_768   IPYISASAYR--FT-----------NEDD--RPSVVNAVGWLQVYQLTTL
UniRef90_A4ZKN2_415_639   VPFISPTHYR--QT-----------SY-T--TPSITSVDGWLSVWQLTPL
UniRef90_E3TMG9_417_639   VPFISPTHYR--QT-----------SY-N--TPTITSVDGWLTVWQLTPL
UniRef90_P08544_417_642   APFISPTHYR--QT-----------SY-T--SPTITSVDGWVTVWKLTPL
UniRef90_B3VTU7_418_646   VPFISPTHYR--QT-----------SY-T--STSITSVDGWLTVWQLTPL
UniRef90_Q66776_543_769   VPYISASAYR--FT-----------HEDD--QPSALNAVGWLQIYQLTNL
UniRef90_G9FTJ7_414_638   VPFISPTHYR--QT-----------SY-T--TPSITSVDGWLSIWQLTPL
UniRef90_Q02472_417_641   VPFISPSHYR--QT-----------SY-T--SPSIAAVDGWLTVWQLTPL
UniRef90_P12296_396_619   VPFISPTHFR--MV-----------GT-D--QANITNVDGWVTVWQLTPL
UniRef90_C3U5A3_447_672   VPFISPTHFR--QT-----------SY-T--TSTITSVDGWISVWQLTPL
UniRef90_G1DFA4_460_684   IPFISVSDFR--LT-----------AS-G--IPSTISVDGWFTVFQYTAL
UniRef90_Q155Z9_448_665   VPYISPSDYR--ET-----------RA-I--TNSVYSADGWFSLHKLTKI
UniRef90_B9VIN1_352_561   VPFISTVDFR--VN-----------VV-S--TSSAVNVDGWFSIFKLNAL
UniRef90_G8HZ08_715_947   VPYISVSQYR--MT-----------SG----ENTAISVCGWISMRYQTSF
UniRef90_B8XTQ3_353_566   VPYISAVDFR--LC-----------AA-S--ASSVVNTDGWFTVWLLNSL
UniRef90_D9D8F3_345_562   VPFISATDFR--YN-----------QV-A--IASASNSDGWLTVWMLNPL
UniRef90_I1YLT4_348_565   VPFISASDFR--YN-----------SV-S--VSSALNSDGWLSVWLLNPL
UniRef90_B8XTP9_335_563   VPFISASDFR--YN-----------SV-S--VSSALNSDGWFTVWLLNPL
UniRef90_I1YLT7_349_565   VPFISACDFR--YN-----------TV-S--VSSALNSNGWISIWLMTPL
UniRef90_I1YLT6_336_558   IPFISAVDFR--LN-----------SS-A--RSSVLNSDGWFSVWLMSPL
UniRef90_B8XTP8_334_562   VPFISASDFR--FN-----------TS-S--VSNALNSDGWITVWLMNPL
UniRef90_B8XTQ0_353_567   VPFISATDFR--YN-----------LV-S--VNSALNSDGWFTVWLLNPL
UniRef90_Q5YJ95_381_603   VPYISSSQYR--QN-----------NV----DQTVLSYNGWVTVFQQTAL
UniRef90_B8XTQ4_341_566   IPYISSVDFR--IN-----------SR-A--VASALNADGWLTIWILNPI
UniRef90_I1YLT5_349_558   VPFVSTVDFR--VN-----------NV-S--ISSAVNADGWFTIFLLNPL
UniRef90_F6IA11_421_636   IPYISISSRR--FA-----------NT----TGTIFSYDGYVTVWYQTAV
UniRef90_G0Z2U8_412_599   VPYISSSQYR--QN-----------NV----DQTTLSYNGWVTIFQQTAL
UniRef90_Q5YJA7_377_599   VPYISSSQYR--QN-----------NL----TNTTLSYDGWVTIFQQTAL
UniRef90_G0Y2E4_357_574   VPYISQSQYR--YN-----------NI----DGNIFSYDGFISMFYQTNI
UniRef90_Q8B552_395_615   IPYISTSVYR--SS-----------ML----TNTTLSYDGWITMWYQTAI
UniRef90_A7KC13_333_552   IPWTAGSQFR--YT-----------DPDT------YTHPGFISCWYQTSL
UniRef90_A5GZD6_337_514   IPWTSGVQFR--YT-----------DKD----------------------
UniRef90_G5CZJ8_367_551   IPWTSGSQFR--YT-----------DPDT------YTHPGYISCWYQTSL
UniRef90_G8HH17_373_575   IPYISQSQYR--FS-----------NI----SGNKLSYDGYITLWYQTAV
UniRef90_G0Y2E3_380_601   VPWVSQTVYR--YN-----------HPK---EENALTYRGFVTVFYQTAL
UniRef90_B9V482_337_556   IPWTSGVQFR--YT-----------DKDT------YTSAGFLSCWYQTSL
UniRef90_A5GZF6_334_555   VPWVSASHYR--YT-----------TPDT------YSKAGYITCWYQTNF
UniRef90_A7KC10_335_556   VPWVSASHFR--FT-----------TPDT------YSKAGYITCWYQTNF
UniRef90_G8IJG2_334_555   VPWISASHYR--YT-----------TPDT------YSKAGYITCWYQTNF
UniRef90_D2IW00_332_553   VPWVSASHYR--LT-----------TPDT------YSKAGYITSWYQTNF
UniRef90_A5GZD9_331_550   IPWTSGVQFR--YT-----------DPDT------YTSAGFVSCWYQTSL
UniRef90_A7KC07_334_555   VPWVSASHFR--FT-----------TPDT------YSKAGYITCWYQTNF
UniRef90_Q672R3_805_1000  IPFISTVQAR--YT-----------GV----EESTLSYCGYITAWLQTKL
UniRef90_B5A1N0_416_617   IPYISSSQYR--QT-----------NV----RETILSYNGWVTIWYQTAI
UniRef90_B9V433_331_550   IPWTSGVQYR--YT-----------DPDT------YTSAGFVSCWYQTSL
UniRef90_Q82081_333_552   IPWTSGVQFR--YT-----------DPDT------YTSAGYLSCWYLTSL
UniRef90_Q5XLP5_336_557   VPWVSASHFR--YT-----------TPDT------YSMAGYITCWYQTNF
UniRef90_B9V457_333_554   VPWISASHYR--FT-----------TPDT------YSQAGYITCWYQTNF
UniRef90_G1JYY8_404_619   IPYISMSQFR--TT-----------VG----DTGFLSSSGTMTMWYQTSV
UniRef90_F6N707_334_556   VPWISASNYR--FT-----------TPDT------YSQAGYITCWYQTNF
UniRef90_G5D5D9_333_552   IPWTSGVQFR--YT-----------DPDT------YTSAGYLSCWYQTSL
UniRef90_Q82122_332_553   VPWISASQYR--FT-----------TPDT------YSSAGYITCWYQTNF
UniRef90_G5D5E2_333_552   IPWTSGVQFR--YT-----------DPDT------YTSAGFLSCWYQTSL
UniRef90_A5GZF5_335_556   IPWVSASHYR--FT-----------TPDI------YTLAGYVTCWYQTNL
UniRef90_G0Y2E2_378_600   VPWVSQTVYR--YN-----------HPK---EENVLTYRGFLSVFHQTAL
```

```
UniRef90_P03303_333_552  IPWTSGVQFR--YT-----------DPDT------YTSAGFLSCWYQTSL
UniRef90_P07210_338_559  VPWVSASHFR--FT-----------TPDT------YSSAGYITCWYQTNF
UniRef90_F6M9Y3_333_554  VPWVSASHYR--FT-----------TPDT------YSQAGYITCWYQTNF
UniRef90_B9V459_336_557  VPWVSASHFR--YT-----------TPDT------YSAAGYITCWYQTNF
UniRef90_B9V477_333_554  VPWVSASHYR--FT-----------TPDM------YSLAGYITCWYQTNL
UniRef90_H9C6N6_333_552  IPWTSGVQFR--YT-----------DPDT------YTSAGHLSCWYQTSL
UniRef90_A5GZE7_336_557  VPWVSASHFR--YT-----------TPDT------YSAAGYITCWYQTNF
UniRef90_B9V485_333_552  IPWTSGVQFR--YT-----------DPDT------YTSAGHLSCWYQTSL
UniRef90_G0Y2E0_419_588  VPWISQTAYR--FN-----------QPSTSADGNVLTYRGYISVFYQTAV
UniRef90_G9K138_332_516  VPWVSASHYR--NT-----------TPDT------YSQA-----------
UniRef90_A5GZE9_336_557  VPWVSASHFR--YT-----------TPDN------YSSAGFITCWYQTNF
UniRef90_B9V434_332_553  IPWVSASHFR--NT-----------TPDT------YSKAGFVTCWYQTNF
UniRef90_P04936_332_552  VPWISASHYR--NT-----------SPGR-------STSGYITCWYQTRL
UniRef90_C7DUC8_336_557  VPWVSASHFR--YT-----------SRDE------HSAAGFITCWYQTNF
UniRef90_G8IQ74_332_548  VPWISSHFYR--RT-----------KRDD------YTDGGYVTLWLQTAF
UniRef90_I2CMI9_334_554  IPWISSGFYR--NT-----------KSDD------HTEAGYVSLWYQTDF
UniRef90_B9V466_338_522  VPWVSSSHFR--YT-----------SRDE------HSAA-----------
UniRef90_A5GZF9_333_554  VPWVSASHFR--NT-----------TPDT------YSLAGYVTCWYQTNL
UniRef90_G8IJE2_332_552  VPWISASHYR--NT-----------SPDI-------SSSGYITCWYQTGL
UniRef90_H8Y6Q0_168_390  VPWISQGYYR--HT-----------KADK------YTEAGFVTLWYQTDF
UniRef90_G0YPI0_327_543  VPWISNTHFR--TV-----------KTGG---YLDYYATGVVTIWYQTNF
UniRef90_B9V473_333_554  VPWISASNYR--FT-----------TPDA------YSQAGFITCWYQTNF
UniRef90_B5BSU6_368_556  VPWISQSHYR--LV-----------QQDE------YSAAGFISGWYQTNI
UniRef90_A8S322_365_548  VPWMSGTFHR--RT-----------KADN------YTQAGYVSLWYQTDF
UniRef90_G8IQ62_334_555  VPWVSASHFR--NT-----------TPDT------YSLAGYITCWYQTNL
UniRef90_B9V453_331_552  IPWISASHFR--NT-----------TPDT------YSKAGYVTCWYQTNF
UniRef90_G9K146_333_520  VPWVSASHFR--NT-----------TPDT------YSLAGYV--------
UniRef90_F6M9Z0_330_551  VPWVSASHFR--NT-----------TPDK------YSLAGYITCWYQTNL
UniRef90_P12916_334_555  VPWVSASHFR--LT-----------ANDK------YSMAGYITCWYQTNL
UniRef90_G5EN04_327_500  IPWISNTHF-----------------------------------------
UniRef90_C7DUC6_357_551  IPWMSSGFYR--RT-----------KSDE------YTHAGYVSLWYQTDF
UniRef90_Q9WN78_367_554  VPWISQTNYR--YV-----------TRDA------YTDAGYITCWYQTSI
UniRef90_G0YPI1_326_499  IPWISNSHY-----------------------------------------
UniRef90_G0YPH9_326_499  VPWISNTHY-----------------------------------------
UniRef90_Q8UY33_326_552  IPWISNTHYR--TQ-----------ATNN---YFEYYTSGIVTLWYQTNY
UniRef90_A5GZE0_334_555  VPWVSASHYR--NT-----------TPDT------YSSAGYITCWYQTNL
UniRef90_G0ZNT7_334_555  VPWISSGFYR--NT-----------KSDN------HTEAGFVSLWHQTDF
UniRef90_A7KC11_332_515  VPWVSASHYR--NT-----------TPDK------FSQA-----------
UniRef90_G1JYY7_238_455  IPFISMSQYR--TT-----------TN----TTGFLSSAGTLSLWYQTSI
UniRef90_H6UKU5_326_499  IPWISNTHY-----------------------------------------
UniRef90_Q03053_367_554  IPWISQTHYR--YV-----------VVDE------YTAGGYITCWYQTNI
UniRef90_Q995Z1_365_551  VPWISQTHYR--YV-----------QSDT------YTDAGFVTCWYQTSI
UniRef90_B9V442_331_555  VPWISASHYR--NT-----------TPDK------YSSAGYITCWYQTNL
UniRef90_G1CSA8_329_506  IPWISNSHF-----------------------------------------
UniRef90_A5GZE4_333_554  VPWVSASHFR--NT-----------TPDT------YSLAGFITCWYQTNL
UniRef90_G8IJF4_357_552  VPWLSSGFYR--RT-----------ESDD------YTHAGYVSLWHQTDF
UniRef90_A5GZF2_336_558  VPWVSASHFR--FT-----------TPDT------YSAAGYITCWYQTNF
UniRef90_A5GZF1_333_554  VPWVSASHFR--NT-----------TPDT------YSLAGYVTCWYQTNL
UniRef90_Q5U904_326_555  IPWISNTHYR--AM-----------GSND---YFDYYSAGIVTIWYQTNF
UniRef90_Q6JKR7_325_551  IPWISNTHYR--AQ-----------SKNQ---YFDYYSSGIISIWYQTNF
UniRef90_G8IJF0_360_553  VPWISSNFYR--RT-----------KSDS------YTEAGYVSLWYQTDF
UniRef90_A1E4A3_319_512  IPWISGSHYR--MF-----------NNDA---KSTNANVGYVTCF-----
UniRef90_Q8QUZ6_317_541  VPWISGGYFR--GV-----------ARTE---GYSYYNAGYITGWYQTNL
UniRef90_Q2LL04_324_545  VPWISASHFR--GV-----------SVDDTL-NYQYYAAGYVTMWYQTNM
UniRef90_F6M9Y7_364_585  VPWVSASHFR--NT-----------TPDT------YSLAGYITCWYQTNL
UniRef90_Q8V601_326_552  IPWISNTHFR--GT-----------STNG---VYNYYSSGIVTIWYQTNF
UniRef90_Q6JKS0_326_499  IPWISNTHF-----------------------------------------
UniRef90_B3SRP1_326_550  IPWISNTHFR--TN-----------ASGT---YFDYYTAGIVTIWYQTNF
UniRef90_G5CZJ4_357_550  APWISSTFFR--RT-----------KADK------YSSGGIITLWHQTAF
UniRef90_B5BSU7_367_555  VPWISQTHYR--QV-----------KTDV------YTSAGYISGWYQTSI
UniRef90_G8IRN3_333_554  VPWIAQGYYR--HT-----------KSNH------YTEAGYITLWYQTDF
UniRef90_A5GZE2_333_554  VPWVSASHFR--NT-----------TPDT------FSSAGYISCWYQTNL
UniRef90_A7KC08_333_554  VPWVSASHFR--NT-----------TPDK------YSLAGYITCWYQTNL
UniRef90_H9BP13_325_500  VPWISNTHY-----------------------------------------
UniRef90_Q80GI2_332_555  VPWISQTHYR--LV-----------DQDE------YTAAGFVSGWYQTNL
UniRef90_B9V435_333_554  VPWVSASHFR--NT-----------TPDK------YSLAGFITCWYQTNL
UniRef90_Q5G9Z5_326_554  IPWISNTHFR--TQ-----------ATGG---YFDYYTAGIVTIWYQTNF
UniRef90_Q9YLG5_370_557  VPWISQTHYR--YT-----------VKDE------YTDSGYITCWYQTNV
UniRef90_G8IJE5_332_553  VPWVSASHYR--NT-----------TPDT------YSLAGFITCWYQTKL
UniRef90_Q8V614_326_499  VPWISNTHF-----------------------------------------
UniRef90_Q5GA06_355_555  IPWISNTHYR--AH-----------GDGG---YFDYYSTGIVTIWYQTNY
UniRef90_G8IJG6_332_548  IPWISAHNYR--RT-----------KADK------FTEGGYVSVWYQTTF
UniRef90_Q05ED6_367_555  VPWISQTHYR--FV-----------HEDV------YTSAGYVTAWYQTTI
```

```
UniRef90_P23008_310_531   VPWVSASHFR--LT-----------ADNK------YSMAGYITCWYQTNL
UniRef90_Q8JRP9_365_551   IPWISQTHYR--YV-----------QSDA------YTDAGFVTCWYQTSI
UniRef90_Q05EE3_367_555   VPWISQTHYR--FV-----------HDNP------YTSAGYITAWYQTTM
UniRef90_Q8V604_326_552   IPWISNTHYR--TQ-----------ATNN---YFEYYSSGIVTIWYQTNY
UniRef90_C7DUC7_331_549   APWISSTYFR--RT-----------KADK------YSSGGIISLWYQTDF
UniRef90_P12915_324_545   IPWISASHFR--GV-----------SNDDVL-NYQYYAAGHVTIWYQTNM
UniRef90_G8IJE1_331_509   VPWVSASHFR--NT-----------TPD----------------------
UniRef90_A4UHT9_332_548   VPWISSHFFR--RT-----------RKDN------YTESGFVTLWHQTAF
UniRef90_Q9QCF0_367_554   VPWISQTHYR--LV-----------QQDE------YTSAGYVTCWYQTSL
UniRef90_Q9QF31_325_500   VPWISNTHY-----------------------------------------
UniRef90_Q65900_325_551   IPWISNTHYR--AH-----------AKDG---YFDYYTTGTITIWYQTNY
UniRef90_E5D8F2_334_553   VPWISSGFYR--RT-----------KADS------FTHGGYVSLWYQTAF
UniRef90_A1E4A2_319_512   IPWISGSHFR--MF-----------NSDA---KSINANVGYVTCF-----
UniRef90_B9V440_332_553   VPWVSASHYR--NT-----------TPDT------FSLAGYITCWYQTNL
UniRef90_Q6W9F4_332_507   VPWISQTHY-----------------------------------------
UniRef90_G8IJH5_359_554   VPWVSASHFR--NT-----------TPDT------YSLAGFITCWYQTNL
UniRef90_B1PZZ4_333_555   IPWISQTHYR--LV-----------QQDE------YTSAGNVTCWYQTGI
UniRef90_O89280_367_554   IPWISQTHYR--FV-----------VADE------YTAGGFITCWYQTNV
UniRef90_P32537_321_545   IPWISGSHYR--MF-----------NTDA---KAINANVGYVTCFMQTNL
UniRef90_G0YPI2_326_499   IPWISNTHF-----------------------------------------
UniRef90_Q9YLJ1_368_556   VPWISQTHYR--MV-----------QQDE------YSAAGYITCWYQTNI
UniRef90_Q2YEN8_367_554   IPWISQTHYR--LV-----------QQDE------YTSAGNVTCWYQTGI
UniRef90_A7YF31_331_554   VPWISQSHYR--MV-----------HKDA------YTTAGYVTCWYQTNI
UniRef90_Q65481_317_544   VPWISSGHFR--AA-----------DINATLFKYKYYEAGFVTMWYQTNL
UniRef90_Q2LKZ0_317_544   VPWISSGHFR--GT-----------TLENTIYKYRYYERGYITMWYQTNM
UniRef90_Q05ED0_367_555   VPWISQTHYR--LV-----------KQDQ------YSAAGFITCWYQTNI
UniRef90_Q8V5Z9_323_547   IPWISNVMFR--NL-----------QSIA---GQPYFYAGIVTMWYQTNF
UniRef90_Q6W9F9_367_555   VPWISQTHYR--LV-----------EHDE------YTSAGYVTCWYQTSI
UniRef90_H8Y6P9_356_549   VPWMSAGFFR--HT-----------MKDK------YSSGGYITLWRQTDF
UniRef90_G5DC71_332_548   IPWISSHYYR--RT-----------KKDS------YTEGGFVTLWHQTAF
UniRef90_C6ZEZ9_341_560   APWMSSTFYR--RT-----------KGDA------YTSGGYITLWYQTDF
UniRef90_Q05ED4_367_554   VPWISQTHYR--LV-----------DQDA------YTDAGNVTCWYQTGV
UniRef90_H6VBU4_358_522   IPYISPTYWR--PTLGFGNHNVRDNTNQPKPAQTTLDSLGVVSVWVYTRF
UniRef90_Q8V398_325_495   IPWIS---------------------------------------------
UniRef90_D8VCT6_342_524   APWISNTDYR--RS-----------TKDD---------------------
UniRef90_D9YV24_514_704   IPYFSSTPWK--TL-----------SRSTSLSLSVNQLAGWLGLYVYSPL
UniRef90_Q66577_367_555   VPWISQTHYR--LV-----------AQDE------YTSAGYITCWYQTNI
UniRef90_A0MHB7_357_547   IPWISSHFFR--RT-----------RKDN------FTEGGFVTLWHQTAF
UniRef90_E9LS20_360_544   VPWISSGFYR--RT-----------KADN------YTEGGYVSLWYQTDF
UniRef90_Q9WHH4_367_554   IPWISQAHYR--FV-----------EKDP------YSAAGYVTCWYQTGV
UniRef90_G0ZNT5_357_550   APWISSTFFR--RT-----------KSDE------YTSGGIISLWHQTDF
UniRef90_Q5YJA3_454_673   VPYVSPYDFSPVQTPYTKTDFPLVSTNVPITVDTPYWSKGYVTIWQYTNL
UniRef90_H9U0H0_456_678   VPYVSPYDFTPVRTSYTRTEIPPVQTTPKVLVDTPYWSKGYVSVWQYTNL
UniRef90_Q6W9E8_367_554   VPWISQTHYR--FV-----------KEDS------YTDAGFITCWYQTSI
UniRef90_Q80GI1_367_556   VPWISHTHYR--LV-----------ERDE------YSAAGFITAWYQTNI
UniRef90_C7B298_377_565

2WZR3                     H------EKDGAVIVTVSAGPDFEFRMPISPSRQ-
UniRef90_F1AW55_504_724   H------EKDGAVIVSVSAGPDFEFRMPLSPSRQ-
UniRef90_Q6PMU0_504_724   H------EKDGAVVVSVSAGPDFEFRMPISPSRQ-
UniRef90_Q6PMU2_504_724   H------EKDGALIVTVSAGPDLEFRLPISPSRQ-
UniRef90_Q8V0G8_305_525   H------EKDGAVVVSVSAGPDLEFRLPISPSRQ-
UniRef90_Q6PMT3_503_723   H------DPDSAVLVSVSAGADFELRLPINPATQ-
UniRef90_Q1L748_304_524   H------DPDSAVLVSVSAGTDLEFRLPINPAPQ-
UniRef90_Q6PMT8_504_725   H------SAEAAVVVSVSAGPDLEFRFPIDPVRQ-
UniRef90_B8Y0J7_504_725   H------SAEAAVVVSVSAGPDLEFRFPIDPVRQ-
UniRef90_Q1L757_305_524   H------NAEAAVVVSVSAGPDLEFRFPIDPVRQ-
UniRef90_Q1L752_305_526   H------SAEAAVVVSVSAGPDLEFRFPIDPVRQ-
UniRef90_Q1L754_305_526   H------SAEAAVVVSVSAGPDLEFRFPIDPVRQ-
UniRef90_P49303_505_725   K------AEQDTLVVSVSAGKDFELRLPIDPRSQ-
UniRef90_Q6PMY9_505_723   K------AEGDALVVSVSAGKDFEFRLPVDARRQ-
UniRef90_A2I8Z5_505_724   K------ADGDALVVLASAGKDFDLRLPVDARTQ-
UniRef90_P15072_505_723   K------ADADALVVSASAGKDFELRLPVDARQQ-
UniRef90_Q8JVD0_304_524   H------DPDSAVLVSVSAGADLEFRLPINPVAQ-
UniRef90_H9AY35_497_713   T------AANLAVAVAFSAGPDMEFRFPCEP----
UniRef90_B0Z3W9_528_746   P------SSTSELLVLFSSGRDFQLRCPVEPVKQ-
UniRef90_Q2XP17_533_745   TSTDIAVNSKGRVLVAVSAGPDFSLRHPVDLPDKQ
UniRef90_Q100Y4_324_550   T-FPADVPTQADILVFLSAGLDFCLRFPID-----
UniRef90_Q91LX8_542_768   T-YPANTPHLSDIVVFVSAGKDFSLRFPID-----
UniRef90_A4ZKN2_415_639   T-YPVGCPTNSDILTLVSGGDDFTLRMPISPTK--
```

```
UniRef90_E3TMG9_417_639    T - Y P L G V P N D S H I L T L V S G G D D F T L R M P V T - - - - -
UniRef90_P08544_417_642    T - Y P S G T P T N S D I L T L V S A G D D F T L R M P I S P T K - -
UniRef90_B3VTU7_418_646    T - Y P A N T P P N A D I L T L V S A G D D F T L R M P I S P T K - -
UniRef90_Q66776_543_769    T - F P P N V P T R A D V L V F F S A G S D F C L R F P V D - - - - -
UniRef90_G9FTJ7_414_638    T - Y P A N T P Q S A D I L T L V S G G D D F T L R M P V T P S K - -
UniRef90_Q02472_417_641    T - F P A N V P P S S D I L T L V S A G N D F T L R M P I S P T K - -
UniRef90_P12296_396_619    T - Y P P G C P T S A K I L T M V S A G K D F S L K M P I S P A - - -
UniRef90_C3U5A3_447_672    T - Y P A N T P T H S D I L T L V S G G D D F T L R M P V T P T K - -
UniRef90_G1DFA4_460_684    T - Y P A N T P Q R S D V L V F I S S G Q D M C F R - - - - - - - - -
UniRef90_Q155Z9_448_665    T - L P P D C P Q S P C I L F F A S A G E D Y T L R L P V D - - - - -
UniRef90_B9VIN1_352_561    T - Y P A N T P P N Q H I L V M L S G G S D F S Y R L P I A P P - - -
UniRef90_G8HZ08_715_947    V - Y P P G T P T T S Q I I T T V A A G E D F Q F R V P M D - - - - -
UniRef90_B8XTQ3_353_566    T - Y P P G T P P T Q N V V V M L S A G E D F S Y R N P I S P A - - -
UniRef90_D9D8F3_345_562    T - Y P P N T P P T Q Q I V M M L S A G K D F S F R L P I S P - - - -
UniRef90_I1YLT4_348_565    T - Y P P Q T P P T Q Q I V M M L S G G A D F T Y R L P I S P P - - -
UniRef90_B8XTP9_335_563    T - Y P P G S P P T Q Q I V V M L S A G E D F S Y R L P I S P - - - -
UniRef90_I1YLT7_349_565    T - Y P P N T P P T Q Q I V M M L S G G S D F T Y R L P I P P N - - -
UniRef90_I1YLT6_336_558    T - Y P P N T P P T Q Q I V M M L S A G D D F S Y R L P I H P P - - -
UniRef90_B8XTP8_334_562    T - Y P P S T P P T Q Q I L M L M S A G S D F S Y R L P I S P - - - -
UniRef90_B8XTQ0_353_567    T - Y P P S T P P T Q Q I A V M M S A G A D F S L R L P I S P - - - -
UniRef90_Q5YJ95_381_603    V - V P P G A P T T C Q L V V T I S A A D N F V L R I P - - - - - - -
UniRef90_B8XTQ4_341_566    T - Y P P Q T P P Q Q A I L L M A S A G S D F S Y R L P I N P P - - -
UniRef90_I1YLT5_349_558    T - Y P P N T P P T Q H I V L M L A G G Q D F S Y R L P I S P P - - -
UniRef90_F6IA11_421_636    V - F A P N C P S S A V V L V T A A A G E D L E Y - - - - - - - - - -
UniRef90_G0Z2U8_412_599    V - V P P G A P S T C Q L V V T A S A A D N F V L R I P - - - - - - -
UniRef90_Q5YJA7_377_599    V - V P P G A P T T C Q L V V T V S A A D N F V L R V P - - - - - - -
UniRef90_G0Y2E4_357_574    V - V P P G A P S T C Q I V L L V S A A K D F T L R L - - - - - - - -
UniRef90_Q8B552_395_615    V - V P P N C P S T C Q I I G L V S A S D N F V F R I P - - - - - - -
UniRef90_A7KC13_333_552    V - L P P D T N Q K V F I I G F V S A C P D F K L R L - - - - - - - -
UniRef90_A5GZD6_337_514    - - - - - - - - - - - - - - - - - - - - - - - - - - - - - - - - - -
UniRef90_G5CZJ8_367_551    I - L P P E T N Q T V Y L L G F I S A C P D F K L R L - - - - - - - -
UniRef90_G8HH17_373_575    V - T P P N C P N Q C A L V C F A S A C K D F E M R L P V D - - - - -
UniRef90_G0Y2E3_380_601    I - V P A D T P H T A S V C V L A S V T S D F V M R V P - - - - - - -
UniRef90_B9V482_337_556    I - L P P E T S G T V Y L L S F V S A C P D F K L R L - - - - - - - -
UniRef90_A5GZF6_334_555    I - V P P S T P N S A D I I C F V S G C K D F C L R M - - - - - - - -
UniRef90_A7KC10_335_556    V - V P P G T P N T A N M L C F V S G C K D F C L R M - - - - - - - -
UniRef90_G8IJG2_334_555    I - V P P N T P N S A N I I C F V S A C K D F C L R M - - - - - - - -
UniRef90_D2IW00_332_553    V - V P P C T P K T A D I I C F V S G C K D F C L R M - - - - - - - -
UniRef90_A5GZD9_331_550    V - L P P Q V E Q T V Y M L G F V S A C P D F K L R L - - - - - - - -
UniRef90_A7KC07_334_555    V - V P P G T P N S A N M L C F V S A C K D F C L R M - - - - - - - -
UniRef90_Q672R3_805_1000   V - H P A G V P T T S P I V V Y A S A S N N F T F K C P I D - - - - -
UniRef90_B5A1N0_416_617    V - V P P G A P S T C Q L V C L A S A S D N F V F R I P - - - - - - -
UniRef90_B9V433_331_550    V - L P P Q T Q Q T V Y M L G F I S A C P D F K L R L - - - - - - - -
UniRef90_Q82081_333_552    I - L P P Q T S G Q V Y L L S F I S A C P D F K L R L - - - - - - - -
UniRef90_Q5XLP5_336_557    V - F P P N T P N N A N M I C F V S G C K D F C L R M - - - - - - - -
UniRef90_B9V457_333_554    V - T P P S T P K Y A D I I C F V S G C K D F C L R M - - - - - - - -
UniRef90_G1JYY8_404_619    I - I P V G A P A T C Q V F C T V S A C D N F C F R I P - - - - - - -
UniRef90_F6N707_334_556    V - V P P Q T P R H A D I I C F V S G C K D F C L R M - - - - - - - -
UniRef90_G5D5D9_333_552    I - L P P N T T G Q V H L L S F I S A C P D F K L R L - - - - - - - -
UniRef90_Q82122_332_553    V - V P P N T P N T A E M L C F V S G C K D F C L R M - - - - - - - -
UniRef90_G5D5E2_333_552    I - L P P D T P N E V Y M L S F I S A C P D F K L R L - - - - - - - -
UniRef90_A5GZF5_335_556    V - V A P N T P T T A R M I C F V S G C K D F C L R M - - - - - - - -
UniRef90_G0Y2E2_378_600    V - I P A S V P H S A E I C V L C S A T S D F V M R C P - - - - - - -
UniRef90_P03303_333_552    I - L P P E T T G Q V Y L L S F I S A C P D F K L R L - - - - - - - -
UniRef90_P07210_338_559    V - V P D S T P D N A K M V C M V S A C K D F C L R L - - - - - - - -
UniRef90_F6M9Y3_333_554    V - V P P N T P T T A N I I C F V S G C K D F C L R M - - - - - - - -
UniRef90_B9V459_336_557    V - F P P N T P N S A D M L C F V S A C K D F C L R M - - - - - - - -
UniRef90_B9V477_333_554    I - V A P N T P S T A R M I C F V S G C K D F C L R M - - - - - - - -
UniRef90_H9C6N6_333_552    I - L P P D T A G T V Y L L A F I S A C P D F K L R L - - - - - - - -
UniRef90_A5GZE7_336_557    V - F P P N T P S T A D M L C F V S A C K D F C L R M - - - - - - - -
UniRef90_B9V485_333_552    I - L P P Q T S G T A Y L L S F I S A C P D F K L R L - - - - - - - -
UniRef90_G0Y2E0_419_588    V - V P V G A - - - - - - - - - - - - - - - - - - - - - - - - - - -
UniRef90_G9K138_332_516    - - - - - - - - - - - - - - - - - - - - - - - - - - - - - - - - - -
UniRef90_A5GZE9_336_557    V - F P P N T P S S A D M L C F V S A C K D F C L R M - - - - - - - -
UniRef90_B9V434_332_553    V - T A P D A P T T A D I I C L V S A C K D F C L R M - - - - - - - -
UniRef90_P04936_332_552    V - I P P Q T P P T A R L L C F V S G C K D F C L R M - - - - - - - -
UniRef90_C7DUC8_336_557    V - F P P N T P N R A D M L C F V S A C K D F C L R M - - - - - - - -
UniRef90_G8IQ74_332_548    V - S - - - N D T V G A I M V T C S A C P D M A V R M - - - - - - - -
UniRef90_I2CMI9_334_554    V - T - P T T A G Q G A I L V T C S G C P D I S V R M - - - - - - - -
UniRef90_B9V466_338_522    - - - - - - - - - - - - - - - - - - - - - - - - - - - - - - - - - -
UniRef90_A5GZF9_333_554    V - V P P N T P P N A K M L C F V S G C K D F C L R M - - - - - - - -
UniRef90_G8IJE2_332_552    V - I P P E T P P T A R L L C F V S A C K D F C L R M - - - - - - - -
UniRef90_H8Y6Q0_168_390    V - A - G S T T G Q G A I L V T C S A C P D M A V R M - - - - - - - -
```

```
UniRef90_G0YPI0_327_543   V - V P P D T P N E A N I I A M G A G - - - - - - - - - - - - - - - -
UniRef90_B9V473_333_554   V - V P P N T P K T A N I I C F V S G C K D F C L R M - - - - - - - -
UniRef90_B5BSU6_368_556   I - V P P K T P T D C R V L C F V S A C N D F S V R M - - - - - - - -
UniRef90_A8S322_365_548   V - - - S P N D G I G T I V A T C S G C P D L S V R M - - - - - - - -
UniRef90_G8IQ62_334_555   V - V P P N T P S T A K M L C F V S G C K D F C L R M - - - - - - - -
UniRef90_B9V453_331_552   I - T A P N T P P T A D I I C L V S A C K D F C L R M - - - - - - - -
UniRef90_G9K146_333_520   - - - - - - - - - - - - - - - - - - - - - - - - - - - - - - - - - - -
UniRef90_F6M9Z0_330_551   I - A P P N T P Q T A R M L C F V S A C K D F C L R M - - - - - - - -
UniRef90_P12916_334_555   V - V P P N T P Q T A D M L C F V S A C K D F C L R M - - - - - - - -
UniRef90_G5EN04_327_500   - - - - - - - - - - - - - - - - - - - - - - - - - - - - - - - - - - -
UniRef90_C7DUC6_357_551   I - P - S S Q T S Q G A I L V T C S G C P D L S V R M - - - - - - - -
UniRef90_Q9WN78_367_554   V - T P P D I P T T S T I L C F V S A C N D F S V R L - - - - - - - -
UniRef90_G0YPI1_326_499   - - - - - - - - - - - - - - - - - - - - - - - - - - - - - - - - - - -
UniRef90_G0YPH9_326_499   - - - - - - - - - - - - - - - - - - - - - - - - - - - - - - - - - - -
UniRef90_Q8UY33_326_552   V - V P E G A P T S A Y I I A L G A A Q P N F S L K L - - - - - - - -
UniRef90_A5GZE0_334_555   I - I P P N A A P V A D M I C F V S G C K D F C L R M - - - - - - - -
UniRef90_G0ZNT7_334_555   I - A - T T T T G Q G A I L V T C S G C P D I S V R M - - - - - - - -
UniRef90_A7KC11_332_515   - - - - - - - - - - - - - - - - - - - - - - - - - - - - - - - - - - -
UniRef90_G1JYY7_238_455   V - V P P S C P Q T C S I L C K I A A G E N F C M R I P - - - - - - -
UniRef90_H6UKU5_326_499   - - - - - - - - - - - - - - - - - - - - - - - - - - - - - - - - - - -
UniRef90_Q03053_367_554   V - V P A D T Q S D C K I L C F V S A C N D F S V R M - - - - - - - -
UniRef90_Q995Z1_365_551   V - V P T G V V T Q S T I L C F V S A C N D F S V R L - - - - - - - -
UniRef90_B9V442_331_555   V - V P P N T P T S A K M L C F V S G C K D F C L R M - - - - - - - -
UniRef90_G1CSA8_329_506   - - - - - - - - - - - - - - - - - - - - - - - - - - - - - - - - - - -
UniRef90_A5GZE4_333_554   V - V P P D T P A T A R M L C F V S G C K D F C L R M - - - - - - - -
UniRef90_G8IJF4_357_552   I - P S S S Q V T G G A I L V T C S G C P D L S V R M - - - - - - - -
UniRef90_A5GZF2_336_558   V - V P P N T P R S A K M V C M V S A C K D F C L R L - - - - - - - -
UniRef90_A5GZF1_333_554   V - V P P N T P T T A K M L C F V S G C K D F C L R M - - - - - - - -
UniRef90_Q5U904_326_555   V - V P S G A P T S A Y I I A L A A A Q K N F T L R L P - - - - - - -
UniRef90_Q6JKR7_325_551   V - V P A E T P T T A N I I V L G A A Q P N F T M R L - - - - - - - -
UniRef90_G8IJF0_360_553   I - P - S V N A G T G T I I A T C S A C P D M S V R M - - - - - - - -
UniRef90_A1E4A3_319_512   - - - - - - - - - - - - - - - - - - - - - - - - - - - - - - - - - - -
UniRef90_Q8QUZ6_317_541   V - V P P N F P T S A T I I A L M A A Q P N F S C R V - - - - - - - -
UniRef90_Q2LL04_324_545   V - I P P G F P N T A G I I M L V A A Q P N F S F R I - - - - - - - -
UniRef90_F6M9Y7_364_585   I - A P T N A P Q T A R M L C F V S A C K D F C L R M - - - - - - - -
UniRef90_Q8V601_326_552   V - V P D G A P T S A Y I I A L A A A Q R N F T M R L - - - - - - - -
UniRef90_Q6JKS0_326_499   - - - - - - - - - - - - - - - - - - - - - - - - - - - - - - - - - - -
UniRef90_B3SRP1_326_550   V - V P A G A P T S A Y L I A L G A A Q P N F T M R L - - - - - - - -
UniRef90_G5CZJ4_357_550   V - S - G D T A N V G T I L A T C S G C P D I S V R M - - - - - - - -
UniRef90_B5BSU7_367_555   V - V P P K T P T E C K I L C F V S A C N D F S V R L - - - - - - - -
UniRef90_G8IRN3_333_554   V - P - G S Q G G Q G A V L V T C S A C P D M A V R M - - - - - - - -
UniRef90_A5GZE2_333_554   V - V P P N T P T T A K M L C F V S G C K D F C L R M - - - - - - - -
UniRef90_A7KC08_333_554   V - V P P N T P T T A K M L C F V S G C K D F C L R M - - - - - - - -
UniRef90_H9BP13_325_500   - - - - - - - - - - - - - - - - - - - - - - - - - - - - - - - - - - -
UniRef90_Q80GI2_332_555   I - V P P N T P T S C E I L C F V S A C N D F S V R M - - - - - - - -
UniRef90_B9V435_333_554   V - V P A D T P T T A N L L C F V S A C K D F C L R M - - - - - - - -
UniRef90_Q5G9Z5_326_554   V - V P D G A P T Q A R I I A L G A A Q K N F T L R L - - - - - - - -
UniRef90_Q9YLG5_370_557   I - A P A D A L S T C Y I M C M V S A C N D F S V R M - - - - - - - -
UniRef90_G8IJE5_332_553   V - V P P N T A S T A D M L C F V S G C K D F C L R M - - - - - - - -
UniRef90_Q8V614_326_499   - - - - - - - - - - - - - - - - - - - - - - - - - - - - - - - - - - -
UniRef90_Q5GA06_355_555   V - V P I G A P T S A Y I I A L G A A Q P N F T M R L - - - - - - - -
UniRef90_G8IJG6_332_548   V - S - - - T S L A G C I M V T C S A C P D M A V R M - - - - - - - -
UniRef90_Q05ED6_367_555   V - V P P Q T P T S C K I L C F V S A C N D F S V R M - - - - - - - -
UniRef90_P23008_310_531   V - V P P S T P Q T A D M L C F V S A C K D F C L R M - - - - - - - -
UniRef90_Q8JRP9_365_551   V - V P V G V V A Q S T I L C F V S A C S D F S V R L - - - - - - - -
UniRef90_Q05EE3_367_555   V - V P P K T P T S C Y I L C F V S A C N D F S V R M - - - - - - - -
UniRef90_Q8V604_326_552   V - V P D G A P T S A Y I I A L G A A Q P N F S L K L - - - - - - - -
UniRef90_C7DUC7_331_549   V - G - G S D P T - G T I V A T C S G C P D I S V R M - - - - - - - -
UniRef90_P12915_324_545   V - I P P G F P N T A G I I M M I A A Q P N F S F R I - - - - - - - -
UniRef90_G8IJE1_331_509   - - - - - - - - - - - - - - - - - - - - - - - - - - - - - - - - - - -
UniRef90_A4UHT9_332_548   V - S - - - N G T S G S I M V T C S A C S D M S V R M - - - - - - - -
UniRef90_Q9QCF0_367_554   V - V P P G A P A T C G V L C L A S A C N D F S V R M - - - - - - - -
UniRef90_Q9QF31_325_500   - - - - - - - - - - - - - - - - - - - - - - - - - - - - - - - - - - -
UniRef90_Q65900_325_551   V - V P I G A P T T A Y I V A L A A A Q D N F T M K L - - - - - - - -
UniRef90_E5D8F2_334_553   V - P - P V S G G T G S I L A T C S A C P D M S V R M - - - - - - - -
UniRef90_A1E4A2_319_512   - - - - - - - - - - - - - - - - - - - - - - - - - - - - - - - - - - -
UniRef90_B9V440_332_553   V - T P P N T P S T A R M L C F V S G C K D F C L R M - - - - - - - -
UniRef90_Q6W9F4_332_507   - - - - - - - - - - - - - - - - - - - - - - - - - - - - - - - - - - -
UniRef90_G8IJH5_359_554   V - F P P D T P T T A R M L C F V S G C K D F C L R M - - - - - - - -
UniRef90_B1PZZ4_333_555   V - V P A G T P T S C S I M C F V S A C N D F S V R L - - - - - - - -
UniRef90_O89280_367_554   I - V P L G A Q S N C S I L C F V S A C N D F S V R M - - - - - - - -
UniRef90_P32537_321_545   V - A P V G A A D Q C Y I V G M V A A K K D F N L R L - - - - - - - -
UniRef90_G0YPI2_326_499   - - - - - - - - - - - - - - - - - - - - - - - - - - - - - - - - - - -
```

```
UniRef90_Q9YLJ1_368_556   I - V P P D T P T D C I V L C F V S A C N D F S V R M - - - - - - - -
UniRef90_Q2YEN8_367_554   V - V P P G T P N K C V V L C F V S A C N D F S V R M - - - - - - - -
UniRef90_A7YF31_331_554   V - V P S G T P T E C V V L G F V S A C N D F S V R L - - - - - - - -
UniRef90_Q65481_317_544   V - V P P S F P N Q A A I L M F V A A Q P N F S M R I - - - - - - - -
UniRef90_Q2LKZ0_317_544   V - V P P N F P N H A S I L M F V A A Q P N F S L R I - - - - - - - -
UniRef90_Q05ED0_367_555   I - V P P D T P T R C A M L C F V S A C N D F S V R M - - - - - - - -
UniRef90_Q8V5Z9_323_547   V - V P D G A P T S A D I I A L G S A Q S N F S L R L - - - - - - - -
UniRef90_Q6W9F9_367_555   V - V P P E T P T H C D I L G F I S A C N D F S V R M - - - - - - - -
UniRef90_H8Y6P9_356_549   V - P - G S S N G V G Y I L A T C S G C P D L S V R M - - - - - - - -
UniRef90_G5DC71_332_548   V - S - - - A G V V G A I M V T C S A C S D M S V R M - - - - - - - -
UniRef90_C6ZEZ9_341_560   I - P - S S S G G V G T I L A T C S A C P D L S V R M - - - - - - - -
UniRef90_Q05ED4_367_554   V - V P P N T P T Q C T I L C F V S A C N D F S V R L - - - - - - - -
UniRef90_H6VBU4_358_522   Q T P S A N N T V P I D F I P F L S A G K G F S F R L P T A P T - - -
UniRef90_Q8V398_325_495   - - - - - - - - - - - - - - - - - - - - - - - - - - - - - - - - - - -
UniRef90_D8VCT6_342_524   - - - - - - - - - - - - - - - - - - - - - - - - - - - - - - - - - - -
UniRef90_D9YV24_514_704   S - C P A F N P D Q A T I A V F I R G G A D F E A R I P - - - - - - -
UniRef90_Q66577_367_555   V - V P P E T P S D C V V L C F V S A C N D F S V R M - - - - - - - -
UniRef90_A0MHB7_357_547   V - S - - - N G Q V G S I M V T C S A C S D M S V R M - - - - - - - -
UniRef90_E9LS20_360_544   I - P - S N G T S A G S I V A T C S - - - - - - - - - - - - - - - - -
UniRef90_Q9WHH4_367_554   V - S P A G N Q P K C Y I M C F V S A C N D F S V R L - - - - - - - -
UniRef90_G0ZNT5_357_550   V - T - G S T T T V G T I L A T C S G C P D I S V R M - - - - - - - -
UniRef90_Q5YJA3_454_673   A - S P P N S P S Q A D F L V F V F A G E D Y V L K G P - - - - - - -
UniRef90_H9U0H0_456_678   A - S P P G S P S Q A D F L L F V F A G K D Y V L K G P S S - - - - -
UniRef90_Q6W9E8_367_554   V - T P P S T Q S K C F I M C L V S A C N D F S V R L - - - - - - - -
UniRef90_Q80GI1_367_556   I - V P P G A S P H C Y T L C F A S A C N D F S V R M - - - - - - - -
UniRef90_C7B298_377_565
```

1	2	3	4	5	6	7	8	9
Variable				Average				Conserved

X - Insufficient data - the calculation for this site was performed on less than 10% of the sequences.

Supplementary Tables:
Table S1

Residue	CASTp	DEPTH	DoGSiteScorer	fpocket	GHECOM	LIGSITE	PDBinder	Pocket-finder	Q-SiteFinder	SiteHound-web	Number of tools
Viral particle 1 (VP1)											
GLY7	√	√	√	√	√	-	√	√	√	√	9
ALA8	√	√	√	√	√	-	√	√	√	√	9
ASP9	√	√	√	√	√	-	√	√	√	√	9
PRO10	√	-	√	√	√	-	√	√	√	√	8
VAL51	√	√	√	√	√	-	√	√	√	√	9
ARG68	√	√	√	-	√	√	-	√	-	√	7
TYR72	-	√	√	√	√	-	-	√	√	√	7
TRP90	-	√	√	√	√	-	√	√	-	√	7
THR91	-	√	√	√	√	-	√	√	√	√	8
PRO92	-	√	√	√	√	-	√	√	√	√	8
ASN93	-	√	√	√	√	-	√	√	√	√	8
GLY94	-	√	√	√	√	-	√	√	√	√	8
SER95	-	√	√	√	√	-	√	√	√	√	8
PRO96	√	√	√	√	√	-	√	√	√	√	9
VAL97	√	√	√	√	√	-	√	√	√	√	9
PRO105	-	√	√	√	√	-	√	√	-	√	7
VAL106	√	√	√	√	√	-	√	√	-	√	8
VAL107	-	√	√	√	√	-	√	√	√	√	8
ARG115	√	√	√	√	√	-	√	√	√	√	9
PHE116	-	√	√	√	√	-	√	√	√	√	8
ALA117	-	√	√	√	√	√	√	√	√	√	9
LEU118	-	√	√	√	√	-	√	√	-	√	7
PRO119	-	√	√	√	√	-	√	√	-	√	7
TYR120	-	√	√	√	√	-	√	√	√	√	8
ALA122	-	√	√	√	√	-	√	√	-	√	7
PRO123	-	√	√	√	√	-	√	√	-	√	7
HIS124	-	√	√	√	√	-	√	√	√	√	8
ARG125	-	√	√	√	√	-	√	√	√	√	8
VAL126	-	-	√	√	√	-	√	√	√	√	7
VAL130	√	√	√	-	√	-	√	√	-	√	7
TYR131	√	√	√	√	√	-	√	√	-	-	7
ASP134	√	√	√	√	√	-	√	√	-	-	7
THR167	√	√	√	√	√	-	√	√	√	√	9
ASN170	√	√	√	√	√	-	√	√	√	√	9
GLY172	-	√	√	√	√	-	√	√	√	√	8
MET173	√	√	√	√	√	-	√	√	√	√	9
TYR175	-	√	√	√	√	-	√	√	√	√	8
ARG188	√	-	√	√	√	-	√	√	√	-	7
ALA189	√	√	√	√	√	-	√	√	-	-	7
GLU190	√	-	-	√	√	√	√	√	√	√	8
LEU191	-	√	√	√	√	√	√	√	√	-	8
TYR192	√	√	√	√	√	√	√	√	√	√	10
CYS193	-	-	√	√	√	-	√	√	√	√	7
ARG195	√	√	√	-	√	-	√	√	√	√	8
PRO196	√	√	√	-	√	√	√	√	-	√	8
Viral particle 2 (VP2)											
VAL30	√	√	√	√	√	-	-	√	-	√	7
GLY31	√	√	√	√	√	-	-	√	-	√	7
VAL32	√	√	√	√	√	-	-	√	√	√	8
THR33	-	√	√	√	√	-	-	√	√	√	7
TYR34	√	√	√	√	√	-	-	√	√	√	8
GLY35	-	√	√	√	√	-	-	√	√	√	7
TYR36	√	√	√	√	√	-	-	√	√	√	8
LEU38	√	√	√	√	√	-	-	√	-	√	7
PRO46	√	√	√	√	√	-	-	√	√	-	7
PRO84	-	√	√	√	√	-	-	√	√	√	7
THR85	√	√	√	√	√	-	-	√	-	√	7
HIS87	√	√	√	√	√	-	-	√	-	√	7
TYR91	-	√	√	√	√	-	-	√	√	√	7
TRP105	-	√	√	√	√	-	-	√	√	√	7
GLN108	√	√	√	√	√	-	-	√	√	-	7
GLU136	√	√	√	-	√	√	-	√	-	√	7
THR141	√	√	√	√	√	-	-	√	√	√	8
LEU142	√	√	√	√	√	-	-	√	√	√	8
PHE143	-	√	√	√	√	-	-	√	√	√	7
PRO144	-	√	√	√	√	-	-	√	√	√	7
HIS145	√	√	√	√	√	-	-	√	√	√	8
ASN153	√	√	√	√	-	-	√	√	√	-	7
TYR162	-	√	√	√	√	-	-	√	√	√	7
LEU163	-	√	√	√	√	-	-	√	√	√	7
GLY164	-	√	√	√	√	-	-	√	√	√	7
THR172	√	√	√	√	√	-	-	√	√	√	8
Viral particle 3 (VP3)											
GLY10	-	√	√	√	√	-	-	√	√	√	7
TYR11	√	√	√	√	√	-	-	√	√	√	8
GLY12	√	√	√	√	√	-	-	√	√	√	8
GLN15	-	√	√	√	√	-	-	√	√	√	7
THR17	√	√	√	√	√	-	-	√	-	√	7
PRO19	√	√	√	√	√	-	-	√	-	√	7
LEU74	√	√	√	√	√	-	-	√	-	√	7
TYR111	√	√	√	√	-	-	-	√	√	√	7
THR150	√	√	√	√	-	-	√	√	√	√	8
GLY151	√	√	√	√	-	-	√	√	√	√	8
SER154	√	√	√	√	-	-	√	√	√	√	8
TYR162	-	√	√	√	√	-	√	√	√	-	7
ASP167	-	√	√	√	√	-	-	√	√	√	7
TYR168	√	√	√	√	√	-	-	√	√	√	8
TYR170	-	√	√	√	√	-	-	√	√	√	7
TYR172	√	√	√	√	√	-	-	√	√	√	8
ALA173	√	√	√	√	√	-	-	√	√	√	8
ASP174	√	√	√	√	√	-	-	√	√	√	8
GLU177	√	√	√	√	√	-	-	√	√	-	7
Viral particle 4 (VP4)											
TYR30	√	√	√	√	√	√	√	-	-	√	8
GLN31	√	√	√	√	√	√	√	-	-	√	8
ASN65	√	√	√	√	√	-	√	-	-	√	7
TRP67	√	√	√	√	√	-	√	-	-	√	7
LYS70	√	√	√	√	√	-	√	-	-	√	7
LEU71	√	√	√	√	√	-	√	-	-	√	7

Supplementary References:

1 Goldenberg, O., Erez, E., Nimrod, G. & Ben-Tal, N. The ConSurf-DB: pre-calculated evolutionary conservation profiles of protein structures. *Nucleic Acids Research* **37**, D323-D327, (2009).

2 Angermüller, C., Biegert, A. & Söding, J. Discriminative modelling of context-specific amino acid substitution probabilities. *Bioinformatics* **28**, 3240-3247, (2012).

3 Biegert, A. & Söding, J. Sequence context-specific profiles for homology searching. *Proceedings of the National Academy of Sciences* **106**, 3770-3775, (2009).

4 Katoh, K., Misawa, K., Kuma, K.-i. & Miyata, T. MAFFT: a novel method for rapid multiple sequence alignment based on fast Fourier transform. *Nucleic Acids Research* **30**, 3059-3066, (2002).

5 Pupko, T., Bell, R. E., Mayrose, I., Glaser, F. & Ben-Tal, N. Rate4Site: an algorithmic tool for the identification of functional regions in proteins by surface mapping of evolutionary determinants within their homologues. *Bioinformatics* **18**, S71-S77, (2002).
